# Rigbox: An Open-Source Toolbox for Probing Neurons and Behavior

**DOI:** 10.1523/ENEURO.0406-19.2020

**Published:** 2020-07-09

**Authors:** Jai Bhagat, Miles J. Wells, Kenneth D. Harris, Matteo Carandini, Christopher P. Burgess

**Affiliations:** 1 UCL Queen Square Institute of Neurology, University College London, London WC2N 5DU, United Kingdom; 2 UCL Institute of Ophthalmology, University College London, London EC1V 9EL, United Kingdom

**Keywords:** behavioral, control, experimental, software, toolbox

## Abstract

Setting up an experiment in behavioral neuroscience is a complex process that is often managed with ad hoc solutions. To streamline this process, we developed Rigbox, a high-performance, open-source software toolbox that facilitates a modular approach to designing experiments (https://github.com/cortex-lab/Rigbox). Rigbox simplifies hardware input-output, time aligns datastreams from multiple sources, communicates with remote databases, and implements visual and auditory stimuli presentation. Its main submodule, Signals, allows intuitive programming of behavioral tasks. Here we illustrate its function with the following two interactive examples: a human psychophysics experiment, and the game of Pong. We give an overview of running experiments in Rigbox, provide benchmarks, and conclude with a discussion on the extensibility of the software and comparisons with similar toolboxes. Rigbox runs in MATLAB, with Java components to handle network communication, and a C library to boost performance.

## Significance Statement

Configuring the hardware and software components required to run a behavioral neuroscience experiment and manage experiment-related data are a complex process. In a typical experiment, software is required to design a behavioral task, present stimuli, read hardware input sensors, trigger hardware outputs, record subject behavior and neural activity, and transfer data between local and remote servers. Here we introduce Rigbox, which, to the best of our knowledge, is the only software toolbox that integrates all of the aforementioned software requirements necessary to run an experiment. This MATLAB-based package provides a platform to rapidly prototype experiments. Multiple laboratories have adopted this package to run experiments in cognitive, behavioral, systems, and circuit neuroscience.

## Introduction

In behavioral neuroscience, much time is spent setting up hardware and software, and ensuring compatibility between them. Experiments often require configuring disparate software to interface with distinct hardware, and integrating these components is no trivial task. Furthermore, there are often separate software components for designing a behavioral task, running the task, and acquiring, processing, and logging the data. This requires learning the fundamentals of different software packages and how to make them communicate appropriately.

Consider a typical experiment focused on decision-making, in which a subject chooses a stimulus among a set of possibilities and obtains a reward if the choice was correct (Carandini and Churchland, 2013). The software setup for this experiment may seem simple: ostensibly, all that is required is software to run the behavioral task, and software to handle experiment data. However, when considering implementation details for these two types of software, the setup can grow quite complex. Running the behavioral task requires software for starting, stopping, and transitioning between task states, presenting stimuli, reading input devices, and triggering output devices. Handling experiment data requires software for acquiring, processing, and logging stimulus history, response history, and subject physiology, and transferring data between servers and databases.

To address this variety of needs in a single software toolbox, we designed Rigbox (github.com/cortex-lab/Rigbox). Rigbox is modular, high-performance, open-source software for running behavioral neuroscience experiments and acquiring experiment-related data. Rigbox facilitates acquiring, time aligning, and managing data from a variety of sources. Furthermore, Rigbox allows users to programmatically and intuitively design and parametrize behavioral tasks via a framework called Signals.

We begin by giving a general overview of Signals, the core package of Rigbox. We illustrate the following two simple interactive examples of its use: an experiment in visual psychophysics, and the game of Pong. Next, we describe how Rigbox runs Signals experiments and manages experiment data. We then discuss the design considerations of Rigbox and the various types of experiments that have been implemented using Rigbox. Last, we detail the requirements of Rigbox and provide benchmarking results. If you wish to try out the code examples used in this paper, please install Rigbox by following the information in the github repository README file (http://github.com/cortex-lab/Rigbox).

## Signals

**Figure 1.**
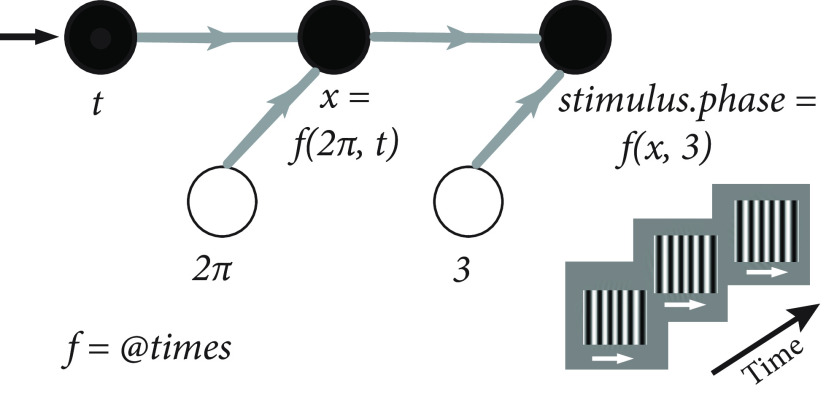
A representation of the time-dependent phase of a visual stimulus in Signals using a clock signal, *t*. *t* represents time in seconds since experiment start (its value therefore constantly increases). An unfilled circle represents a constant value: it becomes a node in the network when combined with another signal in an operation (in this instance, via multiplication, represented by the MATLAB function, times). The bottom right shows how the phase of the grating changes over time: the white arrow indicates the phase shift direction.

Signals is a framework designed for building bespoke behavioral tasks. In Signals, an experiment is built from a reactive network whose nodes (“signals”) represent experiment parameters. This simplifies problems that deal with how experiment parameters change over time by representing relationships between these parameters with straightforward, self-documenting operations. For example, to define a drifting grating, a user could create a signal that changes the phase of a grating as a function of time (Fig. 1). This is shown in the following code below:

theta = 2 * π; % angle of phase in radians

freq = 3; % frequency of phase in Hz

stimulus.phase = theta * freq * t; % phase that cycles at 3 Hz for given stimulus

Whenever the clock signal, *t*, is updated (e.g., by a MATLAB timer callback function), the values of all its dependent signals are then recalculated asynchronously via callbacks. This paradigm is known as functional reactive programming (see D. Lew, “An Introduction to Functional Reactive Programming,” https://blog.danlew.net/2017/07/27/an-introduction-to-functional-reactive-programming/).

**Figure 2.**
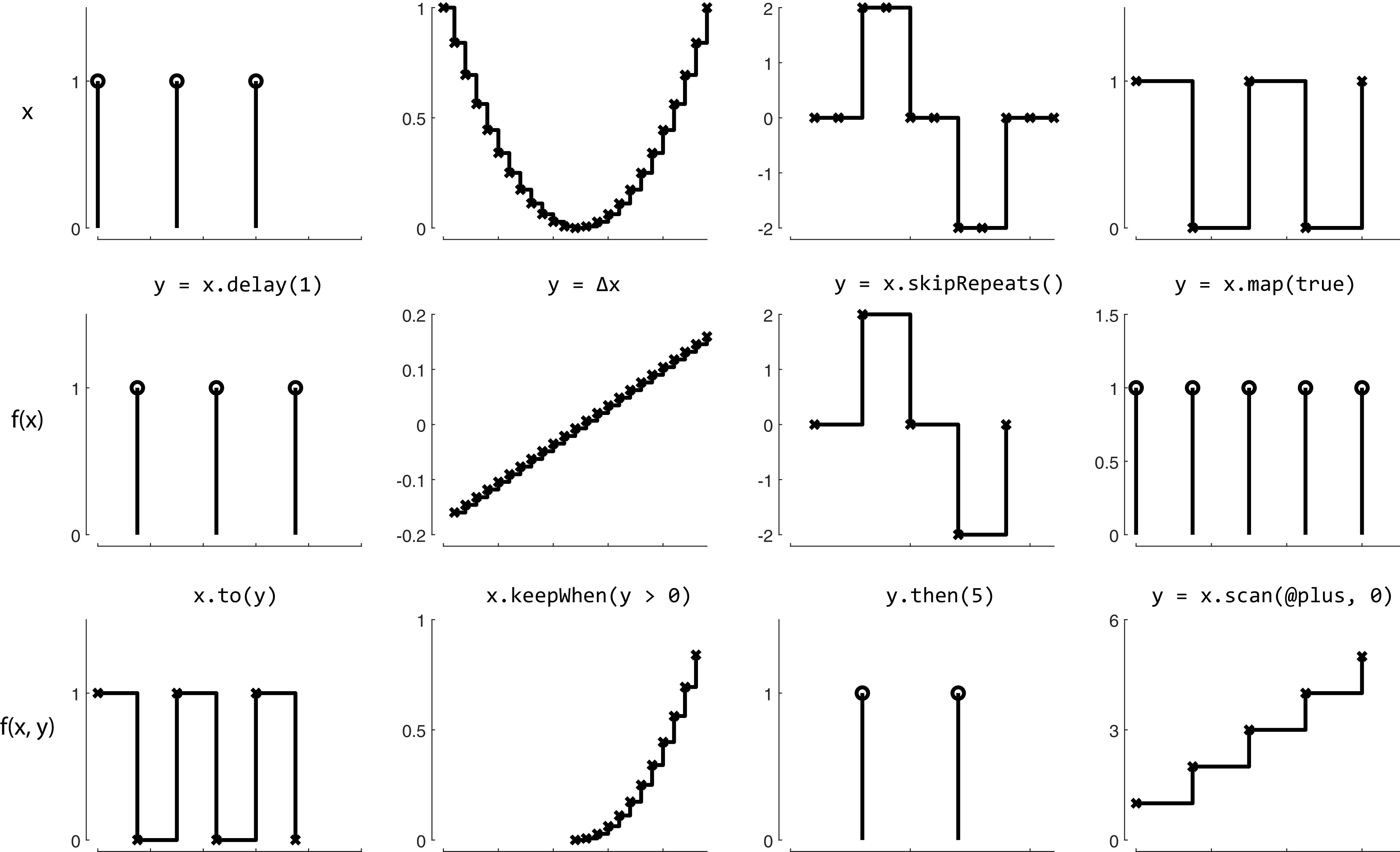
The creation of new signals via example signals methods. Each panel, in which the *x*-axis represents time and the *y*-axis represents value, contains a signal. Each column depicts a set of related transformations. The top row contains four arbitrary signals. The second row depicts a signal that results from applying an operation on the signal in the panel above. The third row depicts a signal that results from applying an operation on the signals in the two panels above. Conceptually, each signal can be thought of as both a continuous stream of discrete values, and as a discrete representation whose value changes over time.

The operations that can be performed on signals are not just limited to basic arithmetic. Many built-in MATLAB functions (including logical, trigonometric, casting, and array operations) have been overloaded to work on signals as they would on basic numeric or char types. Furthermore, a number of classical functional programming functions (e.g., “map” and “scan”) can be used on signals (Fig. 2). These allow signals to gate, trigger, filter, and accumulate other signals to define a complete experiment.

### Example 1: a psychophysics experiment

**Figure 3.**
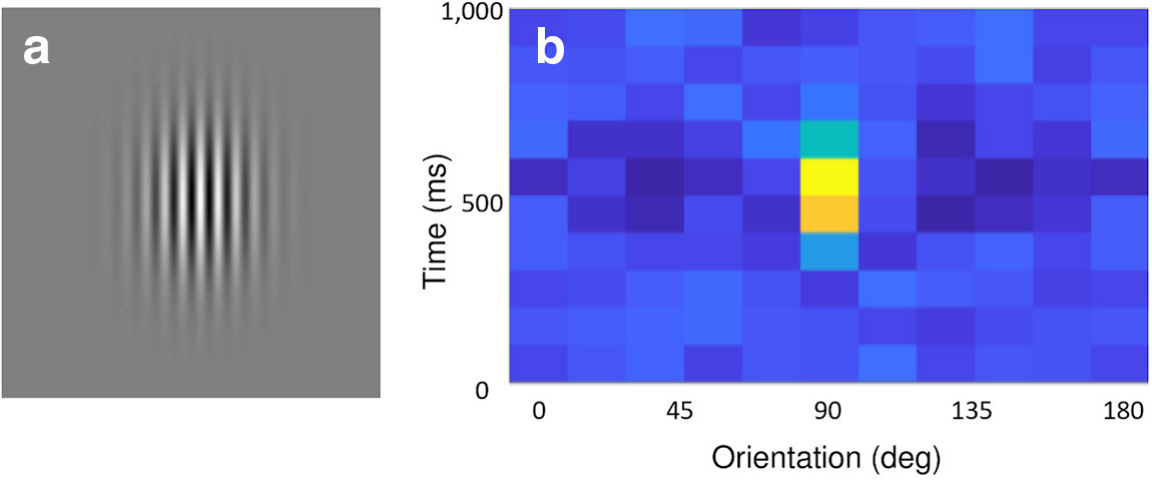
Output shown when running “ringach98.m” ***a***, A sample grating that the subject is required to respond to via a “ctrl” key press. ***b***, A heatmap showing the grating orientations for the 10 frames immediately preceding the key press, summed over all of the key presses for the duration of the experiment. After a few minutes, the distribution of orientations that were presented at each key press resembles a 2D Mexican Hat wavelet, centered on the orientation the subject was reporting at the subject’s average reaction time. In this example, the subject was reporting a vertical grating orientation (90°) with an average reaction time of ∼600 ms.

**Figure 4.**
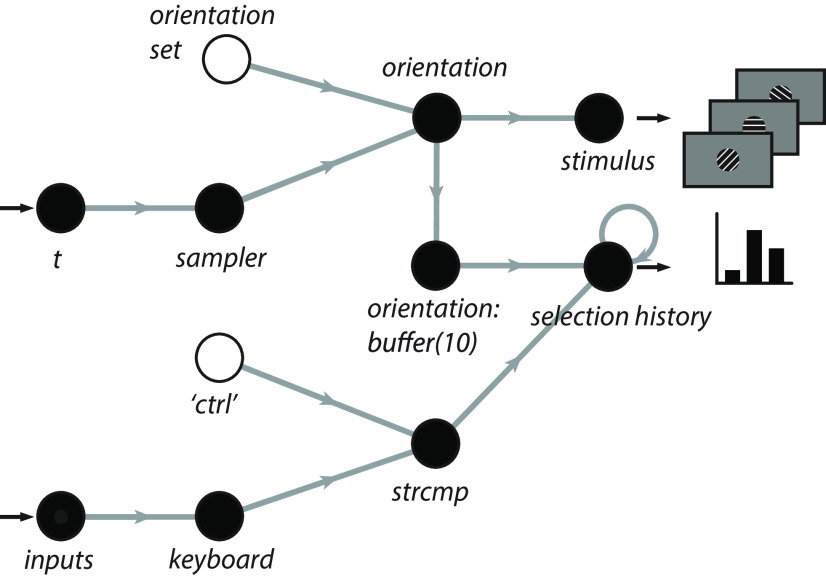
A simplified Signals network diagram of the Ringach experiment. Each circle represents a node in the network that carries out an operation on its direct input. The left-most nodes are inputs to the network, and the values from the right-most layer are used to update the stimulus and the histogram plot. An unfilled circle represents a constant value.

Our first example of a human-interactive Signals experiment is a script that recreates a psychophysics experiment to study the mechanisms that underlie the discrimination of a visual stimulus (Ringach, 1998). In this experiment, the observer looks at visual gratings (Fig. 3*a*) that change rapidly and randomly in orientation and phase. The gratings change so rapidly that they summate in the visual system, and the observer tends to perceive two or three of them as superimposed. The task of the observer is to hit the “ctrl” key whenever the orientation of the grating is vertical. At key press, the probability of detection is plotted as a function of stimulus orientation in the recent past. Typically, this exposes a center-surround type of organization, with orientations near vertical eliciting responses, but orientations further away suppressing responses (Fig. 3*b*). The Signals network representation of this experiment is shown in Figure 4.

To run this experiment, simply run ringach98 script https://github.com/cortex-lab/signals/blob/master/docs/examples/ringach98.m after installing Rigbox and press the “Play” button. Below is a breakdown of the 30-odd lines of code:

First, some constants are defined:

oris = 0:18:162; % set of orientations, deg

phases = 90:90:360; % set of phases, deg

presentationRate = 10; % Hz

winlen = 10; % length of histogram window, frames

Next, we create a figure:

figh = figure(‘Name’, ‘Press “ctrl” key on vertical grating’,…

‘Position’, [680 250 560 700], ‘NumberTitle’, ‘off’);vbox = uix.

VBox(‘Parent’, figh); % container for the play/pause button and axes% Create axes for the histogram plot.

axh = axes(‘Parent’, vbox, ‘NextPlot’, ‘replacechildren’, ‘XTick’, oris);

xlabel(axh, ‘Orientation’);

ylabel(axh, ‘Time (frames)’);

ylim([0 winlen] + 0.5);

vbox.Heights = [30 −1]; % 30 px for the button, the rest for the plot

Next, we create our Signals network. The function playgroundPTB creates a new Signals network and one input signal, *t*. It creates a start button, which, when pressed, starts a MATLAB timer that periodically updates *t* with the time. Finally, it returns an anonymous function, setElemsFn, that, when called with a visual stimulus object, adds the textures to a stimulus renderer:

% Create a new Psychtoolbox stimulus window and renderer, returning a timing

% signal, ‘t’, and function, ‘setElemsFn’, to load the visual elements.

[t, setElemsFn] = sig.test.playgroundPTB(vbox);

net = t.Node.Net; % handle to the network

Now, we derive some new signals from UI key press events and the clock signal:

% Create a signal from the key board presses.

keyPresses = net.fromUIEvent(figh, ‘WindowKeyPress Fcn’);

% Filter it, keeping only ‘ctrl’ key presses. Turn into logical signal.

reports = strcmp(keyPresses.Key, ‘ctrl’);

% Sample the current time at ‘presentationRatè.

sampler = skipRepeats(floor(presentationRate * t));

To change the orientation and phase at a given frequency, we derive some indexing signals that will select a value from the orientation and phase sets. The map method calls a function with the value of a signal each time it changes. @(∼) foo is the MATLAB syntax for creating an anonymous function. Each time the sampler signal changes, a new random integer is generated.

% Randomly sample orientations and phases by generating new indices for selecting values from ‘oris’ and ‘phases’ each time ‘sampler’ updates.

oriIdx = sampler.map(@(∼) randi(numel(oris))); % index for ‘oris’ array

phaseIdx = sampler.map(@(∼) randi(numel(phases))); % index for `phases` array

currPhase = phaseIdx.map(@(idx) phases(idx)); % get current phase

currOri = oriIdx.map(@(idx) oris(idx)); % get current ori

Next, we derive some signals for updating our plot of reaction times. First, a Boolean array the size of our orientation set is created, then we derive a matrix from these vectors, storing the last 10 orientations presented.

% Create a signal to indicate the current orientation (a Boolean column vector)

oriMask = oris’ == currOri;

% Record the last few orientations presented (i.e., ‘buffer’ the last few values that ‘oriMask’ has taken.) as a MxN matrix where M is the number of orientations (the length of ‘oris’) and N is the number of frames (‘winlen’)

oriHistory = oriMask.buffer(winlen);

Each time the user presses the “ctrl” key (represented by the reports signal), the values in the oriHistory matrix are added to the histogram via the scan method, which initializes the histogram with zeros.

% After each keypress, add the `oriHistory` snapshot to an accumulating histogram.

histogram = oriHistory.at(reports).scan(@plus, zeros(numel(oris), winlen));

Now, each time the histogram updates, we call imagesc with its value, updating the plot axes.

% Plot histogram surface each time it changes.

histogram.onValue(@(data) imagesc(oris, 1:winlen, flipud(data’), ‘Parent’, axh));

Finally, we create the visual stimulus signal and send it to the renderer. The vis.grating function returns a subscriptable signal, which has parameter fields related to visual grating properties. When the values of these signal fields are updated, the underlying textures are rerendered by setElemsFn.

% Create a Gabor with changing orientations and phases.

grating = vis.grating(t, ‘sinusoid’, ‘Gaussian’);

grating.show = true; % set grating to be always visible

grating.orientation = currOri; % assign orientation

grating.phase = currPhase; % assign phase

grating.spatialFreq = 0.2; % cyc/deg

% Add the grating to the renderer.

setElemsFn(struct(‘grating’, grating));

With this powerful framework, a user can easily define complex relationships between stimuli, actions, and outcomes to create a complete experiment protocol. This protocol takes the form of a user-written MATLAB function, which we refer to as an “experiment definition” (“exp def”).

**Figure 5.**
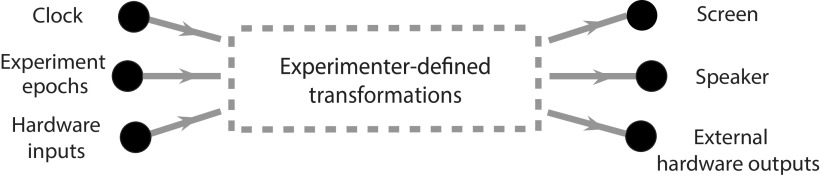
A Signals representation of an experiment. There are three types of input signals in the network, representing a clock, experiment epochs (e.g., new trials and experiment start and end conditions), and hardware input devices (e.g., an optical mouse, keyboard, rotary encoder, lever). In an exp def, the user defines transformations that create new signals (data not shown) from these input signals, which ultimately drive outputs (e.g., a screen, speaker, and external hardware such as a reward valve). The exp def is called once to create these experimenter-defined signals, which are updated during experiment runtime as the input signals they depend on are updated.

When Rigbox initializes an experiment, a new Signals network is created with input layer signals representing time, experiment epochs (e.g., new trials), and hardware input devices (e.g., position sensors). These input signals are passed into the exp def function, and the code in the exp def operates on these signals to create new signals that are added to the network (Fig. 5). The exp def is called just once to set up this network.

At experiment start, values are posted to the input signals of the network. During experiment runtime, these input signals are continuously updated within the main of the experiment while loop or through user interface UI and timer callbacks occur. For example, a position sensor input device may be read from continuously in a while loop to update the signal representing this device. These input signal updates asynchronously propagate to the dependent signals that were created in the exp def. The experiment ends when the “experiment stop” signal is updated (i.e., when all trial conditions have occurred or after a specified duration of time).

The following is a brief overview of the structure of an exp def. An exp def takes up to seven input arguments:

function expDef(t, events, params, visStim, inputs, outputs, audio)

In order, these are as follows: (1) the clock signal; (2) an events structure containing signals that define experiment epochs, and signals—from those created within the exp def—which the experimenter wishes to log; (3) a signal parameters structure that defines session- or trial-specific signals whose values can be changed directly within a graphical UI (GUI) before starting an experiment—signal parameter defaults are set within the exp def, and parameter sets can be saved and loaded across subjects and experiments; (4) the visual stimuli handler, which contains as fields all signals that parametrize the display of visual stimuli—any visual stimulus signal can be assigned various elements, which the viewing model allows to be defined in visual degrees, for being rendered to a screen, and a visual stimulus can be loaded directly from a saved image file; (5) an inputs structure containing signals that map to hardware input devices; (6) an outputs structure containing signals that map to external hardware output devices; and (7) the audio stimuli handler, which can contain as fields signals that map to available audio devices.

Tutorials on creating an exp def, examples of exp defs, and standalone scripts (including those mentioned in this article), and an in-depth overview of Signals can be found in the signals/docs folder https://github.com/cortex-lab/signals/tree/master/docs.

### Example 2: Pong

**Figure 6.**
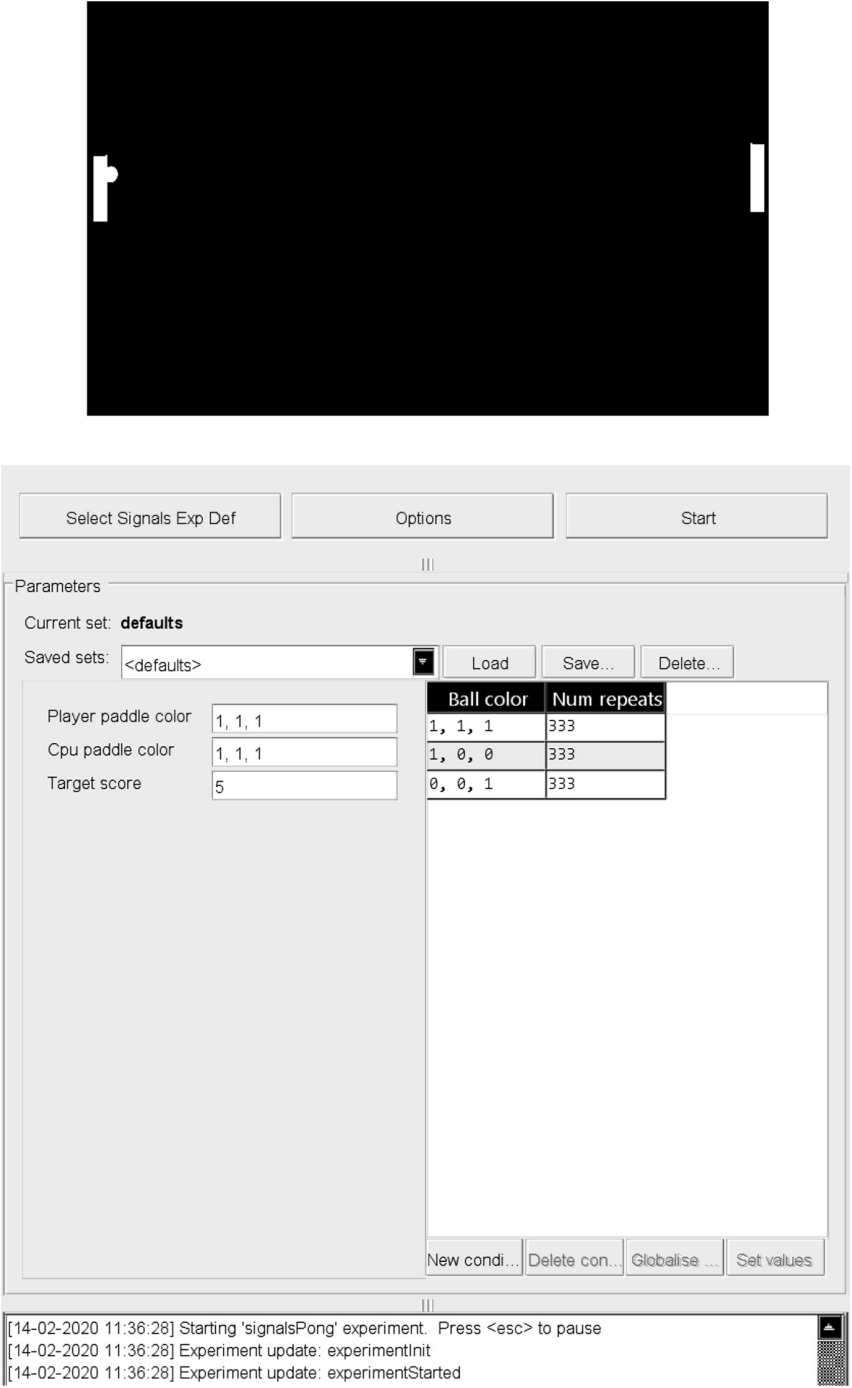
A screenshot of Pong run in Signals. The top shows the paddles and ball during gameplay. The bottom shows the GUI used to launch the game. The paddle colors (represented by an RGB vector) and target score are examples of global signal parameters that can be set once before starting the game. The ball color is an example of a conditional signal parameter that changes randomly after every trial (in this case, after a score) between the arrays indicated in each row (which in this case specify the colors white, red, and blue).

A second human-interactive Signals experiment contained in the Rigbox repository is an exp def that runs the classic computer game Pong (Fig. 6). The signal that sets the player’s paddle position is mapped to the optical mouse. The epoch structure is set so that a trial ends on a score, and the experiment ends when either the player or cpu (central processing unit) reaches a target score. The code is divided into the following three sections: (1) initializing the game; (2) updating the game; and (3) creating visual elements and defining exp def signal parameters. To run this exp def, follow the directions in the header of the signalsPong file https://github.com/cortex-lab/signals/blob/master/docs/examples/signalsPong.m. Because the file itself (including copious documentation) is >300 lines, we will share only an overview here; however, readers are encouraged to look through the full file at their leisure.function signalsPong(t, events, p, visStim, inputs, outputs, audio)

In this first section, we define constants for the game, arena, ball, and paddles:

%% Initialize the game

% how often to update the game in secs

[…]

% initial scores and target score

[…]

% size of arena, ball, and paddle: [w h] in visual degrees

[…]

% ball angle, and ball velocity in visual degrees per second

[…]

% cpu and player paddle X-axis positions in visual degrees

[…]

The helper function, getYPos, returns the y-position of the cursor, which will be used to set the player paddle:function yPos = getYPos()

[…]end

% get cursor’s initial y-positioncursorInitialY = events.expStart.map(@(∼) getYPos);

In the second section, we define how the ball and paddle interactions update the game:

%% Update game

% create a signal that will update the y-position of the player’s paddle using ‘getYPos

‘playerPaddleYUpdateVal = (cursor.map(@(∼)get YPos)-cursorInitialY)*cursorGain

% make sure the y-value of the player’s paddle is within the screen bounds,

playerPaddleBounds = cond(…

playerPaddleYUpdateVal > arenaSz(2)/2, arenaSz(2)/2, …

playerPaddleYUpdateVal < -arenaSz(2)/2, -arenaSz(2)/2, …

true, playerPaddleYUpdateVal);

% and only updates every `tUpdatè secs

playerPaddleY = playerPaddleBounds.at(tUpdate);

% Create a struct, `gameDataInit`, holding the initial game state

gameDataInit = struct;

…

% Create a subscriptable signal, `gameDatà, whose fields represent the current

% game state (total scores, etc.), and which will be updated every `tUpdatè secs

gameData = playerPaddleY.scan(@updateGame, gameDataInit).subscriptable;

The helper function, updateGame, updates gameData. Specifically, it updates the data structure with ball angle, velocity, position, cpu paddle position, and player and cpu scores, based on the current ball position, which is updated at each sampled timestep, as follows:function gameData = updateGame(gameData, playerPaddleY)

[…]end

% define trial end (when a score occurs)

anyScored = playerScore | cpuScore;

events.endTrial = anyScored.then(true);

% define game end (when player or cpu score reaches target score)

endGame = (playerScore == targetScore) | (cpuScore == targetScore);

events.expStop = endGame.then(true);

[…]

In the final section, we create the visual elements representing the arena, ball, and paddles, and define the exp def signal parameters, as follows:

%% Define the visual elements and the experiment signal parameters

% create the arena, ball, and paddles as ‘vis.patch’ subscriptable signals

arena = vis.patch(t, ‘rectangle’);

ball = vis.patch(t, ‘circle’);

ball.color = p.ballColor;

playerPaddle = vis.patch(t, ‘rectangle’);

cpuPaddle = vis.patch(t, ‘rectangle’);

% assign the arena, ball, and paddles to the ‘visStim’ subscriptable signal handlervis

Stim.arena = arena;

visStim.ball = ball;

visStim.playerPaddle = playerPaddle;

visStim.cpuPaddle = cpuPaddle;

% define parameters that will be displayed in the GUI

try

% ‘p.ballColor’ is a conditional signal parameter: on any given trial, the ball

% color will be chosen at random among three colors: white, red, blue

p.ballColor = [1 1 1; 1 0 0; 0 0 1]’; % RGB color vector array

% ‘p.targetScorè is a global signal parameter: it can be changed via the GUI used

% to run this exp def before starting the game

p.targetScore = 5;

catch

end

## Running experiments and managing data in Rigbox

Rigbox contains a suite of packages for interfacing with hardware, acquiring and managing data, communicating with a remote database, time aligning events from a variety of sources, and implementing a user interface for managing experiments.

Rigbox simplifies experiments by providing an abstract interface for hardware interactions. All hardware devices, including screens and speakers, are represented by abstract classes that provide a basic set of interface methods. Methods for initializing, configuring, and communicating with a particular device are handled by specific subclasses. This design choice avoids the creation of device-specific dependencies within the toolbox and the experiment code of the user. In this way, hardware devices can be swapped without modifying code or affecting the experiment workflow, and adding support for new devices is straightforward. For example, to support a new multifunction input/output (I/O) device (e.g., an Arduino or other microcontroller), one could simply extend the +hw/DaqController class, and to support a new hardware input sensor (e.g., a lever or joystick), one could simply subclass the +hw/PositionSensor class.

Intuitive and robust data management is another essential feature of Rigbox. Simple function wrappers save and locate data via human-readable experiment reference strings that reflect straightforward experiment directory structures (i.e., subject/date/session). Data can be saved both locally and remotely, and even distributed across multiple servers. Rigbox uses a single paths config file, making it simple to change the location of data and configuration files. Furthermore, this code can be easily integrated with the personal code of a user to generate read and write paths for arbitrary datasets. A Parameters class, which sets, validates, and assorts experiment conditions for each experiment, simplifies data analysis across experiments by standardizing parameterization. Rigbox can also communicate with an Alyx database to query and post data related to a subject or session. Alyx is a lightweight meta-database that can be hosted on an internal server or in the cloud (e.g., via Amazon Web Services). Alyx allows users to organize experiment sessions and their associated files, and to keep track of subject information, such as diet, breeding, and surgeries (Bonacchi et al., 2020).

**Figure 7.**
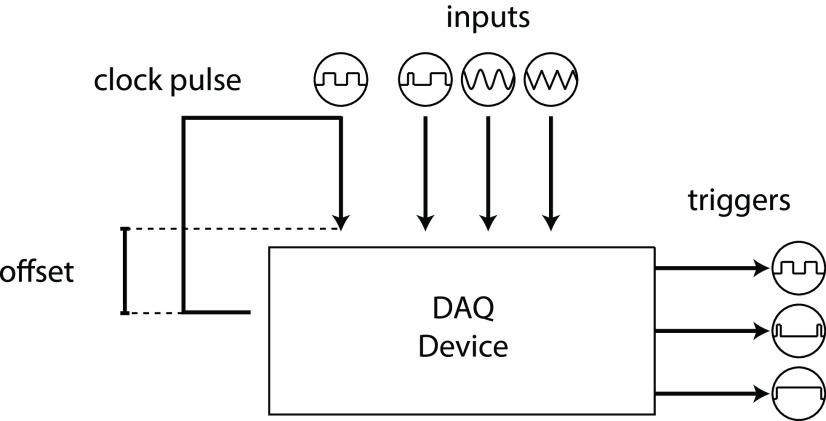
A representation of a Timeline object. The topmost signal is the main timing signal, “chrono,” which is used to unify all time stamps across computers during an experiment. The “inputs” represent different hardware and software input signals read by a NI-DAQ, and the “triggers” represent different hardware output signals, triggered by a NI-DAQ.

Experiments typically involve recording simultaneously from many devices, and temporal alignment of these recordings can be challenging. Rigbox contains a class called Timeline, which manages the acquisition and generation of clocking pulses via a National Instruments multifunction I/O data acquisition device (NI-DAQ; Fig. 7). The main clocking pulse of Timeline, “chrono,” is a digital square wave sent out from the NI-DAQ that can flip each time a new chunk of data and are available to the NI-DAQ. A callback function to this flip event collects the NI-DAQ time stamp of the scan where the flip occurred. The difference between this time stamp and the system time recorded when the flip command was sent is recorded as an offset time. This offset time can be used to unify all event time stamps across computers: all event time stamps are recorded in time relative to chrono. A Timeline object can acquire any number of hardware or software events [e.g., from hardware inputs directly wired to the NI-DAQ, or UDP (user datagram protocol) messages sent from another computer] and record their values with respect to this offset. For example, a Timeline object can record when a reward valve or laser shutter is opened, a sensor is interacted with, or a screen displaying visual stimuli is updated. In addition to chrono, a Timeline object can also output TTL (transistor–transistor logic) and clock pulses for triggering external devices (e.g., to acquire frames at a specific rate).

**Figure 8.**
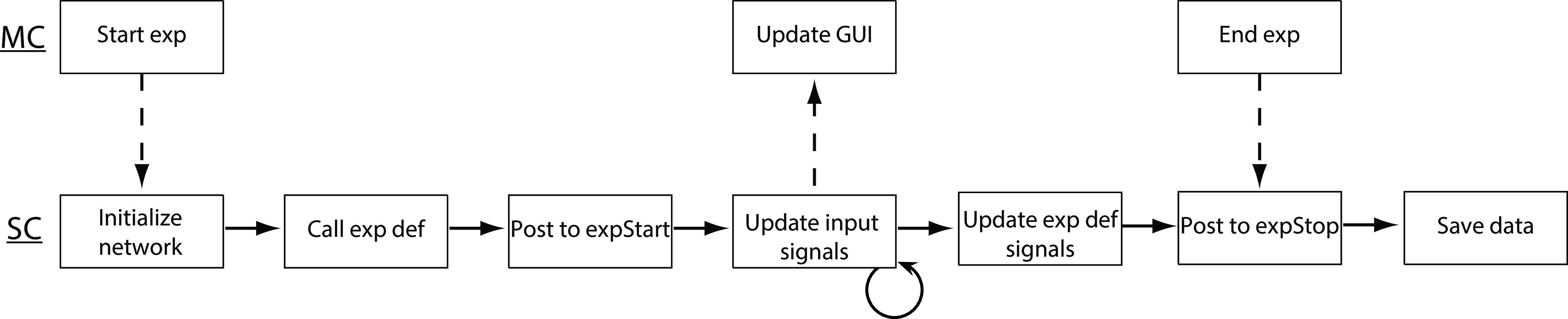
A simplified chronology of events that occur when starting an experiment via the MC GUI. Pushing the “Start” button on the MC GUI sends a message to SC to initialize a Signals network, then call the user’s Signals exp def to create new signals within the network, then post to the ‘expStart’ signal to start the experiment. After starting the experiment, the network input signals are continuously updated via callbacks (e.g., via a MATLAB timer callback, or by reading from hardware input devices), which update the rest of the signals in the network (i.e., those signals defined in the user’s exp def). These updates can then be displayed back to the user on the MC GUI. This continues until the experiment is either ended from the MC GUI, or a condition is met within the user’s exp def that updates the ‘expStop’ signal. After the experiment is ended, experiment data are saved.

**Figure 9.**
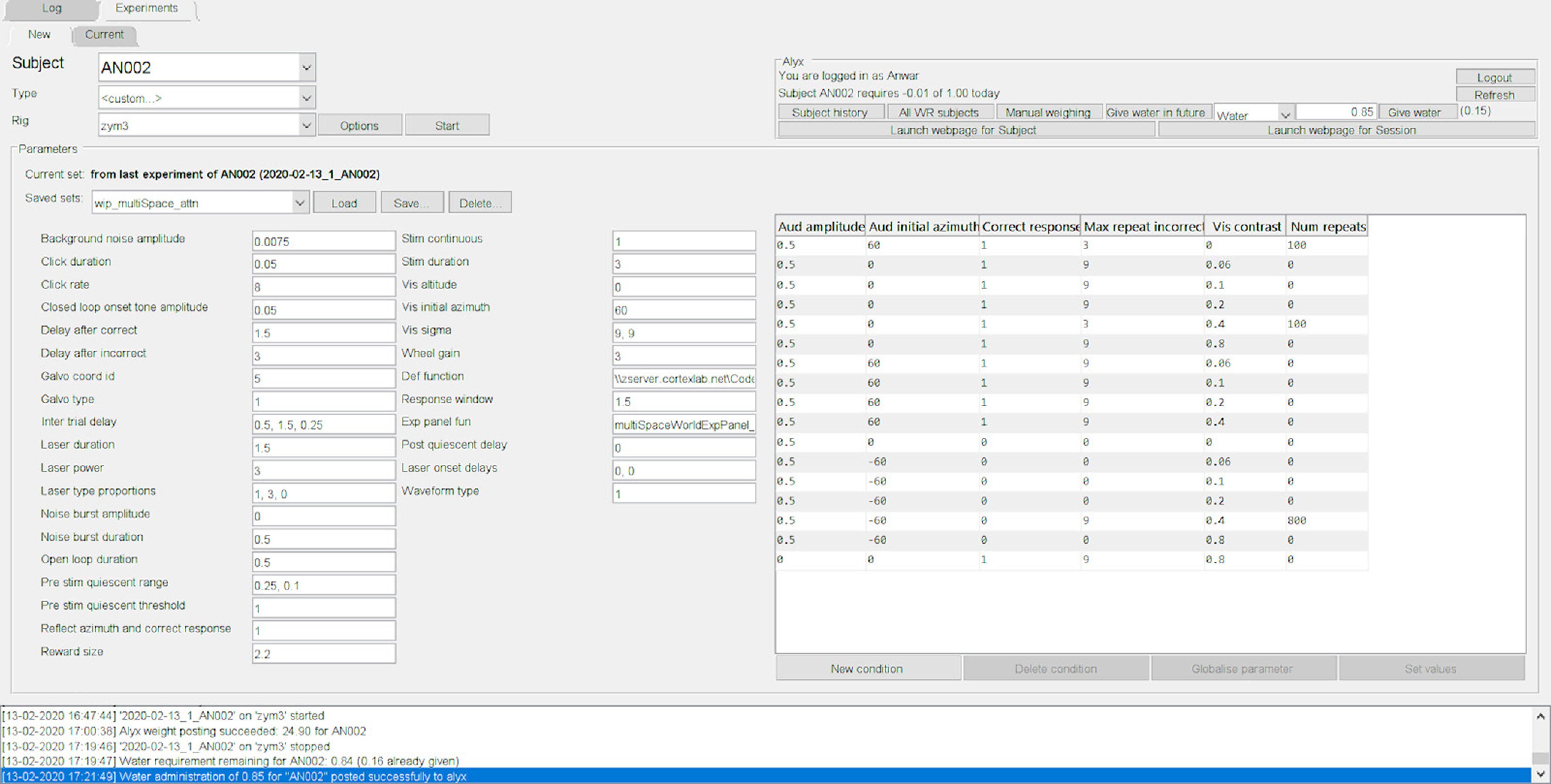
The new experiments tab within the MC GUI. This tab allows a user to select a subject, experiment type, and rig on which to run an experiment. Additionally, rig-specific options can be set via the “Options” button, and signal parameters for the behavioral task can be set via the editable parameter fields.

**Figure 10.**
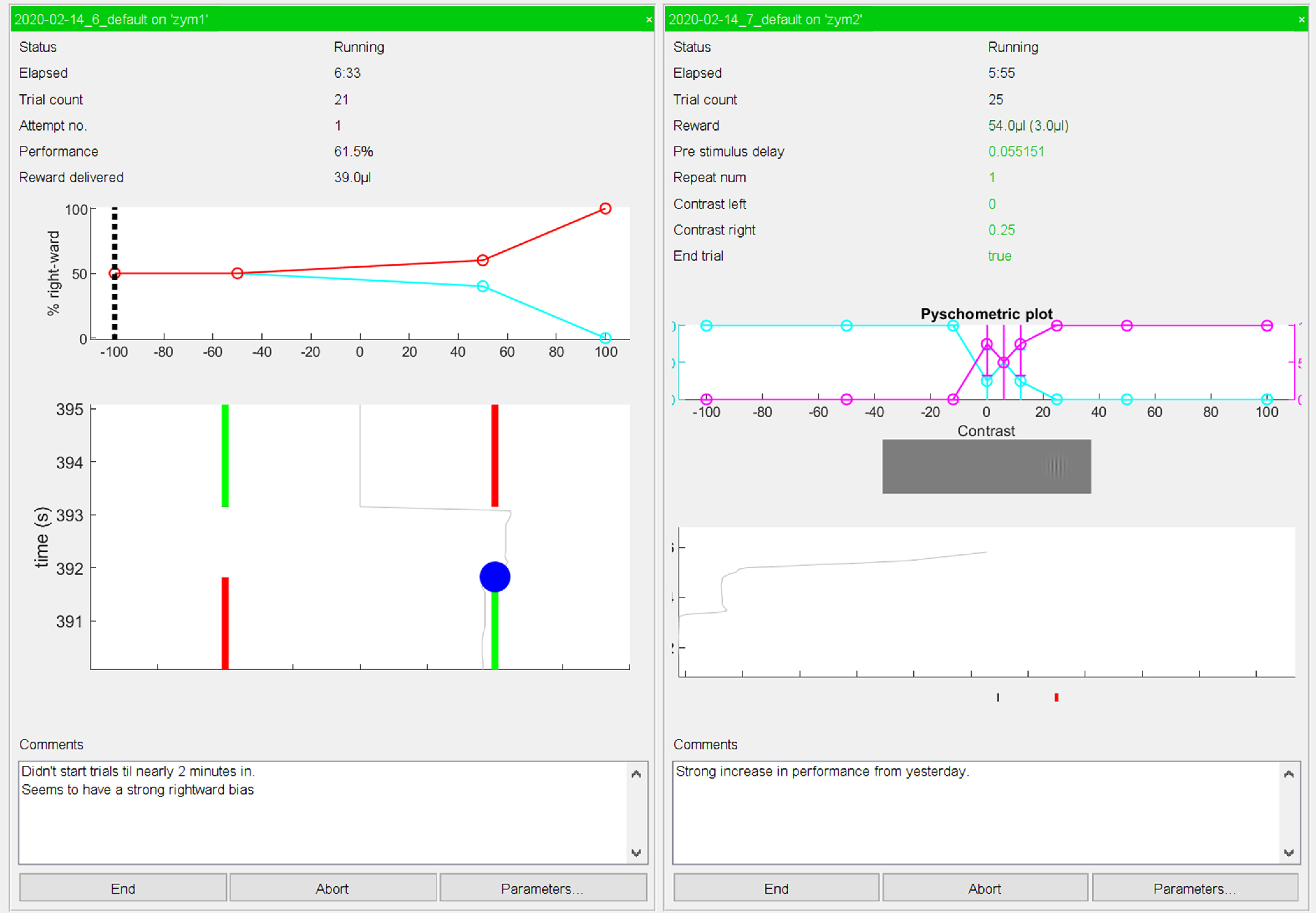
Experiment panels with live updates for two experiments. The top text fields in each panel display experiment information such as elapsed time, trial number, and the current running total of delivered reward. Below the text fields is a psychometric plot showing task performance for specific types of trials, and below this is a plot showing the real-time trace of a hardware input device (the panel on the left shows a two-alternative unforced choice task for which the green bar indicates the direction of the action the subject must make to receive a reward). There is also a text field for logging comments that can be immediately posted to an Alyx database. These experiment panels are highly customizable.

Last, Rigbox provides an intuitive yet powerful user interface for running experiments. For this, two computers are required. An experiment is started from a GUI on one computer, referred to as the “Master Computer” (MC), which runs the experiment on a recording rig, referred to as the “Stimulus Computer” (SC; Fig. 8). An SC is responsible for stimuli presentation, rig hardware interaction, and data acquisition. The MC GUI is used to select, parameterize, and start experiments (Fig. 9). Customizable experiment panels can also be displayed within a different tab in the MC GUI to monitor experiments (Fig. 10). MC and SC communicate during runtime via TCP/IP (transmission control protocol/internet protocol; using WebSockets), and MC can communicate with multiple SCs simultaneously to run multiple experiments in parallel.

Instructions for installation and configuration can be found in the README file and the docs/setup folder https://github.com/cortex-lab/Rigbox/tree/master/docs/setup. This includes information on required dependencies, setting data repository locations, configuring hardware devices, and enabling communication between the MC and SC computers. Hardware and software requirements can also be found in the repository README and the Requirements and benchmarking section of this article.

## Data availability

Rigbox is currently under active, test-driven development. All our code is open source, distributed under the Apache 2.0 license at https://github.com/cortex-lab/Rigbox (Extended Data 1), and we encourage users to contribute. Please see the contributing section of the README for information on contributing code and reporting issues. When using Rigbox to run behavioral tasks and/or acquire data, please cite this publication.

10.1523/ENEURO.0406-19.2020.ed1 Extended Data 1 We recommend looking at and downloading the code directly from the github repository, at https://github.com/cortex-lab/Rigbox. Download Extended Data 1, ZIP file.

## Discussion

In our laboratory, Rigbox is at the core of our operant, passive, and conditioning experiments. The principal behavioral task we use is a two-alternative forced choice visual stimulus discrimination task (Burgess et al., 2017). Using Rigbox, we have been able to rapidly prototype multiple variants of this task, including unforced choice, multisensory choice, behavior matching, and bandit tasks, using wheels, levers, balls, and lick detectors. The Signals exp defs for each variant act as a concise and intuitive record of the task design. In addition, Rigbox has made it easy to combine these tasks with a variety of recording techniques, including electrode recordings, 2-photon imaging, and fiber photometry; and neural perturbations, such as scanning laser inactivation and dopaminergic stimulation (Jun et al., 2017; Jacobs et al., 2018; Zatka-Haas et al., 2018; Shimaoka et al., 2019; Steinmetz et al., 2019; Armin et al., 2020). Rigbox has also enabled us to scale our behavioral training: because one MC can control multiple SCs, we run and manage many experiments simultaneously.

Often, experiments are iterative: task parameters are added or modified many times over, and finding an ideal parameter set can be an arduous process. Rigbox allows a user to develop and test an experiment without having to worry about boilerplate code and UI modifications, as these are handled by Rigbox packages in a modular fashion. Much of the code is object oriented with most aspects of the system represented as configurable objects. Given the modular nature of Rigbox, new features and hardware support may be easily added, provided there is driver support in MATLAB.

To the best of our knowledge, Rigbox is the most complete behavioral control software toolbox currently available in the neuroscience community; however, several other toolboxes implement similar features in different ways (Aronov and Tank, 2014; see also Bpod Wiki, https://sites.google.com/site/bpoddocumentation/home; BControl Behavioral Control System, https://brodywiki.princeton.edu/bcontrol/index.php?title=Main_Page; T. Akam, https://pycontrol.readthedocs.io/en/latest/; Table 1). Some of these toolboxes also include some features not currently available in Rigbox, for example, microsecond precision triggering of within-trial events, and creating 3D virtual environments. Indeed, the features used by a particular toolbox have advantages (and disadvantages) depending on the user’s desired experiment.

**Table 1:** Comparison of major features across behavioral control system toolboxes

	**BControl**	**pyBpod**	**pyControl**	**VirMEn**	**Rigbox**
Behavioral task design paradigm	Procedural	Procedural	Procedural	Object oriented	Functional reactive
Presents visual stimuli? 3D/VR environments?	No	No	No	Yes, yes	Yes, no
Interfaces with hardware?	Yes	Yes	Yes	Yes	Yes
Time aligns multiple datastreams?	Yes	Yes	Yes	No	Yes
Communicates with a remote database?	Yes	Yes	No	No	Yes
Contains unit and integration tests?	?	?	Yes	?	Yes

The top row contains the toolbox names, and the first column contains information on the implementation of a feature. Note: the toolboxes and features mentioned in this table are not exhaustive.

There are pros and cons to following different programming paradigms for software developers who decide how users will design behavioral tasks. Generally, three main paradigms exist: procedural, object oriented, and functional reactive. Here, in the context of programmatic task design, we briefly discuss the differences between these paradigms and in which scenarios one may be favored over the others. Note that here we discuss only the aspect of a toolbox that deals with behavioral task design, not the overall structure of a toolbox (e.g., Rigbox is built on an object-oriented paradigm, but Signals provides a functional reactive paradigm in which to implement a behavioral task).

A procedural approach to task design is probably the most familiar to behavioral neuroscientists. This approach focuses on “how to execute” a task by explicitly defining a control flow that moves a task from one state to the next. The Bcontrol, pyBpod, and pyControl toolboxes follow this paradigm by using real-time finite state machines (RTFSMs), which control the state of a task (e.g., initial state, reward, punishment) during each trial. Some advantages of this approach are that it is simple and intuitive, and guarantees event timing precision down to the minimum cycle of the state machine (e.g., Bcontrol RTFSMs run at a minimum cycle of 6 kHz). Some disadvantages of this approach are that the memory for task parameters are limited by the number of states in the RTFSM, and that the discrete implementation of states is not amenable to experiments that seek to control parameters continuously (e.g., a task that uses continuous hardware input signals).

Like the procedural approach to task design, an object-oriented approach also tends to be intuitive: objects can neatly represent the state of an experiment via datafields. Objects representing experimental parameters can easily pass information to each other and trigger experimental states via event callbacks. The VirMEn toolbox implements this approach by treating everything in the virtual environment as an object and having a runtime function update the environment by performing method calls on the objects based on input sensor signals from a subject performing a task. Some disadvantages of this approach are that the speed of experimental parameter updates are limited by the speed at which the programming language performs dynamic binding (which is often much slower than the RTFSM approach discussed above), and that operation “side effects” (which can alter an experiment’s state in unintended ways) are more likely to occur due to the emphasis on mutability, when compared with a pure procedural or functional reactive approach.

By contrast, Signals follows a functional reactive approach to task design. As we have seen, some advantages of this approach include simplifying the process of updating experiment parameters over time, endowing parameters with memory, and facilitating discrete and continuous event updates with equal ease. In general, a task specification in this paradigm is declarative, which can often make it clearer and more concise than in other paradigms, where control flow and event handling code can obscure the semantics of the task. Some disadvantages are that it suffers from similar speed limitations as in an object-oriented approach, and programmatically designing a task in a functional reactive paradigm is probably unfamiliar to most behavioral neuroscientists. When initially thinking about how a functional reactive network runs a behavioral task, it may be helpful to think of experiment parameters as nodes in the network that get updated via callbacks; there are no procedural calls to the network during experiment runtime.

When considering the entire set of behavioral tasks, no single programming paradigm is perfect, and it is therefore important for a user to consider the goals for the implementation of their task accordingly.

### Hardware requirements

For most experiments, typical, contemporary, factory-built desktops running Windows 10 with dedicated graphics cards should suffice. Specific requirements of an SC will depend on the complexity of the experiment. For example, running an audiovisual integration task on three screens requires quality graphics and sound cards. SCs may additionally require a multifunction I/O device to communicate with external rig hardware, of which only NI-DAQs (e.g., NI-DAQ USB 6211) are currently supported.

Below are some minimum hardware specifications required for computers that run Rigbox:

- CPU: 4 logical processors @ 3.0 GHz base speed (e.g., Intel Core i5-6500)
- RAM: DDR4 16 GB @ 2133 MHz (e.g., Corsair Vengeance 16 GB)
- GPU: 2 GB @ 1000 MHz base and memory speed (e.g., NVIDIA Quadro P400).

### Software requirements

Similar to the hardware requirements, software requirements for an SC will depend on the experiment. For example, if acquiring data through a NI-DAQ, the SC will require the MATLAB NI-DAQmx support package in addition to the following minimum requirements:

- OS: 64 Bit Windows 7 (or later)
- Libraries: Visual C++ Redistributable Packages for Visual Studio 2013 and 2015
- MATLAB: 2018b or later, including the Data Acquisition Toolbox

• Community MATLAB toolboxes:

∘ GUI Layout Toolbox (version 2 or later)

∘ Psychophysics Toolbox (version 3 or later).

### Benchmarking

**Figure 11.**
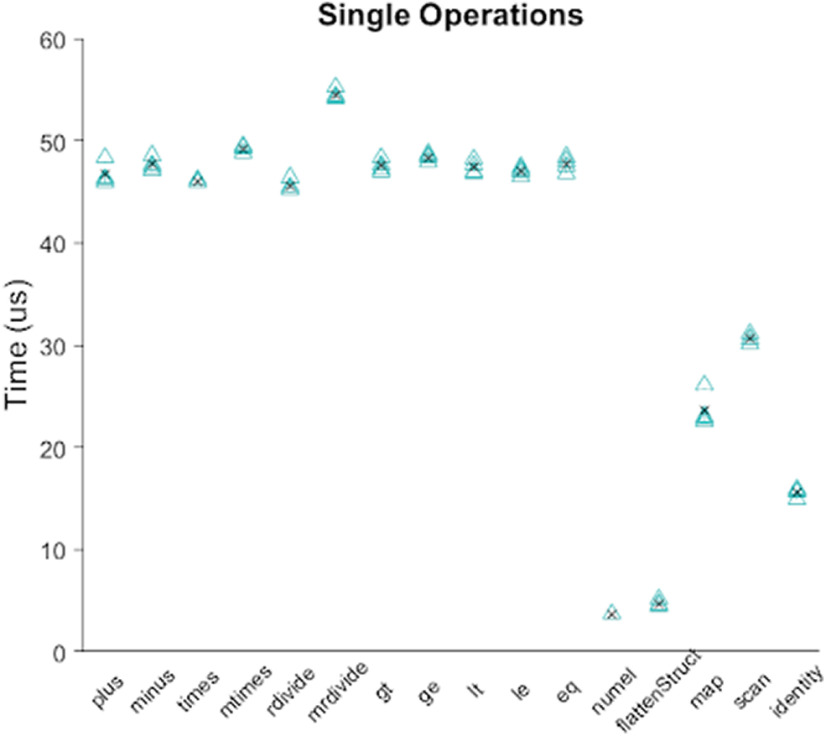
Benchmarking results for operations (specified by the *x*-axis) on a single signal. The black “x” shows the mean value per group.

**Figure 12.**
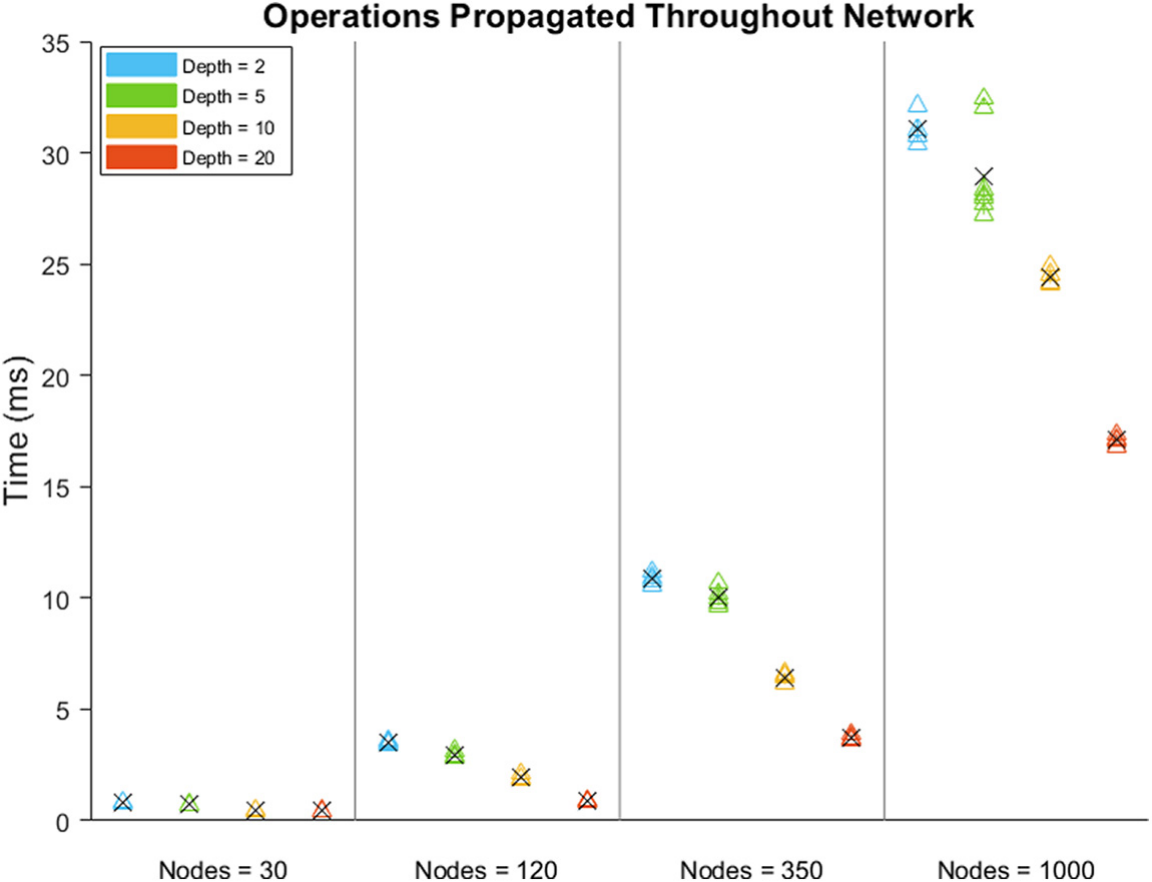
Benchmarking results for updating every signal in a network, for networks of various number of signals (nodes) spread over various number of layers (depth). The black “x” shows the mean value per group.

Fast execution of experiment runtime code is crucial for performing and accurately analyzing results from a behavioral experiment. Here we provide benchmarking results for the Signals framework. We include results for individual operations on a signal and for operations that propagate through each signal in a network. Single built-in MATLAB operations and Signals-specific methods are consistently executed in the microsecond range (Fig. 11). The network used in a typical two-alternative unforced stimulus discrimination task (https://github.com/cortex-lab/signals/blob/558170702aa2e6962c58f8b2a7b603a96b2c6b1a/docs/examples/advancedChoiceWorld.m) contains 338 signals spread over 10 layers; a similar network of 350 signals spread over 20 layers can update all signals in under 5 ms, and a network of 120 signals spread over 20 layers can update all signals with submillisecond precision (Fig. 12). Last, we include results for reading from and triggering hardware devices in the above-mentioned stimulus discrimination task.

**Figure 13.**
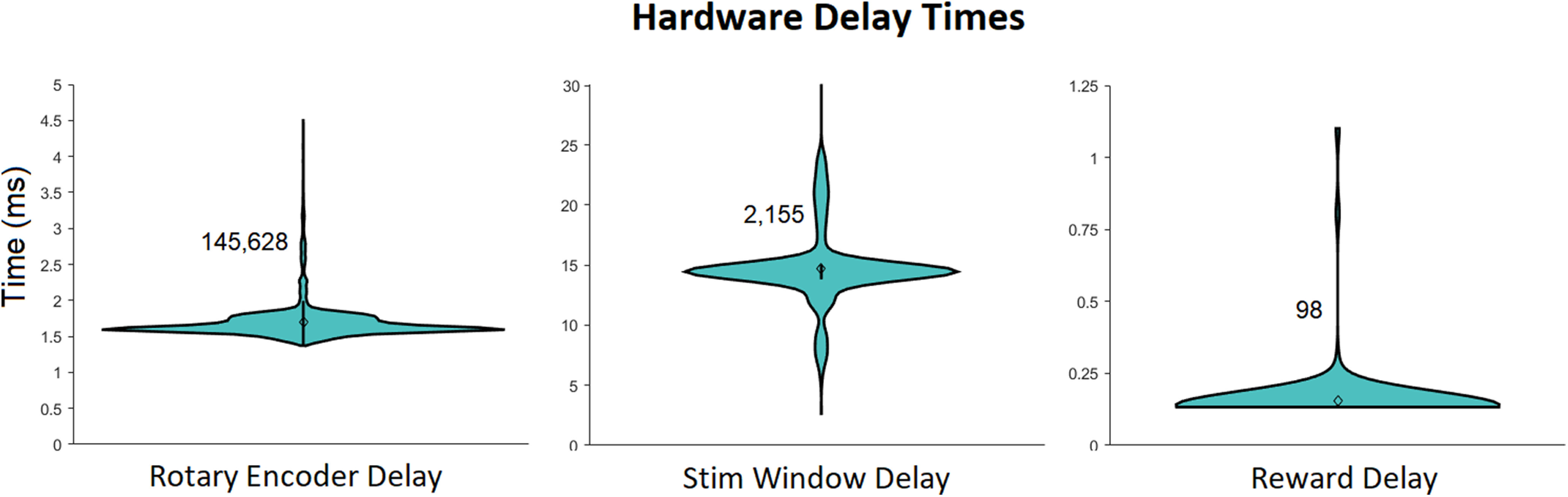
Delay times for specific updates when running a 2AFC (two-alternative forced choice) visual contrast discrimination task. The number next to each violin plot indicates the number of samples in the group. “Rotary Encoder delay” is the time between polling consecutive position values from a rotary encoder. “Stim Window Delay” is the time between triggering a display to be rendered, and its complete render on a screen. “Reward Delay” is the time between triggering and opening a reward valve. The 99th percentile outliers were not included in the plot for “Rotary Encoder delay”: there were 98 instances in which the delay took between 200 and 600 ms, due to the execution time of the NI-DAQmx MATLAB package when sending analog output (reward delivery) via the USB-6211 DAQ.

Updates of the position of a rotary encoder used to indicate choice typically took <2 ms, the time between rendering and displaying the visual stimulus typically took <15 ms, and the delay between triggering and delivering a reward was typically under 0.2 ms (Fig. 13).

All of the results in the Benchmarking section were obtained from running MATLAB 2018b on a Windows 10 64 bit OS with an Intel core i7 8700 processor and 16 GB DDR4 dual-channel RAM clocking at a double data rate of 2133 MHz. Because single executions of signals operations were too quick for MATLAB to measure precisely, we repeated operations 1000 times and divided the MATLAB returned measured time by 1000. The Performance Testing Framework in MATLAB 2018b was used to obtain these results. https://github.com/cortex-lab/signals/blob/558170702aa2e6962c58f8b2a7b603a96b2c6b1a/tests/Signals_perftest.m contains the code used to generate the results shown in Figures 11 and 12; https://github.com/cortex-lab/signals/tree/558170702aa2e6962c58f8b2a7b603a96b2c6b1a/tests/results contains a table of these data; and https://github.com/cortex-lab/signals/tree/558170702aa2e6962c58f8b2a7b603a96b2c6b1a/tests/results contains the data used to generate the results shown in Figure 13*A*. National Instruments USB-6211 was used as the data acquisition I/O device.

## Synthesis

Reviewing Editor: William Stacey, University of Michigan Decisions are customarily a result of the Reviewing Editor and the peer reviewers coming together and discussing their recommendations until a consensus is reached. When revisions are invited, a fact-based synthesis statement explaining their decision and outlining what is needed to prepare a revision will be listed below. The following reviewer(s) agreed to reveal their identity: Daniel Leventhal, Kyle Lillis.

Both of the reviewers found the software package to be interesting and potentially useful. Regarding the paper itself, there are some minor improvements that need to be addressed (A). The larger concern, however, is that neither reviewer was actually able to test the package. One of the reviewers actually spent several hours trying to get it to work and eventually gave up. In order to “announce” this package in eNeuro, it will be necessary to present it in such a way that readers can reasonably install it following the printed instructions (B). As this package has several hardware and software dependencies, it will also be necessary to detail exactly what the minimal requirements are.

A) Manuscript revisions

A1) What do the error bars represent in Figures 9 and 10?

A2) The steering wheel task is unfamiliar, which made it difficult to evaluate Figure 11. If this is going to be used for benchmarking, a brief explanation of the task would be helpful.

A3) Functional reactive programming (FRP) is likely to be pretty unfamiliar to most readers of eneuro. One of the reviewers is experienced in procedural, imperative, and object-oriented programming, but stated that it was not intuitive what this programming workflow was doing. Specifically: “when I begin writing software, I think about what is going to execute first and what happens when inputs are received or state changes occur. In reading through the Introduction and Methods section, I struggled to gain intuition for how that happens in Rigbox. Fig 2 is intended to convey some of this information, but lacks clarity. For example, it is not clear from looking at Fig 2 which packages are running on MC vc SC. And there are several boxes which do not seem to be central to understanding the overall architecture (e.g. srv, alyx-matlab, auxillary services).” It seems the figure had both too much and not enough information. It should be revised to provide a better top-down approach for how this unfamiliar programming style works.

A4) similar to the problems with A3, we would like a diagram showing a timeline of how a program runs from the time you click Start on the MC GUI. The expDef function seems to define the update conditions, input/output transformation, and data storage/analysis, but it is not called every timestep. How does this work?

A5) due to poor understanding from A3 and A4, the reviewers could not understand the logic behind the program itself. Much more description is needed. For example: Where did the sig object come from? what is playgroundPTB? One should be able to gain a basic level of understanding about what is happening by just reading the manuscript (without downloading all of the source code). There is also very little documentation in the playgroundPTB.m file itself. For other investigators to implement this program, they will require a clear understanding that allows them to adapt it to their own use.

A6) example 1 says to press control on horizontal grating, but the figure shows vertical.

B) Code revisions

B1) As this is the presentation of a method for free access, it is imperative that the program be accessible and reproducible. As is often the case when a platform is developed in-house, there are many dependencies that make it challenging for other investigators to implement the program. In this case, there are many such dependencies, and one reviewer spent several hours trying to run this program before giving up. This is their experience:

"Downloading and installing the platform to get the examples to run is not trivial. Checking out Rigbox from github is straightforward, but the steps that follow did not go smoothly for me. Running addRigboxPaths, gave me links to multiple other packages to install, which would be fine except that the link for Psychtoolbox was a git repository indicating that it was for developers only and redirecting me to the website: www.psychtoolbox.org. Following the instructions there is also fairly elaborate. Perhaps the ‘addRigboxPaths.m’ could be modified to call the DownloadPsychToolbox.m function (or a git-based version of it) directly? I was also instructed to manually copy a dll and to overwrite a dll in C:\Windows\System32, which is a little unnerving, though perhaps necessary (Editor note: this step alone would make many scientists avoid this program, as it appears to be a security risk). Finally, the hardware configuration steps are not trivial. Taken individually, none of these issues is insurmountable, but together they create a large barrier to trying out the software. It should be a more streamlined process for a reader to run the examples from the manuscript. There are likely legal issues with distributing all components in one installable package, but it would simplify things if you could have a list of non-redistributable prequisite packages (and links to them) that need to be installed first (e.g. MSVC runtime, gstreamer, git, svn). Then have the user run a script that downloads and configures Rigbox and psychtoolbox and configures hardware as MC/SC running on the the same computer, a basic pc with keyboard, mouse, and display, which should be sufficient to run the example code.”

Therefore, please redesign the presentation, manual, code, and interface so that installation is clear and reproducible. I suggest you contact collaborators uninvolved in this process (and not currently using the ancillary software like Psychtoolbox) and test whether they are able to successfully run the examples without any input from you, then determine/solve the problems they have.

B2). Both reviewers asked for clear description of the software and hardware requirements. This would help the choice of experimental hardware. What are the requirements for the SC and MC? Do both need Windows and Matlab licenses? For the sake of making this platform more accessible to labs with limited financial resources (and/or to reduce cost of scaling up the number of experimental rigs for all labs), would it be possible to have an SC that is based on something like a Raspberry Pi or Arduino? The manuscript mentions the possibility of using such devices as hw i/o peripherals, but could they actually act as the SC? Matlab has resources for compiling code specifically for these platform: https://www.mathworks.com/discovery/arduino-programming-matlab-simulink.html and https://www.mathworks.com/hardware-support/raspberry-pi-matlab.html

## Author Response

Synthesis Statement for Author (Required):

Both of the reviewers found the software package to be interesting and potentially useful. Regarding the paper itself, there are some minor improvements that need to be addressed (A). The larger concern, however, is that neither reviewer was actually able to test the package. One of the reviewers actually spent several hours trying to get it to work and eventually gave up. In order to “announce” this package in eNeuro, it will be necessary to present it in such a way that readers can reasonably install it following the printed instructions.

We thank the reviewers for this suggestion, which is of course crucial. We have streamlined the installation process by excluding extraneous details and making it much easier to get started with a GUI for running the paper’s example experiments. We have also asked two collaborators to give us feedback on their experience installing the toolbox.

(B). As this package has several hardware and software dependencies, it will also be necessary to detail exactly what the minimal requirements are.

Thanks for the suggestion, we have added a minimum hardware requirements section, and made clearer what the minimal software dependencies are in both the paper and the repository’s README.

A) Manuscript revisions

A1) What do the error bars represent in Figures 9 and 10?

Please note that Figures 9 and 10 are now Figures 11 and 12. We now realize that this wasn’t clear, but those were actually just the data points and the mean. We have made these plots clearer by indicating the data samples as small points and the mean as a black “x”.

A2) The steering wheel task is unfamiliar, which made it difficult to evaluate Figure 11. If this is going to be used for benchmarking, a brief explanation of the task would be helpful.

Please note that Figure 11 is now Figure 13. Thank you for bringing this to our attention. We have since removed any explicit mention of the steering wheel task, instead describing it as a 2-alternative choice task, which we hope is the right amount of information needed to get an idea of the hardware device delays observed in this type of experiment.

A3) Functional reactive programming (FRP) is likely to be pretty unfamiliar to most readers of eneuro. One of the reviewers is experienced in procedural, imperative, and object-oriented programming, but stated that it was not intuitive what this programming workflow was doing.

Thank you for this comment, we agree that this term is unfamiliar to most people, and without more context this is not intuitive enough. We have now reworded paragraphs in the “Signals” subsection and the “Discussion” section to mention how this programming paradigm is more rarely encountered than procedural paradigms, and to better introduce the concept first. We have added the following sentences:

Whenever the clock signal, “t", is updated, the values of all its dependent signals are then recalculated asynchronously via callbacks. This paradigm is known as functional reactive programming (Lew, 2017).

When initially thinking about how a functional reactive network runs a behavioral task, it may be helpful to think of experiment parameters as nodes in the network that get updated via callbacks; there are no procedural calls to the network during experiment runtime.

Specifically: “when I begin writing software, I think about what is going to execute first and what happens when inputs are received or state changes occur. In reading through the Introduction and Methods section, I struggled to gain intuition for how that happens in Rigbox.

This is an excellent point. Much of the Signals section has been rewritten to provide a clearer description of this process, starting with a simple, concrete example (a “clock” signal updated by a MATLAB timer callback). Additionally we now state that calling the exp def function (which ‘wires the network’) happens only once, and we remind the user of this throughout the paper. Below is one such paragraph:

During experiment runtime, these input signals are continuously updated within the experiment’s main while loop or through UI and timer callbacks, and the updates of these input signals asynchronously propagate updates to their dependent signals that have been created in the exp def

Further, we acknowledge in the discussion that this way of programming, although mathematically convenient, can be unintuitive to programmers used to writing procedural code. We are also constantly improving our user guides to make this less of a hurdle for new users.

Fig 2 is intended to convey some of this information, but lacks clarity. For example, it is not clear from looking at Fig 2 which packages are running on MC vc SC. And there are several boxes which do not seem to be central to understanding the overall architecture (e.g. srv, alyx-matlab, auxiliary services).” It seems the figure had both too much and not enough information. It should be revised to provide a better top-down approach for how this unfamiliar programming style works.

This figure has now been simplified, and moved to the supplementary section as figure S1. We thank you for this feedback, and can now see how this figure was a little overwhelming while also not conveying enough information to fit the original text it accompanied. This figure’s purpose is now to visually depict the modularity of Rigbox, depicting it as a group of packages that deal with separate parts of a behavior control system. Further, we have included a new figure that clarifies the roles of the two computers (see next point).

A4) similar to the problems with A3, we would like a diagram showing a timeline of how a program runs from the time you click Start on the MC GUI. The expDef function seems to define the update conditions, input/output transformation, and data storage/analysis, but it is not called every timestep. How does this work?

Thank you, this was a very valuable suggestion! A new figure (Figure 8) has been made that shows the chronology of events from the time of clicking Start on the MC GUI to an experiment’s end. This figure shows when and where an exp def function is called, and the caption explains how signals defined in an exp def are updated. This is now also mentioned earlier in the text in the “Signals” subsection. We believe these improvements clarify how and when signals are updated during an experiment.

A5) due to poor understanding from A3 and A4, the reviewers could not understand the logic behind the program itself. Much more description is needed. For example: Where did the sig object come from? What is playgroundPTB? One should be able to gain a basic level of understanding about what is happening by just reading the manuscript (without downloading all of the source code). There is also very little documentation in the playgroundPTB.m file itself. For other investigators to implement this program, they will require a clear understanding that allows them to adapt it to their own use.

We thank the reviews for this valuable feedback. Indeed, we have now provided much more description in the code examples, including where this mentioned sig object is created, and the mechanism by which it is updated. The `playgroundPTB` function is now described in much more detail in the paper and in the file’s documentation, enough to give readers a basic understanding of its purpose and what its outputs are.

A6) example 1 says to press control on horizontal grating, but the figure shows vertical.

Thank you for bringing this to our attention, this is now corrected.

B) Code revisions

B1) As this is the presentation of a method for free access, it is imperative that the program be accessible and reproducible. As is often the case when a platform is developed in-house, there are many dependencies that make it challenging for other investigators to implement the program. In this case, there are many such dependencies, and one reviewer spent several hours trying to run this program before giving up. This is their experience:

"Downloading and installing the platform to get the examples to run is not trivial. Checking out Rigbox from github is straightforward, but the steps that follow did not go smoothly for me.

We wholeheartedly agree with this point. We have updated the codebase with a goal in mind to reduce the dependencies, which we were able to do! Furthermore, the repository README now contains a “Getting Started” section that demonstrates how to run examples in a few lines of code. The installation steps are also much shorter, with extraneous configuration detail removed.

Running addRigboxPaths, gave me links to multiple other packages to install, which would be fine except that the link for Psychtoolbox was a git repository indicating that it was for developers only and redirecting me to the website: www.psychtoolbox.org. Following the instructions there is also fairly elaborate. Perhaps the ‘addRigboxPaths.m’ could be modified to call the DownloadPsychToolbox.m function (or a git-based version of it) directly?

Thanks for this feedback. Unfortunately we cannot improve PsychToolbox’s recommended instructions for installation. We considered distributing the DownloadPsychToolbox script, however after much deliberation have decided that this would be a challenge to maintain. Instead, we have provided our own simplified instructions for installing PsychToolbox in the Rigbox README, and clarified details in `addRigboxPaths`.

I was also instructed to manually copy a dll and to overwrite a dll in C:\Windows\System32, which is a little unnerving, though perhaps necessary (Editor note: this step alone would make many scientists avoid this program, as it appears to be a security risk).

Thanks for sharing this very valid concern. These required Microsoft VS C++ .dlls are now listed in the ‘requirements’ section of the README. Links are provided to the Microsoft Website where they can be downloaded and installed for free as part of Microsoft Redistributable Packages. The user is still provided the option of copying the file if they wish. We are looking into distributing installers with Rigbox in the future.

Finally, the hardware configuration steps are not trivial.

Yes, we agree that the hardware configuration can be quite involved. We’re constantly improving the hardware configuration steps, and the documentation. These steps are no longer necessary for running the examples in this paper. The install instructions no longer mention configuring hardware, these have been moved to the “running a full experiment” section.

Taken individually, none of these issues is insurmountable, but together they create a large barrier to trying out the software. It should be a more streamlined process for a reader to run the examples from the manuscript. There are likely legal issues with distributing all components in one installable package, but it would simplify things if you could have a list of non-redistributable prequisite packages (and links to them) that need to be installed first (e.g. MSVC runtime, gstreamer, git, svn). Then have the user run a script that downloads and configures Rigbox and psychtoolbox and configures hardware as MC/SC running on the the same computer, a basic pc with keyboard, mouse, and display, which should be sufficient to run the example code.”

Yes, as discussed in our previous responses, we have now reduced our toolbox requirements, provided links to installers for all dependencies, provided clear instructions for installing Psychtoolbox, and greatly reduced the steps required to run a test experiment.

Therefore, please redesign the presentation, manual, code, and interface so that installation is clear and reproducible. I suggest you contact collaborators uninvolved in this process (and not currently using the ancillary software like Psychtoolbox) and test whether they are able to successfully run the examples without any input from you, then determine/solve the problems they have.

This is an excellent suggestion. We have asked two collaborators to install the toolbox from scratch and provide us feedback. - MW

B2). Both reviewers asked for clear description of the software and hardware requirements. This would help the choice of experimental hardware. What are the requirements for the SC and MC? Do both need Windows and Matlab licenses?

Thanks, we agree this needed to be clarified. Two computers are recommended for running complete experiments and both have the same minimum software and hardware requirements. Depending on the behavioral task to be run, SC may require additional hardware, e.g. a data acquisition i/o device. This is now explicitly mentioned in the README and in the paper’s appendix. For testing Rigbox and running example experiments, only one computer is required. We have also provided detailed steps in our documentation explaining this.

For the sake of making this platform more accessible to labs with limited financial resources (and/or to reduce cost of scaling up the number of experimental rigs for all labs), would it be possible to have an SC that is based on something like a Raspberry Pi or Arduino?

The manuscript mentions the possibility of using such devices as hw i/o peripherals, but could they actually act as the SC? Matlab has resources for compiling code specifically for these platform: https://www.mathworks.com/discovery/arduino-programming-matlab-simulink.html and https://www.mathworks.com/hardware-support/raspberry-pi-matlab.html

We certainly agree with this point. The paper mentions that the modular nature of Rigbox makes integrating devices like an Arduino fairly simple. We discuss this in the paper in the new “Running Experiments and Managing Data in Rigbox” section. That said, currently Rigbox only works with NI devices, and hw i/o peripherals like microcontrollers cannot serve as SCs. We are investigating this issue and are prioritizing adding support for microcontrollers in the near future. We have reduced our MATLAB toolbox dependencies to aid in this cause.
