# Supplementary material for: Rigbox: An Open-Source Toolbox for Probing Neurons and Behavior
Source: eNeuro. 2020 Jul 9;7(4):ENEURO.0406-19.2020. doi: 10.1523/ENEURO.0406-19.2020 (PMC7363478; doi:10.1523/ENEURO.0406-19.2020)
Supplement: Extended Data 1 — We recommend looking at and downloading the code directly from the github repository, at https://github.com/cortex-lab/Rigbox. Download Extended Data 1, ZIP file. [file enu-eN-OTM-0406-19-s02.zip › Rigbox-master-2020-05-18/Rigbox-master/cb-tools/burgbox/html/num2cellstr.html]

num2cellstr 

```
function cstr = num2cellstr(A)
%NUM2CELLSTR Convert an array of numbers to a cell array of strings
%
% Part of Burgbox

% 2014-01 CB created

cstr = reshape(regexp(sprintf('%g#', A),'[0-9\-\.e]*', 'match'), size(A));
end
```

```
Error using num2cellstr (line 8)
Not enough input arguments.
```

```
for x = 1:10
    disp(x)
end
```

Published with MATLAB® R2013a
