# Supplementary material for: Rigbox: An Open-Source Toolbox for Probing Neurons and Behavior
Source: eNeuro. 2020 Jul 9;7(4):ENEURO.0406-19.2020. doi: 10.1523/ENEURO.0406-19.2020 (PMC7363478; doi:10.1523/ENEURO.0406-19.2020)
Supplement: Extended Data 1 — We recommend looking at and downloading the code directly from the github repository, at https://github.com/cortex-lab/Rigbox. Download Extended Data 1, ZIP file. [file enu-eN-OTM-0406-19-s02.zip › Rigbox-master-2020-05-18/Rigbox-master/cb-tools/java-source/autobahn reports/servers/index.html]

Toggle Details


  


Summary report generated on 2012-02-04T15:47:22Z (UTC) by Autobahn WebSockets v0.4.10.

|  |  |
| --- | --- |
| Pass | Test case was executed and passed successfully. |
| Non-Strict | Test case was executed and passed non-strictly. A non-strict behavior is one that does not adhere to a SHOULD-behavior as described in the protocol specification or a well-defined, canonical behavior that appears to be desirable but left open in the protocol specification. An implementation with non-strict behavior is still conformant to the protocol specification. |
| Fail | Test case was executed and failed. An implementation which fails a test case - other than a performance/limits related one - is non-conforming to a MUST-behavior as described in the protocol specification. |
| Info | Informational test case which detects certain implementation behavior left unspecified by the spec but nevertheless potentially interesting to implementors. |
| Missing | Test case is missing, either because it was skipped via the test suite configuration or deactivated, i.e. because the implementation does not implement the tested feature or breaks during running the test case. |

|  |  |  |
| --- | --- | --- |
| 1 Framing | tootallnate/websocket | |
| 1.1 Text Messages | | |
| Case 1.1.1 | Pass | None |
| Case 1.1.2 | Pass | None |
| Case 1.1.3 | Pass | None |
| Case 1.1.4 | Pass | None |
| Case 1.1.5 | Pass | None |
| Case 1.1.6 | Pass | None |
| Case 1.1.7 | Pass | None |
| Case 1.1.8 | Pass | None |
| 1 Framing | tootallnate/websocket | |
| 1.2 Binary Messages | | |
| Case 1.2.1 | Pass | None |
| Case 1.2.2 | Pass | None |
| Case 1.2.3 | Pass | None |
| Case 1.2.4 | Pass | None |
| Case 1.2.5 | Pass | None |
| Case 1.2.6 | Pass | None |
| Case 1.2.7 | Pass | None |
| Case 1.2.8 | Pass | None |
| 2 Pings/Pongs | tootallnate/websocket | |
| Case 2.1 | Pass | None |
| Case 2.2 | Pass | None |
| Case 2.3 | Pass | None |
| Case 2.4 | Pass | None |
| Case 2.5 | Pass | None |
| Case 2.6 | Pass | None |
| Case 2.7 | Pass | None |
| Case 2.8 | Pass | None |
| Case 2.9 | Pass | None |
| Case 2.10 | Pass | None |
| Case 2.11 | Pass | None |
| 3 Reserved Bits | tootallnate/websocket | |
| Case 3.1 | Pass | None |
| Case 3.2 | Non-Strict | None |
| Case 3.3 | Pass | None |
| Case 3.4 | Pass | None |
| Case 3.5 | Pass | None |
| Case 3.6 | Pass | None |
| Case 3.7 | Pass | None |
| 4 Opcodes | tootallnate/websocket | |
| 4.1 Non-control Opcodes | | |
| Case 4.1.1 | Pass | None |
| Case 4.1.2 | Pass | None |
| Case 4.1.3 | Non-Strict | None |
| Case 4.1.4 | Non-Strict | None |
| Case 4.1.5 | Pass | None |
| 4 Opcodes | tootallnate/websocket | |
| 4.2 Control Opcodes | | |
| Case 4.2.1 | Pass | None |
| Case 4.2.2 | Pass | None |
| Case 4.2.3 | Non-Strict | None |
| Case 4.2.4 | Non-Strict | None |
| Case 4.2.5 | Pass | None |
| 5 Fragmentation | tootallnate/websocket | |
| Case 5.1 | Pass | None |
| Case 5.2 | Pass | None |
| Case 5.3 | Pass | None |
| Case 5.4 | Pass | None |
| Case 5.5 | Pass | None |
| Case 5.6 | Pass | None |
| Case 5.7 | Pass | None |
| Case 5.8 | Pass | None |
| Case 5.9 | Pass | None |
| Case 5.10 | Pass | None |
| Case 5.11 | Pass | None |
| Case 5.12 | Pass | None |
| Case 5.13 | Pass | None |
| Case 5.14 | Pass | None |
| Case 5.15 | Pass | None |
| Case 5.16 | Pass | None |
| Case 5.17 | Pass | None |
| Case 5.18 | Pass | None |
| Case 5.19 | Pass | None |
| Case 5.20 | Pass | None |
| 6 UTF-8 Handling | tootallnate/websocket | |
| 6.1 Valid UTF-8 with zero payload fragments | | |
| Case 6.1.1 | Pass | None |
| Case 6.1.2 | Pass | None |
| Case 6.1.3 | Pass | None |
| 6 UTF-8 Handling | tootallnate/websocket | |
| 6.2 Valid UTF-8 unfragmented, fragmented on code-points and within code-points | | |
| Case 6.2.1 | Pass | None |
| Case 6.2.2 | Pass | None |
| Case 6.2.3 | Pass | None |
| Case 6.2.4 | Pass | None |
| 6 UTF-8 Handling | tootallnate/websocket | |
| 6.3 Invalid UTF-8 differently fragmented | | |
| Case 6.3.1 | Fail | Fail |
| Case 6.3.2 | Fail | Fail |
| 6 UTF-8 Handling | tootallnate/websocket | |
| 6.4 Fail-fast on invalid UTF-8 | | |
| Case 6.4.1 | Non-Strict | None |
| Case 6.4.2 | Non-Strict | None |
| Case 6.4.3 | Non-Strict | None |
| Case 6.4.4 | Non-Strict | None |
| 6 UTF-8 Handling | tootallnate/websocket | |
| 6.5 Some valid UTF-8 sequences | | |
| Case 6.5.1 | Pass | None |
| 6 UTF-8 Handling | tootallnate/websocket | |
| 6.6 All prefixes of a valid UTF-8 string that contains multi-byte code points | | |
| Case 6.6.1 | Pass | None |
| Case 6.6.2 | Pass | None |
| Case 6.6.3 | Pass | None |
| Case 6.6.4 | Pass | None |
| Case 6.6.5 | Pass | None |
| Case 6.6.6 | Pass | None |
| Case 6.6.7 | Pass | None |
| Case 6.6.8 | Pass | None |
| Case 6.6.9 | Pass | None |
| Case 6.6.10 | Pass | None |
| Case 6.6.11 | Pass | None |
| 6 UTF-8 Handling | tootallnate/websocket | |
| 6.7 First possible sequence of a certain length | | |
| Case 6.7.1 | Pass | None |
| Case 6.7.2 | Pass | None |
| Case 6.7.3 | Pass | None |
| Case 6.7.4 | Pass | None |
| 6 UTF-8 Handling | tootallnate/websocket | |
| 6.8 First possible sequence length 5/6 (invalid codepoints) | | |
| Case 6.8.1 | Pass | None |
| Case 6.8.2 | Pass | None |
| 6 UTF-8 Handling | tootallnate/websocket | |
| 6.9 Last possible sequence of a certain length | | |
| Case 6.9.1 | Pass | None |
| Case 6.9.2 | Pass | None |
| Case 6.9.3 | Pass | None |
| Case 6.9.4 | Pass | None |
| 6 UTF-8 Handling | tootallnate/websocket | |
| 6.10 Last possible sequence length 4/5/6 (invalid codepoints) | | |
| Case 6.10.1 | Pass | None |
| Case 6.10.2 | Pass | None |
| Case 6.10.3 | Pass | None |
| 6 UTF-8 Handling | tootallnate/websocket | |
| 6.11 Other boundary conditions | | |
| Case 6.11.1 | Pass | None |
| Case 6.11.2 | Pass | None |
| Case 6.11.3 | Pass | None |
| Case 6.11.4 | Pass | None |
| Case 6.11.5 | Pass | None |
| 6 UTF-8 Handling | tootallnate/websocket | |
| 6.12 Unexpected continuation bytes | | |
| Case 6.12.1 | Pass | None |
| Case 6.12.2 | Pass | None |
| Case 6.12.3 | Pass | None |
| Case 6.12.4 | Pass | None |
| Case 6.12.5 | Pass | None |
| Case 6.12.6 | Pass | None |
| Case 6.12.7 | Pass | None |
| Case 6.12.8 | Pass | None |
| 6 UTF-8 Handling | tootallnate/websocket | |
| 6.13 Lonely start characters | | |
| Case 6.13.1 | Pass | None |
| Case 6.13.2 | Pass | None |
| Case 6.13.3 | Pass | None |
| Case 6.13.4 | Pass | None |
| Case 6.13.5 | Pass | None |
| 6 UTF-8 Handling | tootallnate/websocket | |
| 6.14 Sequences with last continuation byte missing | | |
| Case 6.14.1 | Pass | None |
| Case 6.14.2 | Pass | None |
| Case 6.14.3 | Pass | None |
| Case 6.14.4 | Pass | None |
| Case 6.14.5 | Pass | None |
| Case 6.14.6 | Pass | None |
| Case 6.14.7 | Pass | None |
| Case 6.14.8 | Pass | None |
| Case 6.14.9 | Pass | None |
| Case 6.14.10 | Pass | None |
| 6 UTF-8 Handling | tootallnate/websocket | |
| 6.15 Concatenation of incomplete sequences | | |
| Case 6.15.1 | Pass | None |
| 6 UTF-8 Handling | tootallnate/websocket | |
| 6.16 Impossible bytes | | |
| Case 6.16.1 | Pass | None |
| Case 6.16.2 | Pass | None |
| Case 6.16.3 | Pass | None |
| 6 UTF-8 Handling | tootallnate/websocket | |
| 6.17 Examples of an overlong ASCII character | | |
| Case 6.17.1 | Pass | None |
| Case 6.17.2 | Pass | None |
| Case 6.17.3 | Pass | None |
| Case 6.17.4 | Pass | None |
| Case 6.17.5 | Pass | None |
| 6 UTF-8 Handling | tootallnate/websocket | |
| 6.18 Maximum overlong sequences | | |
| Case 6.18.1 | Pass | None |
| Case 6.18.2 | Pass | None |
| Case 6.18.3 | Pass | None |
| Case 6.18.4 | Pass | None |
| Case 6.18.5 | Pass | None |
| 6 UTF-8 Handling | tootallnate/websocket | |
| 6.19 Overlong representation of the NUL character | | |
| Case 6.19.1 | Pass | None |
| Case 6.19.2 | Pass | None |
| Case 6.19.3 | Pass | None |
| Case 6.19.4 | Pass | None |
| Case 6.19.5 | Pass | None |
| 6 UTF-8 Handling | tootallnate/websocket | |
| 6.20 Single UTF-16 surrogates | | |
| Case 6.20.1 | Fail | Fail |
| Case 6.20.2 | Fail | Fail |
| Case 6.20.3 | Fail | Fail |
| Case 6.20.4 | Fail | Fail |
| Case 6.20.5 | Fail | Fail |
| Case 6.20.6 | Fail | Fail |
| Case 6.20.7 | Fail | Fail |
| 6 UTF-8 Handling | tootallnate/websocket | |
| 6.21 Paired UTF-16 surrogates | | |
| Case 6.21.1 | Fail | Fail |
| Case 6.21.2 | Fail | Fail |
| Case 6.21.3 | Fail | Fail |
| Case 6.21.4 | Fail | Fail |
| Case 6.21.5 | Fail | Fail |
| Case 6.21.6 | Fail | Fail |
| Case 6.21.7 | Fail | Fail |
| Case 6.21.8 | Fail | Fail |
| 6 UTF-8 Handling | tootallnate/websocket | |
| 6.22 Non-character code points (valid UTF-8) | | |
| Case 6.22.1 | Pass | None |
| Case 6.22.2 | Pass | None |
| Case 6.22.3 | Pass | None |
| Case 6.22.4 | Pass | None |
| Case 6.22.5 | Pass | None |
| Case 6.22.6 | Pass | None |
| Case 6.22.7 | Pass | None |
| Case 6.22.8 | Pass | None |
| Case 6.22.9 | Pass | None |
| Case 6.22.10 | Pass | None |
| Case 6.22.11 | Pass | None |
| Case 6.22.12 | Pass | None |
| Case 6.22.13 | Pass | None |
| Case 6.22.14 | Pass | None |
| Case 6.22.15 | Pass | None |
| Case 6.22.16 | Pass | None |
| Case 6.22.17 | Pass | None |
| Case 6.22.18 | Pass | None |
| Case 6.22.19 | Pass | None |
| Case 6.22.20 | Pass | None |
| Case 6.22.21 | Pass | None |
| Case 6.22.22 | Pass | None |
| Case 6.22.23 | Pass | None |
| Case 6.22.24 | Pass | None |
| Case 6.22.25 | Pass | None |
| Case 6.22.26 | Pass | None |
| Case 6.22.27 | Pass | None |
| Case 6.22.28 | Pass | None |
| Case 6.22.29 | Pass | None |
| Case 6.22.30 | Pass | None |
| Case 6.22.31 | Pass | None |
| Case 6.22.32 | Pass | None |
| Case 6.22.33 | Pass | None |
| Case 6.22.34 | Pass | None |
| 6 UTF-8 Handling | tootallnate/websocket | |
| 6.23 Unicode replacement character | | |
| Case 6.23.1 | Pass | None |
| 7 Close Handling | tootallnate/websocket | |
| 7.1 Basic close behavior (fuzzer initiated) | | |
| Case 7.1.1 | Pass | None |
| Case 7.1.2 | Pass | None |
| Case 7.1.3 | Pass | None |
| Case 7.1.4 | Pass | None |
| Case 7.1.5 | Pass | None |
| Case 7.1.6 | Info | None |
| 7 Close Handling | tootallnate/websocket | |
| 7.3 Close frame structure: payload length (fuzzer initiated) | | |
| Case 7.3.1 | Pass | None |
| Case 7.3.2 | Pass | None |
| Case 7.3.3 | Pass | None |
| Case 7.3.4 | Pass | None |
| Case 7.3.5 | Pass | None |
| Case 7.3.6 | Pass | None |
| 7 Close Handling | tootallnate/websocket | |
| 7.5 Close frame structure: payload value (fuzzer initiated) | | |
| Case 7.5.1 | Pass | None |
| 7 Close Handling | tootallnate/websocket | |
| 7.7 Close frame structure: valid close codes (fuzzer initiated) | | |
| Case 7.7.1 | Pass | None |
| Case 7.7.2 | Pass | None |
| Case 7.7.3 | Pass | None |
| Case 7.7.4 | Pass | None |
| Case 7.7.5 | Pass | None |
| Case 7.7.6 | Pass | None |
| Case 7.7.7 | Pass | None |
| Case 7.7.8 | Pass | None |
| Case 7.7.9 | Pass | None |
| Case 7.7.10 | Pass | None |
| Case 7.7.11 | Pass | None |
| Case 7.7.12 | Pass | None |
| Case 7.7.13 | Pass | None |
| 7 Close Handling | tootallnate/websocket | |
| 7.9 Close frame structure: invalid close codes (fuzzer initiated) | | |
| Case 7.9.1 | Pass | None |
| Case 7.9.2 | Pass | None |
| Case 7.9.3 | Pass | None |
| Case 7.9.4 | Pass | None |
| Case 7.9.5 | Pass | None |
| Case 7.9.6 | Pass | None |
| Case 7.9.7 | Pass | None |
| Case 7.9.8 | Pass | None |
| Case 7.9.9 | Pass | None |
| Case 7.9.10 | Pass | None |
| Case 7.9.11 | Pass | None |
| Case 7.9.12 | Pass | None |
| Case 7.9.13 | Pass | None |
| 7 Close Handling | tootallnate/websocket | |
| 7.13 Informational close information (fuzzer initiated) | | |
| Case 7.13.1 | Info | None |
| Case 7.13.2 | Info | None |
| 9 Limits/Performance | tootallnate/websocket | |
| 9.1 Text Message (increasing size) | | |
| Case 9.1.1 | Pass 67 ms | None |
| Case 9.1.2 | Pass 260 ms | None |
| Case 9.1.3 | Pass 1042 ms | None |
| Case 9.1.4 | Pass 4180 ms | None |
| Case 9.1.5 | Pass 8364 ms | None |
| Case 9.1.6 | Pass 16993 ms | None |
| 9 Limits/Performance | tootallnate/websocket | |
| 9.2 Binary Message (increasing size) | | |
| Case 9.2.1 | Pass 39 ms | None |
| Case 9.2.2 | Pass 85 ms | None |
| Case 9.2.3 | Pass 338 ms | None |
| Case 9.2.4 | Pass 1375 ms | None |
| Case 9.2.5 | Pass 2740 ms | None |
| Case 9.2.6 | Pass 5554 ms | None |
| 9 Limits/Performance | tootallnate/websocket | |
| 9.3 Fragmented Text Message (fixed size, increasing fragment size) | | |
| Case 9.3.1 | Pass 70578 ms | None |
| Case 9.3.2 | Pass 20991 ms | None |
| Case 9.3.3 | Pass 8471 ms | None |
| Case 9.3.4 | Pass 5286 ms | None |
| Case 9.3.5 | Pass 4403 ms | None |
| Case 9.3.6 | Pass 4160 ms | None |
| Case 9.3.7 | Pass 4123 ms | None |
| Case 9.3.8 | Pass 4096 ms | None |
| Case 9.3.9 | Pass 4074 ms | None |
| 9 Limits/Performance | tootallnate/websocket | |
| 9.4 Fragmented Binary Message (fixed size, increasing fragment size) | | |
| Case 9.4.1 | Pass 69384 ms | None |
| Case 9.4.2 | Pass 17962 ms | None |
| Case 9.4.3 | Pass 5637 ms | None |
| Case 9.4.4 | Pass 2497 ms | None |
| Case 9.4.5 | Pass 1597 ms | None |
| Case 9.4.6 | Pass 1359 ms | None |
| Case 9.4.7 | Pass 1299 ms | None |
| Case 9.4.8 | Pass 1296 ms | None |
| Case 9.4.9 | Pass 1273 ms | None |
| 9 Limits/Performance | tootallnate/websocket | |
| 9.5 Text Message (fixed size, increasing chop size) | | |
| Case 9.5.1 | Pass 17529 ms | None |
| Case 9.5.2 | Pass 9293 ms | None |
| Case 9.5.3 | Pass 5166 ms | None |
| Case 9.5.4 | Pass 3168 ms | None |
| Case 9.5.5 | Pass 2137 ms | None |
| Case 9.5.6 | Pass 1571 ms | None |
| 9 Limits/Performance | tootallnate/websocket | |
| 9.6 Binary Text Message (fixed size, increasing chop size) | | |
| Case 9.6.1 | Pass 16825 ms | None |
| Case 9.6.2 | Pass 8639 ms | None |
| Case 9.6.3 | Pass 4504 ms | None |
| Case 9.6.4 | Pass 2434 ms | None |
| Case 9.6.5 | Pass 1461 ms | None |
| Case 9.6.6 | Pass 909 ms | None |
| 9 Limits/Performance | tootallnate/websocket | |
| 9.7 Text Message Roundtrip Time (fixed number, increasing size) | | |
| Case 9.7.1 | Pass 267 ms | None |
| Case 9.7.2 | Pass 289 ms | None |
| Case 9.7.3 | Pass 321 ms | None |
| Case 9.7.4 | Pass 551 ms | None |
| Case 9.7.5 | Pass 1435 ms | None |
| Case 9.7.6 | Pass 4887 ms | None |
| 9 Limits/Performance | tootallnate/websocket | |
| 9.8 Binary Message Roundtrip Time (fixed number, increasing size) | | |
| Case 9.8.1 | Pass 227 ms | None |
| Case 9.8.2 | Pass 259 ms | None |
| Case 9.8.3 | Pass 255 ms | None |
| Case 9.8.4 | Pass 355 ms | None |
| Case 9.8.5 | Pass 636 ms | None |
| Case 9.8.6 | Pass 1940 ms | None |
| 10 Autobahn Protocol Options | tootallnate/websocket | |
| 10.1 Auto-Fragmentation | | |
| Case 10.1.1 | Pass | None |

  

---

## Case 1.1.1

Up

**Case Description**  
  
Send text message with payload 0.

**Case Expectation**  
  
Receive echo'ed text message (with empty payload). Clean close with normal code.

  


## Case 1.1.2

Up

**Case Description**  
  
Send text message message with payload of length 125.

**Case Expectation**  
  
Receive echo'ed text message (with payload as sent). Clean close with normal code.

  


## Case 1.1.3

Up

**Case Description**  
  
Send text message message with payload of length 126.

**Case Expectation**  
  
Receive echo'ed text message (with payload as sent). Clean close with normal code.

  


## Case 1.1.4

Up

**Case Description**  
  
Send text message message with payload of length 127.

**Case Expectation**  
  
Receive echo'ed text message (with payload as sent). Clean close with normal code.

  


## Case 1.1.5

Up

**Case Description**  
  
Send text message message with payload of length 128.

**Case Expectation**  
  
Receive echo'ed text message (with payload as sent). Clean close with normal code.

  


## Case 1.1.6

Up

**Case Description**  
  
Send text message message with payload of length 65535.

**Case Expectation**  
  
Receive echo'ed text message (with payload as sent). Clean close with normal code.

  


## Case 1.1.7

Up

**Case Description**  
  
Send text message message with payload of length 65536.

**Case Expectation**  
  
Receive echo'ed text message (with payload as sent). Clean close with normal code.

  


## Case 1.1.8

Up

**Case Description**  
  
Send text message message with payload of length 65536. Sent out data in chops of 997 octets.

**Case Expectation**  
  
Receive echo'ed text message (with payload as sent). Clean close with normal code.

  


## Case 1.2.1

Up

**Case Description**  
  
Send binary message with payload 0.

**Case Expectation**  
  
Receive echo'ed binary message (with empty payload). Clean close with normal code.

  


## Case 1.2.2

Up

**Case Description**  
  
Send binary message message with payload of length 125.

**Case Expectation**  
  
Receive echo'ed binary message (with payload as sent). Clean close with normal code.

  


## Case 1.2.3

Up

**Case Description**  
  
Send binary message message with payload of length 126.

**Case Expectation**  
  
Receive echo'ed binary message (with payload as sent). Clean close with normal code.

  


## Case 1.2.4

Up

**Case Description**  
  
Send binary message message with payload of length 127.

**Case Expectation**  
  
Receive echo'ed binary message (with payload as sent). Clean close with normal code.

  


## Case 1.2.5

Up

**Case Description**  
  
Send binary message message with payload of length 128.

**Case Expectation**  
  
Receive echo'ed binary message (with payload as sent). Clean close with normal code.

  


## Case 1.2.6

Up

**Case Description**  
  
Send binary message message with payload of length 65535.

**Case Expectation**  
  
Receive echo'ed binary message (with payload as sent). Clean close with normal code.

  


## Case 1.2.7

Up

**Case Description**  
  
Send binary message message with payload of length 65536.

**Case Expectation**  
  
Receive echo'ed binary message (with payload as sent). Clean close with normal code.

  


## Case 1.2.8

Up

**Case Description**  
  
Send binary message message with payload of length 65536. Sent out data in chops of 997 octets.

**Case Expectation**  
  
Receive echo'ed binary message (with payload as sent). Clean close with normal code.

  


## Case 2.1

Up

**Case Description**  
  
Send ping without payload.

**Case Expectation**  
  
Pong (with empty payload) is sent in reply to Ping. Clean close with normal code.

  


## Case 2.2

Up

**Case Description**  
  
Send ping with small text payload.

**Case Expectation**  
  
Pong with payload echo'ed is sent in reply to Ping. Clean close with normal code.

  


## Case 2.3

Up

**Case Description**  
  
Send ping with small binary (non UTF-8) payload.

**Case Expectation**  
  
Pong with payload echo'ed is sent in reply to Ping. Clean close with normal code.

  


## Case 2.4

Up

**Case Description**  
  
Send ping with binary payload of 125 octets.

**Case Expectation**  
  
Pong with payload echo'ed is sent in reply to Ping. Clean close with normal code.

  


## Case 2.5

Up

**Case Description**  
  
Send ping with binary payload of 126 octets.

**Case Expectation**  
  
Connection is failed immediately (1002/Protocol Error), since control frames are only allowed to have payload up to and including 125 octets..

  


## Case 2.6

Up

**Case Description**  
  
Send ping with binary payload of 125 octets, send in octet-wise chops.

**Case Expectation**  
  
Pong with payload echo'ed is sent in reply to Ping. Implementations must be TCP clean. Clean close with normal code.

  


## Case 2.7

Up

**Case Description**  
  
Send unsolicited pong without payload. Verify nothing is received. Clean close with normal code.

**Case Expectation**  
  
Nothing.

  


## Case 2.8

Up

**Case Description**  
  
Send unsolicited pong with payload. Verify nothing is received. Clean close with normal code.

**Case Expectation**  
  
Nothing.

  


## Case 2.9

Up

**Case Description**  
  
Send unsolicited pong with payload. Send ping with payload. Verify pong for ping is received.

**Case Expectation**  
  
Nothing in reply to own Pong, but Pong with payload echo'ed in reply to Ping. Clean close with normal code.

  


## Case 2.10

Up

**Case Description**  
  
Send 10 Pings with payload.

**Case Expectation**  
  
Pongs for our Pings with all the payloads. Note: This is not required by the Spec .. but we check for this behaviour anyway. Clean close with normal code.

  


## Case 2.11

Up

**Case Description**  
  
Send 10 Pings with payload. Send out octets in octet-wise chops.

**Case Expectation**  
  
Pongs for our Pings with all the payloads. Note: This is not required by the Spec .. but we check for this behaviour anyway. Clean close with normal code.

  


## Case 3.1

Up

**Case Description**  
  
Send small text message with **RSV = 1**.

**Case Expectation**  
  
The connection is failed immediately (1002/protocol error), since RSV must be 0, when no extension defining RSV meaning has been negoiated.

  


## Case 3.2

Up

**Case Description**  
  
Send small text message, then send again with **RSV = 2**, then send Ping.

**Case Expectation**  
  
Echo for first message is received, but then connection is failed immediately, since RSV must be 0, when no extension defining RSV meaning has been negoiated. The Pong is not received.

  


## Case 3.3

Up

**Case Description**  
  
Send small text message, then send again with **RSV = 3**, then send Ping. Octets are sent in frame-wise chops. Octets are sent in octet-wise chops.

**Case Expectation**  
  
Echo for first message is received, but then connection is failed immediately, since RSV must be 0, when no extension defining RSV meaning has been negoiated. The Pong is not received.

  


## Case 3.4

Up

**Case Description**  
  
Send small text message, then send again with **RSV = 4**, then send Ping. Octets are sent in octet-wise chops.

**Case Expectation**  
  
Echo for first message is received, but then connection is failed immediately, since RSV must be 0, when no extension defining RSV meaning has been negoiated. The Pong is not received.

  


## Case 3.5

Up

**Case Description**  
  
Send small binary message with **RSV = 5**.

**Case Expectation**  
  
The connection is failed immediately, since RSV must be 0.

  


## Case 3.6

Up

**Case Description**  
  
Send Ping with **RSV = 6**.

**Case Expectation**  
  
The connection is failed immediately, since RSV must be 0.

  


## Case 3.7

Up

**Case Description**  
  
Send Close with **RSV = 7**.

**Case Expectation**  
  
The connection is failed immediately, since RSV must be 0.

  


## Case 4.1.1

Up

**Case Description**  
  
Send frame with reserved non-control **Opcode = 3**.

**Case Expectation**  
  
The connection is failed immediately.

  


## Case 4.1.2

Up

**Case Description**  
  
Send frame with reserved non-control **Opcode = 4** and non-empty payload.

**Case Expectation**  
  
The connection is failed immediately.

  


## Case 4.1.3

Up

**Case Description**  
  
Send small text message, then send frame with reserved non-control **Opcode = 5**, then send Ping.

**Case Expectation**  
  
Echo for first message is received, but then connection is failed immediately, since reserved opcode frame is used. A Pong is not received.

  


## Case 4.1.4

Up

**Case Description**  
  
Send small text message, then send frame with reserved non-control **Opcode = 6** and non-empty payload, then send Ping.

**Case Expectation**  
  
Echo for first message is received, but then connection is failed immediately, since reserved opcode frame is used. A Pong is not received.

  


## Case 4.1.5

Up

**Case Description**  
  
Send small text message, then send frame with reserved non-control **Opcode = 7** and non-empty payload, then send Ping.

**Case Expectation**  
  
Echo for first message is received, but then connection is failed immediately, since reserved opcode frame is used. A Pong is not received.

  


## Case 4.2.1

Up

**Case Description**  
  
Send frame with reserved control **Opcode = 11**.

**Case Expectation**  
  
The connection is failed immediately.

  


## Case 4.2.2

Up

**Case Description**  
  
Send frame with reserved control **Opcode = 12** and non-empty payload.

**Case Expectation**  
  
The connection is failed immediately.

  


## Case 4.2.3

Up

**Case Description**  
  
Send small text message, then send frame with reserved control **Opcode = 13**, then send Ping.

**Case Expectation**  
  
Echo for first message is received, but then connection is failed immediately, since reserved opcode frame is used. A Pong is not received.

  


## Case 4.2.4

Up

**Case Description**  
  
Send small text message, then send frame with reserved control **Opcode = 14** and non-empty payload, then send Ping.

**Case Expectation**  
  
Echo for first message is received, but then connection is failed immediately, since reserved opcode frame is used. A Pong is not received.

  


## Case 4.2.5

Up

**Case Description**  
  
Send small text message, then send frame with reserved control **Opcode = 15** and non-empty payload, then send Ping.

**Case Expectation**  
  
Echo for first message is received, but then connection is failed immediately, since reserved opcode frame is used. A Pong is not received.

  


## Case 5.1

Up

**Case Description**  
  
Send Ping fragmented into 2 fragments.

**Case Expectation**  
  
Connection is failed immediately, since control message MUST NOT be fragmented.

  


## Case 5.2

Up

**Case Description**  
  
Send Pong fragmented into 2 fragments.

**Case Expectation**  
  
Connection is failed immediately, since control message MUST NOT be fragmented.

  


## Case 5.3

Up

**Case Description**  
  
Send text Message fragmented into 2 fragments.

**Case Expectation**  
  
Message is processed and echo'ed back to us.

  


## Case 5.4

Up

**Case Description**  
  
Send text Message fragmented into 2 fragments, octets are sent in frame-wise chops.

**Case Expectation**  
  
Message is processed and echo'ed back to us.

  


## Case 5.5

Up

**Case Description**  
  
Send text Message fragmented into 2 fragments, octets are sent in octet-wise chops.

**Case Expectation**  
  
Message is processed and echo'ed back to us.

  


## Case 5.6

Up

**Case Description**  
  
Send text Message fragmented into 2 fragments, one ping with payload in-between.

**Case Expectation**  
  
A pong is received, then the message is echo'ed back to us.

  


## Case 5.7

Up

**Case Description**  
  
Send text Message fragmented into 2 fragments, one ping with payload in-between. Octets are sent in frame-wise chops.

**Case Expectation**  
  
A pong is received, then the message is echo'ed back to us.

  


## Case 5.8

Up

**Case Description**  
  
Send text Message fragmented into 2 fragments, one ping with payload in-between. Octets are sent in octet-wise chops.

**Case Expectation**  
  
A pong is received, then the message is echo'ed back to us.

  


## Case 5.9

Up

**Case Description**  
  
Send unfragmented Text Message after Continuation Frame with FIN = true, where there is nothing to continue, sent in one chop.

**Case Expectation**  
  
The connection is failed immediately, since there is no message to continue.

  


## Case 5.10

Up

**Case Description**  
  
Send unfragmented Text Message after Continuation Frame with FIN = true, where there is nothing to continue, sent in per-frame chops.

**Case Expectation**  
  
The connection is failed immediately, since there is no message to continue.

  


## Case 5.11

Up

**Case Description**  
  
Send unfragmented Text Message after Continuation Frame with FIN = true, where there is nothing to continue, sent in octet-wise chops.

**Case Expectation**  
  
The connection is failed immediately, since there is no message to continue.

  


## Case 5.12

Up

**Case Description**  
  
Send unfragmented Text Message after Continuation Frame with FIN = false, where there is nothing to continue, sent in one chop.

**Case Expectation**  
  
The connection is failed immediately, since there is no message to continue.

  


## Case 5.13

Up

**Case Description**  
  
Send unfragmented Text Message after Continuation Frame with FIN = false, where there is nothing to continue, sent in per-frame chops.

**Case Expectation**  
  
The connection is failed immediately, since there is no message to continue.

  


## Case 5.14

Up

**Case Description**  
  
Send unfragmented Text Message after Continuation Frame with FIN = false, where there is nothing to continue, sent in octet-wise chops.

**Case Expectation**  
  
The connection is failed immediately, since there is no message to continue.

  


## Case 5.15

Up

**Case Description**  
  
Send text Message fragmented into 2 fragments, then Continuation Frame with FIN = false where there is nothing to continue, then unfragmented Text Message, all sent in one chop.

**Case Expectation**  
  
The connection is failed immediately, since there is no message to continue.

  


## Case 5.16

Up

**Case Description**  
  
Repeated 2x: Continuation Frame with FIN = false (where there is nothing to continue), then text Message fragmented into 2 fragments.

**Case Expectation**  
  
The connection is failed immediately, since there is no message to continue.

  


## Case 5.17

Up

**Case Description**  
  
Repeated 2x: Continuation Frame with FIN = true (where there is nothing to continue), then text Message fragmented into 2 fragments.

**Case Expectation**  
  
The connection is failed immediately, since there is no message to continue.

  


## Case 5.18

Up

**Case Description**  
  
Send text Message fragmented into 2 fragments, with both frame opcodes set to text, sent in one chop.

**Case Expectation**  
  
The connection is failed immediately, since all data frames after the initial data frame must have opcode 0.

  


## Case 5.19

Up

**Case Description**  
  
A fragmented text message is sent in multiple frames. After
sending the first 2 frames of the text message, a Ping is sent. Then we wait 1s,
then we send 2 more text fragments, another Ping and then the final text fragment.
Everything is legal.

**Case Expectation**  
  
The peer immediately answers the first Ping before
it has received the last text message fragment. The peer pong's back the Ping's
payload exactly, and echo's the payload of the fragmented message back to us.

  


## Case 5.20

Up

**Case Description**  
  
Same as Case 5.19, but send all frames with SYNC = True.
Note, this does not change the octets sent in any way, only how the stream
is chopped up on the wire.

**Case Expectation**  
  
Same as Case 5.19. Implementations must be agnostic to how
octet stream is chopped up on wire (must be TCP clean).

  


## Case 6.1.1

Up

**Case Description**  
  
Send text message of length 0.

**Case Expectation**  
  
A message is echo'ed back to us (with empty payload).

  


## Case 6.1.2

Up

**Case Description**  
  
Send fragmented text message, 3 fragments each of length 0.

**Case Expectation**  
  
A message is echo'ed back to us (with empty payload).

  


## Case 6.1.3

Up

**Case Description**  
  
Send fragmented text message, 3 fragments, first and last of length 0, middle non-empty.

**Case Expectation**  
  
A message is echo'ed back to us (with payload = payload of middle fragment).

  


## Case 6.2.1

Up

**Case Description**  
  
Send a valid UTF-8 text message in one fragment.  
  
MESSAGE:  
Hello-Âµ@ÃÃ¶Ã¤Ã¼Ã Ã¡-UTF-8!!  
48656c6c6f2dc2b540c39fc3b6c3a4c3bcc3a0c3a12d5554462d382121

**Case Expectation**

  

---
