# Supplementary material for: Rigbox: An Open-Source Toolbox for Probing Neurons and Behavior
Source: eNeuro. 2020 Jul 9;7(4):ENEURO.0406-19.2020. doi: 10.1523/ENEURO.0406-19.2020 (PMC7363478; doi:10.1523/ENEURO.0406-19.2020)
Supplement: Extended Data 1 — We recommend looking at and downloading the code directly from the github repository, at https://github.com/cortex-lab/Rigbox. Download Extended Data 1, ZIP file. [file enu-eN-OTM-0406-19-s02.zip › Rigbox-master-2020-05-18/Rigbox-master/cb-tools/java-source/autobahn reports/servers/tootallnate_websocket_case_3_2.html]

  


  

tootallnate/websocket - **Case 3.2** : Non-Strict - **2** ms @ 2012-02-04T15:41:06Z

**Case Description**  
  
Send small text message, then send again with **RSV = 2**, then send Ping.

**Case Expectation**  
  
Echo for first message is received, but then connection is failed immediately, since RSV must be 0, when no extension defining RSV meaning has been negoiated. The Pong is not received.

**Case Outcome**  
  
Actual events match at least one expected.  
  
*Expected:*  
{'NON-STRICT': [], 'OK': [('message', 'Hello, world!', False)]}  
  
*Observed:*  
[]

**Case Closing Behavior**

  

---

## Opening Handshake

```
GET / HTTP/1.1

User-Agent: AutobahnWebSocketsTestSuite/0.4.10

Host: localhost:9003

Upgrade: websocket

Connection: Upgrade

Sec-WebSocket-Key: naQpeQXM16oTsQGgFhzkCw==

Sec-WebSocket-Version: 13
```

```
HTTP/1.1 101 Switching Protocols

Upgrade: websocket

Connection: Upgrade

Sec-WebSocket-Accept: GWrmDJGkZekAGDtxltB9SFScv/0=
```

  

---

## Closing Behavior

|  |  |  |
| --- | --- | --- |
| Key | Value | Description |
| isServer | False | True, iff I (the fuzzer) am a server, and the peer is a client. |
| closedByMe | False | True, iff I have initiated closing handshake (that is, did send close first). |
| failedByMe | False | True, iff I have failed the WS connection (i.e. due to protocol error). Failing can be either by initiating closing handshake or brutal drop TCP. |
| droppedByMe | False | True, iff I dropped the TCP connection. |
| wasClean | True | True, iff full WebSockets closing handshake was performed (close frame sent and received) \_and\_ the server dropped the TCP (which is its responsibility). |
| wasNotCleanReason | None | When wasClean == False, the reason what happened. |
| wasServerConnectionDropTimeout | False | When we are a client, and we expected the server to drop the TCP, but that didn't happen in time, this gets True. |
| wasCloseHandshakeTimeout | False | When we initiated a closing handshake, but the peer did not respond in time, this gets True. |
| localCloseCode | 1000 | The close code I sent in close frame (if any). |
| localCloseReason | None | The close reason I sent in close frame (if any). |
| remoteCloseCode | None | The close code the peer sent me in close frame (if any). |
| remoteCloseReason | None | The close reason the peer sent me in close frame (if any). |

  

---

## Wire Statistics

### Octets Received by Chop Size

|  |  |  |
| --- | --- | --- |
| Chop Size | Count | Octets |
| 2 | 1 | 2 |
| 129 | 1 | 129 |
| Total | 2 | 131 |

### Octets Transmitted by Chop Size

|  |  |  |
| --- | --- | --- |
| Chop Size | Count | Octets |
| 6 | 1 | 6 |
| 8 | 1 | 8 |
| 19 | 2 | 38 |
| 201 | 1 | 201 |
| Total | 5 | 253 |

### Frames Received by Opcode

|  |  |
| --- | --- |
| Opcode | Count |
| 8 | 1 |
| Total | 1 |

### Frames Transmitted by Opcode

|  |  |
| --- | --- |
| Opcode | Count |
| 1 | 2 |
| 8 | 1 |
| 9 | 1 |
| Total | 4 |

  

---

## Wire Log

```
000 TX OCTETS: 474554202f20485454502f312e310d0a557365722d4167656e743a204175746f6261686e576562536f636b65747354657374
```

```
               53756974652f302e342e31300d0a486f73743a206c6f63616c686f73743a393030330d0a557067726164653a20776562736f
```

```
               636b65740d0a436f6e6e656374696f6e3a20557067726164650d0a5365632d576562536f636b65742d4b65793a206e615170
```

```
               6551584d31366f547351476746687a6b43773d3d0d0a5365632d576562536f636b65742d56657273696f6e3a2031330d0a0d
```

```
               0a
```

```
001 RX OCTETS: 485454502f312e312031303120537769746368696e672050726f746f636f6c730d0a557067726164653a20776562736f636b
```

```
               65740d0a436f6e6e656374696f6e3a20557067726164650d0a5365632d576562536f636b65742d4163636570743a20475772
```

```
               6d444a476b5a656b41474474786c74423953465363762f303d0d0a0d0a
```

```
002 TX FRAME : OPCODE=1, FIN=True, RSV=0, MASK=c8e7cd39, PAYLOAD-REPEAT-LEN=None, CHOPSIZE=None, SYNC=False
```

```
               Hello, world!
```

```
003 TX OCTETS: 818dc8e7cd398082a155a7cbed4ea795a15de9
```

```
004 TX FRAME : OPCODE=1, FIN=True, RSV=2, MASK=48cf9b10, PAYLOAD-REPEAT-LEN=None, CHOPSIZE=None, SYNC=False
```

```
               Hello, world!
```

```
005 TX OCTETS: a18d48cf9b1000aaf77c27e3bb6727bdf77469
```

```
006 TX FRAME : OPCODE=9, FIN=True, RSV=0, MASK=ba7b6257, PAYLOAD-REPEAT-LEN=None, CHOPSIZE=None, SYNC=False
```

```
007 TX OCTETS: 8980ba7b6257
```

```
008 FAIL CONNECTION AFTER 1.000000 sec
```

```
009 RX OCTETS: 8800
```

```
010 RX FRAME : OPCODE=8, FIN=True, RSV=0, MASKED=False, MASK=None
```

```
011 TX FRAME : OPCODE=8, FIN=True, RSV=0, MASK=c2125d8a, PAYLOAD-REPEAT-LEN=None, CHOPSIZE=None, SYNC=False
```

```
               �
```

```
012 TX OCTETS: 8882c2125d8ac1fa
```

```
013 TCP DROPPED BY PEER
```

  

---
