# Supplementary material for: Rigbox: An Open-Source Toolbox for Probing Neurons and Behavior
Source: eNeuro. 2020 Jul 9;7(4):ENEURO.0406-19.2020. doi: 10.1523/ENEURO.0406-19.2020 (PMC7363478; doi:10.1523/ENEURO.0406-19.2020)
Supplement: Extended Data 1 — We recommend looking at and downloading the code directly from the github repository, at https://github.com/cortex-lab/Rigbox. Download Extended Data 1, ZIP file. [file enu-eN-OTM-0406-19-s02.zip › Rigbox-master-2020-05-18/Rigbox-master/cb-tools/java-source/autobahn reports/servers/tootallnate_websocket_case_4_1_4.html]

  


  

tootallnate/websocket - **Case 4.1.4** : Non-Strict - **2** ms @ 2012-02-04T15:41:06Z

**Case Description**  
  
Send small text message, then send frame with reserved non-control **Opcode = 6** and non-empty payload, then send Ping.

**Case Expectation**  
  
Echo for first message is received, but then connection is failed immediately, since reserved opcode frame is used. A Pong is not received.

**Case Outcome**  
  
Actual events match at least one expected.  
  
*Expected:*  
{'NON-STRICT': [], 'OK': [('message', 'Hello, world!', False)]}  
  
*Observed:*  
[]

**Case Closing Behavior**

  

---

## Opening Handshake

```
GET / HTTP/1.1

User-Agent: AutobahnWebSocketsTestSuite/0.4.10

Host: localhost:9003

Upgrade: websocket

Connection: Upgrade

Sec-WebSocket-Key: lWjk0P7OM1q+JBUPL0HfZA==

Sec-WebSocket-Version: 13
```

```
HTTP/1.1 101 Switching Protocols

Upgrade: websocket

Connection: Upgrade

Sec-WebSocket-Accept: d7k1A19qGfNKwuf0DJJ9pJTFpII=
```

  

---

## Closing Behavior

|  |  |  |
| --- | --- | --- |
| Key | Value | Description |
| isServer | False | True, iff I (the fuzzer) am a server, and the peer is a client. |
| closedByMe | False | True, iff I have initiated closing handshake (that is, did send close first). |
| failedByMe | False | True, iff I have failed the WS connection (i.e. due to protocol error). Failing can be either by initiating closing handshake or brutal drop TCP. |
| droppedByMe | False | True, iff I dropped the TCP connection. |
| wasClean | True | True, iff full WebSockets closing handshake was performed (close frame sent and received) \_and\_ the server dropped the TCP (which is its responsibility). |
| wasNotCleanReason | None | When wasClean == False, the reason what happened. |
| wasServerConnectionDropTimeout | False | When we are a client, and we expected the server to drop the TCP, but that didn't happen in time, this gets True. |
| wasCloseHandshakeTimeout | False | When we initiated a closing handshake, but the peer did not respond in time, this gets True. |
| localCloseCode | 1000 | The close code I sent in close frame (if any). |
| localCloseReason | None | The close reason I sent in close frame (if any). |
| remoteCloseCode | None | The close code the peer sent me in close frame (if any). |
| remoteCloseReason | None | The close reason the peer sent me in close frame (if any). |

  

---

## Wire Statistics

### Octets Received by Chop Size

|  |  |  |
| --- | --- | --- |
| Chop Size | Count | Octets |
| 2 | 1 | 2 |
| 129 | 1 | 129 |
| Total | 2 | 131 |

### Octets Transmitted by Chop Size

|  |  |  |
| --- | --- | --- |
| Chop Size | Count | Octets |
| 6 | 1 | 6 |
| 8 | 1 | 8 |
| 19 | 2 | 38 |
| 201 | 1 | 201 |
| Total | 5 | 253 |

### Frames Received by Opcode

|  |  |
| --- | --- |
| Opcode | Count |
| 8 | 1 |
| Total | 1 |

### Frames Transmitted by Opcode

|  |  |
| --- | --- |
| Opcode | Count |
| 1 | 1 |
| 6 | 1 |
| 8 | 1 |
| 9 | 1 |
| Total | 4 |

  

---

## Wire Log

```
000 TX OCTETS: 474554202f20485454502f312e310d0a557365722d4167656e743a204175746f6261686e576562536f636b65747354657374
```

```
               53756974652f302e342e31300d0a486f73743a206c6f63616c686f73743a393030330d0a557067726164653a20776562736f
```

```
               636b65740d0a436f6e6e656374696f6e3a20557067726164650d0a5365632d576562536f636b65742d4b65793a206c576a6b
```

```
               3050374f4d31712b4a4255504c3048665a413d3d0d0a5365632d576562536f636b65742d56657273696f6e3a2031330d0a0d
```

```
               0a
```

```
001 RX OCTETS: 485454502f312e312031303120537769746368696e672050726f746f636f6c730d0a557067726164653a20776562736f636b
```

```
               65740d0a436f6e6e656374696f6e3a20557067726164650d0a5365632d576562536f636b65742d4163636570743a2064376b
```

```
               314131397147664e4b77756630444a4a39704a54467049493d0d0a0d0a
```

```
002 TX FRAME : OPCODE=1, FIN=True, RSV=0, MASK=45843e64, PAYLOAD-REPEAT-LEN=None, CHOPSIZE=None, SYNC=False
```

```
               Hello, world!
```

```
003 TX OCTETS: 818d45843e640de152082aa81e132af6520064
```

```
004 TX FRAME : OPCODE=6, FIN=True, RSV=0, MASK=fa380a4b, PAYLOAD-REPEAT-LEN=None, CHOPSIZE=None, SYNC=False
```

```
               Hello, world!
```

```
005 TX OCTETS: 868dfa380a4bb25d662795142a3c954a662fdb
```

```
006 TX FRAME : OPCODE=9, FIN=True, RSV=0, MASK=cfe1af5c, PAYLOAD-REPEAT-LEN=None, CHOPSIZE=None, SYNC=False
```

```
007 TX OCTETS: 8980cfe1af5c
```

```
008 FAIL CONNECTION AFTER 1.000000 sec
```

```
009 RX OCTETS: 8800
```

```
010 RX FRAME : OPCODE=8, FIN=True, RSV=0, MASKED=False, MASK=None
```

```
011 TX FRAME : OPCODE=8, FIN=True, RSV=0, MASK=4f85959a, PAYLOAD-REPEAT-LEN=None, CHOPSIZE=None, SYNC=False
```

```
               �
```

```
012 TX OCTETS: 88824f85959a4c6d
```

```
013 TCP DROPPED BY PEER
```

  

---
