# Supplementary material for: Rigbox: An Open-Source Toolbox for Probing Neurons and Behavior
Source: eNeuro. 2020 Jul 9;7(4):ENEURO.0406-19.2020. doi: 10.1523/ENEURO.0406-19.2020 (PMC7363478; doi:10.1523/ENEURO.0406-19.2020)
Supplement: Extended Data 1 — We recommend looking at and downloading the code directly from the github repository, at https://github.com/cortex-lab/Rigbox. Download Extended Data 1, ZIP file. [file enu-eN-OTM-0406-19-s02.zip › Rigbox-master-2020-05-18/Rigbox-master/cb-tools/java-source/autobahn reports/servers/tootallnate_websocket_case_6_20_2.html]

  


  

tootallnate/websocket - **Case 6.20.2** : Fail - **501** ms @ 2012-02-04T15:41:19Z

**Case Description**  
  
Send a text message with payload which is not valid UTF-8 in one fragment.  
  
MESSAGE:  
í­¿  
edadbf

**Case Expectation**

**Case Outcome**

**Case Closing Behavior**

  

---

## Opening Handshake

```
GET / HTTP/1.1

User-Agent: AutobahnWebSocketsTestSuite/0.4.10

Host: localhost:9003

Upgrade: websocket

Connection: Upgrade

Sec-WebSocket-Key: TIQj7T90U3MBd7UDV/Tkeg==

Sec-WebSocket-Version: 13
```

```
HTTP/1.1 101 Switching Protocols

Upgrade: websocket

Connection: Upgrade

Sec-WebSocket-Accept: mKV6+d9BjSDvtA484tge96+03HE=
```

  

---

## Closing Behavior

|  |  |  |
| --- | --- | --- |
| Key | Value | Description |
| isServer | False | True, iff I (the fuzzer) am a server, and the peer is a client. |
| closedByMe | True | True, iff I have initiated closing handshake (that is, did send close first). |
| failedByMe | True | True, iff I have failed the WS connection (i.e. due to protocol error). Failing can be either by initiating closing handshake or brutal drop TCP. |
| droppedByMe | False | True, iff I dropped the TCP connection. |
| wasClean | True | True, iff full WebSockets closing handshake was performed (close frame sent and received) \_and\_ the server dropped the TCP (which is its responsibility). |
| wasNotCleanReason | None | When wasClean == False, the reason what happened. |
| wasServerConnectionDropTimeout | False | When we are a client, and we expected the server to drop the TCP, but that didn't happen in time, this gets True. |
| wasCloseHandshakeTimeout | False | When we initiated a closing handshake, but the peer did not respond in time, this gets True. |
| localCloseCode | 1001 | The close code I sent in close frame (if any). |
| localCloseReason | Going Away | The close reason I sent in close frame (if any). |
| remoteCloseCode | None | The close code the peer sent me in close frame (if any). |
| remoteCloseReason | None | The close reason the peer sent me in close frame (if any). |

  

---

## Wire Statistics

### Octets Received by Chop Size

|  |  |  |
| --- | --- | --- |
| Chop Size | Count | Octets |
| 2 | 1 | 2 |
| 3 | 1 | 3 |
| 129 | 1 | 129 |
| Total | 3 | 134 |

### Octets Transmitted by Chop Size

|  |  |  |
| --- | --- | --- |
| Chop Size | Count | Octets |
| 9 | 1 | 9 |
| 18 | 1 | 18 |
| 201 | 1 | 201 |
| Total | 3 | 228 |

### Frames Received by Opcode

|  |  |
| --- | --- |
| Opcode | Count |
| 1 | 1 |
| 8 | 1 |
| Total | 2 |

### Frames Transmitted by Opcode

|  |  |
| --- | --- |
| Opcode | Count |
| 1 | 1 |
| 8 | 1 |
| Total | 2 |

  

---

## Wire Log

```
000 TX OCTETS: 474554202f20485454502f312e310d0a557365722d4167656e743a204175746f6261686e576562536f636b65747354657374
```

```
               53756974652f302e342e31300d0a486f73743a206c6f63616c686f73743a393030330d0a557067726164653a20776562736f
```

```
               636b65740d0a436f6e6e656374696f6e3a20557067726164650d0a5365632d576562536f636b65742d4b65793a205449516a
```

```
               3754393055334d4264375544562f546b65673d3d0d0a5365632d576562536f636b65742d56657273696f6e3a2031330d0a0d
```

```
               0a
```

```
001 RX OCTETS: 485454502f312e312031303120537769746368696e672050726f746f636f6c730d0a557067726164653a20776562736f636b
```

```
               65740d0a436f6e6e656374696f6e3a20557067726164650d0a5365632d576562536f636b65742d4163636570743a206d4b56
```

```
               362b6439426a534476744134383474676539362b303348453d0d0a0d0a
```

```
002 TX FRAME : OPCODE=1, FIN=True, RSV=0, MASK=5711da46, PAYLOAD-REPEAT-LEN=None, CHOPSIZE=None, SYNC=False
```

```
               í­¿
```

```
003 TX OCTETS: 81835711da46babc65
```

```
004 FAIL CONNECTION AFTER 0.500000 sec
```

```
005 RX OCTETS: 81013f
```

```
006 RX FRAME : OPCODE=1, FIN=True, RSV=0, MASKED=False, MASK=None
```

```
               ?
```

```
007 FAILING CONNECTION
```

```
008 TX FRAME : OPCODE=8, FIN=True, RSV=0, MASK=d65c7a61, PAYLOAD-REPEAT-LEN=None, CHOPSIZE=None, SYNC=False
```

```
               éGoing Away
```

```
009 TX OCTETS: 888cd65c7a61d5b53d0ebf321d41972b1b18
```

```
010 RX OCTETS: 8800
```

```
011 RX FRAME : OPCODE=8, FIN=True, RSV=0, MASKED=False, MASK=None
```

```
012 TCP DROPPED BY PEER
```

  

---
