# Supplementary material for: Rigbox: An Open-Source Toolbox for Probing Neurons and Behavior
Source: eNeuro. 2020 Jul 9;7(4):ENEURO.0406-19.2020. doi: 10.1523/ENEURO.0406-19.2020 (PMC7363478; doi:10.1523/ENEURO.0406-19.2020)
Supplement: Extended Data 1 — We recommend looking at and downloading the code directly from the github repository, at https://github.com/cortex-lab/Rigbox. Download Extended Data 1, ZIP file. [file enu-eN-OTM-0406-19-s02.zip › Rigbox-master-2020-05-18/Rigbox-master/cb-tools/java-source/autobahn reports/servers/tootallnate_websocket_case_6_3_2.html]

  


  

tootallnate/websocket - **Case 6.3.2** : Fail - **1002** ms @ 2012-02-04T15:41:09Z

**Case Description**  
  
Send invalid UTF-8 text message in fragments of 1 octet, resulting in frames ending on positions which are not code point ends.  
  
MESSAGE:  
Îºá½¹ÏƒÎ¼Îµí €edited  
cebae1bdb9cf83cebcceb5eda080656469746564

**Case Expectation**

**Case Outcome**

**Case Closing Behavior**

  

---

## Opening Handshake

```
GET / HTTP/1.1

User-Agent: AutobahnWebSocketsTestSuite/0.4.10

Host: localhost:9003

Upgrade: websocket

Connection: Upgrade

Sec-WebSocket-Key: +0fzXkyvkSjPhQlQQX66GA==

Sec-WebSocket-Version: 13
```

```
HTTP/1.1 101 Switching Protocols

Upgrade: websocket

Connection: Upgrade

Sec-WebSocket-Accept: LgO0/Am1g9xEfcTU2myHtnWvixQ=
```

  

---

## Closing Behavior

|  |  |  |
| --- | --- | --- |
| Key | Value | Description |
| isServer | False | True, iff I (the fuzzer) am a server, and the peer is a client. |
| closedByMe | True | True, iff I have initiated closing handshake (that is, did send close first). |
| failedByMe | True | True, iff I have failed the WS connection (i.e. due to protocol error). Failing can be either by initiating closing handshake or brutal drop TCP. |
| droppedByMe | False | True, iff I dropped the TCP connection. |
| wasClean | True | True, iff full WebSockets closing handshake was performed (close frame sent and received) \_and\_ the server dropped the TCP (which is its responsibility). |
| wasNotCleanReason | None | When wasClean == False, the reason what happened. |
| wasServerConnectionDropTimeout | False | When we are a client, and we expected the server to drop the TCP, but that didn't happen in time, this gets True. |
| wasCloseHandshakeTimeout | False | When we initiated a closing handshake, but the peer did not respond in time, this gets True. |
| localCloseCode | 1001 | The close code I sent in close frame (if any). |
| localCloseReason | Going Away | The close reason I sent in close frame (if any). |
| remoteCloseCode | None | The close code the peer sent me in close frame (if any). |
| remoteCloseReason | None | The close reason the peer sent me in close frame (if any). |

  

---

## Wire Statistics

### Octets Received by Chop Size

|  |  |  |
| --- | --- | --- |
| Chop Size | Count | Octets |
| 2 | 1 | 2 |
| 20 | 1 | 20 |
| 129 | 1 | 129 |
| Total | 3 | 151 |

### Octets Transmitted by Chop Size

|  |  |  |
| --- | --- | --- |
| Chop Size | Count | Octets |
| 6 | 1 | 6 |
| 7 | 20 | 140 |
| 18 | 1 | 18 |
| 201 | 1 | 201 |
| Total | 23 | 365 |

### Frames Received by Opcode

|  |  |
| --- | --- |
| Opcode | Count |
| 1 | 1 |
| 8 | 1 |
| Total | 2 |

### Frames Transmitted by Opcode

|  |  |
| --- | --- |
| Opcode | Count |
| 0 | 20 |
| 1 | 1 |
| 8 | 1 |
| Total | 22 |

  

---

## Wire Log

```
000 TX OCTETS: 474554202f20485454502f312e310d0a557365722d4167656e743a204175746f6261686e576562536f636b65747354657374
```

```
               53756974652f302e342e31300d0a486f73743a206c6f63616c686f73743a393030330d0a557067726164653a20776562736f
```

```
               636b65740d0a436f6e6e656374696f6e3a20557067726164650d0a5365632d576562536f636b65742d4b65793a202b30667a
```

```
               586b79766b536a5068516c515158363647413d3d0d0a5365632d576562536f636b65742d56657273696f6e3a2031330d0a0d
```

```
               0a
```

```
001 RX OCTETS: 485454502f312e312031303120537769746368696e672050726f746f636f6c730d0a557067726164653a20776562736f636b
```

```
               65740d0a436f6e6e656374696f6e3a20557067726164650d0a5365632d576562536f636b65742d4163636570743a204c674f
```

```
               302f416d316739784566635455326d7948746e57766978513d0d0a0d0a
```

```
002 TX FRAME : OPCODE=1, FIN=False, RSV=0, MASK=b2f15302, PAYLOAD-REPEAT-LEN=None, CHOPSIZE=None, SYNC=False
```

```
               Î
```

```
003 TX OCTETS: 0181b2f153027c
```

```
004 TX FRAME : OPCODE=0, FIN=False, RSV=0, MASK=c90d6517, PAYLOAD-REPEAT-LEN=None, CHOPSIZE=None, SYNC=False
```

```
               º
```

```
005 TX OCTETS: 0081c90d651773
```

```
006 TX FRAME : OPCODE=0, FIN=False, RSV=0, MASK=cd667264, PAYLOAD-REPEAT-LEN=None, CHOPSIZE=None, SYNC=False
```

```
               á
```

```
007 TX OCTETS: 0081cd6672642c
```

```
008 TX FRAME : OPCODE=0, FIN=False, RSV=0, MASK=024afe11, PAYLOAD-REPEAT-LEN=None, CHOPSIZE=None, SYNC=False
```

```
               ½
```

```
009 TX OCTETS: 0081024afe11bf
```

```
010 TX FRAME : OPCODE=0, FIN=False, RSV=0, MASK=80b6c518, PAYLOAD-REPEAT-LEN=None, CHOPSIZE=None, SYNC=False
```

```
               ¹
```

```
011 TX OCTETS: 008180b6c51839
```

```
012 TX FRAME : OPCODE=0, FIN=False, RSV=0, MASK=62a7724e, PAYLOAD-REPEAT-LEN=None, CHOPSIZE=None, SYNC=False
```

```
               Ï
```

```
013 TX OCTETS: 008162a7724ead
```

```
014 TX FRAME : OPCODE=0, FIN=False, RSV=0, MASK=42f77b2b, PAYLOAD-REPEAT-LEN=None, CHOPSIZE=None, SYNC=False
```

```
               ƒ
```

```
015 TX OCTETS: 008142f77b2bc1
```

```
016 TX FRAME : OPCODE=0, FIN=False, RSV=0, MASK=c1f0a0f1, PAYLOAD-REPEAT-LEN=None, CHOPSIZE=None, SYNC=False
```

```
               Î
```

```
017 TX OCTETS: 0081c1f0a0f10f
```

```
018 TX FRAME : OPCODE=0, FIN=False, RSV=0, MASK=7f9e2838, PAYLOAD-REPEAT-LEN=None, CHOPSIZE=None, SYNC=False
```

```
               ¼
```

```
019 TX OCTETS: 00817f9e2838c3
```

```
020 TX FRAME : OPCODE=0, FIN=False, RSV=0, MASK=06f47c2c, PAYLOAD-REPEAT-LEN=None, CHOPSIZE=None, SYNC=False
```

```
               Î
```

```
021 TX OCTETS: 008106f47c2cc8
```

```
022 TX FRAME : OPCODE=0, FIN=False, RSV=0, MASK=e139a1e1, PAYLOAD-REPEAT-LEN=None, CHOPSIZE=None, SYNC=False
```

```
               µ
```

```
023 TX OCTETS: 0081e139a1e154
```

```
024 TX FRAME : OPCODE=0, FIN=False, RSV=0, MASK=d00820e1, PAYLOAD-REPEAT-LEN=None, CHOPSIZE=None, SYNC=False
```

```
               í
```

```
025 TX OCTETS: 0081d00820e13d
```

```
026 TX FRAME : OPCODE=0, FIN=False, RSV=0, MASK=a93ed31b, PAYLOAD-REPEAT-LEN=None, CHOPSIZE=None, SYNC=False
```

```
027 TX OCTETS: 0081a93ed31b09
```

```
028 TX FRAME : OPCODE=0, FIN=False, RSV=0, MASK=86fe9fa7, PAYLOAD-REPEAT-LEN=None, CHOPSIZE=None, SYNC=False
```

```
               €
```

```
029 TX OCTETS: 008186fe9fa706
```

```
030 TX FRAME : OPCODE=0, FIN=False, RSV=0, MASK=da4f747e, PAYLOAD-REPEAT-LEN=None, CHOPSIZE=None, SYNC=False
```

```
               e
```

```
031 TX OCTETS: 0081da4f747ebf
```

```
032 TX FRAME : OPCODE=0, FIN=False, RSV=0, MASK=0906c2df, PAYLOAD-REPEAT-LEN=None, CHOPSIZE=None, SYNC=False
```

```
               d
```

```
033 TX OCTETS: 00810906c2df6d
```

```
034 TX FRAME : OPCODE=0, FIN=False, RSV=0, MASK=9c8128e5, PAYLOAD-REPEAT-LEN=None, CHOPSIZE=None, SYNC=False
```

```
               i
```

```
035 TX OCTETS: 00819c8128e5f5
```

```
036 TX FRAME : OPCODE=0, FIN=False, RSV=0, MASK=c0b043ed, PAYLOAD-REPEAT-LEN=None, CHOPSIZE=None, SYNC=False
```

```
               t
```

```
037 TX OCTETS: 0081c0b043edb4
```

```
038 TX FRAME : OPCODE=0, FIN=False, RSV=0, MASK=1723209b, PAYLOAD-REPEAT-LEN=None, CHOPSIZE=None, SYNC=False
```

```
               e
```

```
039 TX OCTETS: 00811723209b72
```

```
040 TX FRAME : OPCODE=0, FIN=False, RSV=0, MASK=a1194c70, PAYLOAD-REPEAT-LEN=None, CHOPSIZE=None, SYNC=False
```

```
               d
```

```
041 TX OCTETS: 0081a1194c70c5
```

```
042 TX FRAME : OPCODE=0, FIN=True, RSV=0, MASK=73e98929, PAYLOAD-REPEAT-LEN=None, CHOPSIZE=None, SYNC=False
```

```
043 TX OCTETS: 808073e98929
```

```
044 FAIL CONNECTION AFTER 1.000000 sec
```

```
045 RX OCTETS: 8112cebae1bdb9cf83cebcceb53f656469746564
```

```
046 RX FRAME : OPCODE=1, FIN=True, RSV=0, MASKED=False, MASK=None
```

```
               Îºá½¹ÏƒÎ¼Îµ?edited
```

```
047 FAILING CONNECTION
```

```
048 TX FRAME : OPCODE=8, FIN=True, RSV=0, MASK=52a40872, PAYLOAD-REPEAT-LEN=None, CHOPSIZE=None, SYNC=False
```

```
               éGoing Away
```

```
049 TX OCTETS: 888c52a40872514d4f1d3bca6f5213d3690b
```

```
050 RX OCTETS: 8800
```

```
051 RX FRAME : OPCODE=8, FIN=True, RSV=0, MASKED=False, MASK=None
```

```
052 TCP DROPPED BY PEER
```

  

---
