# Supplementary material for: Rigbox: An Open-Source Toolbox for Probing Neurons and Behavior
Source: eNeuro. 2020 Jul 9;7(4):ENEURO.0406-19.2020. doi: 10.1523/ENEURO.0406-19.2020 (PMC7363478; doi:10.1523/ENEURO.0406-19.2020)
Supplement: Extended Data 1 — We recommend looking at and downloading the code directly from the github repository, at https://github.com/cortex-lab/Rigbox. Download Extended Data 1, ZIP file. [file enu-eN-OTM-0406-19-s02.zip › Rigbox-master-2020-05-18/Rigbox-master/cb-tools/java-source/autobahn reports/servers/tootallnate_websocket_case_6_4_1.html]

  


  

tootallnate/websocket - **Case 6.4.1** : Non-Strict - **2004** ms @ 2012-02-04T15:41:10Z

**Case Description**  
  
Send invalid UTF-8 text message in 3 fragments (frames).
First frame payload is valid, then wait, then 2nd frame which contains the payload making the sequence invalid, then wait, then 3rd frame with rest.
Note that PART1 and PART3 are valid UTF-8 in themselves, PART2 is a 0x11000 encoded as in the UTF-8 integer encoding scheme, but the codepoint is invalid (out of range).
  
  
MESSAGE PARTS:  
PART1 = Îºá½¹ÏÎ¼Îµ (cebae1bdb9cf83cebcceb5)  
PART2 = ô (f4908080)  
PART3 = edited (656469746564)

**Case Expectation**

**Case Outcome**

**Case Closing Behavior**

  

---

## Opening Handshake

```
GET / HTTP/1.1

User-Agent: AutobahnWebSocketsTestSuite/0.4.10

Host: localhost:9003

Upgrade: websocket

Connection: Upgrade

Sec-WebSocket-Key: UpAkZ//RWLrHfexyOMj3mw==

Sec-WebSocket-Version: 13
```

```
HTTP/1.1 101 Switching Protocols

Upgrade: websocket

Connection: Upgrade

Sec-WebSocket-Accept: HUMfuAwHoYZqlf78+bKZQrSw5t0=
```

  

---

## Closing Behavior

|  |  |  |
| --- | --- | --- |
| Key | Value | Description |
| isServer | False | True, iff I (the fuzzer) am a server, and the peer is a client. |
| closedByMe | False | True, iff I have initiated closing handshake (that is, did send close first). |
| failedByMe | False | True, iff I have failed the WS connection (i.e. due to protocol error). Failing can be either by initiating closing handshake or brutal drop TCP. |
| droppedByMe | False | True, iff I dropped the TCP connection. |
| wasClean | True | True, iff full WebSockets closing handshake was performed (close frame sent and received) \_and\_ the server dropped the TCP (which is its responsibility). |
| wasNotCleanReason | None | When wasClean == False, the reason what happened. |
| wasServerConnectionDropTimeout | False | When we are a client, and we expected the server to drop the TCP, but that didn't happen in time, this gets True. |
| wasCloseHandshakeTimeout | False | When we initiated a closing handshake, but the peer did not respond in time, this gets True. |
| localCloseCode | 1000 | The close code I sent in close frame (if any). |
| localCloseReason | None | The close reason I sent in close frame (if any). |
| remoteCloseCode | None | The close code the peer sent me in close frame (if any). |
| remoteCloseReason | None | The close reason the peer sent me in close frame (if any). |

  

---

## Wire Statistics

### Octets Received by Chop Size

|  |  |  |
| --- | --- | --- |
| Chop Size | Count | Octets |
| 2 | 1 | 2 |
| 129 | 1 | 129 |
| Total | 2 | 131 |

### Octets Transmitted by Chop Size

|  |  |  |
| --- | --- | --- |
| Chop Size | Count | Octets |
| 8 | 1 | 8 |
| 10 | 1 | 10 |
| 12 | 1 | 12 |
| 17 | 1 | 17 |
| 201 | 1 | 201 |
| Total | 5 | 248 |

### Frames Received by Opcode

|  |  |
| --- | --- |
| Opcode | Count |
| 8 | 1 |
| Total | 1 |

### Frames Transmitted by Opcode

|  |  |
| --- | --- |
| Opcode | Count |
| 0 | 2 |
| 1 | 1 |
| 8 | 1 |
| Total | 4 |

  

---

## Wire Log

```
000 TX OCTETS: 474554202f20485454502f312e310d0a557365722d4167656e743a204175746f6261686e576562536f636b65747354657374
```

```
               53756974652f302e342e31300d0a486f73743a206c6f63616c686f73743a393030330d0a557067726164653a20776562736f
```

```
               636b65740d0a436f6e6e656374696f6e3a20557067726164650d0a5365632d576562536f636b65742d4b65793a205570416b
```

```
               5a2f2f52574c7248666578794f4d6a336d773d3d0d0a5365632d576562536f636b65742d56657273696f6e3a2031330d0a0d
```

```
               0a
```

```
001 RX OCTETS: 485454502f312e312031303120537769746368696e672050726f746f636f6c730d0a557067726164653a20776562736f636b
```

```
               65740d0a436f6e6e656374696f6e3a20557067726164650d0a5365632d576562536f636b65742d4163636570743a2048554d
```

```
               66754177486f595a716c6637382b624b5a517253773574303d0d0a0d0a
```

```
002 TX FRAME : OPCODE=1, FIN=False, RSV=0, MASK=7ae1bfb5, PAYLOAD-REPEAT-LEN=None, CHOPSIZE=None, SYNC=False
```

```
               Îºá½¹ÏÎ¼Îµ
```

```
003 TX OCTETS: 018b7ae1bfb5b45b5e08c32e3c7bc62f0a
```

```
004 DELAY 1.000000 sec for TAG A
```

```
005 DELAY TIMEOUT on TAG A
```

```
006 TX FRAME : OPCODE=0, FIN=False, RSV=0, MASK=98cbbec9, PAYLOAD-REPEAT-LEN=None, CHOPSIZE=None, SYNC=False
```

```
               ô
```

```
007 TX OCTETS: 008498cbbec96c5b3e49
```

```
008 DELAY 1.000000 sec for TAG B
```

```
009 DELAY TIMEOUT on TAG B
```

```
010 TX FRAME : OPCODE=0, FIN=True, RSV=0, MASK=61ebbee3, PAYLOAD-REPEAT-LEN=None, CHOPSIZE=None, SYNC=False
```

```
               edited
```

```
011 TX OCTETS: 808661ebbee3048fd797048f
```

```
012 FAIL CONNECTION AFTER 1.000000 sec
```

```
013 RX OCTETS: 8800
```

```
014 RX FRAME : OPCODE=8, FIN=True, RSV=0, MASKED=False, MASK=None
```

```
015 TX FRAME : OPCODE=8, FIN=True, RSV=0, MASK=6bde347c, PAYLOAD-REPEAT-LEN=None, CHOPSIZE=None, SYNC=False
```

```
               è
```

```
016 TX OCTETS: 88826bde347c6836
```

```
017 TCP DROPPED BY PEER
```

  

---
