# Supplementary material for: Rigbox: An Open-Source Toolbox for Probing Neurons and Behavior
Source: eNeuro. 2020 Jul 9;7(4):ENEURO.0406-19.2020. doi: 10.1523/ENEURO.0406-19.2020 (PMC7363478; doi:10.1523/ENEURO.0406-19.2020)
Supplement: Extended Data 1 — We recommend looking at and downloading the code directly from the github repository, at https://github.com/cortex-lab/Rigbox. Download Extended Data 1, ZIP file. [file enu-eN-OTM-0406-19-s02.zip › Rigbox-master-2020-05-18/Rigbox-master/cb-tools/java-source/index.html]

TooTallNate/Java-WebSocket @ GitHub


# Java-WebSocket by TooTallNate

A barebones WebSocket client and server implementation written in 100% Java.

## Authors

Davidiusdadi (DavidRohmer@web.de)
  
Bob Corsaro (bob@embed.ly)
  
Don Park (don@donpark.org)
  
David Rohmer (firstlastname@web.de)
  
swax (john.m.marshall@gmail.com)
  
Jarrod Ribble (jribble@netiq.com)
  
Julian Gautier (julian.gautier@alumni.neumont.edu)
  
Kristijan Sedlak (k.sedlak@mandatela.si)
  
morkai (lukasz@walukiewicz.eu)
  
Nathaniel Michael (natecmichael@gmail.com)
  
Nathan Rajlich (nathan@tootallnate.net)
  
sippykup (nobody@nowhere.com)

## Contact

Nathan Rajlich (nathan@tootallnate.net)

## Download

You can download this project in either
zip or
tar formats.

You can also clone the project with Git
by running:

```
$ git clone git://github.com/TooTallNate/Java-WebSocket
```

get the source code on GitHub : TooTallNate/Java-WebSocket
