# Supplementary material for: Rigbox: An Open-Source Toolbox for Probing Neurons and Behavior
Source: eNeuro. 2020 Jul 9;7(4):ENEURO.0406-19.2020. doi: 10.1523/ENEURO.0406-19.2020 (PMC7363478; doi:10.1523/ENEURO.0406-19.2020)
Supplement: Extended Data 1 — We recommend looking at and downloading the code directly from the github repository, at https://github.com/cortex-lab/Rigbox. Download Extended Data 1, ZIP file. [file enu-eN-OTM-0406-19-s02.zip › Rigbox-master-2020-05-18/Rigbox-master/docs/html/AlyxMatlabPrimer.html]

Introduction 

# Introduction

Alyx is a light-weight meta-database for colony management and experimental logging. Its purpose is to facilitate the recording and sharing of experimental data within the neuroscience community. It is currently adopted by the International Brain Lab (IBL).

## Contents

- Example dataset creation & file registration on Alyx
- Logging in/out
- Using endpoints to GET data
- Posting data
- Datasets
- Advance session search
- Getting file paths
- Saving files
- File registration
- Alyx queue
- Debugging with http.jsonPost
- Using Alyx with Rigbox
- Sending Alyx around
- Headless Alyx
- MySQL queries
- Etc.

## Example dataset creation & file registration on Alyx

The simplst way to interface with Alyx via MATLAB is through the RESTful interface. The Alyx class (found in Rigbox/alyx-matlab/@Alyx) creates an instance of an Alyx object that can be passed around rigs and used to get and post data from/to the database. NB: Alyx is actually a value class, not a handle one. More on that later.

```
doc Alyx
```

## Logging in/out

To instantiate an instance of Alyx, call the constructor like so:

```
ai = Alyx;

% When called with no arguments, a login window is automatically displayed.
% To instantiate the object without immediately logging in, call it with
% the first two arguments empty.  This is useful when you want to set a
% different database URL from the default before logging in.  NB: The
% inputs are the Username and Token.
ai = Alyx('','');

% The default database url is set in dat.paths, a member of the dependent
% Rigbox +dat package.
base = getOr(dat.paths, 'databaseURL')

% For playing around you may use the following credentials:
baseURL = 'https://test.alyx.internationalbrainlab.org';
user = 'test_user';
pwd = 'TapetesBloc18';

% To log in use the login method.  Upon success, this sets the Token
% property with a token from Alyx.  To determine whether you're logged in,
% use the IsLoggedIn property. After logging in the object automatically
% flushes any posts in the queue (more later). NB: Alyx is not a handle
% class, so make sure you assign the output to itself.
ai.IsLoggedIn % false
ai = ai.login;

ai.IsLoggedIn % true
ai = ai.logout;

ai.IsLoggedIn % false
```

## Using endpoints to GET data

A list of availiable endpoints can be found in the database documentation Most of these endpoints already have methods associated with them. For instance the listSubjects method returns a list of subjects from the subjects endpoint:

```
ai = ai.login;
subjects = ai.listSubjects();

% You can use the getData method to retrieve data directly from the
% specified endpoint.  For instance to retrieve subject data directly:
subjects = ai.getData('subjects'); % NB: This is called by listSubjects

% getData uses the makeEndpoint method to create the full URL using the
% BaseURL property.  To interact with a different database instead, either
% provide the full URL or change the BaseURL property accordingly.  NB: You
% may need to refresh your token by logging out and back in.
ai.BaseURL = 'https://test.dev.alyx.internationalbrainlab.org';
users = ai.getData('users');

% The data are returned as a struct.  The second output argument is the
% server status code.  For a full list of status codes and their meanings:
doc matlab.net.http.StatusCode
[users, status] = ai.getData('users') % 200 = OK!

% To use any URL queries, just add them the endpoint string in the standard
% URL format:
sessions = ai.getData('sessions?type=Experiment&subject=ZM_335')

% For more info:
doc webread

% The query options are set on the server side.  You can find which options
% are availible for each endpoint by vising the alyx documentation (see
% above).  This information is also availiable on the DJANGO API page but
% be warned it is slow to load as you are running a GET within the browser:

% Example: https://test.alyx.internationalbrainlab.org/sessions
% HTTP 200 OK % HTTP version
% Allow: GET, PUT, PATCH, DELETE, HEAD, OPTIONS % Methods allowed
% Content-Type: application/json % Post using Alyx.jsonPost
% Vary: Accept
```

## Posting data

POST requests (those that create new records on Alyx) can be made with the postData method. Upon success postData returns the created record. For creating a new experiment, use the newExp method. This is the same as the dat.newExp function, but with the addition of creating session records on Alyx.

Alyx.newExp first creates a base session if one doesn't exist. This is a session corresponding to a particular day (the yyyy-mm-dd part of an experiment reference, see dat.parseExpRef). On Alyx, base sessions have the type 'Base'. It then creates a new subsession. Subsessions correspond to the experiment sequence number of an expRef. On the database, these sessions are identified by the type field being 'Experiment'. For example, you may have three subsessions related to a base session, meaning on that particular day, you ran three expeiments, perhaps training the mouse on a rig, then later doing RF mapping, then doing some imaging with behaviour. Alyx supports further nesting of sessions, however Alyx.newExp currently doesn't support this.

The newExp method returns the new subsession URL which may then be saved in SessionURL property of the object for further posts. This URL is in itself an endpoint for ameding (with PATCH) session information (or even creating a nested subsession).

Before you can create a session a few things must be set up: # A subject folder must first be created in your main experiment repository:

```
subject = 'ZM_335'; % Subject for this test
mkdir(fullfile(dat.reposPath('main', 'master'), subject)); % Subject folder
% # The location of your main repository must be added to Alyx through the
% admin interface.
web([ai.BaseURL '/admin/datarepository/']) % Not accessible on test alyx
% The hostname field must match the root of the file path:
rmEmpty({ai.getData('data-repository').hostname}')

% Let's create a session for our subject:
[expRef, expSeq, url] = ai.newExp('ZM_335');
ai.SessionURL = url; % Now holds the current (most recent) subsession URL.

% Let's update the session narrative:
comments = 'This is a dummy session';
narrative = ai.updateNarrative(comments, ai.SessionURL);

% Let's manually create a nested subsession
d = struct;
d.subject = 'test';
d.procedures = {'Behavior training/tasks'};
d.narrative = 'sub-sub-session';
d.start_time = ai.datestr(now); % date in Alyx format
d.type = 'Experiment'; % Sub-sessions have the type 'Experiment'
d.parent_session = ai.SessionURL;
d.number = 1; % First session of today

[subsession, statusCode] = ai.postData('sessions', d)

% With this method you can also make PUT and PATCH requests to amending a
% record.  NB: Only for those endpoints with these options availible.
% Let's update the session's end time:
d = struct('end_time', ai.datestr(now)); % Subject is a required field for PUT
ai.postData(ai.SessionURL, d, 'patch') % Update the record with PATCH

% The postData method uses the jsonPost method, which in turn uses the
% built in MATLAB function webwrite.  More info:
doc Alyx.jsonPost
doc webwrite

clear d subsession statusCode url comments expSeq
```

## Datasets

One of the purposes of Alyx is to make sharing data to a wider group simple and accessible. In order to do this effectively there are a few things imposed on the user in terms of how data and meta-data are stored:

```
% 1. Files must be stored in a directory by mouse name, then experiment
% date (corresponding to the base session on Alyx), then
% experiment/sequence number (corresponding to subsessions on Alyx). Alyx
% infers information about the files based on this directory structure.

% 2. Where the files are saved must correspond to a data repository that
% has been created on Alyx.  One such example is zubjects.  For the IBL
% collaboration, the data repository records are used by Globus to map the
% drive in order to copy selected files to The Flatiron Institute
% (availiable to other labs - only subjects under the project 'IBL' are
% copied).  More information can be found in the
% <https://alyx.readthedocs.io/en/latest/models.html#data alyx database
% documentation>:
web('https://alyx.readthedocs.io/en/latest/models.html#data')

% 3. The files must have a corresponding dataset type.  This is a record on
% Alyx that includes a human readable description of what the data are and
% a filename pattern for identifying the file.  It is suggested that the
% filename pattern correspond either to the ALF standard, or the Rigbox
% standard of expRef_type (more on this later).  Datasets, like sessions,
% are hierachical, for instance you could have a parent dataset type (with
% no corresponding files) that is called 'twoPhoton', with the ALF pattern
% '*_2P*.*'.  Child dataset types could include ROI files (e.g.
% '*_2P_ROI.*') and raw frames (e.g. '*_2P_ROI.*'). More info:
% https://alyx.internationalbrainlab.net/admin/data/datasettype/
web('https://github.com/cortex-lab/ALF')
% The list of current and proposed ALFs may be found
% <https://docs.google.com/spreadsheets/d/1DqyQ-Ho4eObR0B4nZMQz397TAUReaef-9dRWKwIa3JM/edit#gid=0
% here>
%

% 4. The files must have a valid format, defined on Alyx by the data format
% records.  These records include a description of the file format, the
% filename pattern (e.g. '*.mat' or '*.*.npy' for ALF files), and the
% function name for loading the file in MATLAB and Python.  More info:
% https://alyx.internationalbrainlab.net/admin/data/dataformat/
```

## Advance session search

Sessions can be queried in an sophisticated way with the getSessions method:

```
help ai.getSessions

% Session eids can be used as input, as well as experiment reference
% strings.  A single value can be passed in or a whole list:
sessions = ai.getSessions('cf264653-2deb-44cb-aa84-89b82507028a') % eid
sessions = ai.getSessions('2018-07-13_1_flowers') % expRef

% It can be used to convert session eids to expRefs:
refs = ["2018-07-13_1_flowers" "2019-08-16_2_ZM_335"];
[~, eids] = ai.getSessions(refs)

% Sessions can be filtered based on subject, date, or associated dataset
% types:
sessions = ai.getSessions('cf264653-2deb-44cb-aa84-89b82507028a', ...
  'subject', {'flowers', 'ZM_307'})
sessions = ai.getSessions('lab', 'cortexlab', ...
  'date_range', datenum([2018 8 28 ; 2018 8 31]))
sessions = ai.getSessions('date', now)
sessions = ai.getSessions('data', {'clusters.probes', 'eye.blink'})
```

## Getting file paths

The +dat package is primarily used to retrieve data from a rig's repositories, however this information can also be retrieved from the database, as well as any additional remote locations if Globus is set up:

```
help Alyx.getFile

% Get paths associated with a particular session's dataset:
eid = 'c41dd877-d511-42cb-90a3-01bb19297117'; % dataset uuid
[fullPath, exists] = ai.getFile(eid)
% If exist == false it means the file was registered to that location but
% has not yet been copied there.

% We can retrieve only 'remote' files, meaning those in repositories with a
% data_url field:
[fullPath, exists] = ai.getFile(eid, 'dataset', true) % remoteOnly = true

% Finally we can also retrieve paths for inividual file records:
eids = ["00c3df4f-99ab-4cc0-b305-b508bcfb07ab",...
      "0b747a70-1309-4f84-98f6-5f3aa9815b4c"] % File record uuids
fullPath = ai.getFile(eids, 'file', true) % remoteOnly = true

% Note: Inputs may be cellstr, char or string.  The first output will be
% either a char or cellstr.
```

## Saving files

Saving files in a standard way can be easily achieved by using dat.expFilePath to return the path and file name for your data. The main data repository is defined in dat.paths, the folder tree by dat.canstructExpRef (used by dat.newExp), and the file name is constructed from the expRef + '\_filetype'. The list of file types are found here:

```
opentoline(which('dat.expFilePath'),45,1)
doc dat % further info
open(fullfile(getOr(dat.paths, 'rigbox'), 'docs', 'using_dat_package.m'))

% For example, let's create a new experiment, then save the associated
% eye tracking data:
expRef = ai.newExp(subject); % Create new experiment ref and Alyx (sub)session
fullpath = dat.expFilePath(expRef, 'eyetracking', 'master')
% Without specifying the which repo location (i.e. 'master' or 'local'),
% you get both paths returned.
fullpaths = dat.expFilePath(expRef, 'eyetracking')

clear fullpaths
```

## File registration

In order to successfully register a file to Alyx, you must make sure the dataset type, repository and format records are created on the database first. To register a file, you can use the registerFile method:

```
[datasets, filerecords] = ai.registerFile(fullpath) % Register a specific file
% The returned dataset records should contain the associated session URL
% and the dataset type, as well as a list of filerecords.  The filerecords
% contain the path to the files and what repository they're found on.

% You can also input a cell array of paths, including whole directories:
expPath = dat.expPath(expRef, 'main', 'master') % Returns the directory
[datasets, filerecords] = ai.registerFile(expPath)
% Returns the datasets are filerecords of all successfully registered files
% in the provided directory.  You can register the same file multiple
% times.  If a filerecord already exists, Alyx simply returns the original.
```

## Alyx queue

All posts to Alyx are first saved as a JSON file in a location specified by the objects QueueDir property. This means that when a post fails for reasons other than user error, the posts remain in the queue until further notice. Each time a user 'logs in', the queue is flushed, meaning that all saved posts are re-submitted to Alyx. You can manually flush the queue by called in the flushQueue method:

```
ai.flushQueue
```

## Debugging with http.jsonPost

Unfortunately, the MATLAB built in http interface functions are limited in terms of debugging, as they don't directly return the server's responses upon failure. Status codes must be extracted from the error message bodies, and the full reponse of the server is usually not returned. In order to debug your Alyx posts, you can use the missing http package's jsonPost function instead. See line 47 of Alyx.flushQueue:

```
opentoline(which('flushQueue'),47,1)
web('https://github.com/psexton/missing-http/releases') % To download toolbox
```

## Using Alyx with Rigbox

Alys can be used with Rigbox. In this way subject information such as weight can be posted to Alyx through MC or the AlyxPanel GUI, and files and reward volumes can be posted automatically at the end of the session.

To activate Alyx simply set the databaseURL field in dat.paths to a non-empty string (should be a valid database url).

```
getOr(dat.paths, 'databaseURL')

% Once active you will be able to use the standalone AlyxPanel and the one
% in MC:
doc eui.AlyxPanel

% Let's create a new panel:
eui.AlyxPanel;

% You can log in through this panel, select subjects and view session
% histories.  You can also post weight and water information to alyx here.
% If you have a digital weigh scale installed, you can directly record the
% weights by clicking the 'record' button (otherwise appears as 'manual
% weight').  More info on setting this up can be found here:
hw_setup = fullfile(getOr(dat.paths, 'rigbox'), 'docs', 'setup', 'hardware_config.m');
opentoline(hw_setup, 497, 1)
```

## Sending Alyx around

An Alyx instance can be sent between rigs in one of two ways: 1. Via Java Websockets using the io.WSJCommunicator.server/client object. This object uses the hlp\_serialize/deserialize functions to send the object. 2. Via UDP using Rigbox services objects (using udp and pnet functions). The Alyx.parseAlyxInstance method can be used to convert the object into a JSON string and back again. Both methods ultimately use saveobj and loadobj methods to convert the Alyx object. This isn't really important to know. More info on services:

```
doc Alyx.parseAlyxInstance % Used by srv.BasicUDPService and tl.mpepListener
doc io.WSJCommunicator.server % Used by expServer to communicate with mc
doc srv.StimulusControl % Used by mc to communicate with expServer
open(fullfile(getOr(dat.paths, 'rigbox'), 'docs', 'setup', 'using_services.m'))
```

## Headless Alyx

Before sending an instance of Alyx to a stimulus computer, consider setting the Headless property to 'true'. This means that the object will not spawn any user prompts, and will supress some errors. This can also be set when the database become unreachable. All posts are still saved in the queue for when a user logs in on that computer. NB: Even when headless the Alyx object may throw errors. On recording computers, always put GET/POST related methods in a try-catch, or only attempt such things at the end of the experiment

```
ai.Headless = true;
ai = ai.logout;
ai = ai.login; % Dialog surpressed
ai.registerFile(expPath); % Dialog surpressed
```

## MySQL queries

One can also interact with Alyx through connection to the underlying MySQL database. This currently isn't really supported by the alyx-matlab package and isn't encouraged. More information:

```
alyxPath = fileparts(which('Alyx'));
open(fullfile(alyxPath, '..', 'docs', 'sql', 'openAlyxSQL.m'))
open(fullfile(alyxPath, '..', 'docs', 'sql', 'expFilePath.m'))
```

## Etc.

Author: Miles Wells

v1.1.0

```
%#ok<*NASGU,*ASGLU,*NOPTS>
```

Published with MATLAB® R2018b
