# Supplementary material for: Rigbox: An Open-Source Toolbox for Probing Neurons and Behavior
Source: eNeuro. 2020 Jul 9;7(4):ENEURO.0406-19.2020. doi: 10.1523/ENEURO.0406-19.2020 (PMC7363478; doi:10.1523/ENEURO.0406-19.2020)
Supplement: Extended Data 1 — We recommend looking at and downloading the code directly from the github repository, at https://github.com/cortex-lab/Rigbox. Download Extended Data 1, ZIP file. [file enu-eN-OTM-0406-19-s02.zip › Rigbox-master-2020-05-18/Rigbox-master/docs/html/Burgess_hardware_setup.html]

 

Instructions for steering wheel setup: hardware

Version 1.0.0, 14 February 2020

Lauren E Wool\*, Miles J Wells\*\*, Hamish Forrest, and Matteo Carandini  
\*l.wool@ucl.ac.uk, \*\*miles.wells@ucl.ac.uk   
Cortical Processing Laboratory, University College London

# Table of contents

Introduction

Master computer

Stimulus computer

Installing the video card

Installing the DAQ device

Frame and components

Assembling the frame

Preparing the screens

Attaching the Fresnel lenses

Installing the screens

Configuring the screens

Assembling the mouse holder

Adding the camera and speaker

Assembling the water-reward system

Connecting the water-reward system

Connecting the rotary encoder

Connecting the photodiode

Comments and suggestions

Appendix: Components list

# Introduction

This document gives instructions on how to build a basic version of the steering wheel setup to probe mouse behavior, introduced by Burgess et al. (Cell Reports, 2017). The goal is to make it easy for other laboratories, including those that make the International Brain Laboratory, to replicate the task and extend it in various directions. To this end, these instructions rely entirely on materials that can be bought off the shelf, or ordered online based on 3-D drawings.

In this steering wheel setup, we place a steering wheel under the front paws of a head-fixed mouse, and we couple the wheel’s rotation to the horizontal position of a visual stimulus on the screens. Turning the wheel left or right moves the stimulus left or right. The mouse is then trained to decide whether a stimulus appears on its left or its right. Using the wheel, the mouse indicates its choice by moving the stimulus to the center. A correct decision is rewarded with a drop of water and short intertrial interval, while an incorrect decision is penalized with a longer timeout and auditory noise.  

We use this setup throughout our laboratory, and deploy it in training rigs and experimental rigs. Training rigs are used to train head-fixed mice on the steering-wheel task and acquire behavioral data. Experimental rigs have additional apparatus to collect electrophysiological and imaging data, measure eye movements and licking activity, provide optogenetic perturbations, and so on.

Up until recently, constructing these setups required a machine shop that could provide custom-made components. However, for the purposes of spreading this setup to other laboratories, we here describe a new version that does not require a machine shop: all components can be ordered online or 3D-printed.

NB: An improved frame design can be found on the IBL Website.  The improved frame and its components (mouse holder, etc.) may still be used with Rigbox.

The behavioral rig. A basic behavioral setup with three iPad screens (with Fresnel lenses), a mouse holder (with steering wheel), a water-delivery system, a head-fixation assembly, and a photodiode.

The main components of the behavioral setup are a mouse holder with steering wheel encoder, a water-delivery system, and three iPad screens for stimulus presentation. These and other components are  held together by a “frame”. A “stimulus computer” controls all this apparatus. Additionally, a “master computer” controls the stimulus computer.

A 3D view of the rig is available here, and a detailed list of the components can be found in this spreadsheet. Note that this list was assembled in the UK; though we have aimed to use international vendors, you may need to pick different ones depending on your location (please let us know if you encounter difficulties). Place the orders as soon as possible, as some items will take time to arrive. Also, start planning how you will 3D-print the mouse holder.

Configuring the “stimulus computer” is more involved, and so it helps to have it in place first. This way you can use it to test other components as they arrive. The “master computer” can be configured once you are ready to run software.

# Master computer

The “master computer” controls the stimulus computer and manages the experimental session, and monitors the mouse with a camera. It runs Windows 10. The computer should be connected to the network, ideally through a high-speed connection (not WiFi). Instructions for installing and managing experimental software for this computer can be found in the document "Software instructions for steering wheel setup." If you are not using our software, you do not necessarily need this computer.

# Stimulus computer

The “stimulus computer” controls stimulus display and water delivery, and acquires the mouse’s movements on the steering wheel and the output of a photodiode to synchronize stimulus output with other measurements. It runs Windows 10. The computer should be connected to the network, ideally through a high-speed connection (not WiFi). Instructions for installing and managing experimental software for this computer can be found in the document "Software instructions for steering wheel setup."

## Installing the video card

The required video card is the NVIDIA NVS 510. This card drives four monitors: a main display monitor and the three iPad screens. This is a typical installation that would apply to any card for a Windows machine; if you are not familiar with the process, detailed instructions are below.

1. Ensure your main monitor is connected to the onboard graphics card
2. If you are removing a currently graphics card, open 'Device Manager' and check the 'Display adapters' drill-down menu for the current graphics card
3. Double-click the graphics device: under the 'Driver' tab, select 'Uninstall' to remove any drivers associated with the current card.
4. After powering down and unplugging the computer, open the case and install the NVIDIA NVS 510 in the PCI slot. If you are replacing an existing card, remove it and replace it with the NVS 510.
5. Power up the computer and open 'Device Manager' and check that the 'Display adapters' drill-down menu shows a new object: 'Microsoft Basic Display Adapter,' or similar
6. Via your browser, find and download, then install, an appropriate driver for your new card. As of October 2017, the current driver for NVIDIA NVS 510 is the Quadro Desktop/Notebook Driver Release 387, Version R387 U1 (387.95).
7. Back in Device Manager, check that 'Microsoft Basic Display Adapter' has changed to the name of your GPU ('NVIDIA NVS 510').
8. Restart the computer for changes to take effect
9. Migrate the main display monitor to an NVS graphics card port using a suitable mini-DisplayPort cable, and confirm that you have visual output

## Installing the DAQ device

To acquire data from the steering wheel and from the photodiode and to deliver water rewards to the mouse, we use a DAQ (data acquisition) device, the NI-DAQ 6211.

1. Plug in the device to a powered USB port on the stimulus computer
2. Visit the Drivers page on the National Instruments website and select ‘NI DAQmx’ from the ‘Popular Drivers and Updates’ list, then download the latest version of the device driver (17.1.1, as of October 2017).
3. Once installation is complete, restart the computer.
4. Launch ‘NI Device Monitor’ from the System Tray. The program should recognize that the NI-DAQ 6211 is connected, and a green light on the DAQ should flash

# Frame and components

The setup itself comprises three iPad screens installed on posts, a mouse holder with a steering wheel encoder and head-fixation assembly, and a water-delivery system. Here we describe the assembly of the frame required to hold these and other components together.

Rig footprint diagram. The steering-wheel setup is contained entirely within a frame made of Thorlabs components, all seated on a 300mm x 300mm aluminium breadboard (MB3030/M). Components can be secured to the breadboard using 16mm M6 cap screws. The screen/Fresnel assemblies are seated between two grooved screws on clamps (one bottom, one top) that are attached to 300mm posts. The mouse itself is seated on a 3D-printed holder (not shown) that attaches to a magnetic plate in the center of the frame. In order for the Fresnel lenses to collimate light properly, the mouse’s head position must be equidistant from the three screens (indicated by the yellow ‘x’). This requirement is fulfilled with proper attachment of the holder to the magnetic plate (described below).

## Assembling the frame

The frame is composed chiefly of Thorlabs components.

1. Assemble the three screen post mounts, by attaching each post holder (PH50/M) to a mounting base (BA2T2/M), then attach them to the breadboard (MB3030/M) as shown in the footprint diagram
2. Insert and secure 1 TR300/M post into each of the mounts
3. Wrap the thread of six PCMV/M V-groove screws 2-3 times with a ~5mm-wide piece of thread-seal tape. (The tape enables fine positioning and better tightening)
4. For each of six PMTR/M clamp arms, screw 1 PCMV/M into the first M6 tap (i.e., closest to the clamp) until the groove is perpendicular to the clamp arm. Rotate just until the screw is tight, and try not to reverse as this ruins the grip
5. On each post, firmly secure 1 PMTR/M clamp directly above the PH50/M, with the PCMV/M V-groove screw facing up. Each clamp arm should be perpendicular to its BA2T2/M plate and facing the center of the breadboard
6. On each post, attach 1 PMTR/M clamp ~20 cm above the lower clamp, facing the same direction, with the PCMV/M V-groove screw facing down. Tighten just to secure to the posts, as these will be readjusted

## Preparing the screens

To protect the screens from damage, keep the plastic film on until you are ready to attach the Fresnel lenses

1. On a soft surface, turn the iPad over so that the back is facing up and the input cable is running along the top
2. Check that the cable input on the driver board has its locking mechanism (black plastic bar) pressed up
3. Carefully connect a driver board to the input cable. There will be ~1mm of gold contacts left visible. DO NOT FORCE THE CABLE.
4. Attach the driver to the back of the screen with some lab tape, ensuring the cable is not bending or shearing
5. Check that each driver board switch is set to 'OFF'

## Attaching the Fresnel lenses

The purpose of the Fresnel lenses is to ensure that the intensity is homogeneous across the screen when viewed from a specific location (marked by a yellow ‘x’ in the rig footprint diagram above). To avoid pixel aliasing, the iPad screen and Fresnel lens must be separated by ~1 mm, using silicone pads. Various brands of window film may have different application instructions, so you may need to adapt steps 2–13 below.

1. Ensure the smooth side of each Fresnel lens is clean and dry, with no fingerprints (use an air duster if needed to remove debris)
2. Lay the lens on a bed of paper towels, smooth-side up with the short edge nearest
3. Cut a piece of window film that is slightly larger than the fresnel lens (~2 cm border)
4. Carefully peel back the adhesive and lay the window film next to the lens, adhesive-side up with the short edge nearest
5. Generously spray both the lens and the film with soap/water solution
6. Lift the window film from a short edge, letting it hang orthogonally to the surface of the fresnel lens
7. Slowly lower the film to the lens surface, allowing the bottommost short edges to begin contacting each other
8. Continue lowering to make contact with the lens, meanwhile using the squeegee to press out extra bubbles and solution to the sides
9. Continue working away from the bottom edge, lowering the film and using the squeegee to remove bubbles and solution
10. Lifting the lens/film, replace the wet paper towels with some new dry ones
11. Carefully flip the lens/film over so that the ridged side is now facing up
12. Trim the film edges with a razor or scalpel blade
13. Flip the lens over to remove any remaining bubbles with a squeegee, and further trim edges with a blade as needed. You may find there are white patches on the lens after applying the film. These will usually disappear after a few hours
14. Carefully remove the plastic protective film from the iPad screen and lay it face up
15. Cut four small pieces of silicone pad (~3mm x ~20mm) from the silicone rubber sheet, and apply them to each corner of the screen, atop the screen bezel, with the long dimension of the pad running along the long edges of the screen. The pads should not touch the screen display
16. Center the fresnel lens over the screen, with the rough side facing in, adjusting the silicone pads so that they lie between the screen bezel and the edge of the lens
17. Using four binder clips, clip the screen/Fresnel assembly together at each corner, centered over the pads (two clips on the top and two clips on the bottom). Ensure that the clips do not intrude on the viewable area of the screen, but only grip along the bezel where the pads are located.

## Installing the screens

If you haven't already done so, ensure that the main monitor of the Stimulus computer is connected to the NVIDIA video card via a mini-DisplayPort cable, at port 4.

1. For each screen, center the bottom edge into the V-groove screw of the lower PMTR/M clamp. The output cable and driver board should be at the top of the screen on the back
2. While holding the screen assembly in place, lower the upper PMTR/M clamp down the post until its V-groove screw holds the top edge of the screen assembly, and then firmly secure its position with the thumbscrew
3. After each screen assembly has been attached to a post, make minor adjustments at each BA2T2/M plate to ensure that the screens are perpendicular to each other and that the edges of the fresnel lenses are flush
4. Once the screens are firmly in place and all connection points are secured tightly to the breadboard, plug in the AC power cable to each.
5. Connect each screen to the remaining NVS ports via the miniDP-to-DP display cables. Facing the screens, the leftmost screen should connect to port 1, the center to port 2, and the rightmost to port 3. The main monitor should connect to port 4.
6. Turn the driver board switches to 'ON'
7. Check that visual output appears onscreen. It is possible that the screens are out of order or the 'main display' option defaults away from your main monitor. This can be reconfigured, below.

## Configuring the screens

The three iPad screens must be designated as a single viewable output, separate from the main monitor. This is accomplished using the NVIDIA Control Panel.

1. Launch the NVIDIA Control Panel
2. In the left panel, select ‘Select a task…’ > ‘Workstation’ > ‘Set up Mosaic’
3. Click ‘Create new configuration’
4. Under the ‘1. Select topology’ tab, select ‘Number of displays’ as 3 and choose a 1 x 3 topology with landscape orientation.
5. Check the box ‘I am using recommended connections for the selected topology’ and then click ‘Enable Mosaic’
6. Under the ‘2. Select displays’ tab, check the three iPad screens. These should be numbered 1 (0,0), 2 (0,1), and 3 (0,2). If they are not, rearrange the connections at the ports until this is satisfied.
7. Assign ‘Resolution per display’ to 1280 x 1024. If they are not already in the ‘Selected display sources’ box, drag them there and then click ‘Next’
8. Under the ‘3. Arrange displays’ tab, arrange the screen configuration so that 1-3 are arranged left to right. Apply the changes and select ‘yes’ to the dialog box prompting to save. Note: The main monitor will switch off and your working window will move to the 3-screen arrangement. Click ‘Finish’ ( skip overlap and bezel correction).
9. Return to the main control panel, this time choosing ‘Select a task…’ > ‘Displays’ > ‘Set up multiple displays’
10. Of the available displays, check the remaining box corresponding to the main monitor. In addition to the mosaic screens (labeled ‘3’), it will appear in the configuration window next to the 3-screen mosaic (display ‘3’) as display ‘4.’
11. Rearrange the screens in the preferred configuration, then right-click screen 4 and choose ‘make primary’. Apply and accept changes. The main monitor should now reactivate.
12. Launch Windows 10 > Settings > Display
13. For each of the displays, ensure that ‘Change the size of text, apps and other items’ is set to 100%

## Assembling the mouse holder

The mouse holder comprises seating for the mouse, a steering wheel/encoder assembly, an articulated arm for delivering water rewards, and a head-fixation assembly.

The mouse holder assembly. Left: The mouse holder, seen from the front, shows the center bore that will accommodate the wheel assembly, as well as key attachment points to the KBM1/M breadboard. Center: The front of the holder shows the attachment point for the Fisso arm. Right: The back of holder shows the attachment point for the head-fixation assembly.

1. 3D-print the (a) mouse holder, (b) mouse cover, and (c) wheel coupler designs. This can be done in-house or can be outsourced to third parties. Use PLA plastic for best results. If desired, the mouse cover can be printed in clear PLA so as to better observe mouse body movements
2. Connect the wheel coupler to the shaft of the rotary encoder. It should attach securely with a just strong push
3. Feed the rotary encoder through the large center bore on the front plate of the mouse holder, so that the coupler is facing outward and the encoder body is housed on the underside of the mouse holder. The encoder cable should be oriented downward
4. Feed three 5-mm M3 cap screws through the small bores on the front plate of the mouse holder, and then screw them into the threaded holes on the face of the encoder
5. To the top plate of the two-piece KBM1/M kinematic breadboard, attach the larger Fisso articulated arm to the front center hole (row 1, hole 3) of the KBM1/M, and secure tightly with an adjustable wrench
6. Connect the 3D-printed mouse holder to the KBM1/M top plate using four 45-mm M6 cap screws, as shown in the diagram above. The two front screws will be in row 1 (holes 1 and 7) and the two back screws will be in row 3 (holes 1 and 7). Ensure that the magnetic hex switch is oriented to one side for accessibility
7. Once the mouse holder is in position, assemble the LEGO wheel and hub, then gently secure it to the front cross of the coupler. There should be 1-2 mm of space between the wheel and the front face of the mouse holder
8. Rotate the KBM1/M hex screw to release the bottom plate from the mouse holder assembly
9. With a 20-mm M6 set screw, attach the TR200/M post to the KBM1/M top plate at row 6, hole 7
10. Attach the RA90/M right-angle clamp 2 mm below the top of the TR200/M post, with the second post bore pointed toward the back of the KBM1/M top plate.
11. Attach the RA180/M end clamp to the base of the TR50/M post, then feed the assembly through the open bore of the RA90/M, so that the RA180/M is centered over the mouse holder and the RA180/M bore is parallel to the KBM1/M top plate. Secure the RA90/M thumb screw
12. Attach the headplate holder to the base of the TR100/M post with a 16-mm M6 cap screw
13. Feed the holder/post assembly through the bore of the RA180/M until the headplate holder screw points are centered over the steering wheel. Secure the RA180/M thumb screw
14. Insert the two M3 thumb screws top-down into the headplate holder. These are to secure the animal’s headplate
15. Screw the KBM1/M bottom plate to the MB3030/M breadboard at the positions shown on the footprint diagram, then attach the holder assembly to the plate via the magnetic switch. (To avoid damage or injury, always make sure the magnetic switch is OFF before attempting to add or remove the top plate.)
16. Cut a 65mm x 35mm rectangle of silicone rubber sheeting, and use it to line the bore where the mouse will be seated. This adds a bit of padding for comfort, and can be washed with soap & warm water between sessions.

## Adding the camera and speaker

A camera is essential for monitoring the mouse’s behavior during the task, and a speaker is necessary to transmit simple task-related sounds, like beeps and white noise.

1. Place the camera atop the left corner of the screens, where the left and center screens meet. If needed, use small pieces of poster putty to keep it stable. This needs to be positioned to view the front of the mouse holder at the top of the wheel, where the mouse’s face will be located during the task
2. Plug the camera into the Master computer
3. The camera can be managed with freeware (such as WebcamViewer,  ManyCam, or the software that comes with the camera), which should be installed on the Master computer. This runs independently of any experimental software.
4. Plug the USB speaker into the Stimulus computer, and place the speaker on one corner of the MB3030/M breadboard, facing the mouse holder

## Assembling the water-reward system

Making a spout guide. Use a length of 16AWG copper wire to extend the reach of the Fisso arm and enable water delivery to the mouse.

1. To make a spout guide, first cut an 8-cm length of copper wire. Using long-nose pliers, wrap the end into a 2-mm-diameter loop, then bend it to a right angle
2. Wrap the other end of the wire around the free M6 screw shaft of the Fisso arm, and secure with an M6 hex nut
3. Bend the spout guide so that it extends the length of the Fisso arm toward the top of the steering wheel
4. Position the water valve on one corner of the MB3030/M breadboard.
5. On each end of the tubing already in the pinch valve, connect the larger end of a 3/32" x 1/16 barbed reducing connector
6. Cut 50 cm of 1/32" ID x 3/32" OD tubing, and attach one end to one of the barbed connectors
7. Feed the tubing along the back of the Fisso arm and through the 2-mm loop, and adjust the tubing so ~5 mm extends. Gently secure the tubing to the Fisso with a piece of laboratory tape
8. Adjust the Fisso arm and spout guide so that the tube tip is centered in the bore of the mouse holder. Make sure the Fisso does not obstruct free movement of the wheel
9. Additional 1/32" ID x 3/32" OD tubing will connect to the other barbed connector. This length should be cut in order to reach a nearby water reservoir (use a graduated cylinder or other vessel with milliliter precision)

## Connecting the water-reward system

Before troubleshooting, always power off the valve system by unplugging the power supply directly.

1. Cut two lengths each of red and black hookup wire. One red/black pair will connect the power supply and the other the DAQ board, so cut the lengths accordingly.
2. Strip 5mm of insulation from both ends of each wire
3. Connect the DC cable mount to the red/black wire pair that will supply power. Insert the red wire into the ‘+’ terminal and the black wire into the ‘–’ terminal. Secure the wires in the terminals by using a screwdriver to tighten the set screws. Ensure that no exposed wire is outside the mount housing.
4. Insert the CoolDrive valve controller driver into the prototyping mini-breadboard, so that its pins span multiple rows, not columns (i.e., the driver should run parallel to the center dividing well on the breadboard). The orientation of the driver is reversible, and pin information is twinned at each end (i.e., position 1 = position 9; position 2 = position 8, etc.). However, there is a LED trigger indicator on the driver, so consider its visibility when installing.
5. Connect the red (+) and black (GND) power-supply wires at position 1 and 2 on the breadboard, respectively.
6. Connect the two wires of the valve at position 3 and 5. Note that these leads are interchangeable.
7. Connect the red (+) and black (GND) DAQ wires at positions 4 and 8, respectively.
8. Using a piece of electrical tape, tape over all the breadboard connections to avoid short-circuiting or injury.
9. On the back of the power-supply plug, adjust the voltage of the power supply to the 12V by rotating the switch position with a flathead screwdriver
10. Connect the 5.5mm DC (male) plug to the two-pin output of the power-supply lead. Ensure the correct polarity by matching up the ‘+’ symbols between the plug and the lead
11. Connect the red DAQ (+) wire at position 4 to the NI DAQ 6211 at port ‘AO0.’ Insert the wire into the port, and using a 2-mm flathead screwdriver, tighten the port’s set screw until the wire is secure
12. Connect the black DAQ (GND) wire at position 8 to the NI DAQ 6211 at port ‘AOGND.’ Insert the wire into the port, and using a 2-mm flathead screwdriver, tighten the port’s set screw until the wire is secure
13. Connect the male and female DC connectors for the power supply
14. Power the breadboard by plugging in the power supply to the wall socket
15. To test for valve function, launch ‘NI Device Monitor’ from the System Tray on the stimulus computer. The program should recognize the NI-6211, and a green light on the DAQ should flash
16. Select the ‘NI USB-6211:Dev1’ icon, and then select ‘Test this device’
17. In the Test Panels dialog box, migrate to the ‘Analog Output’ tab, then select ‘Mode: Voltage DC’
18. Alternate the ‘Output Value’ between (+) and (-) 5 V in the right pane, clicking the Update button each time.
19. The state of the valve (open or closed) will itself alternate with the alternating +/- 5V outputs—this will be evidenced by a small click and illumination of the green LED light on the driver board.

## Connecting the rotary encoder

1. Isolate the gray, green, brown, and white wires in the cable output of the rotary encoder.
2. Connect the wires to the NI DAQ 6211 at positions PFI0 (gray), PFI1 (green), +5V (brown), GND (white) by inserting the exposed wire into the correct DAQ socket and securing it with a 2-mm flathead screwdriver until tight
3. Ensure the other, loose wires don’t short one another by trimming them or insulating each one with electrical tape
4. To test for encoder function, launch ‘NI Device Monitor’ from the System Tray.
5. Select the ‘NI USB-6211:Dev1’ icon, and then select ‘Test this device’
6. In the Test Panels dialog box, migrate to the ‘Counter I/O’ tab, then select ‘Mode: Edge Counting’ and ‘Pulse Terminal: /Dev1/PF10’
7. Click the Start button, then manually spin the wheel on the mouse holder. If the encoder is working properly, the counter value will increase quickly, irrespective of wheel direction.

## Connecting the photodiode

A photodiode is critical for gamma-correcting the luminance output of the screens, and is important for measuring stimulus timing. However, it is not required for starting basic behavioral training and can be added at a later stage.

1. Attach the photodiode horizontally to the TR50/M post via the M4 screw
2. Insert the TR300/M post into the PH50E/M post holder
3. Place the PH50E/M on the breadboard so that the TR300/M post is near the rightmost edge of the right screen
4. Mount the PH50E/M to the breadboard using a CF125 clamping fork and M6 cap screw
5. Use the RA90/M right-angle clamp to connect the TR50/M and TR300/M post so that the photodiode lens is facing the screen
6. Adjust the clamp and post positions so that the photodiode lens is flush with the iPad screen and as far into the top righthand corner of the screen as possible
7. Connect the photodiode’s power supply and a male-male BNC coaxial cable to the photodiode output port.
8. Connect the open end of the coaxial cable to the female BNC socket with test leads
9. Strip 5mm of insulation from both ends of each wire
10. Connect the red BNC (+) wire to the NI DAQ 6211 at port ‘AI1.’ Insert the wire into the port, and using a 2-mm flathead screwdriver, tighten the port’s set screw until the wire is secure
11. Connect the black BNC (GND) wire to the NI DAQ 6211 at port ‘AI9.’ Insert the wire into the port, and using a 2-mm flathead screwdriver, tighten the port’s set screw until the wire is secure
12. Power on the photodiode (there are switches at both the photodiode housing and power supply)
13. To test for photodiode function, launch ‘NI Device Monitor’ from the System Tray on the stimulus computer. The program should recognize the NI-6211, and a green light on the DAQ should flash
14. Select the ‘NI USB-6211:Dev1’ icon, and then select ‘Test this device’
15. In the Test Panels dialog box, migrate to the ‘Analog Input’ tab, then select Channel Name: Dev1/ai1,’ ‘Mode: On Demand,’ and ‘Input Configuration: Differential’
16. Click the ‘Start’ button.
17. Free the photodiode slightly from its position near the screen so the sensor is visible. Without touching the sensor, carefully use your hand or a piece of cardstock to perturb the amount of light reaching it. This will cause the voltage reading to fluctuate, which will be visible on the onscreen graph.

# Comments and suggestions

Now that you have your behavioral setup, you will want to place it in a sound insulation box or other quiet place to train your mice, or somewhere else in your lab, where you can acquire physiological data.

In following these instructions, you certainly will find that parts of this document can be improved. By all means, please make those improvement, by using the “suggest” feature if you are reading this in Google Docs, or by sending an email to Lauren or Miles.

An example of a steering-wheel setup used in a 2-photon imaging rig

# Appendix: Components list

|  |  |  |  |  |  |
| --- | --- | --- | --- | --- | --- |
| Category | QTY | Product | Specifications | Supplier | Notes |
| Stimulus computer | 1 | Computer | Processor: Intel Core i3 7100, 3.9 GHz  Memory: 16 GB (2 x 8 GB) Corsair 2133MHz DDR4 RAM, 1.2 V  Storage: 1TB WD Blue WD10EZEX, 7200rpm, 64MB Cache  OS: Windows 10 64-bit | Scan V10i | This computer controls the rig. For more details, see the document: "Software Instructions for Steering Wheel Setup". Note: When building multiple rigs, EACH rig needs its own Stimulus Computer, but these can all be controlled by one Master Computer |
|  |  |  |  |  |  |
|  |  |  |  |  |  |
|  |  |  |  |  |  |
|  | 1 | Graphics card | NVIDIA NVS 510 | Insight Cat# VCNVS510VGA-PB |  |
|  | 1 | Keyboard & mouse |  |  |  |
|  | 1 | Monitor |  |  |  |
|  | 1 | mini-DP display cable |  |  | Connects the monitor to the video card |
|  | 1 | USB stereo speaker |  | Adafruit 3369 |  |
| Master computer | 1 | Computer | Processor: Intel Core i3 4170, 3.70 GHz  Memory: 4 GB (2 x 2 GB) DDR3/DDR3L  OS: Windows 10 64-bit  Graphics Card: On-board graphics support | HP ProDesk 400 G2.5 (or similar) | This computer controls the Stimulus Computer. For more details, see the document: "Software Instructions for Steering Wheel Setup". Note: When building multiple rigs, EACH rig needs its own Stimulus Computer, but can all be controlled by one Master Computer. If you are not using our software, you do not necessarily need this machine. |
|  |  |  |  |  |  |
|  |  |  |  |  |  |
|  |  |  |  |  |  |
|  |  |  |  |  |  |
|  |  |  |  |  |  |
|  | 1 | Keyboard & mouse |  |  |  |
|  | 1 | Monitor |  |  |  |
|  | 1 | USB camera |  | Logitech C270 |  |
| Stimulus displays | 3 | iPad 3/4 Retina display | LP097QX1 | Adafruit 1751 |  |
|  | 3 | Qualia bare driver board |  | Adafruit 1716 |  |
|  | 3 | Mini DP-to DP cable | DisplayPort 1.2, 2m length | StarTech MDP2DPMM2M |  |
|  | 3 | 9V DC, 1A power supply | 9V DC, 1A, 1 output | RS 720-3726 (UK) / 720-3720 (USA/Japan) / 720-3739 (EU) |  |
|  | 3 | Fresnel lens | Size: 200 x 160 mm  Thickness: 2mm  Material: PMMA  Groove pitch: 0.5mm  Focal length: 220mm | Wuxi Bohai Optics (BHLens.com) BHPA220-2-5 (request the listed specifications) | Cortexlab have a number of these lenses cut to spec; please contact us if you are interested in obtaining some for your build |
|  |  |  |  |  |  |
|  |  |  |  |  |  |
|  | 1 | Roll adhesive window film (50 cm x 5 m) | Smooth surface, frosted-effect, adhesive (not self-adhesive/static) | The Window Film Company "Frostbrite" (or similar) |  |
|  | 12 | Binder clips | 19mm | Staples 831594 |  |
|  | 1 | Roll silicone rubber sheet | 600mm x 600mm x 1.5mm | RS 840-5541 |  |
| Mouse holder | 1 | Incremental rotary encoder | 24mm, push-pull, 5V, 1024 PPR | Kubler 05.2400.1122.1024 |  |
|  | 1 | USB data acquisition (DAQ) I/O device | Interface for rotary encoder and water reward system | National Instruments USB-6211 |  |
|  | 1 | LEGO wheel/axle hub | Wheel 62.4 x 20 with Short Axle Hub, with Black Tire 62.4 x 20 (86652 / 32019) | LEGO parts 86652 & 32019 (www.bricklink.com) |  |
|  | 1 | Mouse holder block | 3D printed: [link] |  |  |
|  | 1 | Mouse holder block cover | 3D printed: [link] |  |  |
|  | 1 | Wheel/encoder coupler | 3D printed: [link] |  |  |
|  | 3 | 5mm M3 screw |  | Thorlabs SH3M5 (or part of HW-KIT5/M) | SH3M5 is a pack of 50, but only 3 total are required |
|  | 4 | 45mm M6 cap screw |  | RS 293-397 (or part of Thorlabs HW-KIT2/M) | RS 293-397 is a pack of 50, but only 4 total are required |
|  | 1 | Post |  | Thorlabs TR200/M |  |
|  | 1 | Post |  | Thorlabs TR50/M |  |
|  | 1 | Post |  | Thorlabs TR100/M |  |
|  | 1 | Right-angle clamp |  | Thorlabs RA90/M |  |
|  | 1 | Right-angle end clamp |  | Thorlabs RA180/M |  |
|  | 1 | 20mm M6 set screw |  | Thorlabs SS6MS20 (or part of HW-KIT2/M) | SS6MS20 is a pack of 25, but only 1 is required |
|  | 1 | Magnetic breadboard |  | Thorlabs KBM1/M |  |
|  | 1 | Aluminium Headplate Holder | STP file: [link]  Thread size for the 2 thumbscrews: M3  Material: Aluminium 6082 - T651 Grey Aluminium | ProtoLabs.com | On ProtoLabs.com, click 'Get a quote', select 'CNC Machining' and upload the STL file. |
|  |  |  |  |  |  |
|  |  |  |  |  |  |
|  | 2 | Stainless thread M3 thumb screw | 6mm shaft | Mechanical Components P0480.030-006-A4 |  |
|  | 1 | Stainless steel headplate | Specs: [link] |  |  |
|  | 1 | Articulated arm | Strato XS-13 130 mm with M6 mounts (arm only) | Fisso 4.000 | http://fisso.com/pdf/FISSO\_StratoLine\_E.pdf |
|  | 1 | Roll copper wire | Solid core, bare, 16 AWG | RS 357-794 |  |
|  | 1 | M6 hex nut |  | RS 525-919 (or part of Thorlabs HW-KIT2/M) | RS 525-919 is a pack of 250, but only 1 is required |
| Reward system | 1 | Pinch valve | 1-tube, normally closed, 12V DC | NResearch 225P011-21 |  |
|  | 1 | Valve controller driver | NResearch specifications (May 2017) | Nresearch CoolDriveSolo CDS-V01 (UK), CoolDriveSolo CDS-US (USA) | Confirm the linked specifications, as part numbers can be inconsistent |
|  | 1 | Tubing | 1/32" ID x 3/32" OD, 50 ft | Cole Parmer WZ-95702-00 |  |
|  | 2 | Tube connector | Barbed Reducing Connector 3/32" x 1/16" | Cole Parmer WZ-41518-22 |  |
|  | 1 | Power supply | 1.5–12 V DC, 1A linear power supply | RS 615-8919 |  |
|  | 1 | Mini breadboard |  | Pimoroni COM0101 |  |
|  | 1 | Cable mount | 2.1 x 5.5 mm DC (female), 2-pin | RS 810-4605 |  |
|  | 1 | Roll red hookup wire | 300V 22AWG, solid core | Alpha Wire 3051/1 RD005 |  |
|  | 1 | Roll black hookup wire | 300V 22AWG, solid core | Alpha Wire 3051/1 BK005 |  |
| Rig frame | 1 | Breadboard |  | Thorlabs MB3030/M |  |
|  | 3 | Post |  | Thorlabs TR300/M |  |
|  | 3 | Post holder |  | Thorlabs PH50/M |  |
|  | 3 | Adjustable mounting base |  | Thorlabs BA2T2/M |  |
|  | 6 | Component clamp |  | Thorlabs PMTR/M |  |
|  | 6 | V-groove base adapter |  | Thorlabs PCMV/M |  |
|  | 1 | USB camera |  | Logitech C270 |  |
|  | 1 | USB stereo speaker |  | Adafruit 3369 |  |
|  | 12 | 16mm M6 cap screw |  | RS 281-114 (or part of HW-KIT2/M) | RS 281-114 is a pack of 50, but only 12 screws total are required |
| Photo-  diode | 1 | Post |  | Thorlabs TR300/M |  |
|  | 1 | Post |  | Thorlabs TR50/M |  |
|  | 1 | Right-angle clamp |  | Thorlabs RA90/M |  |
|  | 1 | Post holder |  | Thorlabs PH50E/M |  |
|  | 1 | Clamping fork |  | Thorlabs CF125 |  |
|  | 1 | Photodiode |  | Thorlabs PDA100A-EC |  |
|  | 1 | Power supply |  | Thorlabs LDS1212 |  |
|  | 1 | Coaxial cable | 50 Ω, Male BNC to Male BNC, 1m | RS 284-3792 |  |
|  | 1 | Coaxial socket with test leads | Female BNC | Pomona 4969 |  |
| Tools | 1 | Scissors |  |  |  |
|  | 1 | Razor blade |  |  |  |
|  | 1 | Squeegee/straightedge |  |  | This is to remove bubbles during film application, and usually comes with the film |
|  | 1 | Roll of write-on lab tape |  | Cole Parmer WZ-06530-21 |  |
|  | 1 | Spray bottle with ~10% soap solution | ~9:1 water:soap. The solution should be lightly coloured | Any regular dish detergent (e.g., Dawn, Fairy) | Mix gently until the soap is dissolved but the solution is not frothy |
|  | 1 | Air Duster 200 mL |  | Office Depot, Staples, etc. |  |
|  | 1 | 5mm hex key |  | Thorlabs BD-5M | Ensure this is long enough for the bores of the mouse holder |
|  | 1 | 2mm flathead screwdriver |  |  |  |
|  | 1 | Wire stripper/cutter |  |  |  |
|  | 1 | Long-nose pliers |  |  |  |
|  | 1 | Adjustable wrench |  |  |  |
|  | 1 | Roll electrical tape |  |  |  |
|  | 1 | Roll PTFE thread seal tape | 12mm wide x 0.075mm thick (or similar) | RS 512-238 |  |
|  | 1 | Pack poster putty |  | Bostik 'blu tack', Elmer's 'poster tack', etc. |  |

Page
