# Supplementary material for: Rigbox: An Open-Source Toolbox for Probing Neurons and Behavior
Source: eNeuro. 2020 Jul 9;7(4):ENEURO.0406-19.2020. doi: 10.1523/ENEURO.0406-19.2020 (PMC7363478; doi:10.1523/ENEURO.0406-19.2020)
Supplement: Extended Data 1 — We recommend looking at and downloading the code directly from the github repository, at https://github.com/cortex-lab/Rigbox. Download Extended Data 1, ZIP file. [file enu-eN-OTM-0406-19-s02.zip › Rigbox-master-2020-05-18/Rigbox-master/docs/html/Burgess_setup.html]

Burgess steering wheel 

# Burgess steering wheel

Below are some reasonable default hardware configurations for the Burgess steering wheel task

## Contents

- Retrieving hardware file path
- Configuring the stimulus window
- ScreenNum
- SyncBounds:
- PxDepth
- OpenBounds
- MonitorId
- - Performing gamma calibration from command window
- Calibration
- - Signals viewing model
- Hardware inputs
- - DAQ rotary encoder
- - Lick detector
- Hardware outputs
- Timeline
- Weigh scale
- - Using the scale
- Audio devices
- Loading your hardware file
- FAQ
- I tried loading an old hardware file but the variables are not objects.
- I'm missing the time of the first flip only, why?
- The PsychToolbox window covers the wrong monitors when I run the experiment server
- I get a ‘PTB synchronization error’ when I run the experiment server.
- Error using hw.DaqRotaryEncoder/readAbsolutePosition (line 143)
- The experiment server is unable to open my DAQ on ‘Dev1’
- My rotary encoder has a different resolution, how do I change the hardware config?
- Notes
- Etc.

## Retrieving hardware file path

The location of the configuration file is set in DAT.PATHS. If running this on the stimulus computer you can use the following syntax:

```
hardware = fullfile(getOr(dat.paths, 'rigConfig'), 'hardware.mat');
```

## Configuring the stimulus window

The +hw Window class is the main class for configuring the visual stimulus window. It contains the attributes and methods for interacting with the lower level functions that interact with the graphics drivers. Currently the only concrete implementation is support for the Psychophysics Toolbox, the HW.PTB.WINDOW class.

```
stimWindow = hw.ptb.Window;
```

## ScreenNum

The Windows screen index to display the stimulus on. If Windows detects just one monitor (even if you have more plugged into the graphics card), set this to 0 (meaning all screens). Otherwise if you want just the primary display (the one with the menu bar), set it to 1; secondary to 2, etc.

```
stimWindow.ScreenNum = 0; % Use all screen
```

## SyncBounds:

The area over which you can place a photodiode to record stimiulus update times. The simplist way to set this is with the POSITIONSYNCREGION method. Let's put a 100 px^2 sync square in the top right of the window:

```
stimWindow.positionSyncRegion('NorthEast', 100, 100)
```

## PxDepth

Sets the depth (in bits) of each pixel; default is 32 bits. You can usually simply set it based on what the system uses:

```
stimWindow.PxDepth = Screen('PixelSize', stimWindow.ScreenNum);
```

## OpenBounds

The size and position of the window. When left empty the screen will cover the entire screen:

```
stimWindow.OpenBounds = [];
```

## MonitorId

A handy place to store the make or model of monitor used at that rig. As a copy of the hardware is saved each experiment this may be useful for when looking back at old experiments in the future:

```
stimWindow.MonitorId = 'LG LP097QX1'; % The screens used in Burgess et al.
```

## - Performing gamma calibration from command window

## Calibration

This stores the gamma correction tables (See Below) The simplist way to to run the calibration is through SRV.EXPSEERVER once the rest of the hardware is configures, however it can also be done via the command window, assuming you have an NI DAQ installed:

```
lightIn = 'ai0'; % The input channel of the photodiode used to measure screen
clockIn = 'ai1'; % The clocking pulse input channel
clockOut = 'port1/line0 (PFI4)'; % The clocking pulse output channel
% Connect the photodiode to `lightIn` and user a jumper to bridge a
% connection between `clockIn` and `clockOut`.

% Make sure the photodiode is placed against the screen before running
stimWindow.Calibration = stimWindow.calibration(DaqDev); % calibration


save(hardware, 'stimWindow', '-append') % Save the stimWindow to file
```

## - Signals viewing model

Signals currently only supports a single viewing odel. For now the function VIS.SCREEN is used to configure this. Below is an example of configuring the viewing model for the Burgess wheel task, where there are three small screens located at right-angles to one another:

```
help vis.screen
% Below is a schematic of the screen configuration (top-down view).
% ^ represents the observer:
%   _____
%  |     |
%  |  ^  |

% First define some physical dimentions in cm:
screenDimsCm = [19.6 14.7]; %[width_cm heigh_cm], each screen is the same
centerPt = [0, 0, 9.5] % [x, y, z], observer position in cm. z = dist from screen
centerPt(2,:) = [0, 0, 10]% Middle screen, observer slightly further back
centerPt(3,:) = centerPt; % Observer equidistant from left and right motitors
angle = [-90; 0; 90]; % The angle of the screen relative to the observer

% Define the pixel dimentions for the monitors
r = Screen('Resolution', stimWindow.ScreenNum) % Returns the current resolution
pxW = r.width; % e.g. 1280
pxH = r.height; % e.g. 1024

% Plug these values into the screens function:
screens(1) = vis.screen(centerPt(1,:), angle(1), screenDimsCm, [0 0 pxW pxH]);        % left screen
screens(2) = vis.screen(centerPt(2,:), angle(2), screenDimsCm, [pxW 0 2*pxW pxH]);    % ahead screen
screens(3) = vis.screen(centerPt(3,:), angle(3), screenDimsCm, [2*pxW  0 3*pxW pxH]); % right screen

save(hardware, 'screens', '-append');
```

## Hardware inputs

In this example we will add two inputs, a DAQ rotatary encoder and a beam lick detector.

## - DAQ rotary encoder

Create a input for the Burgess LEGO wheel using the HW.DAQROTARYENCODER class:

```
doc hw.DaqRotaryEncoder % More details for this class
mouseInput = hw.DaqRotaryEncoder;

% To deteremine what devices you have installed and their IDs:
daq.getDevices
mouseInput.DaqId = 'Dev1'; % NI DAQ devices are named Dev# by default

% The counter channel which the rotary encoder is connected to:
mouseInput.DaqChannelId = 'ctr0';

% Size of DAQ counter range for detecting over- and underflows (e.g. if
% the DAQ's counter is 32-bit, this should be 2^32).
mouseInput.DaqCounterPeriod = 2^32;

% Setting the encoder resolution and wheel diameter allows us to express
% related experiment parameters in mm and degrees.  These two properties
% are used to calculate the MillimetresFactor property.

% Number of pulses per revolution.  Found at the end of the KÜBLER product
% number, e.g. 05.2400.1122.0100 has a resolution of 100
mouseInput.EncoderResolution = 1024
% Diameter of the wheel in mm
mouseInput.WheelDiameter = 62
```

## - Lick detector

A beam lick detector may be configured to work with an edge counter channel. We can use the HW.DAQEDGECOUNTER class for this:

```
lickDetector = hw.DaqEdgeCounter;

% This is actually a subclass of the HW.DAQROTARYENCODER class, and
% therefore has a few irrelevant properties such as WheelDiameter.  These
% can be ignored.

% To deteremine what devices you have installed and their IDs:
lickDetector.DaqId = 'Dev1'; % NI DAQ devices are named Dev# by default

% The counter channel which the rotary encoder is connected to:
lickDetector.DaqChannelId = 'ctr1';

% Save these two into our hardware file
save(hardware, 'stimWindow', 'lickDetector', '-append')
```

## Hardware outputs

HW.DAQCONTROLLER

```
doc hw.DaqController
daqController = hw.DaqController;

% This class deals with creating DAQ sessions, assigning output
% channels and generating the relevant waveforms to output to each
% channel.

% Example: Setting up water valve interface for a Signals behavour task In
% the romote rig's hardware.mat, instantiate a hw.DaqController object to
% interface with an NI DAQ

% Set the DAQ id (can be found with daq.getDevices)
daqController.DaqIds = 'Dev1';
% Add a new channel
daqController.ChannelNames = {'rewardValve'};
% Define the channel ID to output on
daqController.DaqChannelIds = {'ai0'};
% As it is an analogue output, set the AnalogueChannelsIdx to true
daqController.AnalogueChannelIdx(1) = true;
% Add a signal generator that will return the correct samples for
% delivering a reward of a specified volume
daqController.SignalGenerators(1) = hw.RewardValveControl;
% Set some of the required fields (see HW.REWARDVALVECONTROL for more info)
daqController.SignalGenerators(1).OpenValue = 5; % Volts
daqController.SignalGenerators(1).Calibrations = ...
valveDeliveryCalibration(openTimeRange, scalesPort, openValue,...
  closedValue, daqChannel, daqDevice);

% Save your hardware file
save(hardware, 'daqController', '-append');
```

## Timeline

Timeline unifies various hardware and software times using a DAQ device.

```
doc hw.Timeline

% Let's create a new object and configure some channels
timeline = hw.Timeline

% Setting UseTimeline to true allows timeline to be started by default at
% the start of each experiment.  Otherwise it can be toggled on and off by
% pressing the 't' key while running SRV.EXPSERVER.
timeline.UseTimeline = true;

% Timeline is not usually necessary outside of physiology recordings and
% can be left disabled.

% To set up chrono a wire must bridge the terminals defined in
% Outputs(1).DaqChannelID and Inputs(1).daqChannelID
% The current channal IDs are printed to the command by running the this:
timeline.wiringInfo('chrono');

% They may be changed by setting the above fields, e.g.
timeline.Outputs(1).DaqChannelID = 'port1/line1';
timeline.wiringInfo('chrono'); % New port # displayed

% INPUTS
% Add the rotary encoder
timeline.addInput('rotaryEncoder', 'ctr0', 'Position');
% For a lick detector
timeline.addInput('lickDetector', 'ctr1', 'EdgeCount');
% For a photodiode (see 'Configuring the visual stimuli' above)
timeline.addInput('photoDiode', 'ai2', 'Voltage', 'SingleEnded');

% OUTPUTS
% Say we wanted to trigger camera aquisition at a given frame rate:
clockOut = hw.TLOutputClock;
clockOut.DaqChannelID = 'ctr2'; % Set channal
clockOut.Name = 'Cam-Trigger'; % A memorable name
clockOut.Frequency = 180; % Hz
clockOut.Enable = 'on'; % Switch to enable and disable output
timeline.Outputs(end+1) = clockOut; % Assign to outputs

%Save your hardware.mat file
save(hardware, 'timeline', '-append')

% For more information on configuring and using Timeline, see
% USING_TIMELINE:
open(fullfile(getOr(dat.paths,'rigbox'), 'docs', 'using_timeline.m'))
```

## Weigh scale

MC allows you to log weights through the GUI by interfacing with a digital scale connected via a COM port. This is the only object of use in the MC computer's hardware file.

```
scale = hw.WeighingScale

% The Name field should be set to the name or product code of the scale you
% connect.
scale.Name = 'SPX222';
% The COM port should be set to whichever port the scale is connected to.
% You can find out which ports are availiable in Windows by opening the
% Device Manager (Win + X, then M).  Under Universal Serial Bus, you can
% see all current USB and serial ports.  If you right-click and select
% 'Properties' you can view the port number and even reassign them (under
% Advanced)
scaleComPort = 'COM4'; % Set to a different port
% The TareCommand and FormatSpec fields should be set based on your scale's
% input and output configurations.  Check the manual.
TareCommand = 84; % 'T'
% For SPX222 the weight is transmitted directly, without any units.
% Other scales such as the ES-300HA transmit the weight along with the sign
% and units, e.g. '+ 24.01 g'.
FormatSpec = '%f'

%Save your hardware.mat file
save(hardware, 'scale', '-append')
```

## - Using the scale

The methods are rather self-explanatory. To use the scale the port must first be opened using the INIT method:

```
scale.init()

% To tare (zero) the scale, use the TARE method:
scale.tare()

% To return the last measured weight, use READGRAMS:
g = scale.readGrams()

% Finally the NewReading event allows one to add a listener for weight
% change events.  Let's print the readings to the command window:
callback = @(src,~) fprintf('New reading of %.2fg\n', src.readGrams);
lh = event.listener(scale, 'NewReading', callback);

% To clean up you can simply clear the object from the workspace:
clear scale lh
```

## Audio devices

```
InitializePsychSound
devs = PsychPortAudio('GetDevices')
% Sanitize the names
names = matlab.lang.makeValidName({devs.DeviceName}, 'ReplacementStyle', 'delete');
names = iff(ismember('default', names), names, @()[{'default'} names(2:end)]);
for i = 1:length(names); devs(i).DeviceName = names{i}; end
audioDevices = devs;

save(hardware, 'audioDevices', '-append')
```

## Loading your hardware file

To load your rig hardware objects for testing at a rig, you can use HW.DEVICES:

```
rig = hw.devices;

% To load the hardware file or a different rig, you can input the rig name.
% Note HW.DEVICES initializes some of the hardware by default, including
% creating DAQ sessions and adding any required channels.  To load without
% initializing:
rigName = 'ZREDONE';
initialize = false;
rig = hw.devices(rigName, initialize);
```

## FAQ

## I tried loading an old hardware file but the variables are not objects.

This was probably accompanied with an error such as: \* % Warning: Variable 'rewardController' originally saved as a hw.DaqRewardValve cannot be instantiated as an object and will be read in as a uint32. \*

```
% This usually means that there has been a substantial change in the code
% since the object was last saved and MATLAB can no longer load it into the
% workspace.  One solution is to revert your code to a release dated around
% the time of the hardware file's modified date:
hwPath = fullfile(getOr(dat.paths, 'rigConfig'), 'hardware.mat');
datestr(file.modDate(hwPath)) % Find the time file was last modified

% Once you have the previous parameters, create a new object with the
% current code version, assign the parameters and resave.
```

## I'm missing the time of the first flip only, why?

Perhaps the first flip is always too dark a colour. Try reversing the order stimWindow.SyncColourCycle:

```
scc = stimWindow.SyncColourCycle;
scc = iff(size(scc,1) > size(scc,2), @() flipud(scc), @() fliplr(scc));
stimWindow.SyncColourCycle = scc;
```

## The PsychToolbox window covers the wrong monitors when I run the experiment server

Make sure Mosaic is still running (sometimes if the computer loses a monitor input the graphics card disables Mosaic). One indication of this is that the task bar should stretch across all three of the stimulus screens. Also check that the stimWindow.ScreenNum is correct in the hardware.mat file. When set to 0, PsychToolbox uses all screens available to Windows; 1 means Windows’ primary screen (see the Display Settings); 2 means Windows’ secondary screen, etc.

## I get a ‘PTB synchronization error’ when I run the experiment server.

This happens from time-to-time. When a PsychToolbox window is opened it runs some synchronization to retrace tests, checking whether buffer flips are properly synchronized to the vertical retrace signal of your display. Synchronization failiures indicate that there tareing or flickering may occur during stimulus presentation. More info on this may be found here :

```
web('http://psychtoolbox.org/docs/SyncTrouble')
% The problem may be exacerbated if you're running other programs that
% interfere with the graphics, such as remote window viewers (VNC, Remote
% Desktpo, etc.), or if you are running multiple monitors that do not have
% the same make.  Sometimes simply re-running expServer works.
% If you know what you're doing and are confident that things are working,
% you can skip the tests by setting the following property:
stimWindow.PtbSyncTests = false;
```

## Error using hw.DaqRotaryEncoder/readAbsolutePosition (line 143)

NI Error -88709 ?or Error using hw.DaqRotaryEncoder/createDaqChannel (line 81): The requested subsystem 'CounterInput' does not exist on this device.

```
% This happens from time to time, particularly after the computer has gone
% to sleep. Unplugging the DAQ USB cable and plugging it back in helps.
% Restart MATLAB. If the error persists, restart the computer with the DAQ
% unplugged.
```

## The experiment server is unable to open my DAQ on ‘Dev1’

If you have multiple NI devices on this computer, set the DaqIds properties to the correct id in your hardware.mat file, i.e. daqController.DaqIds, mouseInput.DaqId, rewardController.DaqId

```
d = daq.getDevices % Availiable devices and their info
```

## My rotary encoder has a different resolution, how do I change the hardware config?

Change the mouseInput.EncoderResolution peroperty to the value found at the end of your rotary encoder’s product number: e.g. 05.2400.1122.1024 means EncoderResolution = 1024.

## Notes

(1) DOI:10.1016/j.celrep.2017.08.047

## Etc.

Author: Miles Wells v1.1.0

```
%#ok<*NOPTS>
%#ok<*NASGU>
%#ok<*ASGLU>
```

Published with MATLAB® R2019a
