# Supplementary material for: Rigbox: An Open-Source Toolbox for Probing Neurons and Behavior
Source: eNeuro. 2020 Jul 9;7(4):ENEURO.0406-19.2020. doi: 10.1523/ENEURO.0406-19.2020 (PMC7363478; doi:10.1523/ENEURO.0406-19.2020)
Supplement: Extended Data 1 — We recommend looking at and downloading the code directly from the github repository, at https://github.com/cortex-lab/Rigbox. Download Extended Data 1, ZIP file. [file enu-eN-OTM-0406-19-s02.zip › Rigbox-master-2020-05-18/Rigbox-master/docs/html/glossary.html]

Glossary 

# Glossary

Below is a list of terms and their meaning, along with some links for finding out more information.

## Contents

- Block file
- Experiment Definition (expDef)
- Experiment Framework
- Experiment Reference String (expRef)
- expServer
- Main Repository
- Master Computer (MC)
- mc
- Stimulus Computer (SC)
- Etc.

## Block file

The 'block' file is a MAT file that contains most of the data acquired during an experiment. It is saved by the Experiment class upon experiment quit and follows the following file name pattern: yyyy-mm-dd\_n\_subject\_Block.mat.

See also dat.paths, loading\_experiment\_data

## Experiment Definition (expDef)

The experiment definition function (expDef) is a user-created function that the Signals Experiment class uses to map the hardware inputs to the outputs. The function has the following signature:

```
function exampleWorld(t, evts, p, vs, in, out, audio)
    [...]
end
```

See also SignalsPrimer2?

## Experiment Framework

The 'Experiment Framework' is everything defined by the exp.Experiment base class. This class defines some basic methods (e.g. run, quit, saveDave, useRig) and 'experiment phases' (experimentInit, experimentStarted, experimentEnded) within which stuff happens and updates are broadcast to any listener such as MC. An Experiment object stores and uses various objects throughout the experiment, for instance an exp.ConditionServer object that manages the trial parameter conditions and an io.Communicator that manages the sending of updates to remote listeners. All experiments run in Rigbox (i.e. via mc and/or srv.expServer) use this framework.

exp.SignalsExp extends this framework to use Signals for implementing a user defined experiment function (expDef). NB: Signals can be set up and used outside of this framework. For an example of this, see signals/docs/example/ringach98.m

For more info, see the Experiment package:

```
helpwin +exp
```

## Experiment Reference String (expRef)

An 'experiment reference string' or 'expRef' (sometimes just 'ref') is a char array of the form yyyy\_mm\_dd\_n\_subject. These are unique, human-readable references to an experiment session. Whenever an experiment is started in mc, a new expRef is created, reflecting the folder structure of the main repository, where the parameter set is saved.

See The Data Package

## expServer

srv.expServer is the function that loads rig hardware and allows users to run experiments either locally or via MC.

## Main Repository

The 'main experiment repository' or 'main repo' is the primary location where your experiment data is saved. It is the 'mainRepository' field returned by dat.paths (defined in your +dat/paths.m file). When new experiments are created, a parameter set is saved into this location according to the following directory structure: subject/date/number. srv.expServer looks in this location when loading parameters for a given experiment, and saves the main data files here.

See also dat\_package, dat.expExists

## Master Computer (MC)

The computer that controls the starting and stopping of experiments. This computer runs the mc function, which creates a GUI for doing this.

## mc

mc is the function used for logging, parameterizing and creating new experiments.

## Stimulus Computer (SC)

The 'stimulus computer', 'stim server' or 'SC' is the computer that runs srv.expServer and produces visual and auditory stimuli. All hardware devices used during an experiment are configured for this computer.

## Etc.

Home

Author: Miles Wells

v1.0.0

Published with MATLAB® R2019a
