# Supplementary material for: Rigbox: An Open-Source Toolbox for Probing Neurons and Behavior
Source: eNeuro. 2020 Jul 9;7(4):ENEURO.0406-19.2020. doi: 10.1523/ENEURO.0406-19.2020 (PMC7363478; doi:10.1523/ENEURO.0406-19.2020)
Supplement: Extended Data 1 — We recommend looking at and downloading the code directly from the github repository, at https://github.com/cortex-lab/Rigbox. Download Extended Data 1, ZIP file. [file enu-eN-OTM-0406-19-s02.zip › Rigbox-master-2020-05-18/Rigbox-master/docs/html/hardware_config.html]

Introduction 

# Introduction

When running srv.expServer the hardware settings are loaded from a MAT file and initialized before an experiment. The MC computer may also have a hardware file, though this isn't essential. The below script is a guide for setting up a new hardware file, with examples mostly pertaining to replicating the Burgess steering wheel task(1). Not all uncommented lines will run without error, particularly when a specific hardware configuration is required. Always read the preceeding text before running each line.

It is recommended that you copy this file and keep it as a way of versioning your hardware configurations. In this way the script can easily be re-run when after any unintended changes are made to your hardware file. If you do this, make sure you save a copy of the calibration strutures or re-run the calibrations.

Note that the variable names saved to the hardware file must be the same as those below in order for various Rigbox functions to recognize them, namely these variables:

- stimWindow - The hw.Window object for PsychToolbox parameters
- stimViewingModel - A viewing model used by legacy experiments
- mouseInput - The rotary encoder device
- lickDetector - A lick detector device
- timeline - The Timeline object
- daqController - NI DAQ output settings for use during an experiment
- scale - A weighing scale device for use by the MC computer
- screens - Parameters for the Signals viewing model
- audioDevices - Struct of parameters for each audio device

NB: Not all uncommented lines will run without error, particularly when a specific hardware configuration is required. Always read the preceeding text before running each line.

## Contents

- The +hw packages
- Retrieving hardware file path
- Configuring the stimulus window
- ScreenNum
- SyncBounds
- SyncColourCycle
- PxDepth
- OpenBounds
- DaqSyncEchoPort
- BackgroundColour
- MonitorId
- PtbSyncTests
- PtbVerbosity
- ColourRange, White, Black, etc.
- Calibration (performing gamma calibration from command window)
- Using the Window object
- - Setting the background colour
- - Displaying a Gabor patch
- - Clearing the window
- - Drawing text to the screen
- - Closing a window
- Viewing models
- Basic screen viewing model
- Using the model
- Pseudo-Circular screen viewing model
- Signals viewing model
- Hardware inputs
- DAQ rotary encoder
- Lick detector
- Hardware outputs
- Timeline
- Inputs
- Outputs
- Weigh scale
- Using the scale
- Audio devices
- Loading your hardware file
- FAQ
- I tried loading an old hardware file but the variables are not objects.
- I'm missing the time of the first flip only, why?
- The PsychToolbox window covers the wrong monitors when I run the experiment server
- I get a ‘PTB synchronization error’ when I run the experiment server.
- Error using hw.DaqRotaryEncoder/readAbsolutePosition (line 143)
- The experiment server is unable to open my DAQ on ‘Dev1’
- My rotary encoder has a different resolution, how do I change the hardware config?
- Notes
- Etc.

## The +hw packages

Many of these classes for are found in the HW package. This package contains all of the code that interfaces with lower-level hardware code (e.g. PsychToolbox's OpenGL functions, the NI +daq package):

```
doc hw
```

## Retrieving hardware file path

The location of the configuration file is set in DAT.PATHS. If running this on the stimulus computer you can use the following syntax:

```
hardware = fullfile(getOr(dat.paths, 'rigConfig'), 'hardware.mat');

% For more info on setting the paths and using the DAT package:
rigbox = getOr(dat.paths, 'rigbox'); % Location of Rigbox code
open(fullfile(rigbox, 'docs', 'scripts', 'paths_config.m'))
open(fullfile(rigbox, 'docs', 'scripts', 'using_dat_package.m'))
```

## Configuring the stimulus window

The +hw Window class is the main class for configuring the visual stimulus window. It contains the attributes and methods for interacting with the lower level functions that interact with the graphics drivers. Currently the only concrete implementation is support for the Psychophysics Toolbox, the hw.ptb.Window class.

```
doc hw.ptb.Window
stimWindow = hw.ptb.Window;

% Most of the properties directly mirror PsychToolbox parameters, therefore
% it's recommended to check their documentation for clarification:
%
help Screen
Screen OpenWindow? % Most properties are used as inputs to this function

% Look at these for a deeper understanding of PTB:
help PsychDemos
help PsychBasic

% Below are some of the more important properties:
%
```

## ScreenNum

The Windows screen index to display the stimulus on. If Windows detects just one monitor (even if you have more plugged into the graphics card), set this to 0 (meaning all screens). Otherwise if you want just the primary display (the one with the menu bar), set it to 1; secondary to 2, etc.

```
stimWindow.ScreenNum = 0; % Use the single, main screen
```

## SyncBounds

The area over which you can place a photodiode to record stimiulus update times. A 4-element vector with [topLeftX topLeftY bottomRightX bottomRightY] results in a square in that location that flips between the values in SyncColourCycle each time the window updates (see 'Screen Flip?'). By default the sync square alternates between black and white. These filps can be acquired by Timeline in order to record the times at which stimuli actually appeared on the monitor. (See 'Timeline' section below)

```
% Leave this empty if you don't need to record the screen update times.  By
% convention pixel [0, 0] is defined as the top left-most pixel of the
% monitor. For a screen of 1024 px height create a 100 px^2 sync patch in
% the bottom left corner of the screen:
stimWindow.SyncBounds = [0 924 100 1024];
% The simplist way to set this is with the POSITIONSYNCREGION method.
% Let's put a 100 px^2 sync square in the top right of the window:
stimWindow.positionSyncRegion('NorthEast', 100, 100)
```

## SyncColourCycle

A vector of luminance values or nx3 matrix RGB values to cycle through each time the window updates. Starts at the first index / row. Cycle between black and white on each flip:

```
stimWindow.SyncColourCycle = [0; 255];
```

## PxDepth

Sets the depth (in bits) of each pixel; default is 32 bits. You can usually simply set it based on what the system uses:

```
Screen PixelSize? % More info here
stimWindow.PxDepth = Screen('PixelSize', stimWindow.ScreenNum);
```

## OpenBounds

The size and position of the window. When left empty the screen will cover the entire screen. For debugging it is useful to set the bounds:

```
res = Screen('Resolution', stimWindow.ScreenNum);
% Set to 800x600 window 50 px from the top left:
stimWindow.OpenBounds = [50,50,850,650];
```

## DaqSyncEchoPort

The DaqSyncEchoPort is the channel on which to output a pulse each time the stimulus window is re-drawn. This can be useful for convolving the photodiode signal during analysis, particularly when the photodiode trace is noisy. It can also be a way of confirming whether a photodiode is detecting all of the sync square changes. Note: Sync pulses are not yet supported in Signals, only in legacy experiments.

```
% If this is left empty no sync pulse is set up.
% Ensure the DaqVendor and DaqDev properties are set correctly.
daq.getVendors % Query availiable vendors
daq.getDevices % Query availiable devices and their IDs
DaqSyncEchoPort = 'port1/line0'; % Output fulse on first digital output chan
```

## BackgroundColour

The clut index (scalar, [r g b] triplet or [r g b a] quadruple) defining background colour of the stimulus window during legacy experiments. These should be integers between 0-255. If empty the default is usually middle grey:

```
stimWindow.BackgroundColour = 127*[1 1 1];

% Note that for Signals experiment, the background colour can currently
% only be at when calling SRV.EXPSERVER before an experiment, e.g.
srv.expServer([], [0 127 127]) % Run the experiment with no red gun
```

## MonitorId

A handy place to store the make or model of monitor used at that rig. As a copy of the hardware is saved each experiment this may be useful for when looking back at old experiments in the future:

```
stimWindow.MonitorId = 'LG LP097QX1'; % The screens used in Burgess et al.
```

## PtbSyncTests

A logical indicting whether or not to test synchronization to retrace upon open. When true it tests whether buffer flips are properly synchronized to the vertical retrace signal of your display. If these tests fail PTB throws a warning but continues as normal. Synchronization failiures indicate that there tareing or flickering may occur during stimulus presentation. More info on this may be found here:

```
web('http://psychtoolbox.org/docs/SyncTrouble')
% When blank the global setting is used:
not(Screen('Preference', 'SkipSyncTests')) % Default true; run tests
stimWindow.PtbSyncTests = true;
```

## PtbVerbosity

A number from 0 to 5 indicating the level of verbosity during the experiment. If empty the global preference is used.

```
Screen('Preference', 'Verbosity') % Global verbosity setting
% Below are the levels:
% 0 - Disable all output - Same as using the SuppressAllWarnings flag.
% 1 - Only output critical errors.
% 2 - Output warnings as well.
% 3 - Output startup information and a bit of additional information. This
%     is the default.
% 4 - Be pretty verbose about information and hints to optimize your code
%     and system.
% 5 - Levels 5 and higher enable very verbose debugging output, mostly
%     useful for debugging PTB itself, not generally useful for end-users.
stimWindow.PtbVerbosity = 2;
```

## ColourRange, White, Black, etc.

These properties are set by the object iteself after running OPEN, based on the colour depth of the screen. For more info see these docs:

```
help WhiteIndex
help BlackIndex
```

## Calibration (performing gamma calibration from command window)

This stores the gamma correction tables (See Below) The simplist way to to run the calibration is through srv.expServer once the rest of the hardware is configured, however it can also be done via the command window, assuming you have an NI DAQ installed:

```
lightIn = 'ai0'; % The input channel of the photodiode used to measure screen
clockIn = 'ai1'; % The clocking pulse input channel
clockOut = 'port1/line0 (PFI4)'; % The clocking pulse output channel
% Connect the photodiode to `lightIn` and user a jumper to bridge a
% connection between `clockIn` and `clockOut`.

% Make sure the photodiode is placed against the screen before running
stimWindow.Calibration = ...
  stimWindow.calibration(DaqDev, lightIn, clockIn, clockOut);

save(hardware, 'stimWindow', '-append') % Save the stimWindow to file
```

## Using the Window object

Let's check the Window object is set up correctly and explore some of the methods...

## - Setting the background colour

```
stimWindow.open() % Open the window
stimWindow.BackgroundColour = stimWindow.Green; % Change the background
stimWindow.flip(); % Whoa!
```

## - Displaying a Gabor patch

Make a texture and draw it to the screen with makeTexture and drawTexture Let's make a Gabor patch as an example:

```
sz = 1000; % size of texture matrix
[xx, yy] = deal(linspace(-sz/2,sz/2,sz)');
phi = 2*pi*rand; % randomised cosine phase
sigma = 100; % size of Gaussian window
thetaCos = 90; % grating orientation
lambda = 100; % spatial frequency
targetImg = vis.gabor(xx, yy, sigma, sigma, lambda, 0, thetaCos, phi);
blankImg = repmat(stimWindow.Gray, [size(targetImg), 1]);
targetImg = repmat(targetImg, [1 1 3]); % replicate three colour channels
targetImg = round(blankImg.*(1 + targetImg));
targetImg = min(max(targetImg, 0), 255); % Rescale values to 0-255

% Convert the Gabor image to an OpenGL texture and load into buffer.
% For more info: Screen MakeTexture?, Screen PreloadTextures?
tex = stimWindow.makeTexture(round(targetImg));
% Draw the texture into window (More info: Screen DrawTexture?)
stimWindow.drawTexture(tex)
% Flip the buffer:
stimWindow.flip;
```

## - Clearing the window

To clear the window, the use the clear method:

```
stimWindow.clear % Re-draw background colour
stimWindow.flip; % Flip to screen
```

## - Drawing text to the screen

Drawing text to the screen can be done with the drawText method:

```
[x, y] = deal('center'); % Render the text to the center
[nx, ny] = stimWindow.drawText('Hello World', x, y, stimWindow.Red);
stimWindow.flip;

% The nx and ny outputs may be used again as inputs to add to the text:
[nx, ny] = stimWindow.drawText('Hello World', x, y, stimWindow.Red);
stimWindow.drawText('! What''s up?', nx, ny, stimWindow.Red);
stimWindow.flip;
```

## - Closing a window

Finally lets clear and close the window:

```
stimWindow.clear
stimWindow.close
```

## Viewing models

The viewing model classes allow one to configure the relationship between physical dimentions and pixel space. The viewing model classes contain methods for converting between visual degrees, pixels and physical dimentions. Note: The viewing model classes are currently only implemented in legacy experiments such as ChoiceWorld. See below section for configuring the viewing model in Signals.

There are currently two viewing model classes to choose from...

## Basic screen viewing model

The basic viewing model class deals with single screens positioned straight in front of an observer (^):| \_\_\_ | ^

```
doc hw.BasicScreenViewingModel

% Let's set this up:
stimViewingModel = hw.BasicScreenViewingModel;
% There are three parameters to set:
% A position vector [x,y,z] of the subject in metres, with respect to
% the (centre of the) top left pixel of the screen. x and y are aligned
% with the standard graphics axes (i.e. x to the right, y going down),
% while z extends out from the screen perpendicular to the plane of the
% display).
stimViewingModel.SubjectPos = [0, 0, 0.5]; % Observer centered at 50cm from screen

% Number of pixels across the screen. Also see the function
% USEGRAPHICSPIXELWIDTH to deduce this directly from the graphics hardware:
stimViewingModel.useGraphicsPixelWidth(stimWindow.ScreenNum)
stimViewingModel.ScreenWidthPixels % e.g. 1900 px

% The physical width of the screen, in metres. Pixels are assumed to have a
% 1:1 aspect ratio.
stimViewingModel.ScreenWidthMetres = 0.4750;

save(hardware, 'stimViewingModel', '-append')
```

## Using the model

The object contains useful methods for converting between visual and graphics space:

```
% Visual field coordinates of a specified pixel.  The presumed
% 'straight-ahead' view pixel should map to the centre of the visual field
% (zero polar and visual angles)
x = 0; y = 100; % Convert this pixel coordinate to visual angle
[polarAngle, visualAngle] = stimViewingModel.viewAtPixel(x, y)
% Polar angle is just the angle from central fixation pixel to specified
% (and increases anticlockwise from horizon->right).

% We can get the screen pixel of a given visual field locus. This may be
% useful e.g. for placing stimuli at a certain point in the subject's visual
% field. Let's convert
% back to pixel space:
[x, y] = stimViewingModel.pixelAtView(polarAngle, visualAngle) % ~[0, 100]

% Visual angle between two pixel points.  This is useful if you want to
% measure graphics dimensions in visual angles:
[x2, y2] = deal(0); % Compare above to centre pixel
rad = stimViewingModel.visualAngleBetweenPixels(x, y, x2, y2)
% Radians to degrees:
deg = rad2deg(rad)

% Return the 'visual' pixel density (px per rad) at a point.  This is
% useful for choosing spatial frequency of stimuli at a certain point on
% the screen:
pxPerRad = stimViewingModel.visualPixelDensity(x, y)
% Screen distance in pixels, d, as a function of visual angle, t:
% d(t) = zPx*tan(t)
% Derivative w.r.t. t yields pixel density at a given visual angle:
% d'(t) = zPx*sec(t)^2
```

## Pseudo-Circular screen viewing model

```
doc hw.PseudoCircularScreenViewingModel

stimViewingModel = hw.PseudoCircularScreenViewingModel
```

## Signals viewing model

Signals currently only supports a single viewing odel. For now the function VIS.SCREEN is used to configure this. Below is an example of configuring the viewing model for the Burgess wheel task, where there are three small screens located at right-angles to one another:

```
help vis.screen
% Below is a schematic of the screen configuration (top-down view).
% ^ represents the observer:
%   _____
%  |     |
%  |  ^  |

% First define some physical dimentions in cm:
screenDimsCm = [19.6 14.7]; %[width_cm heigh_cm], each screen is the same
centerPt = [0, 0, 9.5] % [x, y, z], observer position in cm. z = dist from screen
centerPt(2,:) = [0, 0, 10]% Middle screen, observer slightly further back
centerPt(3,:) = centerPt; % Observer equidistant from left and right motitors
angle = [-90; 0; 90]; % The angle of the screen relative to the observer

% Define the pixel dimentions for the monitors
r = Screen('Resolution', stimWindow.ScreenNum) % Returns the current resolution
pxW = r.width; % e.g. 1280
pxH = r.height; % e.g. 1024

% Plug these values into the screens function:
screens(1) = vis.screen(centerPt(1,:), angle(1), screenDimsCm, [0 0 pxW pxH]);        % left screen
screens(2) = vis.screen(centerPt(2,:), angle(2), screenDimsCm, [pxW 0 2*pxW pxH]);    % ahead screen
screens(3) = vis.screen(centerPt(3,:), angle(3), screenDimsCm, [2*pxW  0 3*pxW pxH]); % right screen

save(hardware, 'screens', '-append');
```

## Hardware inputs

In this example we will add two inputs, a DAQ rotatary encoder and a beam lick detector.

## DAQ rotary encoder

Create a input for the Burgess LEGO wheel using the HW.DAQROTARYENCODER class:

```
doc hw.DaqRotaryEncoder % More details for this class
mouseInput = hw.DaqRotaryEncoder;

% To deteremine what devices you have installed and their IDs:
daq.getDevices
mouseInput.DaqId = 'Dev1'; % NI DAQ devices are named Dev# by default

% The counter channel which the rotary encoder is connected to:
mouseInput.DaqChannelId = 'ctr0';

% Size of DAQ counter range for detecting over- and underflows (e.g. if
% the DAQ's counter is 32-bit, this should be 2^32).
mouseInput.DaqCounterPeriod = 2^32;

% Setting the encoder resolution and wheel diameter allows us to express
% related experiment parameters in mm and degrees.  These two properties
% are used to calculate the MillimetresFactor property.

% Number of pulses per revolution.  Found at the end of the KÜBLER product
% number, e.g. 05.2400.1122.0100 has a resolution of 100
mouseInput.EncoderResolution = 1024
% Diameter of the wheel in mm
mouseInput.WheelDiameter = 62
```

## Lick detector

A beam lick detector may be configured to work with an edge counter channel. We can use the HW.DAQEDGECOUNTER class for this:

```
lickDetector = hw.DaqEdgeCounter;

% This is actually a subclass of the HW.DAQROTARYENCODER class, and
% therefore has a few irrelevant properties such as WheelDiameter.  These
% can be ignored.

% To deteremine what devices you have installed and their IDs:
lickDetector.DaqId = 'Dev1'; % NI DAQ devices are named Dev# by default

% The counter channel which the rotary encoder is connected to:
lickDetector.DaqChannelId = 'ctr1';

% Save these two into our hardware file
save(hardware, 'stimWindow', 'lickDetector', '-append')
```

## Hardware outputs

HW.DAQCONTROLLER

```
doc hw.DaqController
daqController = hw.DaqController;

% This class deals with creating DAQ sessions, assigning output
% channels and generating the relevant waveforms to output to each
% channel.

% Example: Setting up water valve interface for a Signals behavour task In
% the romote rig's hardware.mat, instantiate a hw.DaqController object to
% interface with an NI DAQ

% Set the DAQ id (can be found with daq.getDevices)
daqController.DaqIds = 'Dev1';
% Add a new channel
daqController.ChannelNames = {'rewardValve'};
% Define the channel ID to output on
daqController.DaqChannelIds = {'ai0'};
% As it is an analogue output, set the AnalogueChannelsIdx to true
daqController.AnalogueChannelIdx(1) = true;
% Add a signal generator that will return the correct samples for
% delivering a reward of a specified volume
daqController.SignalGenerators(1) = hw.RewardValveControl;
% Set some of the required fields (see HW.REWARDVALVECONTROL for more info)
daqController.SignalGenerators(1).OpenValue = 5; % Volts
daqController.SignalGenerators(1).Calibrations = ...
valveDeliveryCalibration(openTimeRange, scalesPort, openValue,...
  closedValue, daqChannel, daqDevice);

% Save your hardware file
save(hardware, 'daqController', '-append');
```

## Timeline

Timeline unifies various hardware and software times using a DAQ device. There is a separate guide for Timeline here.

```
doc hw.Timeline

% Let's create a new object and configure some channels
timeline = hw.Timeline

% Setting UseTimeline to true allows timeline to be started by default at
% the start of each experiment.  Otherwise it can be toggled on and off by
% pressing the 't' key while running SRV.EXPSERVER.
timeline.UseTimeline = true;

% Timeline is not usually necessary outside of physiology recordings and
% can be left disabled.

% To set up chrono a wire must bridge the terminals defined in
% Outputs(1).DaqChannelID and Inputs(1).daqChannelID
% The current channal IDs are printed to the command by running the this:
timeline.wiringInfo('chrono');

% They may be changed by setting the above fields, e.g.
timeline.Outputs(1).DaqChannelID = 'port1/line1';
timeline.wiringInfo('chrono'); % New port # displayed
```

## Inputs

Add the rotary encoder

```
timeline.addInput('rotaryEncoder', 'ctr0', 'Position');
% For a lick detector
timeline.addInput('lickDetector', 'ctr1', 'EdgeCount');
% For a photodiode (see 'Configuring the visual stimuli' above)
timeline.addInput('photoDiode', 'ai2', 'Voltage', 'SingleEnded');
```

## Outputs

Say we wanted to trigger camera aquisition at a given frame rate:

```
clockOut = hw.TLOutputClock;
clockOut.DaqChannelID = 'ctr2'; % Set channal
clockOut.Name = 'Cam-Trigger'; % A memorable name
clockOut.Frequency = 180; % Hz
clockOut.Enable = 'on'; % Switch to enable and disable output
timeline.Outputs(end+1) = clockOut; % Assign to outputs

%Save your hardware.mat file
save(hardware, 'timeline', '-append')

% For more information on configuring and using Timeline, see
% TIMELINE:
open(fullfile(getOr(dat.paths,'rigbox'), 'docs', 'scripts', 'Timeline.m'))
```

## Weigh scale

MC allows you to log weights through the GUI by interfacing with a digital scale connected via a COM port. This is the only object of use in the MC computer's hardware file.

```
scale = hw.WeighingScale

% The Name field should be set to the name or product code of the scale you
% connect.
scale.Name = 'SPX222';
% The COM port should be set to whichever port the scale is connected to.
% You can find out which ports are availiable in Windows by opening the
% Device Manager (Win + X, then M).  Under Universal Serial Bus, you can
% see all current USB and serial ports.  If you right-click and select
% 'Properties' you can view the port number and even reassign them (under
% Advanced).  You can also list all available ports by running |seriallist|
% (|serialportlistt("available")| for >2019b).
scaleComPort = 'COM4'; % Set to a different port
% The TareCommand and FormatSpec fields should be set based on your scale's
% input and output configurations.  Check the manual.
TareCommand = 84; % 'T'
% For SPX222 the weight is transmitted directly, without any units.
% Other scales such as the ES-300HA transmit the weight along with the sign
% and units, e.g. '+ 24.01 g'.
FormatSpec = '%f'

%Save your hardware.mat file
save(hardware, 'scale', '-append')
```

## Using the scale

The methods are rather self-explanatory. To use the scale the port must first be opened using the INIT method:

```
scale.init()

% To tare (zero) the scale, use the TARE method:
scale.tare()

% To return the last measured weight, use READGRAMS:
g = scale.readGrams()

% Finally the NewReading event allows one to add a listener for weight
% change events.  Let's print the readings to the command window:
callback = @(src,~) fprintf('New reading of %.2fg\n', src.readGrams);
lh = event.listener(scale, 'NewReading', callback);

% To clean up you can simply clear the object from the workspace:
clear scale lh
```

## Audio devices

On MS-Windows you'll have the choice between up to 5 different audio subsystems: WASAPI (on Windows-Vista and later), or WDMKS (on Windows-2000/XP) should provide ok latency. DirectSound is the next worst choice if you have hardware with DirectSound support. If everything else fails, you'll be left with MME, a completely unusable API for precise or low latency timing.

```
InitializePsychSound
devs = PsychPortAudio('GetDevices')

% Sanitize the names
names = matlab.lang.makeValidName({devs.DeviceName}, 'ReplacementStyle', 'delete');
names = iff(ismember('default', names), names, @()[{'default'} names(2:end)]);
for i = 1:length(names); devs(i).DeviceName = names{i}; end
audioDevices = devs;

save(hardware, 'audioDevices', '-append')

% @TODO Substantiate
% @body Info on PTB support, mention the helper for testing devices,
% mention how audio device naming works
```

## Loading your hardware file

To load your rig hardware objects for testing at a rig, you can use hw.devices:

```
rig = hw.devices;

% To load the hardware file or a different rig, you can input the rig name.
% Note HW.DEVICES initializes some of the hardware by default, including
% creating DAQ sessions and adding any required channels.  To load without
% initializing:
rigName = 'ZREDONE';
initialize = false;
rig = hw.devices(rigName, initialize);
```

## FAQ

## I tried loading an old hardware file but the variables are not objects.

This was probably accompanied with an error such as: \* % Warning: Variable 'rewardController' originally saved as a hw.DaqRewardValve cannot be instantiated as an object and will be read in as a uint32. \*

```
% This usually means that there has been a substantial change in the code
% since the object was last saved and MATLAB can no longer load it into the
% workspace.  One solution is to revert your code to a release dated around
% the time of the hardware file's modified date:
hwPath = fullfile(getOr(dat.paths, 'rigConfig'), 'hardware.mat');
datestr(file.modDate(hwPath)) % Find the time file was last modified

% Once you have the previous parameters, create a new object with the
% current code version, assign the parameters and resave.
```

## I'm missing the time of the first flip only, why?

Perhaps the first flip is always too dark a colour. Try reversing the order stimWindow.SyncColourCycle:

```
scc = stimWindow.SyncColourCycle;
scc = iff(size(scc,1) > size(scc,2), @() flipud(scc), @() fliplr(scc));
stimWindow.SyncColourCycle = scc;
```

## The PsychToolbox window covers the wrong monitors when I run the experiment server

Make sure Mosaic is still running (sometimes if the computer loses a monitor input the graphics card disables Mosaic). One indication of this is that the task bar should stretch across all three of the stimulus screens. Also check that the stimWindow.ScreenNum is correct in the hardware.mat file. When set to 0, PsychToolbox uses all screens available to Windows; 1 means Windows' primary screen (see the Display Settings); 2 means Windows' secondary screen, etc.

## I get a ‘PTB synchronization error’ when I run the experiment server.

This happens from time-to-time. When a PsychToolbox window is opened it runs some synchronization to retrace tests, checking whether buffer flips are properly synchronized to the vertical retrace signal of your display. Synchronization failiures indicate that there tareing or flickering may occur during stimulus presentation. More info on this may be found here :

```
web('http://psychtoolbox.org/docs/SyncTrouble')
% The problem may be exacerbated if you're running other programs that
% interfere with the graphics, such as remote window viewers (VNC, Remote
% Desktpo, etc.), or if you are running multiple monitors that do not have
% the same make.  Sometimes simply re-running expServer works.
% If you know what you're doing and are confident that things are working,
% you can skip the tests by setting the following property:
stimWindow.PtbSyncTests = false;
```

## Error using hw.DaqRotaryEncoder/readAbsolutePosition (line 143)

NI Error -88709 ?or Error using hw.DaqRotaryEncoder/createDaqChannel (line 81): The requested subsystem 'CounterInput' does not exist on this device.

```
% This happens from time to time, particularly after the computer has gone
% to sleep. Unplugging the DAQ USB cable and plugging it back in helps.
% Restart MATLAB. If the error persists, restart the computer with the DAQ
% unplugged.
```

## The experiment server is unable to open my DAQ on ‘Dev1’

If you have multiple NI devices on this computer, set the DaqIds properties to the correct id in your hardware.mat file, i.e. daqController.DaqIds, mouseInput.DaqId, rewardController.DaqId

```
d = daq.getDevices % Availiable devices and their info
```

## My rotary encoder has a different resolution, how do I change the hardware config?

Change the mouseInput.EncoderResolution peroperty to the value found at the end of your rotary encoder’s product number: e.g. 05.2400.1122.1024 means EncoderResolution = 1024.

## Notes

(1) DOI:10.1016/j.celrep.2017.08.047

## Etc.

Author: Miles Wells

v1.1.1

```
%#ok<*NOPTS,*NASGU,*ASGLU>
```

Published with MATLAB® R2019a
