# Supplementary material for: Rigbox: An Open-Source Toolbox for Probing Neurons and Behavior
Source: eNeuro. 2020 Jul 9;7(4):ENEURO.0406-19.2020. doi: 10.1523/ENEURO.0406-19.2020 (PMC7363478; doi:10.1523/ENEURO.0406-19.2020)
Supplement: Extended Data 1 — We recommend looking at and downloading the code directly from the github repository, at https://github.com/cortex-lab/Rigbox. Download Extended Data 1, ZIP file. [file enu-eN-OTM-0406-19-s02.zip › Rigbox-master-2020-05-18/Rigbox-master/docs/html/id_index.html]

Error and warning IDs 

# Error and warning IDs

Below is a list of Rigbox error & warning IDs. This list is currently incomplete and there aren't yet very standard. Typically the ID has the following structure: module:package:function:error

These are here for search convenience and may soon contain more detailed troubleshooting information.

## Contents

- copyPaths
- noRemoteFile
- notInTest
- behaviourNotSet
- mkdirFailed
- newExp:expFoldersAlreadyExist
- ----- ! PTB - ERROR: SYNCHRONIZATION FAILURE ! ----
- Undocumented IDs

## copyPaths

**Problem**:

In order to load various essential configuration files, and to load and save experimental data, user specific paths must be retrieved via calls to dat.paths. This error means the function is not on MATLAB's search path.

**Solution**:

Add your +dat\paths.m file to MATLAB's search path. A template is present in docs\setup\paths\_template.m. This file is automatically copied by addRigboxPaths to +dat\. If you haven't already done so, run addRigboxPaths to ensure all other paths have been correctly set.

See also README.md for further setup information.

**IDs**

```
Rigbox:git:update:copyPaths
signals:test:copyPaths
```

## noRemoteFile

**Problem**: % TODO Add problem & solution for noRemoteFile error

**Solution**:

**IDs**

```
Rigbox:mc:noRemoteFile
```

## notInTest

**Problem**:

This occurs when a mock function is called when the INTEST global variable is not set. These mock functions shadow Rigbox and builtin functions, meaning they have the same name.

**Solution**:

If this function was called during a test, add the following to the top of your test or in the constructor:

```
global INTEST
INTEST = true
```

Ensure that this is cleared during the teardown:

```
addteardown(@clear, INTEST) % If in a class
mess = onCleanup(@clear, INTEST) % If in a function
```

If the mock in question is a class, set the InTest flag instead of the global variable:

```
mock = MockDialog; % An example using MockDialog class
mock.InTest = true;
addteardown(@clear, MockDialog) % Clear mock class when done
mess = onCleanup(@clear, MockDialog) % If in a function
```

If you are in not running tests, ensure that tests/fixtures is not in your MATLAB path and that you are in a different working directory. It is best to remove all Rigbox paths and readd them using `addRigboxPaths`

**IDs**

```
Rigbox:tests:system:notInTest
Rigbox:tests:modDate:notInTest
Rigbox:tests:paths:notInTest
Rigbox:tests:pnet:notInTest
Rigbox:tests:modDate:notInTest
Rigbox:MockDialog:newCall:notInTest
```

## behaviourNotSet

**Problem**:

A mock function was called while in a test, however the behaviour for this particular input has not been defined.

**Solution**:

If not testing a specific behavior for this function's output, simply supress the warning in your test, remembering to restore the warning state:

```
origState = warning;
addteardown(@warning, origState) % If in a class
mess = onCleanup(@warning, origState) % If in a function
warning('Rigbox:MockDialog:newCall:behaviourNotSet', 'off')
```

If you're specifically testing the behavior when the mock returns a particular output then check that you've set the input-output map correctly: usually this is done by first calling the mock with input identical to function under test as well as the output you want to see. Check the input is formatted correctly. For more information see the help of the particular mock you are using.

**IDs**

```
Rigbox:tests:system:valueNotSet % TODO change name
Rigbox:MockDialog:newCall:behaviourNotSet
```

## mkdirFailed

**Problem**:

MATLAB was unable to create a new folder on the system.

**Solution**:

In general Rigbox code only creates new folders when a new experiment is created. The folders are usually created in the localRepository and mainRepository locations that are set in your paths file. If either of these are remote (e.g. a server accessed via SMB) check that you can navigate to the location in Windows' File Explorer (sometimes the access credentials need setting first). If you can, next check the permissions of these locations. If the folders are read-only, MATLAB will not be able to create a new experiment folder there. Either change the permissions or set a different path in dat.paths. One final thing to check is that the folder names are valid: the presence of a folder that is not correctly numbered in the subject's date folder may lead to an invalid expRef. Withtin a date folder there should only be folders name '1', '2', '3', etc.

**IDs**

```
Alyx:newExp:mkdirFailed
Rigbox:dat:newExp:mkdirFailed
```

## newExp:expFoldersAlreadyExist

**Problem**:

The folder structure for a newly generated experiment reference is already in place.

Experiment references are generated based on subject name, today's date and the experiment number, which is found by looking at the folder structure of the main repository. In a subject's experiment folder for a given date there are numbered folders. When running a new experiment, the code takes the folder name with the largest number and adds 1. It then checks that this numbered folder doesn't exist in the other repositories. If it does, an error is thrown so that no previous experiment data is overwritten.

**Solution**:

Check the folder structure for all your repositories (namely the localRepository and mainRepository set in dat.paths). It may be that there is an empty experiment folder in the localRepository but not the mainRepository, in which case you can delete it. Alternatively, if you find a full experiment folder in the local but not the main, copy it over so that the two match. This will avoid a duplicate expRef being created (remember, new expRefs are created based on the folder structure of the mainRepository only).

**IDs**

```
Alyx:newExp:expFoldersAlreadyExist
Rigbox:dat:newExp:expFoldersAlreadyExist
```

```
% ..:..:expRefNotFound
% *Problem*:
%
% The experiment reference string does not correspond to the folder
% structure in your mainRepository path.  Usually determined via a call to
% |dat.expExists|.
%
% *Solution*:
%
% Check that the mainRepository paths are the same on both the computer
% that creates the experiment (e.g. MC) and the one that loads the
% experiment (e.g. the one that runs |srv.expServer|).  For an experiment
% to exist, the subject > date > sequence folder structure should exist in
% the mainRepository.  To see the mainRepository location, run the
% following:
%
%   getOr(dat.paths, 'mainRepository')
%
```

For example if the output is \\server\Subjects\ then for the expRef '2019-11-25\_1\_test' to exist, the following folder should exist: \\server\Subjects\test\2019-11-25\1

**IDs**

```
Rigbox:srv:expServer:expRefNotFound
```

## ----- ! PTB - ERROR: SYNCHRONIZATION FAILURE ! ----

**Problem**:

To quote PsychToolbox: One or more internal checks indicate that synchronization of Psychtoolbox to the vertical retrace (VBL) is not working on your setup.This will seriously impair proper stimulus presentation and stimulus presentation timing!

**Solution**:

There are many, many reasons for this error. Here's a quick list of things to try, in order:

1. Simply re-trying a couple of times. Sometimes it happens sporadically.
2. Check the monitor(s) are on and plugged in. If you're using multiple monitors they should be of the same make and model. If they aren't, try with just one monitor first.
3. If you're using multiple screens in NVIDEA's 'Mosaic' mode, the settings may have changed: sometimes Mosiac becomes deactivated and you should set it up again.
4. If you're using a remote connection for that computer it may be interfering with the graphics settings. Examples of a remote connection include VNC servers, TeamViewer and Windows Remote Desktop. Try opening the PTB Window without any of these remote services.
5. Update the graphics card drivers and firmware. This often helps.
6. Read the PTB docs carefully and follow their suggestions. The docs can be found at http://psychtoolbox.org/docs/SyncTrouble.
7. If all else fails. You can skip these tests and check that there is no taring manually. This is not recommended but can be done by setting your stimWindow object's PtbSyncTests property to false:

```
stimWindow = getOr(hw.devices([],false), 'stimWindow');
stimWindow.PtbSyncTests = false;
hwPath = fullfile(getOr(dat.paths, 'rigConfig'), 'hardware.mat');
save(hwPath, 'stimWindow', '-append')
```

## Undocumented IDs

```
Below is a list of all other error and warning ids.
```

```
% Rigbox:git:runCmd:nameValueArgs
% Rigbox:git:runCmd:gitNotFound
% Rigbox:git:update:valueError
%
% Rigbox:hw:calibrate:noscales
% Rigbox:hw:calibrate:deadscale
% Rigbox:hw:calibrate:partialPVpair
%
% Rigbox:srv:unexpectedUDPResponse
% Rigbox:srv:unexpectedUDP
% Rigbox:srv:expServer:noHardwareConfig
%
% Rigbox:dat:expPath:NotEnoughInputs
% Rigbox:exp:SignalsExp:NoScreenConfig
% Rigbox:exp:Parameters:wrongNumberOfColumns
%
% Rigbox:dat:expFilePath:NotEnoughInputs
%
% Rigbox:MockDialog:newCall:EmptySeq
%
% Rigbox:exp:SignalsExp:noTokenSet
%
% Rigbox:eui:choiceExpPanel:toolboxRequired
% Rigbox:setup:toolboxRequired
%
% Alyx:newExp:subjectNotFound
% Alyx:registerFile:InvalidPath
% Alyx:registerFile:UnableToValidate
% Alyx:registerFile:EmptyDNSField
% Alyx:registerFile:InvalidRepoPath
% Alyx:registerFile:InvalidFileType
% Alyx:registerFile:InvalidFileName
% Alyx:registerFile:NoValidPaths
% Alyx:updateNarrative:UploadFailed
%
% Alyx:getFile:InvalidID
% Alyx:getExpRef:InvalidID
% Alyx:getFile:InvalidType
% Alyx:expFilePath:InvalidType
% Alyx:url2Eid:InvalidURL
%
% toStr:isstruct:Unfinished
%
% squeak.hw
% shape:error
% window:error
```

Published with MATLAB® R2019a
