# Supplementary material for: Rigbox: An Open-Source Toolbox for Probing Neurons and Behavior
Source: eNeuro. 2020 Jul 9;7(4):ENEURO.0406-19.2020. doi: 10.1523/ENEURO.0406-19.2020 (PMC7363478; doi:10.1523/ENEURO.0406-19.2020)
Supplement: Extended Data 1 — We recommend looking at and downloading the code directly from the github repository, at https://github.com/cortex-lab/Rigbox. Download Extended Data 1, ZIP file. [file enu-eN-OTM-0406-19-s02.zip › Rigbox-master-2020-05-18/Rigbox-master/docs/html/index.html]

Rigging Toolbox Documentation 

# Rigging Toolbox Documentation

Welcome to the main Rigging Toolbox (Rigbox) documentation. Here you can find detailed documentation and guides on how to set up Rigbox, including writing new experiments, setting up recording devices, running experiments and processing the resulting data.

## Contents

- Installing Rigbox
- Setting up experiments
- Running full experiments
- Creating experiments
- Working with Rigbox Experiment data
- Troubleshooting
- User guides
- Miscellaneous
- Etc.

## Installing Rigbox

Below are some instructions for installing Rigbox. There are two guides, the first is a thorough guide for users unfarmilliar with MATLAB and Git. The second is for 'power users' who have a basic understanding of these things.

1. Full install instructions
2. Installing for power users

## Setting up experiments

Below is a set of steps for setting up a full experiment in Rigbox. A full experiment being one where you record quality, reliable data that gets saved into the main experiment repository.

Briefly, before you can run a full experiment you must 1) set up your paths so that Rigbox knows from where to load rig settings and parameters, 2) save a hardware configuration file so that Rigbox can properly initialize its hardware, and 3) locate or create an experiment definition function to define your experiment.

1. Setting up dat.paths
2. How to configure hardware on the stimulus computer
3. Setting up communication between the stimulus computer and MC
4. Setting up mc and srv.expServer on the same computer

## Running full experiments

Before you can run a complete experiment, you must set up Rigbox (see above section). Once this is done there you can follow on of the below sections to run a full experiment.

## Creating experiments

The principle way to create a new is experiment (i.e. passive stimulation or behaviour task) is write an expDef. Below will be a set of guides for how to write an expDef, and how to test it.

- Playing around with Signals Experiment Definitions

## Working with Rigbox Experiment data

Below are some guides on how to work with the experimental data saved by Rigbox. These guides instruduce some functions for loading and processing these data, and explain the forms in which data are saved.

- Working with wheel data
- Working with block files
- Working with ALF files

## Troubleshooting

Rigbox is a mountain of code and there are many things that can go wrong when using it. Below are a few guides for how to fix problems that arise in Rigbox.

- Basic MATLAB troubleshooting - this guide is for users that are unfamiliar with MATLAB.
- General troubleshooting - this guide gives a list of steps to follow when an error is encountered.
- ID index - A list of Rigbox error/warning IDs along with the a detailed description of what they mean and an exhastive list of causes and solutions.
- FAQ - A list of frequently asked questions regarding problems and pointers to the solutions.

## User guides

Below is a list of in-depth guides for users who want to learn the ins-and-outs of various packages and classes in Rigbox.

- The Data Package - How to query data locations and log experiments using the +dat package.
- How Signals works - An in-depth guide to how Signals works. This shows you how to work with Signals outside of the Experiment Framework and gives demonstrations of all Signals methods ( scan, etc.)
- Parameters - How to create and edit experiment parameters.
- Alyx - How to interact with an Alyx database
- Timeline - Using Timeline for time alignment

## Miscellaneous

Below is a list of useful topics:

- Setting up auxiliary services
- How to create a custom Experiment Panel
- Glossary of Rigbox terminology

## Etc.

Author: Miles Wells

v0.1.1

Published with MATLAB® R2019a
