# Supplementary material for: Rigbox: An Open-Source Toolbox for Probing Neurons and Behavior
Source: eNeuro. 2020 Jul 9;7(4):ENEURO.0406-19.2020. doi: 10.1523/ENEURO.0406-19.2020 (PMC7363478; doi:10.1523/ENEURO.0406-19.2020)
Supplement: Extended Data 1 — We recommend looking at and downloading the code directly from the github repository, at https://github.com/cortex-lab/Rigbox. Download Extended Data 1, ZIP file. [file enu-eN-OTM-0406-19-s02.zip › Rigbox-master-2020-05-18/Rigbox-master/docs/html/install.html]

Installing Rigbox 

# Installing Rigbox

Below are some easy step-by-step instructions for installing Rigbox without any prior knowledge of Git or MATLAB. For more succinct installation instructions, see the Rigbox README. The first section lists the requirements. NB: Please read this section carefully before attempting to install anything.

## Contents

- Requirements
- Software
- Hardware
- Install steps
- Notes
- Etc.

## Requirements

For exploring Rigbox's features and running test experiments, Rigbox only needs to be installed on a single computer.

For running full experiments, we recommend running Rigbox on two PCs: one is used for presenting stimuli and communicating with rig hardware, and the other runs a GUI which the user interacts with to monitor the experiment. Currently only National Instruments DAQs are supported for acquiring data from hardware devices.

## Software

Rigbox reqquires the following software to work properly:

- Windows Operating System (7 or later, 64-bit)
- MATLAB (2018b or later, also known as version 9.5)
- Visual C++ Redistributable Packages for Visual Studio 2013 & 2015-2019 (*free*)
- The MATLAB Data Acquisition Toolbox
- *If using an NI DAQ* the NI-DAQmx support package (*free*)
- The GUI Layout Toolbox (v2 or later, *free*)
- Psychophysics Toolbox (v3 or later, *free*)

## Hardware

Below are a few minimum hardware requirements for both PCs.

- **Processor**: Intel Core i5-6500 @ 3.0 GHz (or similar)
- **Graphics**: NVIDIA Quadro P400 (or similar)
- **Memory**: DDR4 16 GB @ 2133 MHz (e.g. Corsair Vengeance 16 GB)

## Install steps

Below are detailed steps on installing all required software. If you already have software installed for a particular step, feel free to skip that step. Unless you are installing Rigbox on a computer which will use a NI-DAQ for data acquistion, you can skip step 5.

1. Install Windows 7 or later (Windows 10 is recommended). Windows must be must be 64-bit (sometimes called x64, x86\_64, AMD64 or Intel 64).
2. Download and install https://uk.mathworks.com/downloads/ MATLAB> by following their installation guide (see note 1). At step 9, make sure to check the box for the Data Acquisition Toolbox, along with any other MATLAB Mathworks toolboxes you want, though for testing Rigbox, no other toolboxes are required (See note 2). NB: This step may take a while.
3. Once downloaded, open MATLAB by double-clicking on the MATLAB icon in the start menu.
4. Within MATLAB, install the GUI Layout Toolbox (See note 2). Make sure the version number is greater than 2.
5. Within MATLAB, install the NI-DAQmx Support Package (See note 2). NB: This step may take a while.
6. Download and install the Microsoft Visual C++ Redistributable Packages for Visual Studio 2013 installer (See note 3).
7. Download and install the Microsoft Visual C++ Redistributable Packages for Visual Studio 2015-2019 installer (See note 4). NB: This is 2015-2019; the previous step was for 2013. Both are required.
8. Download and install SilkSVN (See note 5).
9. Download and install the 64-Bit GStreamer-1.16.0 MSVC runtime, making sure to install all offered packages (See note 6).
10. Download the PsychToolbox installer function and save it into your Documents/MATLAB folder.
11. In the MATLAB Command Window (see note 7), type DownloadPsychtoolbox(userpath) (no quotes) and press enter. This will download and install PsychToolbox to MATLAB folder. At certain points in the installation it will print stuff to the Command Window and ask you to press any key to continue. Do this until the two angled brackes ('|>>|') reappear.
12. Close MATLAB by pressing the '|X|' in the top right corner of the window.
13. Download and install Git Bash for Windows (See note 8). Use all defaults.
14. Launch Git Bash (See note 9). A black command line window should appear.
15. Type the following line into Git Bash (or copy/paste): cd ~/Documents/Github
16. Copy this line and paste it into Git Bash (use right click for pasting): git clone --recurse-submodules https://github.com/cortex-lab/Rigbox
17. Launch MATLAB and navigate to the following folder (See note 10): Documents\Github\Rigbox
18. Type the following into the MATLAB Command Window and press enter (See note 11): addRigboxPaths
19. You should be done now. To check this, type eui.SignalsTest; into the MATLAB Command Window and select signalsPong.m. Then click Start. Your mouse controls the right paddle.

## Notes

1. MATLAB is not free and requires a MATLAB account in order to download. If you are part of an academic institution you may be able to get MATLAB for free. If in doubt ask your lab supervisor or institute IT department. For more information see MATLAB's install guide.
2. Once MATLAB is installed, toolboxes can be downloaded and installed directly within MATLAB via the "Add-Ons" button in the "Home" top toolstrip. This opens the MATLAB 'AddOn Explorer' where you can search and install toolboxes.
3. To install download and install, follow the link and click the orange 'Download' button. A 'Choose the download you want' window will come up. Select 'vcredist\_x64.exe' and click 'Next'. A download window will pop up; click 'Save file'. Once downloaded, double-click the installer and follow the steps.
4. To download and install the MSVC 2015-2019 libraries, follow the link and click 'Save file'. You can then run the file and follow the installation steps.
5. To download and install SilkSVN, follow the link and click the blue button that says 'SVN 1.12.0, 64 bit' on the left-hand side. The numbers might be slightly different but the important thing is that you choose the one that says '64 bit'. Click 'OK' in the pop-up window to save the installer zip file. Once downloaded double-click the zip file and open the exe file contained. Follow all the steps in the installer.
6. To download and install the 64-Bit GStreamer-1.16.0 MSVC runtime, follow the link and clock 'Save file'. Then open the file and follow the installation steps. Choose the full/complete installation with all packages selected.
7. The MATLAB Command Window is usually at the bottom of the MATLAB window and has a '|>>|' in it. For more information, please read the MATLAB documentation about the Command Window.
8. To download and install Git Bash for Windows, follow the link and click 'Save file' when the download window pops up. Open the installer file and click 'Next' repeatedly until the end, then click 'Finish'.
9. There might be more than one program installed that has 'Git' in the name. Make sure the one you open is called 'Git Bash'.
10. To navigate to a folder in MATLAB, either use the Address Field or type the following into the MATLAB Command Window, replacing USER with the name of the Windows user that's currently logged in: cd('C:\Users\USER\Documents\Github\rigbox\')
11. If you've followed the above steps you can safely ignore any warnings you may see for trying out Rigbox.

## Etc.

Authors: Jai Bhagat, Matteo Caranini, Miles Wells

v0.1.0

Published with MATLAB® R2019a
