# Supplementary material for: Rigbox: An Open-Source Toolbox for Probing Neurons and Behavior
Source: eNeuro. 2020 Jul 9;7(4):ENEURO.0406-19.2020. doi: 10.1523/ENEURO.0406-19.2020 (PMC7363478; doi:10.1523/ENEURO.0406-19.2020)
Supplement: Extended Data 1 — We recommend looking at and downloading the code directly from the github repository, at https://github.com/cortex-lab/Rigbox. Download Extended Data 1, ZIP file. [file enu-eN-OTM-0406-19-s02.zip › Rigbox-master-2020-05-18/Rigbox-master/docs/html/packages.html]

Code organization 

# Code organization

Rigbox is organized into various MATLAB packages(1). Functions and classes within a package have their own namespace. We seperate our functions based broadly on what part of Rigbox they're for. For example files in the +eui folder are part of the 'Experiment UI' package and are all related to GUIs in Rigbox (e.g. the MC GUI or Parameter Editor GUI).

*Fig. 1* - An overview of the packages and their loose relationships to one another

Below is a list of Rigbox's subdirectories and an overview of their general purpose. Shown is the command for viewing the package overview doc, which lists the package contents. For further details, see the REAME.md and Contents.m files for each package folder.

## Contents

- +dat
- +eui
- +exp
- +hw
- +psy
- +srv
- cb-tools/burgbox
- cortexlab
- tests
- docs
- submodules
- Notes
- Etc.

## +dat

The 'data' package contains code pertaining to the organization and logging of data. It contains functions that generate and parse unique experiment reference ids, and return file paths where subject data and rig configuration information is stored. Other functions include those that manage experimental log entries and parameter profiles. A nice metaphor for this package is a lab notebook.

```
doc +dat
```

## +eui

The 'experiment user interface' package contains code pertaining to the Rigbox user interface. It contains code for constructing the mc GUI (MControl.m), and for plotting live experiment data or generating tables for viewing experiment parameters and subject logs.

This package is exclusively used by the master computer.

```
doc +eui
```

## +exp

The 'experiment' package is for the initialization and running of behavioural experiments. It contains code that define a framework for event- and state-based experiments. Actions such as visual stimulus presentation or reward delivery can be controlled by experiment phases, and experiment phases are managed by an event-handling system (e.g. ResponseEventInfo).

The package also triggers auxiliary services (e.g. starting remote acquisition software), and loads parameters for presentation for each trial. The principle two base classes that control these experiments are 'Experiment' and its 'signals package' counterpart, 'SignalsExp'.

```
helpwin +exp
```

## +hw

The 'hardware' package is for configuring, and interfacing with, hardware (such as screens, DAQ devices, weighing scales and lick detectors). Within this is the '+ptb' package which contains classes for interacting with PsychToolbox.

hw.devices loads and initializes all the hardware for a specific experimental rig. There are also classes for unifying system and hardware clocks.

```
doc hw
```

## +psy

The 'psychometrics' package contains simple functions for processing and plotting psychometric data.

```
doc psy
```

## +srv

The 'stim server' package contains the 'expServer' function as well as classes that manage communications between rig computers.

The 'Service' base class allows the stimulus computer to start and stop auxiliary acquisition systems at the beginning and end of experiments.

The 'StimulusControl' class is used by the master computer to manage the stimulus computer.

**Note**: Lower-level communication protocol code is found in the 'cortexlab/+io' package.

```
doc +srv
```

## cb-tools/burgbox

'Burgbox' contains many simple helper functions that are used by the main packages. Within this directory are additional packages:

- +bui --- Classes for managing graphics objects such as axes
- +aud --- Functions for interacting with PsychoPortAudio
- +file --- Functions for simplifying directory and file management, for instance returning the modified dates for specified folders or filtering an array of directories by those that exist
- +fun --- Convenience functions for working with function handles in MATLAB, e.g. functions similar cellfun that are agnostic of input type, or ones that cache function outputs
- +img --- Classes that deal with image and frame data (DEPRECATED)
- +io --- Lower-level communications classes for managing UDP and TCP/IP Web sockets
- +plt --- A few small plotting functions (DEPRECATED)
- +vis --- Functions for returning various windowed visual stimuli (i.g. gabor gratings)
- +ws --- An early Web socket package using SuperWebSocket (DEPRECATED)

## cortexlab

The 'cortexlab' directory is intended for functions and classes that are rig or CortexLab specific, for example, code that allows compatibility with other stimulus presentation packages used by CortexLab (e.g. MPEP)

## tests

The 'tests' directory contains code for running unit tests within Rigbox.

## docs

Contains various guides for how to configure and use Rigbox.

## submodules

Additional information on the alyx-matlab, npy-matlab, signals and wheelAnalysis submodules can be found in their respective github repositories.

## Notes

(1) For more information about using packages in MATLAB, see Scoping Classes With Packages

## Etc.

Author: Miles Wells

v0.0.2

Published with MATLAB® R2019a
