# Supplementary material for: Rigbox: An Open-Source Toolbox for Probing Neurons and Behavior
Source: eNeuro. 2020 Jul 9;7(4):ENEURO.0406-19.2020. doi: 10.1523/ENEURO.0406-19.2020 (PMC7363478; doi:10.1523/ENEURO.0406-19.2020)
Supplement: Extended Data 1 — We recommend looking at and downloading the code directly from the github repository, at https://github.com/cortex-lab/Rigbox. Download Extended Data 1, ZIP file. [file enu-eN-OTM-0406-19-s02.zip › Rigbox-master-2020-05-18/Rigbox-master/docs/html/Parameters.html]

Parameters 

# Parameters

This document contains information on how experiment parameters work in Rigbox. How to create them, edit them and save them.

NB: Not all uncommented lines will run without error, particularly when a specific hardware configuration is required. Always read the preceeding text before running each line.

## Contents

- Loading parameters
- Parameter structures
- Special parameters
- numRepeats
- randomiseConditions
- defFunction
- expPanelFun
- type
- services
- waterType
- isPassive
- repeatIncorrect
- Saving parameter sets
- The Parameters object
- The ParamEditor GUI
- Global panel
- Conditions panel
- Advanced trial conditions
- Using Set values
- Notes and warning suppressions

## Loading parameters

Parameters are structures that contain experiment and session specific variables that may be set via a GUI and stored for each session. Parameters are stored as structures saved MAT files and may also be saved as JSON. They typically end in '\_parameters' and are saved when creating a new experiment using the dat.newExp function or Alyx/newExp method. You can generate and retrieve paths for experiment parameter files using the dat package:

```
expRef = dat.constructExpRef('fake', now, 1); % Example experiment reference
paramPath = dat.expFilePath(expRef, 'parameters') % Generate path for saving

% Parameters can be loaded using an experiment reference via DAT.EXPPARAMS:
paramStruct = dat.expParams(expRef); % Returns empty if params don't exist

% More info on generating paths can be found in USING_DAT_PACKAGE:
root = getOr(dat.paths,'rigbox');
open(fullfile(root, 'docs', 'scripts', 'using_dat_package.m'))
```

## Parameter structures

In signals a set of parameters may be extracted using EXP.INFERPARAMETERS (see note 1).

defFunction should be either an experiment definition function handle, or if the function isn't on MATLAB's search path, a char of the full path to such a definition function.

```
defFunction = fullfile(getOr(dat.paths,'rigbox'), 'tests', ...
  'fixtures', 'expDefinitions', 'advancedChoiceWorld.m');
paramStruct = exp.inferParameters(defFunction);

% Parameter structures are scalar structs where each field is a parameter
% name written in CamelCase/pascalCase (written this way the parameters
% will be displayed as seperate words in GUIs such as the ParamEditor)
%
% There are two kinds of parameters: global and conditional.  Global
% parameter values are independent of trial number, whereas conditional
% parameters represent a range of possible values which vary across
% trials.  Conditional parameters are those that have more than one column.
% Note that for this reason, all conditional parameters much have the same
% number of columns.
%
% Examples:

% A global parameter whose value is a charecter
paramStruct.rewardKey % (1,1)
% A global parameter whose value is a column vector
paramStruct.preStimulusDelay % (3,1)
% A conditional parameter whose values are column (2,1) column vectors.
% There are 11 different possible trial conditions
paramStruct.stimulusContrast % (2,11)
% Note that for char arrays, this convention is reversed.  Thus the folling
% is treated as a global parameter, despite having more that 1 columns
paramStruct.expPanelFun % (1,27)

% Parameters may take a number of different value types, including
% numerical, string and char arrays.
```

## Special parameters

There are a few parameters added by EXP.INFERPARAMETERS, EXP.PARAMETERS and EUI.PARAMEDITOR that should not be set in your experiment definition:

## numRepeats

The number of times each condition shall be repeated. By default there are 1000 'trials', split evenly across each condition (parameter column). When trial number > sun(numRepeats), the experiment will automatically end. If numRepeats is a global parameter (i.e. a scalar value) each condition will be repeated the same number of times.

## randomiseConditions

When true (default) the conditional trial parameters are shuffled at the start of the experiment and thus occur in a random order.

## defFunction

For signals experiments this parameter is the file path of the experiment definition. This is required for the stimulus computer to load the correct experiment. It does not appear as a parameter during the experiment.

## expPanelFun

A char or function handle of the experiment panel class to use for visualization during the experiment in MC. This allows users to display custom plots, etc. for monitoring different experiments. See EUI.EXPPANEL, EUI.SIGNALSEXPPANEL (default) for more info.

## type

A legacy parameter that defines what experiment class to use. Options include 'ChoiceWorld' and 'custom', where the latter indicates a signals experiment. For these two options the experiment classes are EXP.CHOICEWORLD and EXP.SIGNALSEXP, respectively (see note 2).

## services

A cellstr array of service names to be activaed during experiment setup. These are external systems that can be triggered via websockets and UDPs. (See note 3)

## waterType

The type of water reward to administer during the experiment. This could allow one to switch between different DAQ outputs and will change the water\_type field when posting to Alyx. This feature is not yet fully implemented.

## isPassive

When true the experiment is a re-play of a previous one. This feature is not yet fully implemented.

In addition to these, parameters can be attributed descriptions and units by setting fields with the same name the end in 'Description' and 'Units' respectively. These are displayed in the ParamEditor GUI. See below.

```
paramStruct.wheelGain % An example parameter that needs a description
paramStruct.wheelGainDescription = ['Visual stimulus translation per ',...
  'movement at wheel surface (for stimuli ahead)'];
paramStruct.visWheelGainUnits = '°/mm';

% There is one special parameter default that may be set in the definition
% function: repeatIncorrect
```

## repeatIncorrect

This is the only special parameter that may have a default set in the definition function. This parameter must be a boolian and in signals, when both the repeatIncorrect parameter and the endTrial signal evaluate as true, the current conditional parameters will be repeated on the following trial. See note 4.

## Saving parameter sets

exp.inferParameters is useful for loading an experiment's default parameters (set in the definition function itself). Also, each time you run an experiment a copy of the modified parameter set is saved in the experiment folder. The last used parameters for a given experiment and subject are loaded by default in MC.

Suppose however that you want to save and load a parameter set for a given experiment, independent of the subject? For this we use the dat package...

To save a new parameter set simply call dat.saveParamProfile:

```
paramStruct.rewardSize = 5; % We want to save our modified params
paramSetName = 'highReward'; % Give our saved set a name
dat.saveParamProfile('custom', paramSetName, paramStruct)

% The saved parameter sets are usually found in the main repository:
fullfile(dat.reposPath('main', 'master'), 'parameterProfiles.mat')

% This file contains a stuct for each experiment type, e.g. a variable
% called 'custom' for saved signals parameter sets.  This contains multiple
% fields corresponding to the given name of the parameter set.
savedSets = dat.loadParamProfiles('custom') % Returns all saved parameters for signals

% Finally parameters profiles may be deleted using DAT.DELPARAMPROFILE:
dat.delParamProfile('custom', paramSetName);

% These parameter sets can also be loaded and saved in MC using the
% drop-down menu above the ParamEditor.
```

## The Parameters object

Modifying sturctures directly is error-prone and time consuming for certain tasks. To manipulate parameters more easily we use the exp.Parameters class.

```
parameters = exp.Parameters(paramStruct) % a new Parameters object

% The Parameters object contains fields indicating which parameters are
% conditional and which are global.  The underlying structure is stored in
% the `Struct` property and it is this that should be saved, not the object
% itself.

% A parameter may be created or modified using the SET method:
% parameters.set(name, value, description, units)
parameters.set('incorrectTimeout', 2, 'Timeout on incorrect trials', 's')
% You can retrieve the description for one or more parameters with the
% DESCRIPTION method:
parameters.description({'incorrectTimeout', 'wheelGain'})
% The TITLE method returns the parameter name formatted for titles:
parameters.title('onsetToneFrequency') % 'Onset tone frequency'

% To remove a parameter, simply remove it from the Struct property:
parameters.Struct = rmfield(parameters.Struct, 'incorrectTimeout');

% Parameters may moved between global and condition using the
% MAKETRIALSPECIFIC and MAKEGLOBAL methods:
parameters.isTrialSpecific('wheelGain') % false; parameter is global
parameters.makeTrialSpecific('wheelGain') % Make trial conditional
parameters.isTrialSpecific('wheelGain') % true

% Note the values are now repeated:
numTrialConditions(parameters) % There are 11 trial conditions
parameters.Struct.wheelGain % (1,11)

% Let's make global again.  Note you can set the new value at the same
% time.  If no `newValue` arg is given, the value of the first trial
% conditon is used.
parameters.makeGlobal('wheelGain', 3)
parameters.isTrialSpecific('wheelGain') % false

% Conditions may be removed using the REMOVECONDITIONS parameter.  This
% removes the given column indices in all conditional parameters
numTrialConditions(parameters) % There are 11 trial conditions
parameters.removeConditions([2, 8]) % Remove conditions 2 and 8
numTrialConditions(parameters) % There are 9 trial conditions

% Finally, parameters may be assorted for an experiment using the following
% two methods...

% ASSORTFOREXPERIMENT returns the global parameters and trial condition
% parameters as separate structures.  The `globalParams` structure is a
% scalar struct of all global parameters, while `trialParams` is a
% non-scalar struct of all trial conditions.  This can be useful for
% looking over the parameters without using a GUI.
[globalParams, trialParams] = parameters.assortForExperiment;
trialParams % (1,9) struct

% TOCONDITIONSERVER replicates the trial conditions be however many times
% are specified by the 'numRepeats' parameter and if the `randomOrder`
% input arg is true, these are shuffled into a random order.  This method
% is used by the experiment class during setup.  In addition to returning
% the assorted global and conditional parameters, a Condition Server
% object, `cs` is also returned (see note 5)
[cs, globalParams, trialParams] = parameters.toConditionServer(true);
sum(parameters.Struct.numRepeats) % Total number of trials
trialParams % (1,810) struct

% c.f. [~, ~, trialParams] = parameters.toConditionServer(false)
% See section below for more details on randomizing.
```

## The ParamEditor GUI

The easiest way to modify parameters is via the ParamEditor GUI, managed by the eui.ParamEditor class. A ParamEditor object is embedded into MC and can also be instantiated via the Experiment Panels.

To instantiate a standalone editor, call EUI.PARAMEDITOR with a Parameters object. Additionally a parent figure handle may be provided.

There are two panels that make up the editor. On the left are the global parameters and on the right is the trial conditions table, containing the conditional parameters.

```
PE = eui.ParamEditor(parameters)
```

## Global panel

The global panel is pretty simple. Each parameter is represented by the parameter name (formatted as a title, see above section) and an input field. When a field is modified the title turns red. For numrical and string parameters, vectors may be added by inputting values separated by commas. For instance typing '1, 3, 5' (without quotes) would set the value of that parameter to [1; 3; 5]. For numrical parameters spaces alone will suffice, e.g. '1 3 5'.

To make a parameter conditional, right-clicking on the input field or title and select 'Make conditional'. The parameter will now appear in the conditions table to the right.

## Conditions panel

Here each parameter is represented as a table column, and its conditions (i.e. the columns of the parameter) as table rows. The rows may be re-ordered by dragging the field name of the column you with to move.

New blank conditions (i.e. rows) can be added by clicking the 'New condition' button at the bottom of the table.

Individual cells can be selected and edited as expected. To select multiple cells, hold down the ctrl key while clicking. To select multiple conditions in a row, hold the shift key. Once you've selected a cell in at least one column, the other buttons become available:

'Delete condition' allows you to delete the table rows of the selected cells (i.e. the selected trial conditions).

'Globalise parameter' makes the columns of the selected cells global parameters, whereby they are moved to the left panel. The value in the last selected cell for that parameter is used as the new parameter value. This may also be done via the context menu.

'Set values' allows you can set multiple cells are once. One clicked a small dialog appears with am input field for each selected column. The number of selected cells for each column is shown in brackets. Entering a single value will set each cell to that value. Entering a semicolon-separated list will assign each cell one value going from top to bottom. For example by selecting three cells in a column and typing '4; 5; 6' (without quotes) the top most cell will take the value '4' and so forth.

This is particularly useful for more involved stimulus sets with many conditions.

By right-clicking anywhere in the condition table you get two extra options:

'Sort by selected column' will sort the columns by whichever column the selected cell is in. Currently only sorting by one column is supported.

'Randomise conditions' is a checkbox that sets the underlying hidden parameter of the same name. By default this is checked. When unchecked, the rows of the table are given an index, indicating the set order trial conditions.

Once your parameters have been modified via the GUI they can be saved by extracting the underlying parameter stuct and saving to a file:

```
dat.saveParamProfile('custom', 'variant_2', PE.Parameters.Struct)
```

## Advanced trial conditions

Let's look at some ways you can set trial condition parameters for different sorts of experiments. In the below examples imagine you have a signals experiment definition with three conditional parameters, A, B and C (see note 6).

By default all conditions are presented in a random order n times, where n is defined by the numRepeats parameter. If numRepeats is made a global parameter, then all conditions are presented the same number of times.

```
% Example 1: An illustration of how the trial order may turn out when
% 'randomiseConditions' is false and 'numRepeats' is a conditional parameter:

% +---+---+---+-------------+
% | A | B | C | Num Repeats |
% +---+---+---+-------------+
% | 1 | 4 | 2 |           2 |
% | 2 | 5 | 1 |           1 |
% | 3 | 3 | 4 |           2 |
% +---+---+---+-------------+
%
% [~, ~, trialParams] = parameters.toConditionServer;
% trialParams =
%
% # | A | B | C |
%   +---+---+---+
% 1 | 3 | 3 | 4 |
% 2 | 2 | 5 | 1 |
% 3 | 3 | 3 | 4 |
% 4 | 1 | 4 | 2 |
% 5 | 1 | 4 | 2 |
%   +---+---+---+


% Example 2: What happens when we globalize 'numRepeats' and give it the
% value of 2?

% +---+---+---+
% | A | B | C |
% +---+---+---+
% | 1 | 4 | 2 |
% | 2 | 5 | 1 |
% | 3 | 3 | 4 |
% +---+---+---+
%
% [~, ~, trialParams] = parameters.toConditionServer;
% trialParams =
%
% # | A | B | C |
%   +---+---+---+
% 1 | 1 | 4 | 2 |
% 2 | 3 | 3 | 4 |
% 3 | 1 | 4 | 2 |
% 4 | 2 | 5 | 1 |
% 5 | 3 | 3 | 4 |
% 6 | 2 | 5 | 1 |
%   +---+---+---+

% Randomized trial conditions are useful for many behaviour protocols and
% even for some passive stimulus mapping experiments, however sometimes you
% require multiple repeats in a row of each conditon.

% Example 3: To present stimuli in a sequence, right click anywhere on the
% conditions table and deselect 'randomise conditions' in the context menu.
% Now each row will be numbered sequentially from 1 to the number of
% conditions (see note 7 for an alternate solution):

%     +---+---+---+-------------+
%     | A | B | C | Num Repeats |
% +---+---+---+---+-------------+
% | 1 | 1 | 4 | 2 |           2 |
% | 2 | 2 | 5 | 1 |           1 |
% | 3 | 3 | 3 | 4 |           2 |
% +---+---+---+---+-------------+
%
% # | A | B | C |
%   +---+---+---+
% 1 | 1 | 4 | 2 |
% 2 | 1 | 4 | 2 |
% 3 | 2 | 5 | 1 |
% 4 | 3 | 3 | 4 |
% 5 | 3 | 3 | 4 |
%   +---+---+---+
```

## Using Set values

For more involved stimulus sets, we can use Set Values to conveniently set the values of multiple cells at once. This is particularly useful for experiments with many conditions.

```
% Example 4: Say you want to present each condition twice in a row, and the
% entire set two times, e.g.
%
% #  | A | B | C |
%    +---+---+---+
% 1  | 1 | 4 | 2 |
% 2  | 1 | 4 | 2 |
% 3  | 2 | 5 | 1 |
% 4  | 2 | 5 | 1 |
% 5  | 3 | 3 | 4 |
% 6  | 3 | 3 | 4 |
% 7  | 1 | 4 | 2 |
% 8  | 1 | 4 | 2 |
% 9  | 2 | 5 | 1 |
% 10 | 2 | 5 | 1 |
% 11 | 3 | 3 | 4 |
% 12 | 3 | 3 | 4 |
%    +---+---+---+
%
% 1) Add enough new conditions to the table so that there are 6 rows.
% 2) Highlight all cells and select 'Set values'.
% 3) An input dialog appears where you can input the values for each
% parameter. For A, you can type repmat([1 2 3],1,2), for B the vector
% would be [4 5 3], etc.
% 4) Globalize the numRepeats parameter and set it to 2
% 5) Ensure 'randomise conditions' is deselected
%
% Note that you can sequences of values using any MATLAB function this way,
% for instance inputting 2:2:12 would set the conditions to be [2 4 6 8
% 12].
%
% Example 5: With set values you can also apply functions to the current
% set of values, for instance to multiply every value of A from the
% previous example by 2, we input @(v)v*2 and to set every value of B to a
% random integer between 1 and 5, @(~)randi(5):
%
% #  | A | B | C |
%    +---+---+---+
% 1  | 2 | 2 | 2 |
% 2  | 2 | 4 | 2 |
% 3  | 4 | 3 | 1 |
% 4  | 4 | 3 | 1 |
% 5  | 6 | 2 | 4 |
% 6  | 6 | 5 | 4 |
% 7  | 2 | 4 | 2 |
% 8  | 2 | 1 | 2 |
% 9  | 4 | 2 | 1 |
% 10 | 4 | 2 | 1 |
% 11 | 6 | 4 | 4 |
% 12 | 6 | 1 | 4 |
%    +---+---+---+
```

## Notes and warning suppressions

(1) To load default parameters for legacy experiments, there are simple functions that return these paramteters. NB: The below are no longer used as signals provides more flexbility in running experiments.

```
choiceParams = exp.choiceWorldParams; % For the original Burgess task
diskParams = exp.discWorldParams; % For a similar orientation-based protocol
rangeParams = exp.rangeParams; % A Burgess task prototype experiment
% Various visual field mapping experiment parameteres
barMappingParams = exp.barMappingParams;
gaborMappingParams = exp.gaborMappingParams; % Gabor patch mapping
flashedColumnParams = exp.flashedColumnParams; % Kalatsky-type

% (2) The type parameter would be a useful way of implementing a new
% experiment stucture, e.g. if you wanted to sub-class EXP.SIGNALSEXP in
% order to implement an entirely differnt set of hardware, changing the
% type parameter would cause the experiment server to call the correct
% experiment class during setup. See also SRV.PREPAREEXP,
% EXP.CONFIGURESIGNALSEXPERIMENT

% (3) Services may be set via the options interface in MC.  For more info
% see SRV.PREPAREEXP, SRV.FINDSERVICE, EUI.MCONTROL/RIGOPTIONS and
% SRV.STIMULUSCONTROL.

% (4) The name originates from the fact that this behaviour is useful as a
% means of anti-biasing during bahaviour training.  For instance in the
% Burgess task the side of the stimulus will be repeated on high contrast
% trials until the subject gived the correct response.  This parameter may
% be used in other contexts, however.

% (5) The condition server object is used in legacy experiments such as
% ChoiceWorld, however EXP.SIGNALSEXP uses a different implementation.  For
% more info see EXP.CONDITIONSERVER and EXP.TRIALCONDITIONS.

% (6) Below is an example definition function:
% function passiveStimWorld(t, evts, par, vs, in, out, audio)
% [...]
% elem.shape = p.A*sin(p.B) + p.C;
% vs.stimulus = elem;
%
% % parameters with more than one column are trial conditions, where each
% % column corresponds to a condition.  All conditional parameters must have
% % the same number of columns.
% try
%   p.A = [1 2 3];
%   p.B = [4 5 3];
%   p.C = [2 1 4];
% catch
% end
% end

% (7) Another way to achieve this is to use the events.endTrial signal:
% when endTrial takes a value the current trial ends. If the value is 0
% (false), then the same condition is repeated on the next trial, otherwise
% the next condition is used. This allows you to present each condition n
% times in a row but with the order of each condition block randomized:

%   function passiveStimWorld(t, evts, p, vs, in, out, audio)
%   % First define a Signal that counts the repeat number of current condition
%   repNum = events.newTrial.scan(@plus, p.skipRepeats().map(0));
%   % Define when the next block of repetitions should occur
%   nextCondition = repNum == p.numConditionReps;
%   % Define trial end
%   events.endTrial = events.newTrial.delay(10).then(nextCondition);
%   [...]
%
% +---+---+---+--------------------+
% | A | B | C | Num Condition Reps |
% +---+---+---+--------------------+
% | 1 | 4 | 2 |                  2 |
% | 2 | 5 | 1 |                  2 |
% | 3 | 3 | 4 |                  2 |
% +---+---+---+--------------------+
% Num Repeats = 1 (global)
%
%
% # | A | B | C |
%   +---+---+---+
% 1 | 2 | 5 | 1 |
% 2 | 2 | 5 | 1 |
% 3 | 3 | 3 | 4 |
% 4 | 3 | 3 | 4 |
% 5 | 1 | 4 | 2 |
% 6 | 1 | 4 | 2 |
%   +---+---+---+
% (Note the blocks of repeated conditions are shuffled)

% Author: Miles Wells
%
% v1.1.1

% INTERNAL:
%  ln209 ParamEditor.png
%#ok<*NOPTS,*ASGLU,*NASGU>
```

Published with MATLAB® R2019a
