# Supplementary material for: Rigbox: An Open-Source Toolbox for Probing Neurons and Behavior
Source: eNeuro. 2020 Jul 9;7(4):ENEURO.0406-19.2020. doi: 10.1523/ENEURO.0406-19.2020 (PMC7363478; doi:10.1523/ENEURO.0406-19.2020)
Supplement: Extended Data 1 — We recommend looking at and downloading the code directly from the github repository, at https://github.com/cortex-lab/Rigbox. Download Extended Data 1, ZIP file. [file enu-eN-OTM-0406-19-s02.zip › Rigbox-master-2020-05-18/Rigbox-master/docs/html/paths_config.html]

Introduction 

# Introduction

The dat.paths function is used for configuring important paths for the computers which Rigbox runs on. These include paths to:

1. either the shared folder(s) OR the remote server(s) on which organization-wide configuration files, subject data and experiment data is stored, and a local directory for generating redundant copies of this data.
2. optionally, paths to a remote database (if using Alyx), and a local redundant copy of that database
3. optionally, paths to any other directories for storing additional back-ups (e.g. for working analyses, tapes, etc...)
4. optionally, a path to a custom config file for the local computer.

## Contents

- Setting up the paths
- Manually copying the paths template
- Sharing a folder in Windows
- Etc.

## Setting up the paths

dat.paths is simply a function that returns a struct of directory paths to various things. Much of the code in Rigbox calls this function to determine where to save and load data.

Running the addRigboxPaths function should result in a copy of the paths template file being moved to the +dat folder. If running `which dat.paths` shows this isn't the case, manually copy the template (see below) and open the file. The inline comments should explain each field.

```
open dat.paths
```

## Manually copying the paths template

The below code should copy docs\setup\paths\_template.m to +dat\paths.m:

```
assert(exist('addRigboxPaths','file') == 2, ...
  'Rigbox not installed.  Please run addRigboxPaths.m before continuing')
root = fileparts(which('addRigboxPaths')); % Location of Rigbox root dir
source = fullfile(root, 'docs', 'setup', 'paths_template.m');
destination = fullfile(root, '+dat', 'paths.m');
assert(copyfile(source, destination), 'Failed to copy the template file')
```

## Sharing a folder in Windows

If you don’t yet have a data server, follow the steps below to set up a shared folder on the stimulus server computer:

1. Create a folder in C:\ called ‘LocalExpCode’ and one called ‘LocalExpData’ (if it doesn’t already exist)
2. Copy everything inside the GitHub\Rigbox\Repositories\data folder into ‘LocalExpData’ and everything inside GitHub\Rigbox\Repositories\code into ‘LocalExpCode’
3. Right click on the ‘LocalExpData’ folder and select Properties and select the Sharing tab and click ‘Share...’
4. Under the drop-down list select ‘Everyone’ and click ‘Add’, then ‘Share’.
5. Now click ‘Advanced Sharing…’, make sure the ‘Share this folder’ check box is selected. Click the ‘Permissions’ button and ensure that under the ‘Permissions for Everyone’ section, the ‘Full Control’ is allowed.
6. Repeat step 3, 4 and 5 for ‘LocalExpCode’.
7. You should now be able to navigate to these folder from other computers on the network be going to \\<StimulusServerName>\LocalExpData (where ‘<StimulusServerName>’ is the computer name of the stimulus server)
8. In dat.paths, change line 21 to be the following: serverName = '\\<StimulusServerName>'; % where '<StimulusServerName>' is the stimulus server’s computer name
9. On lines 26 and 32, replace ‘data’ with ‘LocalExpData’ and on lines 37 and 41, replace the word ‘code’ with ‘LocalExpCode’.
10. Save the paths file in Documents\MATLAB\+dat and do the same on the mc computer (the two computers must have the same paths).

## Etc.

Author: Miles Wells

v0.0.1

Published with MATLAB® R2019a
