# Supplementary material for: Rigbox: An Open-Source Toolbox for Probing Neurons and Behavior
Source: eNeuro. 2020 Jul 9;7(4):ENEURO.0406-19.2020. doi: 10.1523/ENEURO.0406-19.2020 (PMC7363478; doi:10.1523/ENEURO.0406-19.2020)
Supplement: Extended Data 1 — We recommend looking at and downloading the code directly from the github repository, at https://github.com/cortex-lab/Rigbox. Download Extended Data 1, ZIP file. [file enu-eN-OTM-0406-19-s02.zip › Rigbox-master-2020-05-18/Rigbox-master/docs/html/SignalsPrimer.html]

Introduction 

# Introduction

The purpose of this script is to introduce Signals from the bottom up. It explains the logic of Signals, its machinary, how to wire a network and a few of the important functional methods associated. After reading this you should have a near complete understanding of how Signals works and thus how to create the experiments you want. Understanding the machanics will greatly reduce the number of errors you will encounter while making your first experiment. Later, the structure of a Signals Experiment will be introduced. For a briefer guide, see some of the 'tutorials' found in the docs folder. A secod part to this guide will focus on the SignalsExp class and on testing and debugging your experiments.

Bracketed numbers correspond to notes at the bottom of the file. These sections are intended to be run in order. Please report any errors as GitHub issues. Thanks!

## Contents

- Network architecture
- Origin signals
- Demonstration on sig.Signal/output() method
- Relationships between signals
- Mathematical expressions
- Example 1: cos(x \* pi)
- Example 2: x -> degrees
- Logical operations
- mod, floor, ceil
- Arrays
- Matrix arithmatic
- (Take a moment to clear your workspace)
- A note about Signals variables
- Signals can derive from multiple signals
- Complicated expressions
- Example 1: Upper bound
- Example 2: Gabor
- Indexing
- Indexing into a Signal
- Indexing with a Signal
- selectFrom
- indexOfFirst
- Use more complex functions with map
- Anonymous functions
- Example 2: a complex counter
- fun.partial
- Sampling with map
- Mapping constants
- Map multiple Signals through a function with mapn
- More complex conditionals
- Demonstration of scan
- The seed value may be a signal
- Growing an array with scan
- Introducing extra parameters
- Parameters may be Signals
- When pars take new values, accumulator function is not called!
- Scan can call any number of functions at the same time
- Timing in signals
- Now let's increase the frequency to 10 ms...
- When we clear the handle, the value is no longer displayed
- Due to the timer, the value of 'time' continues to update
- When the timer is stopped, the value of 'time' is no longer updated
- Filtering
- Example 2: keepWhen
- Example 3: setTrigger & skipRepeats
- Example 4: to & lag
- Timing 2 - Scheduling
- TODO SetEpochTrigger
- Demonstration of subscriptable signals
- You can not post to subscriptable Signals, nor assign values to them
- Helpful methods
- delta
- lag
- buffer
- bufferUpTo
- Demonstration of sig.Signal/log() method
- Logging signals in a registry
- Visual stimuli
- Notes
- Etc.

## Network architecture

Every signal is part of a network, managed through a sig.Net object. The network object holds all the ids of all the signals' nodes(1).

```
% Every signal has an underlying node; a |sig.node.Node| object that
% contains a number of important properties:
% * Net: a handle to the parent network (a sig.Net object)
% * Inputs: an array of input nodes (other sig.node.Node objects)
% * Id: an integer node ID used by the low level C code
% * NetId: an integer ID for the parent network, used by the low level C code
% * CurrValue: the current value that the node holds

net = sig.Net; % Create a new signals network
```

## Origin signals

An origin signal is a special sub-class of the sig.node.Signal class whose value can be updated directly using the post method. The function call for creating an origin signal takes two inputs: the parent network and optionally, a string identifier.

These origin signals are the input nodes to the reactive network. All other signals are either directly or indirectly dependent on origin signals. Origin signals can take values of any type, as demonstrated below.

In the context of a Signals Experiment, the origin signals would be the timing signal and signals representing hardware devices (a wheel, lever, keyboard, computer mouse, etc...). These origin Signals should be defined outside of your experiment definition function and be input variables. Your experiment definition function (expDef) will define the mapping of these input origin signals to various hardware outputs (more on this later):

```
% inputs --> |          | -->               --> |\ /| -->
%        --> | (expDef) | -->               --> |-X-| -->
%        --> |          | --> outputs       --> |/ \| --> outputs

% You can post values to an origin Signal by using the |post| method.  This
% is not possible with other classes of Signals as their values instead
% depend on the values of their input nodes.

% It is worth noting that every Signal has a |Name| property which may be
% set manually or be set based on its inputs.  The name of a Signal may
% be used by visualization functions(2) to describe its functional
% relationship within the network.  The name property of an origin Signal
% is set as its second input.  Signals are handle objects and therefore may
% be assigned to any variable name.  Hence there are two means to identify
% a Signal: it's true name (the string held in the Name property) and the
% name of the variable or variables to which it is assigned. Below a Signal
% whose name is 'input' is created and assigned to the variable
% `originSignal`.  Two values are posted to it, first a double, then a char
% array:

originSignal = net.origin('input'); % Create an origin signal
originSignal.Node.CurrValue % The current value is empty

post(originSignal, 21) % Post a new value to originSignal
originSignal.Node.CurrValue % The current value is now 21

post(originSignal, 'hello') % Post a new value to originSignal
originSignal.Node.CurrValue % The current value is now 'hello'

% You can see there are two names for this signal.  The string identifier
% ('input') is the Signal object's name, stored in the Name property:
disp(originSignal.Name)

% Any Signals derived from this will include this identifier in their Name
% property (an example will follow shortly).  The variable name
% 'originSignal' is simply a handle to the Signal object and can be changed
% or cleared without affecting the object it references(3).

% Although the value is stored in the Node's CurrValue field, it is not
% intended that you use this field directly.  The purpose of using a
% reactive network is that callbacks will access these values automatically
% if and when they change.  Accessing this property directly will most
% likely lead to unintended behaviour.
```

```
ans =

     []


ans =

    21


ans =

    'hello'

input
```

## Demonstration on sig.Signal/output() method

The output method is a useful function for understanding the relationship between signals. It simply displays a signal's output each time it takes a value. The output method returns an object of the class TidyHandle, which is like a normal listener handle, however when its lifecyle ends it will delete itself. What this means is that when the handle is no longer referenced anywhere (i.e. stored as a variable), the callback will no longer function.

```
net = sig.Net; % Create a new signals network
clc % Clear previous output for clarity

simpleSignal = net.origin('simpleSignal');
h = output(simpleSignal);
class(h)

simpleSignal.post(false) % Value printed to the command window
simpleSignal.post(true)

% The output method can't be used within an experiment definition function.
% It should instead be used only for playing around with Signals in the
% command prompt.
```

```
ans =

    'TidyHandle'

   0

   1
```

## Relationships between signals

Once you have one or more inputs to the network you can start to build a reactive network. Most of MATLAB's elementary operators work with Signals objects in the way you would expect, as demonstrated below. You may type ctrl + enter to run this entire secion at once...

```
net = sig.Net; % Create a new signals network
x = net.origin('x'); % Create an origin signal
a = 5; b = 2; c = 8; % Some constants to use in our equation
y = a*x^2 + b*x + c; % Define a quadratic relationship between x and y

% Let's call a little function that will show the relationship between our
% two signals.  The plot updates each time the two input Signals update:
ax = sig.plot(x,y,'b-');
xlim(ax, [-50 50]);

% Post some values to x and observe how the value of y changes
for i = -50:1:50
  pause(0.05)
  x.post(i)
end

% Note the axes labels display the Name property of the two signals.
```

## Mathematical expressions

Signals allows a good degree of clarity in defining methematical equations, particularly those where time is a direct or indirect variable

## Example 1: cos(x \* pi)

```
x = net.origin('x'); % Create an origin signal
y = cos(x * pi);
sig.timeplot(x, y, 'mode', [0 2]); % Plot each variable against time

for i = 0:0.1:10
  pause(0.05)
  x.post(i)
end
```

## Example 2: x -> degrees

Let's imagine you needed a Signal that showed the angle of its input between 0 and 360 degrees:

```
x = net.origin('x');
y = iff(x > 360, x - 360*floor(x/360), x); % More about conditionals later

ax = sig.plot(ax, x, y, 'b-'); % NB: Using the previous plot axes
xlim([0 1080]); ylim([0 360])

for i = 1:4:1080
  pause(0.005)
  x.post(i)
end
```

## Logical operations

Note that the short circuit operators && and are not implemented in Signals, always use & and | instead.

```
x = net.origin('x'); % Create an origin signal
bool = x >= 5 & x < 10;

ax = sig.plot(ax, x, bool, 'bx');
xlim(ax, [0 15]), ylim(ax, [-1 2])

for i = 1:15
  x.post(i)
end
```

## mod, floor, ceil

A simple example of using mod and floor natively with Signals:

```
x = net.origin('x'); % Create an origin signal

even = mod(floor(x), 2) == 0;
odd = ~even;

ax = sig.timeplot(x, even, odd, 'tWin', 1);
for i = 1:15
  x.post(i)
end
```

## Arrays

You can create numerical arrays and matricies with Signals in an intuitive way. NB: Whenever you perform an operation on one or more Signals objects, always expect a new Signals object to be returned. In the below example we create a 1x3 vector Signal, X, which is not an array of Signals but rather a Signal that carries a numrical array as its value:

```
x = net.origin('x');
X = [x 2*x 3]; % Create an array from signal x
X_sz = size(X); % Reports the size of object's underlying value

h = [output(X), output(X_sz)]; % Output the value and size of X

x.post(5)
```

```
     5    10     3

     1     3
```

## Matrix arithmatic

```
Xt = X';
Y = X.^3 ./ 2;

% For a full list see doc sig.Signal.  NB: Sometimes due to limitations of
% syntax, it's necessary to do away with syntactic sugar.  It is therefore
% worth remembering the basic functions, i.e. not(), plus(), times(), etc.
```

## (Take a moment to clear your workspace)

For the below sections to read more clearly, let's clear all these nodes and graphs we've created. You may ignore any text printed to the command window when clearing variables(4)

```
clear all; close all; clc
```

```
**net.delete**
Deleting net(0)
**net.delete**
Deleting net(1)
Unloading sqNetwork
Deleting net(2)
2 is not a valid network id
2 is not a valid network id
2 is not a valid network id
2 is not a valid network id
2 is not a valid network id
2 is not a valid network id
2 is not a valid network id
2 is not a valid network id
2 is not a valid network id
2 is not a valid network id
2 is not a valid network id
2 is not a valid network id
[...]
2 is not a valid network id
2 is not a valid network id
2 is not a valid network id
2 is not a valid network id
2 is not a valid network id
2 is not a valid network id
2 is not a valid network id
2 is not a valid network id
2 is not a valid network id
2 is not a valid network id
2 is not a valid network id
2 is not a valid network id
2 is not a valid network id
2 is not a valid network id
2 is not a valid network id
2 is not a valid network id
**net.delete**
2 is not a valid network id
```

## A note about Signals variables

Signals are objects that constantly update their values each time the Signals they depend on update. A Signal will not a take a value post-hoc after a newly defined Signal takes a value. Consider the following:

```
net = sig.Net;
x = net.origin('x');
x.post(5)

x.Node.CurrValue % 5
y = x^2;
y.Node.CurrValue % empty; does not evalute 5^2
x.post(3)
y.Node.CurrValue % 9

% In the context of a Signals Experiment, the experiment definition
% function is run once to set up all Signals, before any inputs are posted
% into the network.  More on this later.

% Likewise if you re-define a Signal, any previous Signals will continue
% using the old values and any future Signals will use the new values,
% regardless of whether the variable name is the same.  Remember that
% variable names are simply object handles so clearing or reassigning those
% variable names doesn't necessarily change the underlying object:
y = x^2;
a = y + 2;
y = x^3; % A new Signal object is assigned to the variable y
b = y + 2;

% Looking at the name of your Signals may help you here
% TODO add node digraph
y.Name
a.Name % *(x^3 + 2)
b.Name
```

```
ans =

     5


ans =

     []


ans =

     9


ans =

    'x^3'


ans =

    '(x^2 + 2)'


ans =

    '(x^3 + 2)'
```

## Signals can derive from multiple signals

Signals can be defined from any number of other signals, as well as by constants(5). Mathematically, Signals can be viewed as variables which, any time they take a new value, cause any dependent equations to be re-evaluated.

```
% Create some origin signals to post to
x = net.origin('x');
a = net.origin('a');
b = net.origin('b');
c = net.origin('c');

y = a*x^2 + b*x + c;

sig.timeplot(x, a, b, c, y);

x.post(1), pause(1)
a.post(pi), pause(1)
b.post(3), pause(1)
c.post(8), pause(1)

a.post(5)
```

## Complicated expressions

Below are some examples of more complex mathematical expressions that can be defined in Signals. Note that mapn simply maps the signal values through an arbitrary MATLAB function. On on this later.

## Example 1: Upper bound

Currently the following line won't work as Max does not yet work natively on Signals, however in the next version of Signals this will be implemented:

upperBound = max([abs(a), abs(b), abs(c)]) / abs(a) \* (1 + sqrt(5))/2;

```
upperBound = map([abs(a), abs(b), abs(c)], @max) / abs(a) * (1 + sqrt(5))/2;
disp(upperBound.Name)
```

## Example 2: Gabor

Let's reproduce the equation for generating a Gabor patch, i.e convolving a sinusoid with a 2D Gaussian function:

```
net = sig.Net;
xx = net.origin('xx');
yy = net.origin('yy');

% Orientation
theta = net.origin(char(hex2dec('03B8'))); % U+03B8 = Unicode Greek theta
% Standard deviation of Gaussian envelope
sigma = net.origin(char(hex2dec('03C3')));
% Wavelength
lambda = net.origin(char(hex2dec('03bb')));
% Phase
phi = net.origin(char(hex2dec('03C6')));

[X, Y] = xx.mapn(yy, @meshgrid);
X.Name = 'x'; Y.Name = 'y';
Xe = X.*cos(theta(1)) + Y.*sin(theta(1));
Ye = Y.*cos(theta(1)) - X.*sin(theta(1));
Xe.Name = 'x'''; Ye.Name = 'y''';
Xc = X.*cos(theta(2) - pi/2) + Y.*sin(theta(2) - pi/2);
gauss = map(-Xe.^2./(2*sigma(1)^2) + -Ye.^2./(2*sigma(2)^2), @exp);
% gauss = exp( -Xe.^2./(2*sigma(1)^2) + -Ye.^2./(2*sigma(2)^2) ); % Soon
grate = cos( 2*pi*Xc./lambda + phi );
G = gauss.*grate;

fprintf(['Gaussian equation: %s\n',...
  'Grating equation: %s\n',...
  'Convolved: %s\n'],...
  gauss.Name, grate.Name, G.Name)
```

```
[|a| |b| |c|].map(@max)/|a|*3.2361/2
Gaussian equation: (-x'.^2./2*σ(1)^2 + -y'.^2./2*σ(2)^2).map(@exp)
Grating equation: cos((6.2832*(x.*cos((θ(2) - 1.5708)) + y.*sin((θ(2) - 1.5708)))./λ + φ))
Convolved: (-x'.^2./2*σ(1)^2 + -y'.^2./2*σ(2)^2).map(@exp).*cos((6.2832*(x.*cos((θ(2) - 1.5708)) + y.*sin((θ(2) - 1.5708)))./λ + φ))
```

## Indexing

## Indexing into a Signal

Signals can be indexed as expected with brackets and the colon operator.

```
A = net.origin('A');
a = A(2); % index a and assign to b
B = A(5:end);

h = [output(A) output(a) output(B)];
A.post(1:10); % assign a vector of values from 1 to 10
% a.Node.CurrValue == 2
% B.Node.CurrValue == [5 6 7 8 9 10]
```

## Indexing with a Signal

Another Signal may be used to index another Signal:

```
i = net.origin('index'); % Define a new Signal
a = A(i);
h = [output(A) output(a) output(i)];

A.post(1:10);
i.post(5); % Both i and a = 5
A.post(10:20); % i = 5; a = 14
```

## selectFrom

The selectFrom method allows for indexing from a list of Signals whose values may be of different types. In some ways this is comparable to indexing to a cell array(6):

```
A = net.origin('A');
B = net.origin('B');
C = net.origin('C');

y = i.selectFrom(A, B, C);
h = output(y);
sig.timeplot(i, A, B, C, y);

A.post('helloSignal'), B.post([1 2 3]), C.post(pi)
pause(1.5)
i.post(2)
pause(1.5)
i.post(1)

% When the index is out of bounds the Signal simply doesn't update
i.post(4)
```

## indexOfFirst

The indexOfFirst method returns a Signal with the index of the first true predicate in a list of Signals. This has a similar functionality to find(arr, 1, 'first'):

```
idx = indexOfFirst(A > 5, B < 1, C == 5); % Better examples welcome!
```

```
     1     2     3     4     5     6     7     8     9    10

     2

     5     6     7     8     9    10

     1     2     3     4     5     6     7     8     9    10

     5

     5

    10    11    12    13    14    15    16    17    18    19    20

    14

     1     2     3

helloSignal
```

## Use more complex functions with map

It is not possible to call all functions with signals objects as inputs, and therefore expressions like fliplr(A), where A is a Signal will cause an error:

```
A = net.origin('A'); % Create an origin signal
B = fliplr(A); % Conversion to logical from sig.node.Signal is not possible

% Instead we can use the function map, which will call a given function
% with a Signal's current value as its input each time that signal takes a
% new value
B = A.map(@fliplr);
A.post(eye(5)); % You can guess the value of B
```

## Anonymous functions

Sometimes your Signal must be in a different positional argument. For this we simply create an anonymous function and use that. See the MATLAB doumentation for more information on how to use these.

```
delta = A.map(@(A)diff(A,1,2)); % Take 1st order difference over 2nd dimension
a = A.map(@(A)sum(A,2)); % Take sum over 2nd dimension
A.post(magic(3));

delta.Node.CurrValue
a.Node.CurrValue
```

## Example 2: a complex counter

In the following example we have a signal, responseType, that may be an element of [-1 0 1]. A response type of 0 means the trial timed out before the subject gave a response. Let's say we want to count the number of times in a row a trial timeout occurs:

```
% First we store the responseType values for up to the last 1000 trials
timeOuts = responseType.bufferUpTo(1000);
% Then count the number of recent timeouts that occured in a row using an
% anonymous function that works on this array with find and sum:
timeOutCount = timeOuts.map(@(x) sum(x(find([1 x~=0],1,'last'):end) == 0));
```

## fun.partial

fun.partial can be a convenient way to map a function when there are a number of constant positional args required before your signal.

```
% The line below returns a function handle which will call sprintf with the
% first argument as '%.1f%%':
f = fun.partial(@sprintf, '%.1f%%');
class(f)
y = x.map(f);
% y = x.map(@(num)sprintf('%.1f%%',num)); % This line is equivalent

y1 = map(100-x, f);
h = [output(y) output(y1)];

x.post(58.4)
```

## Sampling with map

Map is particularly handy for 'sampling' functions, that is, for generating a new index, value, or whatever, each time an event occurs. Below we derive a signal, side, that each time `newTrial` updates takes a new value from [-1 1] (technically 0 is also possible!). Note that the anonymous function discards the value of newTrial. We're only using it as a way to trigger the evaluation of this function.

```
newTrial = net.origin('new trial');
side = newTrial.map(@(~) sign(rand-0.5));

h = output(side);
newTrial.post(true), newTrial.post(true), newTrial.post(true)
```

## Mapping constants

Sometimes you want to derive a Signal whose value is always constant but nevertheless updates depending on another Signal, thus acting as a trigger for something. This can be achieved by using a constant value instead of a function in map. For example below we produce a signal that is always mapped to true and updates whenever its dependent value updates.

Here we also introduce two more methods: at and then. These functions sample the value of a Signal each time another Signal updates. What's more, the Signal that updates must evaluated to true:

```
x = net.origin('x');
% sample = n.map(@(~)double(rand > 0.5)); % Samples zero or one
y = net.origin('y');
y = x.at(x > 5 & x < 10);

ax = sig.timeplot(x,y);
ylim(ax,[0 15])
% Run this loop manually
for i = 1:15
  pause(0.3)
  x.post(i)
end

% This may also be achieved with keepWhen:
y = x.keepWhen(x > 5 & x < 10);

% NB c.f. with at, then and skipRepeats
y = net.origin('y');
updatedAndTrue = y.at(updated);
% updatedAndTrue = updated.then(y); % then is the same as at but with the
% inputs in a reverse order.  This is simply to make things more
% self-documenting.
updatedAndTrue = x.at(y > 3);
h = output(updatedAndTrue);

% Sometimes you want to sample one signal at the time that another updates,
% regardless of whether it's value evaluates true.  For this we can simply
% map that signal to true:
z = x.at(y);
zi = x.at(y.map(true));

ax = sig.timeplot(x, y, z, zi, 'mode', [0,1,0,0], 'tWin', 10);
% Run this loop manually
for i = 1:50
  x.post(i)
  pause(0.3)
  if rand > .5
    y.post(rand > .5)
  end
end

% Note that if the input arg is a value rather than a function handle, it
% is truely constant, even if it's the output of a function:
c = x.map(rand);
rnd = x.map(@(~)rand);
% The tilda here means that the value of Signal x is ignored, instead of
% being assigned to a temporary variable or being mapped into the functon
% rand, thus rand is called with no arguments.
```

```
Conversion to logical from sig.node.Signal is not possible.

Error in matlab.internal.builtinhelper.flip (line 37)
if dimsize <= 1 && (isreal(x) || ~isnumeric(x))

Error in fliplr (line 13)
x = flip(x,2);

Error in SignalsPrimer (line 388)
B = fliplr(A); % Conversion to logical from sig.node.Signal is not possible
```

## Map multiple Signals through a function with mapn

mapn takes any number of inputs where the last argument is the function that the other arguments are mapped to. The arguments may be any combination of Signals and normal data types. It's important to note that the below 'dot notation' only works if the first input is a Signal, otherwise you must use the traditional syntax e.g. mapn(5, A, @f)

```
B = A.mapn(n, 1, @repmat); % repmat(A,n,1)

% NB: Map will only assign the first output argument of the function to the
% resulting Signal.  Mapping to a variable number of output Signals will be
% possible in the next version of Signals:
a = net.origin('a');
[b,n]  = a.mapn(@shiftdim); % b is 3-by-1-by-2 and n is 2.
c = b.mapn(-n, @shiftdim);  % c == a.
d = a.mapn(3, @shiftdim);   % d is 1-by-2-by-1-by-1-by-3.

a.post(rand(1,1,3,1,2))
```

## More complex conditionals

Above we saw how logical operations work with Signals. These can also be used in conditional statements that alter the value or operation on a given Signal. For example, to construct something similar to an if/else statement, we can use the iff method:

```
x = net.origin('x');
y = iff(x.map(@ischar), x.map(@str2num), x);

% In order to construct if/elseif statements we use the cond method, where
% the input arguments are predicate-value pairs, for example:
y = cond( ...
  x < 5, a, ... % If x < 5, y = a
  x > 10, b); % elseif x > 10, y = b

% As with all Signals, the condition statement is re-evaluated when any of
% its inputs update.  Any input may be a Signal or otherwise, and if no
% predicate evaluates as true then the resulting Signal does not update.

% Likewise, the condition statement will terminate if any of the source
% Signals of a particular pred-value pair do not yet have values.  Also, in
% the same way as a traditional if-elseif statement, each predicate is only
% evaluated so long as the previous one was false.  For this reason, the
% order of pred-value pairs is particularly important. Below we use true as
% the last predicate to ensure that the resulting Signal always has a
% value.
y = cond(...
  x > 0 & x < 5, a, ...
  x > 5, b, ...
  true, c);
```

## Demonstration of scan

Scan is a very powerful method that allows one to map a signal's current value and its previous value through a function. This allows one to define signals that have some sort of history to them. This is similar to the fold or reduce functions found in other functional programming applications.

Below we take the value of x and return a value that is the accumulation of x by using scan with the function plus. The third argument to scan here is the initial, or 'seed', value. Because the seed is zero, the first time x takes a value, scan maps zero and the value of x respectively to the plus function and assigns the output to Signal y. The second time x updates, scan maps the current value of y, our accumulated value, and the new value of x to the plus function.

```
net = sig.Net;
x = net.origin('x');

y = x.scan(@plus, 0); % plus(y, x)

sig.timeplot(x, y, 'tWin', 0.5);
for i = 1:10
  x.post(1)
end
```

## The seed value may be a signal

As with other Signals methods, any of the inputs except the function handle may be a Signal. This is particularly useful as the seed value can act as a reset of the accumulator as demonstrated below.

```
x = net.origin('x');
seed = net.origin('seed');
y = x.scan(@plus, seed);

sig.timeplot(x, y, seed, 'tWin', 1, 'mode', [0 0 1])
seed.post(0); % Initialize seed with value

for i = 1:10
  if i == 5
    seed.post(0)
  end
  x.post(1)
end
```

## Growing an array with scan

You can grow arrays with scan by using the vertcat or horzcat functions. The accumulated/seed value is always the first argument to the function, however, you can of course assign them to temporary variables beforehand as below.

```
x = net.origin('x');
f = @(acc,itm) [itm acc]; % Prepend char to array
y = x.scan(f, '!');
h = y.output();
for i = 1:10
  x.post('>')
end

% Below, each time the Signal 'correct' takes a new, truthy value, the
% Signal 'trialSide' updates and scan will call the function horzcat with
% the values of hist and trialSide like so: horzcat(hist, trialSide), which
% is syntactically equivalent to [hist trialSide]
hist = trialSide.at(correct).scan(@horzcat);
```

## Introducing extra parameters

Some functions require any number of extra inputs. A function can be called with these extra parameters by defining them after the 'pars' name argument. All arguments after the input 'pars' are treated as extra parameters to map to the function when either the input or seed Signals update.

```
x = net.origin('x');
seed = net.origin('seed');
seed.post('!'); % Initialize seed with value
f = @(acc,itm,p1) [itm p1 acc]; % Prepend char to array
y = x.scan(f, seed, 'pars', '.'); % Pars may be signals or no
h = y.output();

x.post('>')
```

## Parameters may be Signals

Below we use the scan function to build a character array with strjoin...

```
x = net.origin('x');
seed = net.origin('seed');
seed.post('0'); % Initialize seed with value
f = @(acc,itm,delim) strjoin({acc, itm}, delim); % Prepend char to array
% f = @(acc,itm) strjoin({acc, itm}, ' + ');
y = x.scan(f, seed, 'pars', ' + '); % Pars may be signals or any other data type
h = y.output();

x.post('1')
x.post('12')
x.post('18')
x.post('5')
x.post('8')
```

## When pars take new values, accumulator function is not called!

Unlike most other Signals, parameters Signals can take new values without causing the function to be called. Below we define a Signal, p, into which we can post the delimiter for the function strjoin.

```
x = net.origin('x');
seed = net.origin('seed');
p = net.origin('delimiter');
seed.post('0'); % Initialize seed with value
p.post(' + '); % Initialize seed with value
f = @(acc,itm,delim) strjoin({acc, itm}, delim); % Prepend char to array
y = x.scan(f, seed, 'pars', p); % Pars may be signals or any other data type
h = y.output();

x.post('1')
x.post('12')
p.post(' - '); % Updating p doesn't affect scan
x.post('18')
x.post('5')
p.post(' * ');
x.post('8')
```

## Scan can call any number of functions at the same time

Scan can call any number of functions each time one of the input Signals updates. Only the functions whose named inputs update will be called. Remember that all functions called by scan have the accumulated value as their first input argument, followed by the input Signal and any parameters following the 'pars' input.

```
x = net.origin('x');
y = net.origin('y');
z = net.origin('z');
seed = net.origin('seed');
seed.post(0); % Initialize seed with value
f1 = @plus; %
f2 = @minus; %
f3 = @times; %
v = scan(x, f1, y, f2, z, f3, seed); % Pars may be signals or any other data type
h = v.output();

x.post(1) % 1
x.post(1) % 2
x.post(1) % 3

y.post(1) % 2
y.post(1) % 1

z.post(2) % 2
z.post(2) % 4
z.post(2) % 8
```

## Timing in signals

Most experiments require things to occur at specific times. This can be achieved by keeping a timing signal that has a clock value posted to it periodically. In the following example, we will create a 'time' signal that takes the value returned by 'now' every second. We achieve this with a fixed-rate timer. In the context of a Signals Experiment, the time signal has a time in seconds from the experiment start posted every iteration of a while loop. Read through the below section then run it as a block by pressing ctrl + enter.

```
net = sig.Net; % Create a new signals network
clc % Clear previous output for clarity
time = net.origin('t'); % Create a time signal
% NB: The onValue method is very similar to the output method, but allows
% you to define any callback function to be called each time the signal
% takes a value (so long as the handle is still around).  Here we are using
% it to display the formatted value of our 't' signal.  Again, the output
% and onValue methods are not suitable for use within an experiment as the
% handle is deleted.
handle = time.onValue(@(t)fprintf('%.3f sec\n', t*10e4));

t0 = now; % Record current time
% Create a timer that posts the time since t0 to the 'time' signal, at a
% given rate given by 'frequency'.
frequency = 1; % Update the timer every second
tmr = timer('TimerFcn', @(~,~)post(time, now-t0),...
    'ExecutionMode', 'fixedrate', 'Period', 1/frequency);
start(tmr) % Start the timer
disp('Timer started')
% ...Because of the output method, we are seeing the value of the time
% signal displayed every second
pause(3)
```

## Now let's increase the frequency to 10 ms...

```
stop(tmr) % Stop the timer
frequency = 1e-2; % Frequency now 10x higher
disp('Let''s increase the timer frequency to 10 times per second...')
set(tmr, 'Period', frequency)
pause(1) % Ready... steady... go!
start(tmr)
pause(3) % ...
```

## When we clear the handle, the value is no longer displayed

```
disp('Clearing the output TidyHandle')
clear handle
pause(1) % ...The values of the 'time' Signal are no longer displayed
```

## Due to the timer, the value of 'time' continues to update

```
fprintf('%.3f sec\n', time.Node.CurrValue*10e4)
pause(1)% ...
fprintf('%.3f sec\n', time.Node.CurrValue*10e4)
pause(1)
```

## When the timer is stopped, the value of 'time' is no longer updated

```
disp('Stopping timer');
stop(tmr)
pause(1)% ...
fprintf('%.3f sec\n', time.Node.CurrValue*10e4)
pause(1)% ...
fprintf('%.3f sec\n', time.Node.CurrValue*10e4)
pause(1)% ...
% Let's clear the variables
delete(tmr); clear tmr frequency t0 time
```

## Filtering

Signals become very useful when you want to define a relationship between two events in time. In addition to viewing Signals as values that change over time, they can also be treated as a series of discrete values or events used to gate and trigger other Signals. We saw this earlier with the map method.

```
net = sig.Net;
time = net.origin('t'); % Create a time signal
t0 = GetSecs; % Record current time
frequency = 0.5;
tmr = timer('TimerFcn', @(~,~)post(time, GetSecs-t0),...
    'ExecutionMode', 'fixedrate', 'Period', 1/frequency);

gate = floor(time/5);

ax = sig.timeplot(time, gate, skipRepeats(gate), gate.map(true), ...
  'tWin', 20, 'mode', [0 0 0 1]);
% set(ax, 'ylim', [0 30])
start(tmr) % Start the timer
```

## Example 2: keepWhen

```
theta = sin(time);
sig.timeplot(time, theta, theta.keepWhen(theta > 0), 'mode', [0 2 2], 'tWin', 20);
```

## Example 3: setTrigger & skipRepeats

```
x = -1 + delta(time);
y = 2*x.^2 - x.^3;
y.Name = 'y'; % Change the name of this Signal to something more readable
arm = skipRepeats(y < 1);
trig = arm.setTrigger(y > 1);
% Reset timer
% time = time - GetSecs;
sig.timeplot(x, arm, y, trig, 'tWin', 60);%, 'mode', [0 2 3 2])
```

## Example 4: to & lag

```
a = mod(floor(time),3) == 0;
b = a.lag(1);
c = a.to(b);
sig.timeplot(time, a, b, c);
% while strcmp(tmr.Running, 'on')
% at, then, setTrigger, skipRepeats, keepWhen
```

## Timing 2 - Scheduling

The net object contains an attribute called Schedule which stores a structure of node ids and their due time. Each time the schedule is run using the method runSchedule, the nodes whose TODO

```
net = sig.Net; % Create network
frequency = 10e-2;
tmr = timer('TimerFcn', @(~,~)net.runSchedule,...
    'ExecutionMode', 'fixedrate', 'Period', frequency);
start(tmr) % Run schedule every 10 ms
s = net.origin('input'); % Input signal
delayedSig = s.delay(5); % New signal delayed by 5 sec
h = output(delayedSig); % Let's output its value
h(2) = delayedSig.onValue(@(~)toc); tic
delayedPost(s, pi, 5) % Post to input signal also delayed by 5 sec
disp('Delayed post of pi to input signal (5 seconds)')
% After creating a delayed post, an entry was added to the schedule
disp('Contents of Schedule: '); disp(net.Schedule)
fprintf('Node id %s corresponds to ''%s'' signal\n\n', num2str(s.Node.Id), s.Node.Name)
% ...
disp('... 5 seconds later...'); pause(5.1)
% ...
% ... a second entry was added to the schedule, this time for 'delayedSig'.
% This was added to the schedule as soon as the value of pi was posted to
% our 'input' signal.
disp('Contents of Schedule: '); disp(net.Schedule)
fprintf('Node id %s corresponds to ''%s'' signal\n\n',...
    num2str(net.Schedule.nodeid), delayedSig.Node.Name)
% ...
disp('... another 5 seconds later...'); pause(5.1)
% ...
% 3.14
stop(tmr); delete(tmr); clear tmr s frequency h delayedSig
```

## TODO SetEpochTrigger

## Demonstration of subscriptable signals

If you have a signal that holds a value that is either an object or a struct, you can make that value subscriptable with the method below:

```
net = sig.Net;
s = struct('A', now, 'B', rand);
x = net.origin('struct');

% The below would not work:
x.post(s); A = x.A;
% >> No appropriate method, property, or field 'A' for class
% 'sig.node.OriginSignal'.

% Deriving a subscriptable Signal allows us to subscript:
x_sub = x.subscriptable(); % TODO Add note about subsref
a = x_sub.A;
h = output(a);
% We must repost our structure as there are new Signals which won't have
% had the value propergated to them:
x.post(s);

% Note that with subscriptable Signals the returned value is a new Signal,
% whose value will be updated each time any field is updated in the
% source Signal.  You can subscript such a Signal even if the field doesn't
% exist:
c = x_sub.C;
h = output(c);
x.post(s);
% Note that c never updates as the underlying value in x has no field 'C'.
```

## You can not post to subscriptable Signals, nor assign values to them

Even if the Signal is derived from an origin Signal:

```
x_sub.post(s); % Returns a Signal for the subscript of a 'post' field.
post(x_sub, s);
% >> Undefined function 'post' for input arguments of type
% 'sig.node.SubscriptableSignal'.

% Instead, we use another class of Signal called a Subscriptable Origin
% Signal.  With these Signals we do not post structures, instead one can
% assign individual values to each subscript, which may themselves be
% Signals or otherwise.

x_sub = net.subscriptableOrigin('x');
a = x_sub.A; b = x_sub.B; %c = scan(x_sub.A, @plus, []);
h = [output(a), output(b)] %, output(c)];
x_sub.A = 5;
x_sub.B = x;
x_sub.A = 10;
% TODO Add timeplot
% To repeat, using the post method on any subscripatble Signal, origin or
% otherwise will not have the desired effect.  Instead, simply assign your
% values directly to a subscripatble origin Signal.

% Note again that all Signals update each time any of the subscriptable origin
% Signal's subscripts update.  Thus if we assign a new value to x_sub.B,
% x_sub.A will update but with the same value it had before.

% If you wish to return a plain structure each time a subscriptable Signal
% updates, use the flattenStruct method:
flat = x_sub.flattenStruct();
h = output(flat);
x_sub.A = 10;
x_sub.B = pi;
x_sub.C = true;

% Cache subscripts caches subscripts (not subassigns)
```

## Helpful methods

There are a few other helpful methods implemented in Signals. Let's look briefly at them:

## delta

```
x = net.origin('x'); % Create an origin signal
a = 5; b = 2; c = 8; % Some constants to use in our equation
y = a*x^2 + b*x + c; % Define a quadratic relationship between x and y

sig.timeplot(x,y,y.delta,'mode',[0 2 0],'tWin',6);

for i = -50:2:50
  pause(0.01)
  x.post(i)
end
```

## lag

## buffer

## bufferUpTo

## Demonstration of sig.Signal/log() method

Sometimes you want the values of a signal to be logged and timestamped. The log method returns a signal that carries a structure with the fields 'time' and 'value'. Log takes two inputs: the signal to be logged and an optional clock function to use for the timestamps. The default clock function is GetSecs, a PsychToolbox MEX function that returns the most reliable system time available.

```
net = sig.Net; % Create our network
simpleSignal = net.origin('simpleSignal'); % Create a simple signal to log
loggingSignal = simpleSignal.log(@now); % Log that signal using MATLAB's now function
loggingSignal.onValue(@(a)disp(toStr(a))); % Each time our loggingSignal takes a new value, let's display it

simpleSignal.post(3)
pause(1); fprintf('\n\n')

simpleSignal.post(8)
pause(1); fprintf('\n\n')

simpleSignal.post(false)
pause(1); fprintf('\n\n')

simpleSignal.post('foo')
```

## Logging signals in a registry

In order to simplify things, one can create a registry which will hold the logs of all signals added to it. When the experiment is over, the registry can return all the logged values in the timestampes optionally offset to another clock. This can be useful for returning values in seconds since the start of the experiment

```
net = sig.Net; % Create our network
t0 = now; % Let's use this as our example reference time
events = sig.Registry(@now); % Create our registy
simpleSignal = net.origin('simpleSignal'); % Create a simple signal to log
events.signalA = simpleSignal^2; % Log a new signal that takes the second power of the input signal
events.signalB = simpleSignal.lag(2); % Log another signal that takes the last but one value of the input signal
simpleSignal.post(3) % Post some values to the input signal
simpleSignal.post(3)
simpleSignal.post(8)

s = logs(events, t0); % Return our logged signals as a structure
disp(s)
```

## Visual stimuli

```
[t, setgraphic] = sig.playgroundPTB;
grating = vis.grating(t);    % we want a gabor grating patch
grating.phase = 2*pi*t*3; % with it's phase cycling at 3Hz
grating.show = true;

elements = StructRef;
elements.grating = grating;

setgraphic(elements);
```

## Notes

1. The sig.Net class itself does not store the nodes in its properties, however the underlying mexnet does. This network is created by calling the MEX function createNetwork. New nodes are created by calling the MEX function addNode. This is done for you in the sig.Net and sig.node.Node class constructors.

```
% 2. Two such examples of visualization functions are introduced later,
% |sig.plot| and |sig.timeplot|.

% 3. Signals objects that are entirely out of scope are cleaned up by
% MATLAB and the underlying C code.  That is, if a Signal is created,
% assigned to a variable, and that variable is cleared then the underlying
% node is deleted if there exist no dependent Signals:
net = sig.Net;
x = net.origin('orphan');
networkInfo(net.Id) % Net with 1/4000 active nodes
clear x
networkInfo(net.Id) % Net with 0/4000 active nodes

% If the Signal is used by another node that is still in scope, then it
% will not be cleaned up:
x = net.origin('x');
y = x + 2; % y depends of two nodes: 'x' and '2' (a root node)
networkInfo(net.Id) % Net with 3/4000 active nodes
clear x % After clearing the handle 'x', the node is still in the network
networkInfo(net.Id) % Net with 3/4000 active nodes
% The node still exists because another handle to it is stored in the
% Inputs property of the node 'y':
str = sprintf('Inputs to y: %s', strjoin(mapToCell(@(n)n.Name, [y.Node.DisplayInputs]), ', '));
disp(str)
disp(['y.Node.DisplayInputs(1) is a ' class(y.Node.DisplayInputs(1))])

% 4. The command window message '**net.delete**' simply indicates that a
% Signals network has been deleted, most likely as a result of a net object
% being cleared from the workspace.  The message '0 is not a valid network
% id' is nothing to worry about.  It is simply a result of an over-zealous
% cleanup proceedure in the underlying MEX code.  In future versions this
% will only show up when debugging.

% 5. Note that constants are in fact made into signals using the rootNode
% method.  These are nodes that only ever have one value.  There are often
% more nodes in a network than you might expect, for example the following
% line indicates there are at least 4 nodes in the network:
x = mod(floor(x), 1*2)
% These would be x, 2 (a root node), floor(x) and mod(floor(x), 2)

% 6. It should be noted here that you are responsible for handling
% potential problems that may arise from a Signal changing data type:
y = x*5;
x.post(2) % y = 10
x.post({'bad'}) % Undefined operator '*' for input arguments of type 'cell'
% Within a Signals Experiment this rarely is a problem as parameters may
% not change type, although you may still encounter issues, for example the
% below signal `evts.newTrial` holds the value `true` which must be
% typecast to an int or float before being used with randsample:
side = evts.newTrial.map(@(k)randsample([-1 1], int32(k)));
% The below line demonstrates how a signal can change type:
s = merge(str, int, mat);

% #. Rule exceptions: merge and scan pars There are only two exceptions to
% this.
%
% 1. merge - a merge signal will take the value of the last updated input
% signal, even if not all of the inputs have taken a value.  To only take
% values once all are updated, use the at/then methods:
s = merge(a, b, c).at(map([a b c], true)); % map(true) for if a, b, c = 0

% 2. scan - any signals passed into scan after the 'pars' named parameter
% do not cause the scan function to be re-evaluated when they update.  See
% section on scan above for more info.
s = a.scan(f, [], 'pars', b, c); % b and c values used in f when a updates
```

## Etc.

Author: Miles Wells

v1.1.0

```
%#ok<*NOPTS,*CLALL,*NASGU>
```

Published with MATLAB® R2018b
