# Supplementary material for: Rigbox: An Open-Source Toolbox for Probing Neurons and Behavior
Source: eNeuro. 2020 Jul 9;7(4):ENEURO.0406-19.2020. doi: 10.1523/ENEURO.0406-19.2020 (PMC7363478; doi:10.1523/ENEURO.0406-19.2020)
Supplement: Extended Data 1 — We recommend looking at and downloading the code directly from the github repository, at https://github.com/cortex-lab/Rigbox. Download Extended Data 1, ZIP file. [file enu-eN-OTM-0406-19-s02.zip › Rigbox-master-2020-05-18/Rigbox-master/docs/html/Timeline.html]

Timeline 

# Timeline

Timeline manages the acquisition and generation of experimental timing data using a NI-DAQ. The main timing signal, 'chrono', is a digital square wave that flips each time a new chunk of data is available from the DAQ. A callback function to this flip event collects the DAQ timestamp of the scan where each flip occured. The difference between this timestamp and the system time recorded when the flip command was given is recorded as the offset time and can be used to unify all timestamps across computers during an experiment. Thus, all event timestamps across all computers for a given experiment are recorded in times relative to the DAQ's clock. These timestamps can be interchanged with MATLAB and system times during an experiment. Timeline can acquire any number of hardware events and record their values with respect to this offset; for example, Timeline can use a photodiode to record the times at which the screen updates.

NB: Not all uncommented lines will run without error, particularly when a specific hardware configuration is required. Always read the preceeding text before running each line.

## Contents

- Using Timeline
- Using settings from a previous experiment
- Inputs
- Activating Inputs
- Dealing with ghosting
- Wiring information
- Outputs
- Acquire Live
- Output Clock
- Acquisitions
- Live plotting
- Clocks
- Notes
- Etc.

## Using Timeline

For more details on setting up Timeline, see hardware\_config:

```
opentoline(fullfile(getOr(dat.paths,'rigbox'), ...
  'docs', 'setup', 'hardware_config.m'), 58)
```

**NB** : Not all uncommented lines will run without error, particularly when a specific hardware configuration is required. Always read the preceeding text before running each line.

```
% Let's set up a timeline object:
timeline = hw.Timeline;
```

## Using settings from a previous experiment

To set up a new timeline object using setting from a previous experiment, call the constructor with the variable saved in \*\_Timeline.mat for that experiment:

```
ref = dat.constructExpRef('example', now - 10, 2); % Example experiment
hwPath = dat.expFilePath(ref, 'timeline', 'master'); % Path to timeline MAT
timeline = hw.Timeline(loadVar(hwPath, 'hw')); % Load and instantiate
```

A JSON file is also saved after each experiment, currently with the name TimelineHW.json:

```
hwPath = fullfile(dat.expPath(ref,'main','master'), 'TimelineHW.json');
timeline = hw.Timeline(jsondecode(fileread(hwPath))); % Load from JSON copy
```

## Inputs

The Inputs property contains a structure for configuring which channels to aquire data from.

```
timeline.Inputs
```

```
ans =
```

```
   struct with fields:
```

```
               name: 'chrono'
        arrayColumn: -1
       daqChannelID: 'ai0'
        measurement: 'Voltage'
     terminalConfig: 'SingleEnded'
          axesScale: 1
```

You can add a channel by adding directly to the Inputs property or by using addInput:

```
name = 'rotary encoder'; % Name of the device or signal you're acquiring
channel = 'ctr0'; % The channel the device is connected to
measurement = 'Position'; % The measurement type, i.e. 'volts', 'pos', 'edge'
timeline.addInput(name, channel, measurement)
```

Timeline input 'rotary encoder' successfully added.

Extra parameters include terminal configuration (Differential or SingleEnded, otherwise the channel default is used), axes scale, and a flag indicating whether to use the input (true by default):

```
timeline.addInput('lick detecter', 'ctr1', 'EdgeCount', [], 2, false)
```

The axes scale (set to 2 above) sets the vertical plot scale when the Timeline property LivePlot is set to true (more on this later).

## Activating Inputs

The 'UseInputs' property contains a cellstring of the input names to be acquired. This allows one to set up mutiple inputs that aren't necessarily acquired every experiment. The 'use' flag of addInput can set whether an input is added to UseInputs upon adding. The default is true.

```
timeline.UseInputs % 'lick detecter' missing as we set the use arg to false above
% To activate an input again simply add it to UseInputs again:
timeline.UseInputs{end+1} = 'lick detecter';
```

**Note** that the order of channels in the UseInputs array determines the order in which the channels are added to the DAQ session and thus the order in which they are scanned during acquisition (see next section).

## Dealing with ghosting

Multiplexing NI DAQ boards (as opposed simultaneously sampling boards) can be susceptible to 'ghosting', whereby weak differential channels (those with a small current source) are corrupted by neighbouring channels. A solution to this is to isolate the channel of interest by adding one dummy channel either side of it. This is a page on NI website explaining how to avoid ghosting: https://knowledge.ni.com/KnowledgeArticleDetails?id=kA00Z0000019KzzSAE

For instance consider that we want to acquire the p.d. of a passive piezo:

```
timeline.addInput('piezoLickDetector', 'ai5', 'Volts', 'Differential',1,0)
% Now we can isolate this channel to prevent bleed-through by adding a
% couple of 'dummy' channels that are scanned before and after our channel:
timeline.addInput('dummy1', 'ai4', 'Volts',[],1,0)
timeline.addInput('dummy2', 'ai12', 'Volts',[],1,0)

% Add them to UseInputs in the following order:
use = [timeline.UseInputs {'dummy1', 'piezoLickDetector', 'dummy2'}]
timeline.UseInputs = use;
```

**NB** : Channel names beginning with the word 'dummy' are not extracted as ALF files . For more info see alyx-matlab:

```
open(fullfile(getOr(dat.paths,'rigbox'), ...
  'alyx-matlab', 'docs', 'AlyxMatlabPrimer.m'))
web('https://github.com/cortex-lab/ALF')
```

## Wiring information

Once an input is added you can view the wiring information with the wiringInfo method:

```
name = 'rotary encoder';
timeline.wiringInfo(name)
```

Connect rotary encoder to terminal ctr0 of the DAQ

## Outputs

Timeline outputs are for setting up pulses for triggering external hadware acquisition devices. The main output class for timeline is hw.TLOutput

```
doc hw.TLOutput

% An array of configured outputs are stored in the Outputs property of
% timeline:
timeline.Outputs

% By default there is one output, chrono.  This is the generator for the
% clocking pulse that Timeline itself uses and therefore is required.
%   _   _   _   _   _   _   _
% _| |_| |_| |_| |_| |_| |_| |__
% ^ start()                  ^ stop()

% All output objects have Enable and Verbose switches.  To disable an
% output for a particular experiment, simply set the Enable to 'off'
% (equivalent to UseInputs for the inputs):
timeline.Outputs(1).Enable = false;

% The Verbose switch when 'on' will print extra information to the command
% window during the experiment:
timeline.Outputs(1).Verbose = true;
```

## Acquire Live

```
% Let's add another output:
timeline.Outputs(2) = hw.TLOutputAcqLive;

% The Acquire Live output by default outputs HIGH for the entire time an
% experiment is running (that is, after calling HW.TIMELINE/START).  When
% the session ends (by calling HW.TIMELINE/STOP) the signal goes LOW.  This
% can be used to trigger imaging software to begin acquisition:
%   ____________..._____________
% _|                            |_
% ^ start()                     ^ stop()

% As with the main Timeline constructor, the outputs may be called with the
% timeline hardware struct from a previous experiment.
timeline.Outputs(2).Name = 'Instra-Triggar'; % Can be anything
timeline.Outputs(2).DaqChannelID = 'PFI4'; % Set the channel to output on

% The toStr method will print the object's information:
timeline.Outputs(2).toStr

% By setting the PulseDuration property to a value < Inf turns this into a
% pulse at the beginning and end of the experiment:
%   _                         _
% _| |__________...__________| |_
% ^ start()                  ^ stop()
```

## Output Clock

```
timeline.Outputs(end+1) = hw.TLOutputClock;

% The output clock object will produce a regular pulse troughout the
% experiment, useful for triggaring frame acquisitions of a camera:
%   _      _        _      _
% _| |____| |__..._| |____| |___
% ^ start()                  ^ stop()

% For more info on the Frequency and DutyCycle properties, see their
% corresponding doc pages:
web(fullfile(matlabroot,'help','daq','ref','dutycycle.html'))
web(fullfile(matlabroot,'help','daq','ref','frequency.html'))

% Delays can be added to these output classes using the InitialDelay
% property:
timeline.Outputs(end).InitialDelay = 3;
%            _      _        _      _
% __________| |____| |__..._| |____| |___
% ^ start()                          ^ stop()

% For custom output signals that are not configurable with the current
% TLOutput classes, simply create a new subclass with your desired
% implementation.
```

## Acquisitions

Timeline may be run automatically on the stimulus computer, or on a separate computer that's triggred by the stimulus computer via UDPs (for more info see using\_services):

```
% Open the 'Using Services' script
open(fullfile(getOr(dat.paths,'rigbox'), 'docs', 'using_services.m'))

% You can also start Timeline manually from the command prompt with
% HW.TIMELINE/START.  An experiment reference string is required:
ref = dat.newExp('default'); % Create an experiment for subject 'default'
timeline.start(ref, Alyx('','')) % Start acquisition
timeline.IsRunning % true

% In order to register Timeline file to the alyx database, an alyx object
% may also be passed to timeline:
ai = Alyx('',''); % Not logged in in this example
ref = ai.newExp('default') % Note we create the experiment with ALYX/NEWEXP
timeline.start(ref, ai)
% For more info see alyx-matlab:
open(fullfile(getOr(dat.paths,'rigbox'), ...
  'alyx-matlab', 'docs', 'AlyxMatlabPrimer.m'))

% Once timeline is started, you can log software events using RECORD:
name = 'udp'; % Imagine logged each time a UDP is received
data = 'coordinates: 12.4, 32.0, 23.9'; % Some data to record
timeline.record(name, data)
% This data will be recorded in saved data structure

% To stop Timeline, call the STOP method:
timeline.stop()

% The output channels are immediately stopped, followed by the input
% channel acquisition after the delay set by the StopDelay property.  The
% minimum delay is 2 seconds in order to allow time for the final samples
% to be collected. The data are then saved to file.
hwPath = dat.expFilePath(ref, 'timeline', 'master');
hw = loadVar(hwPath, 'hw'); % Load our data
% Note the 'udpTimes', 'udpEvents' and 'udpCount' fields from above

% If the WriteBufferToDisk switch is set to 'on', the data are saved to
% binary files during acquisition.  This ensures that data aren't lost in
% the even of MATLAB crashes.
timeline.WriteBufferToDisk = true; % Save data as they're collected
```

## Live plotting

During acquisition you can plot the inputs live by setting the 'LivePlot' property to true / 'on'. The default plot figure position(1) can be set using the 'FigureScale' property. The values are in normalized units and is set as fullscreen by default. **NB:** This must be set before calling start.

```
timeline.LivePlot = 'on'; % Plot input data during acquisition
timeline.FigureScale = [0 0 0.5 1]; % Take up half screen width and full height
```

The axes scale for individual inputs can be set be changing the 'axesScale' of the Inputs struct. This field controls the vertical scaling of each trace (multiplicative) and can be set when adding an input with the addInput method (see Inputs section). Below we change it manually:

```
I = strcmp('rotary encoder', {timeline.Inputs.name})
timeline.Inputs(I).axesScale = 2; % 2x vertical scaling
```

## Clocks

```
doc hw.Clock

% While timeline is running, timestampts can be returned using the TIME
% method:
timeline.time()
% If called with false, a system timestamp is returned when timeline is not
% running:
strict = false; % Return a time even when not running
timeline.time(strict)

% In order for other classes and functions to operate on the same time
% scale, a clock object may be passed around.  For example, EXP.SIGNALSEXP
% and HW.DAQCONTROLLER.

% When running timeline, all signals and hardware logs are time aligned.
% The timeline clock object is instantiated with HW.TIMELINECLOCK
clock = hw.TimelineClock(timeline)
clock.now()

% When timeline is disabled during an experiment, all event times are
% system times using the PsychToolbox function GETSECS:
clock = hw.ptb.Clock;
clock.now()

% Clocks can be zeroed, whereby all times are returned relative to some
% reference time:
clock.zero()
clock.now()
```

## Notes

(1) For information on the MATLAB figure properties Position and Units, see their documentation page.

## Etc.

Author: Miles Wells

v1.1.0

```
% INTERNAL
% Image after Intro header:
% <img  height="200" hspace="5" src="Fig7_timeline.png" style="float:right" alt="">
% execute off

%#ok<*NOPTS,*NASGU>
```

Published with MATLAB® R2019a
