# Supplementary material for: Rigbox: An Open-Source Toolbox for Probing Neurons and Behavior
Source: eNeuro. 2020 Jul 9;7(4):ENEURO.0406-19.2020. doi: 10.1523/ENEURO.0406-19.2020 (PMC7363478; doi:10.1523/ENEURO.0406-19.2020)
Supplement: Extended Data 1 — We recommend looking at and downloading the code directly from the github repository, at https://github.com/cortex-lab/Rigbox. Download Extended Data 1, ZIP file. [file enu-eN-OTM-0406-19-s02.zip › Rigbox-master-2020-05-18/Rigbox-master/docs/html/troubleshooting.html]

Troubleshooting 

# Troubleshooting

Often finding the source of a problem seems daunghting when faced with a huge Rigbox error stack. Below are some tips on how to quickly get to the root of the issue and hopefully solve it.

## Contents

- Update the code
- Examining the stack
- Paths
- Reverting
- Posting an issue on Github
- FAQ
- Etc.

## Update the code

Check what version of the code you're using and that you're up-to-date:

```
git.runCmd('status'); % Tells me what branch I'm on
git.update(0); % Update now

% If you're on a development or feature branch try moving to the master
% branch, which should be most stable.
git.runCmd('checkout master'); git.update(0);
```

## Examining the stack

Don't be frightened by a wall of red text! Simply start from the top and work out what the errors might mean and what part of code they came from. The error at the top is the one that ultimately caused the crash. Try to determine if this is a MATLAB builtin function, e.g.

```
Warning: Error occurred while executing the listener callback for event UpdatePanel defined for class eui.SignalsTest:
Error using griddedInterpolant
Interpolation requires at least two sample points in each dimension.
```

```
Error in interp1 (line 151)
F = griddedInterpolant(X,V,method);
```

```
TODO Add better example of builtin errors
```

If you're debugging a signals experiment definition, check for the line in your experiment where this particular builtin function was called. NB: You can check whether it is specific to your experiment by running one of the example experiment definitions such as advancedChoiceWorld.m, found in signals/docs/examples. If this runs without error then you're problem may be specific to your experiment. You should see the name of your definition function and exp.SignalsExp in the stack if they are involved.

If you don't know what a function is, try checking the documentation. Consider the following:

```
Error using open
Invalid number of channels
```

```
Error in audstream.fromSignal (line 16)
  id = audstream.open(sampleRate, nChannels, devIdx);
[...]
```

If you're unsure what `audstream.fromSignal` does, try typing `doc audstream`. This should tell you that the package deals with audio devices in signals. In this case the issue might be that your audio settings are incorrect. Take a look at the audio section of `docs\setup\hardware\_config.m` and see if you can setup your audio devices differently.

## Paths

By far the most common issue in Rigbox relates to problems with the MATLAB paths. Check the following:

1. Do you have a paths file in the +dat package? Check the location by running `which dat.paths`. Check that a file is on the paths and that it's the correct one.
2. Check the paths set in this file. Run `p = dat.paths` and inspect the output. Perhaps a path is set incorrectly for one of the fields. Note that custom rig paths overwrite those written in your paths file. More info found in `using\_dat\_package` and `paths\_template`.
3. Do you have path conflicts? Make sure MATLAB's set paths don't include other functions that have the same name as Rigbox ones. Note that any functions in ~/Documents/MATLAB take precedence over others. If you keep seeing the following warning check that you've set the paths correctly: Warning: Function system has the same name as a MATLAB builtin. We suggest you rename the function to avoid a potential name conflict. This warning can occur if the tests folder has been added to the paths by mistake. Always set the paths by running `addRigboxPaths` and never set them manually as some folders should not be visible to MATLAB.
4. Check your working directory MATLAB prioritizes functions found in your working directory over any others in your path list so try to change into a 'safe' folder before re-running your code: pwd % display working directory cd ~/Documents/MATLAB
5. Check your variable names Make sure your variable names don't shadow a function or package in Rigbox, for instance if in an experiment definition you create a varible called `vis`, you will no longer be able to access functions in the +vis package from within the function: vis = 23; img = vis.image(t); Error: Reference to non-existent field 'image'.

## Reverting

If these errors only started occuring after updating the code, particularly if you hadn't updated in a long time, try reverting to the previous version of the code. This can help determine if the update really was the culprit and will allow you to keep using the code on outdated machines. Previous stable releases can be found on the Github page under releases. NB: For the most recent stable code always pull directly from the master branch

## Posting an issue on Github

If you're completely stumped, open an issue on the Rigbox Github page (or alyx-matlab if you think it's related to the Alyx database). When creating an issue, read the bug report template carefully and be sure to provide as much information as possible.

If you tracked down the problem but found the error to be confusing or too vague, feel free to post a feature request describing how better to present the error. This is an area in need of improvment. You could also make a change yourself and submit a pull request. For more info see CONTRIBUTING.md

## FAQ

Below are some frequently asked questions and suggestions for fixing them. Note there are plenty of other FAQs in the various setup scripts with more specific information.

## Etc.

Author: Miles Wells

v0.1.0

```
% INTERNAL
% execute off
```

Published with MATLAB® R2019a
