# Supplementary material for: Rigbox: An Open-Source Toolbox for Probing Neurons and Behavior
Source: eNeuro. 2020 Jul 9;7(4):ENEURO.0406-19.2020. doi: 10.1523/ENEURO.0406-19.2020 (PMC7363478; doi:10.1523/ENEURO.0406-19.2020)
Supplement: Extended Data 1 — We recommend looking at and downloading the code directly from the github repository, at https://github.com/cortex-lab/Rigbox. Download Extended Data 1, ZIP file. [file enu-eN-OTM-0406-19-s02.zip › Rigbox-master-2020-05-18/Rigbox-master/docs/html/using_dat_package.html]

The Data Package 

# The Data Package

The 'data' package contains code pertaining to the organization and logging of data. It contains functions that generate and parse unique experiment reference ids, and return file paths where subject data and rig configuration information is stored. Other functions include those that manage experimental log entries and parameter profiles. A nice metaphor for this package is a lab notebook.

## Contents

- Setting up the paths
- Using expRefs
- Loading experiments
- Manually creating experiments
- Loading parameters
- Using the log
- Setting custom paths
- Etc.

## Setting up the paths

In order to use Rigbox, a 'paths' file must be placed in a +dat folder somewhere in the MATLAB path. You can copy docs/setup/paths\_template.m to +dat/paths.m, then customise the file according to your setup. The paths used by the wider Rigbox code are found in the 'essential paths' section of the paths\_template.m file. These paths are required to run experiments. Any number of custom repositories may be set, allowing them to be queried using functions such as DAT.REPOSPATH and DAT.EXPPATH (see below).

It may be prefereable to keep the paths file in a shared network drive where all rigs can access it. This way only one file needs updating when a path gets changed. You can also override and add to the fields set by the paths file in a rig specific manner. To do this, create your paths as a struct with the name `paths` and save this to a MAT file called `paths` in your rig specific config folder:

```
rigConfig = getOr(dat.paths('exampleRig'), 'rigConfig');
customPathsFile = fullfile(rigConfig, 'paths.mat');
paths.mainRepository = 'overide/path'; % Overide main repo for `exampleRig`
paths.addedRepository = 'new/custom/path'; % Add novel repo

save(customPathsFile, 'paths') % Save your new custom paths file.

% More info in the paths template:
root = getOr(dat.paths, 'rigbox');
opentoline(fullfile(root, 'docs', 'scripts', 'paths_template.m'), 75)
```

## Using expRefs

Experiment reference strings are human-readable labels constructed from the subject name, date and sequence (i.e. session number). Many of the following functions take one or experiment references as inputs and an experiment reference is constructed each time your create an experiment.

```
ref = dat.constructExpRef('subject', now, 2); % subject's 2nd session today
[subjectRef, expDate, expSequence] = dat.parseExpRef(ref);
```

## Loading experiments

Below are some common ways to query data and paths.

```
% Listing all subjects
subjects = dat.listSubjects;

% The subjects list is generated from the folder names in the main
% repository path
mainRepo = getOr(dat.paths, 'mainRepository');

% To get all paths you should save to for the 'main' repository:
savePaths = dat.reposPath('main'); % savePaths is a string cell array
% To get the master location for the 'main' repository:
loadPath = dat.reposPath('main', 'master'); % loadPath is a string
% If you have alternate repos (e.g. 'main2Respository', 'altRepository'),
% use the remote flag to return all of them (used by the below functions).
% NB: the 'altRepository' is returned for all named repos, not just 'main'
loadPath = dat.reposPath('main', 'remote');
% To return all paths ending in 'Repository':
endInRepos = dat.reposPath('*');

% List experiments for a given subject
[ref, date, seq] = dat.listExps(subject);

% Return experiment path
% These functions can take the input as both a ref or three inputs
% (subject, date and sequence).  The input may also be a cell array of
% these.
p = dat.expPath(ref);
[p, ref] = dat.expPath(subject, now, 1, 'main');

% Check a given experiment exists
bool = dat.expExists(ref);

% Return specific file path
[fullpath, filename] = dat.expFilePath(ref, 'block');
[fullpath, filename] = dat.expFilePath(ref, 'block', 'master', 'json');
[fullpath, filename] = dat.expFilePath(subject, now, 1, 'timeline');

parameters = dat.expParams(ref);
block = dat.loadBlock(ref, expType);
clearCBToolsCache % Clear the cached block file
```

## Manually creating experiments

The expParams variable will be saved to 'localRepository' and master 'mainRepository' paths

```
[expRef, expSeq] = dat.newExp(subject, expDate, expParams);
```

## Loading parameters

The dat package allows you to load both session specific and experiment specific parameters .

```
expType = 'custom'; % signals experiments have the type 'custom'
p = dat.loadParamProfiles(expType);
dat.saveParamProfile(expType, profileName, params);
dat.delParamProfile(expType, profileName);

% More info on how parameters work can be found in USING_PARAMETERS:
open(fullfile(getOr(dat.paths,'rigbox'), 'docs', 'scripts', 'using_parameters.m'))
```

## Using the log

The log object, is primarily dealt with through MC, however you can also use it from the command line:

```
e = dat.addLogEntry(subject, timestamp, type, value, comments, AlyxInstance);
p = dat.logPath(subject, 'all');
e = dat.logEntries(subject);
e = dat.updateLogEntry(subject, id, newEntry);
```

## Setting custom paths

Some people keep the paths file in a shared remote location that all rigs can access. This reduces the number of files to change when a repository path needs updating to one. In this case, rig-specific paths may be set using a custom paths file that overrides any paths set in DAT.PATHS:

```
opentoline(fullfile(getOr(dat.paths,'rigbox'), ...
  'docs', 'scripts', 'paths_template.m'), 78, 1)

% The paths file, 'paths.mat', must contain a variable `paths` that is a
% struct of custom paths.  The file should be located in the location set
% by 'rigConfig'.  For obvious reasons do not overrive 'rigConfig' in you
% custom paths without making the appropriate changes to DAT.PATHS.

% Let's create a custom paths file for rig 'ZREDONE' containing a new
% location for the 'expDefinitions' path:
clear paths
paths.expDefinitions = 'C:\ExpDefinitions';
customPathsFile = fullfile(getOr(dat.paths('ZREDONE'), 'rigConfig'), 'paths');
save(customPathsFile, 'paths', '-mat')
```

## Etc.

Author: Miles Wells

v1.0.1

```
%#ok<*NASGU,*ASGLU,*ASGLU>
```

Published with MATLAB® R2019a
