# Supplementary material for: Rigbox: An Open-Source Toolbox for Probing Neurons and Behavior
Source: eNeuro. 2020 Jul 9;7(4):ENEURO.0406-19.2020. doi: 10.1523/ENEURO.0406-19.2020 (PMC7363478; doi:10.1523/ENEURO.0406-19.2020)
Supplement: Extended Data 1 — We recommend looking at and downloading the code directly from the github repository, at https://github.com/cortex-lab/Rigbox. Download Extended Data 1, ZIP file. [file enu-eN-OTM-0406-19-s02.zip › Rigbox-master-2020-05-18/Rigbox-master/docs/html/using_ExpPanel.html]

Introduction 

# Introduction

ExpPanels are panels under the Experiment > Current tab of mc that display information about events occuring during an experiment. This document contains information on how to set up an ExpPanel for customizing the monitoring of an Experiment.

## Contents

- exp.ExpPanel
- Basic layout
- Title
- InfoGrid
- CustomPanel
- CommentsBox
- ButtonPanel
- Method call sequence
- exp.SignalsExpPanel
- Custom Signals ExpPanels
- Notes, etc.

## exp.ExpPanel

The base class for the ExpPanel is the exp.ExpPanel. All subclasses chain a call to this class.

When starting a new experiment in MC a new ExpPanel is created by calling the static contructor method `live`:

```
p = live(parent, ref, remoteRig, paramsStruct, activateLog)
doc eui.ExpPanel/live
```

The precise subclass used depends on the `type` parameter in the paramsStruct input. Currently supported types include SingleTargetChoiceWorld, ChoiceWorld, DiscWorld, SurroundChoiceWorld (eui.ChoiceExpPanel); BarMapping (eui.MappingExpPanel); custom a.k.a. Signals (eui.SignalsExpPanel).

For Signals experiments the default ExpPanel class may be overridden by providing a parameter named `expPanelFun` whose value is either a function handle for an ExpPanel constructor or path to the class to be instantiated. This parameter is automatically added in MC if the folder from which the experiment function was loaded contains an ExpPanel. The name must be the same as the experiment function but with 'ExpPanel' added, e.g. for 'advancedChoiceWorld.m', the corresponding ExpPanel file would be 'advancedChoiceWorldExpPanel.m'.

## Basic layout

The ExpPanel has the following basic layout...

## Title

The panel title contains the experiment reference and the name of the remote rig. When the experiment is initializing or during the cleanup/post-delay phase the title is amber. During the main experiment phase the title turns green and when complete, red. This title colour and other properties are set in the `live` method then subsequently by the `event` method. The title is stored in the Root.Title property.

## InfoGrid

The info grid contains all experiment event labels and their current values. As new events occur they're added to the last via a call to addInfoField. There are 4 default fields:

- Status - The current experimental phase, e.g. 'Pending', 'Complete'. The status is set based on 'ExpUpdate' events from the remote rig (see the `expUpdate` method).
- Duration - The time elapsed since the experiment began. This is updated each time the `update` method is called (every 100ms in MC).
- Trial count - The total number of trials. This field is updated based on the 'newTrial' ExpUpdate status (see `expUpdate` method).
- Condition - The current trial condition. This only appears if `conditionId` parameter is defined.

The fields may be hidden by right-clicking one and selecting 'Hide field'. The hidden fields may be reset by selecting 'Reset hidden'.

## CustomPanel

A container for subclasses to build plots into. For example in the ChoiceWorld Experiment, this contains a psychometric curve plot and the trace of the wheel input.

## CommentsBox

An input field for taking notes. These are automatically saved to the Log (see dat.logPath, dat.logEntries). If logged into Alyx the notes are also saved to the database session narrative (see Alyx.updateNarrative). The comments box may be hidden by right-clicking and selecting 'Hide comments'.

## ButtonPanel

A set of buttons for ending/aborting the experiment as well as viewing the parameter set. If End is pressed, the experiment is ended after the post delay, the block's endStatus field is set to 'quit', and ALF files may be extracted from the block. If Abort is pressed, the post delay is skipped, the endStatus is set to 'aborted' and no ALF files are extracted from the block during save.

## Method call sequence

Below is the sequence of method calls. This is useful to be aware of when making your own subclass.

```
mc/beginExp
     |
eui.ExpPanel/live
     |
eui._ExpPanel/_ExpPanel (may be a subclass constructor, e.g.
     |                   SignalsExpPanel)
eui._ExpPanel/build (subclasses should chain call to superclass build)
     |
eui.ExpPanel/addInfoField (adds any default info labels, e.g.
                           TrialCount)
```

```
eui._ExpPanel/update (mc timer callback)
     | (if eui.SignalsExpPanel or subclass.
     |  NB: All subclasses should chain a call to superclass update)
eui._ExpPanel/processUpdates (method only present in SignalExpPanel
     |                        classes)
eui.ExpPanel/addInfoField (adds any new Signals event fields)
```

## exp.SignalsExpPanel

The subclass, eui.SignalsExpPanel, is the default class for Signals Experiments. In this class, all Signals updates are shown as InfoFields whose colours pulse green as the values update. The signals sent from the stimulus computer includes events, parameters, inputs and outputs signals. The 'Trial count' field reflects the value of events.trialNum.

The UpdatesFilter property contains a list of signals updates to create a label for, or if Exclude == true, all signals names in this list are ignored. This is useful when your events structure is large and you don't wish to see all of them during the experiment.

exp.SignalsExp periodically(1) sends signals event updates to the MC computer(2). These updates trigger the expUpdate method which stores the updates in the SignalUpdates property. All updates in this property are delt with and removed by the processUpdates method, which is called via the update by the MC Refresh timer once per 100ms(3).

The SignalUpdate property is a struct with the following fields:

- name - The name of the signal, e.g. 'events.newTrial'
- value - The value of the signal.
- timestamp - a date vector of the date and time when the signal was queued. (NB: This is in the system time of the remote rig and depends on its timezone. These timestamps aren't as precise as those in the block file).

When new updates are processed in eui.SignalsExpPanel, if an info field does not already exist, one is created. When the FormatLabels property is true the Signals update labels are formatted as sperate words. For example 'events.newTrial' is displayed as 'New trial'. This flag and others such as the UpdatesFilter can be set it your subclass constructor.

## Custom Signals ExpPanels

Below is a list of steps to follow when creating a custom Signals ExpPanel, for an example of this see advancedChoiceWorldExpPanel(4):

1. Subclass eui.SignalsExpPanel Subclassing means you will inherit all of the methods and properties found in eui.SignalsExpPanel and eui.ExpPanel.
2. Add any extra properties specific to your ExpPanel For example if you're creating a new plot you may wish to store the axes in a property. (c.f. PsychometricAxes in advancedChoiceWorldExpPanel)
3. Add a constructor to initialize any properties if required Chain a call to the superclass method like so: obj = obj@eui.SignalsExpPanel(parent, ref, params, logEntry);
4. Add a build method to initialize an axes or extra UI elements. Typically everything built here will use obj.CustomPanel as the parent container. This method must have protected access. Chain a call to the superclass method first: build@eui.SignalsExpPanel(obj, parent);
5. Add a processUpdates to deal with your experiment-specific events. Here you can add code to update plots, etc. based on the event updates. This method must have protected access. Instead of chaining a call, copy the code from eui.SignalsExpPanel/processUpdates directly and use it as a template for your own functions.

There are some useful superclass methods that are useful to keep in mind:

- mergeTrialData - Update the local block structure with data from the last trial. This is found in eui.ExpPanel.
- newTrial - This doesn't do anything in the superclasses but is a good place to put code that should be executed at events.newTrial. Call it from processUpdates.
- trialCompleted - As with newTrial, this could be used as a place for code that runs after e.g. an outputs or feedback event.
- expStopped - Useful for executing code when the session ends. Must chain a call to superclass here.
- expStarted - See above.
- cleanup - Place code here for e.g. stopping timers, clearing listeners, etc.

Here are some useful properties to be aware of:

- Parameters - A copy of that experiment's paramStruct.
- UpdatesFilter - As mentioned above, this holds a cell array of events you wish to ignore/include. It's behaviour depends on whether the Exclude property is true or false.
- RecentColour - The colour of recently updated Signals update events in the InfoGrid. This can be changed dynamically during the session, for instance could turn red as the subject's performance declines or towards the end of the session.

Finally there are some other useful untilities to be aware of:

- +psy - The psy package contains useful functions for plotting psychometrics and producing fits.
- bui.Axes - This class provides a convenient way to interact with plotting axes. It's particularly useful if you wish to add multple elements to the same axes. Below is an example from advancedChoiceWorld:

```
obj.ExperimentAxes = bui.Axes(plotgrid); % Create new bui.Axes object
obj.ExperimentAxes.ActivePositionProperty = 'position';
obj.ExperimentAxes.XTickLabel = []; % Remove the X tick labels
obj.ExperimentAxes.NextPlot = 'add'; % Add new plots without clearing axes
% First initialize a plot for the wheel trace and store the resulting axes
obj.ExperimentHands.wheelH = plot(obj.ExperimentAxes,...
  [0 0],...
  [NaN NaN],...
  'Color', .75*[1 1 1]);
% Now initialize a threshold line on the same plot
obj.ExperimentHands.threshL = plot(obj.ExperimentAxes, ...
  [0 0],...
  [NaN NaN],...
  'Color', [1 1 1], 'LineWidth', 4);
% Note that updating plots can be memory intensive, so consider tweaks such
% as updating the underlying plot data instead of clearing and redrawing:
set(obj.ExperimentHands.wheelH,...
  'XData', xx,...
  'YData', tt);
% Also take a look at the drawnow builtin function:
doc drawnow
```

## Notes, etc.

(1) exp.SignalsExp sends any new Signals event updates once every 100ms: opentoline(which('exp.SignalsExp'), 731, 9)

(2) Any number of computers may listen for these updates, see websocket\_config

(3) See eui.MControl: opentoline(which('eui.MControl'), 84, 7)

(4) The below three lines will open this file:

```
% rigbox = getOr(dat.paths, 'rigbox'); % Location of Rigbox code
% exampleExps = fullfile(rigbox, 'signals', 'docs', 'examples');
% open(fullfile(exampleExps, 'advancedChoiceWorldExpPanel.m'))

% Author: Miles Wells
%
% v1.0.0

%#ok<*NOPTS,*ASGLU,*NASGU>
```

Published with MATLAB® R2019a
