# Supplementary material for: Rigbox: An Open-Source Toolbox for Probing Neurons and Behavior
Source: eNeuro. 2020 Jul 9;7(4):ENEURO.0406-19.2020. doi: 10.1523/ENEURO.0406-19.2020 (PMC7363478; doi:10.1523/ENEURO.0406-19.2020)
Supplement: Extended Data 1 — We recommend looking at and downloading the code directly from the github repository, at https://github.com/cortex-lab/Rigbox. Download Extended Data 1, ZIP file. [file enu-eN-OTM-0406-19-s02.zip › Rigbox-master-2020-05-18/Rigbox-master/docs/html/using_services.html]

Introduction 

# Introduction

Timeline outputs are useful for triggering external hardware, but what about notifying other software of an experiment? Services in Rigbox allow one to trigger software on other devices, including starting and stopping Timeline if it is running on a separate computer. Services are simply objects that send UDP messages at various times during an experiment.

## Contents

- The Service class
- Workflow
- Customising your services
- Primative UDP Service
- Basic UDP Service
- MPEP UDP Data Hosts
- Selecting services
- Conclusion
- Notes
- Etc.

## The Service class

Service classes have at least two methods, start and stop. At the beginning of an experiment srv.expServer will load these services objects and run the start method for each, likewise at the send on an experiment the stop method is called. This provides a flexible way to perform custom tasks during an experiment, and is technically not limited to triggering software with UDPs: if you wanted you could create your own subclass that does something entirely different, such as opening a file or running system commands.

```
doc srv.Service
```

## Workflow

The sequence of events leading up to services starting is somewhat convoluted!

When srv.expServer receives a messages from MC to begin a new experiment it first starts Timeline locally (if required), then calls srv.prepareExp which initializes the experiment, then calls srv.findService with a list of services to be started. srv.findService loads srv.basicServices which loads all configured service objects. srv.findService returns all requested services to srv.prepareExp, which creates stores them in both an exp.StartServices and exp.StopServices object. These objects are added as callbacks to the experimentInit event. srv.expServer now calls run on the experiment object, which during in turn triggers the experimentInit event, whereby the Service objects send their UDPs to start the auxillary software. Below is the stack starting at #1 and going down, left to right:

```
% |srv.expServer/runExp|-(2)------>|
%  ^       (1)                     |
%  |        |                      V
%  | |srv.prepareExp| ----> |exp.SignalsExp| (or other experiment)
%  |        |                      |
%  |        |--> |srv.findService|-:-> |srv.basicServices| --> |srv.Service|
%  |        |           |          |           |<--------------------|
%  |        |           |<---------|-----------|
%  |        |<----------|          V
%  |        |-------------->|exp.StartServices|
%  |<-------|
%
```

## Customising your services

The services are created by expServer at the start of the experiment. It calls the function srv.findService which in turn calls the function srv.basicServices. Either of these files may be edited to configure the services you need.

A simple way of doing this is to create a folder named +srv in the MATLAB folder (usually found in ~\Documents). Anything in this folder appears at the top of the your paths and therefor you can shadow Rigbox's default basicServices function with the one you create in the MATLAB folder. This way your modified function won't be affected by pulls from Git.

Your srv.basicServices function should have the host names for each service as input and ouput a cell array of srv.Service objects: function [services] = basicServices(timelineHost, neuralImgHost, eyeTrackingHost)

Let's have a look at configuring some services with the srv.PrimitiveUDPService subclass. This used pnet, a networking function that comes with PsychToolbox to sent and recieve UDP messages:

```
% Configure scanimage/neural acquisition service
% The class is called with the remote computer's host name.
neuralImg = srv.PrimitiveUDPService(neuralImgHost);
% The title is something human-readable that will be used in command window
% status updates
neuralImg.Title = 'Neural imaging';
% The id must match the name stored in your StimulusControl object (more on
% this later)
neuralImg.Id = 'neural-imaging';
% The response timeout in seconds.  If the remote devices fails to respond
% in this time, expServer with throw an error and the experiment won't
% start.
neuralImg.ResponseTimeout = 5;
% The listen and remote ports may also be set either here are as positional
% arguments to the constructor.  The defaults are 10000.
neuralImg.RemotePort = 10000;

services = {timeline neuralImg eyeTracking}; % Our example output
```

## Primative UDP Service

The service is started by calling the start method with an experiment reference string:

```
ref = dat.constructExpRef('fake', now, 1);
neuralImg.start(ref)

% The object sends the message GOGO<ref>*<host> where <ref> is the
% experiment reference and <host> is the hostname.  The remote computer is
% expected to echo message within the timeout period.  It is printed to the
% command window.

% Calling stop is similar:
neuralImg.stop(ref) % Send STOP*<host>
% The message should be echoed back

% The is also a status method.  When the property Status is accessed the
% following message is sent:
status = neuralImg.Status; % Send WHAT814724*<host>
%
% The same random number is expected to be returned along with the status,
% e.g.
% GOGO814724 % means device running
```

## Basic UDP Service

Another subclass is called srv.basicUDPService. This is similar to primativeUDPService however it is a little more flexible and uses MATLAB's instrument control toolbox. It can also send messages asynchronously.

```
doc srv.basicUDPService

% Below is a configuration for starting a remote computer that controls
% some galvo moters during an experiment:

% Some default hosts
if nargin < 1
  galvoHost = 'zimbabwe';
end
% Configure galvo service
galvo = srv.BasicUDPService(galvoHost, 10002, 10000);
galvo.Title = sprintf('Galvo on %s', galvoHost);
galvo.Id = 'galvo';
galvo.ResponseTimeout = 5;

services = {galvo};

% The messages sent by this class are similar to those of
% srv.PrimativeUDPService.
```

## MPEP UDP Data Hosts

Finally there is also a subclass called io.MpepUDPDataHosts(1). This class can also pass around an instance of Alyx via UDP, allowing remote computers to register their files to the Alyx database. Again, unlike srv.PrimativeUDPService which sends and receives messages synchronously, io.MpepUDPDataHosts sends the start and stop messages to all remote hosts one after the other without waiting for responses.

Below is some example code. Note that this code is found in srv.findService, rather than srv.basicServices, which has `id` as its first input argument, which matches the Id property of a Service:

```
timelineHost = iff(any(strcmp(id, 'timeline')), {'zcamp3'}, {''});
neuralImgHost = iff(any(strcmp(id, 'b-scope')), {'zscope'}, {''});
eyeTrackingHost = iff(any(strcmp(id, 'eye-tracking')), {'zquad'}, {''});

remoteHosts = [timeHost neuralImgHost eyeTrackingHost];
emp = cellfun(@isempty, remoteHosts);

MpepHosts = io.MpepUDPDataHosts(remoteHosts(~emp));
MpepHosts.ResponseTimeout = 30;
MpepHosts.Id = 'MPEP-Hosts';
MpepHosts.Title = 'mPep Data Acquisition Hosts';
MpepHosts.open();
s = {MpepHosts};

% The messages sent by this class are different to the others.  With this
% object you can send an instance of Alyx with the start command.  Below
% are a selection of messages sent at the start of an experiment:

% Ping
obj.broadcast('hello');
% Send Alyx instance UDP
UDP_msg = Alyx.parseAlyxInstance(expRef, ai);
[subject, seriesNum, expNum] = dat.expRefToMpep(expRef);
alyxmsg = sprintf('alyx %s %d %d %s', subject, seriesNum, expNum, UDP_msg);
confirmedBroadcast(obj, alyxmsg);
% Send ExpStart UDP
expStartMsg = sprintf('ExpStart %s %d %d', subject, seriesNum, expNum);
confirmedBroadcast(obj, expStartMsg);
% Send the BlockStart UDP
blockStartMsg = sprintf('BlockStart %s %d %d 1', subject, seriesNum, expNum);

% There are also methods for ending stimulus start and status messages.
% Note that the order in which the devices are started is sometimes
% important, particularly if you many inter-connected devices.  For example
% you may have a camera that is triggered by Timeline's Clock Output and
% some camera software is programmed to start saving the frames to file
% after receiving a UDP:

% expServer       Computer A               Computer B
% ------------------------------------------------------
% Service ----> Timeline.start() -----> [trigger camera]
%                       |                       |
% Service <----started--|                       |
%                                               |
% Service -----> start(eye-tracking) <-----------
%                       |
% Service <-----started-|
```

## Selecting services

Once you have set up your devices you need to add them to your rig's StimulusControl object. This is the object MC uses to access information about availiable stimulus computers.

```
doc srv.StimulusControl

% One of the properties of this object is Services.  Here you can list all
% the configured services for that rig.  This must match the Id properties
% of the services:
sc = srv.stimulusControllers % Load our StimulusControl objects
sc(1).Services = {'neural-imaging', 'eye-tracking', 'timeline'};

% These appear as toggles in the 'rig options' dialog in MC, allowing you
% to select which services you need starting for a given experiment.  You
% can also set the default state by changing the SelectedServices property:
sc(1).SelectedServices = [false, false, true]; % Only timeline on by default

% Setting default delays is sometimes useful.  These can also be changed in
% the 'rig options' dialog in MC.  The ExpPreDelay property is the time in
% seconds to wait between starting the services and actually beginning the
% experiment (for a Signals experiment this means updating the
% 'events.expStart' signal).  This can be useful when a service takes some
% time to initialize, or if you want to record some sort of baseline
% activity before the first stimulus appears.
sc(1).ExpPreDelay = 10; % Send the start message to services then wait 10s

% Likewise the ExpPostDelay is the time in seconds between the experiment
% ending (i.e. the events.expStop signal updating), and the stop command
% being sent to the services.  This may be useful if you wish to record
% some baseline after the stimulus presentation has ended for example.
sc(1).ExpPostDelay = 30; % Wait 30s then send the stop message to services

% Save your objects:
stimulusControllers = sc; % Variable must be saved as stimulusControllers
save(fullfile(p.globalConfig, 'remote.mat'), 'stimulusControllers')

% More information can be found in the <./websocket_config.html
% websocket_config> script:
root = getOr(dat.paths, 'rigbox');
open(fullfile(root, 'docs', 'scripts', 'websocket_config.m'))
```

## Conclusion

Ultimately the way you configure your services will be idiosyncratic as it depends on what devices you need to trigger and what information they require from the experiment. Hopefully the above gives you a good enough idea of the ways in which you can integrate other equipment and devices into Rigbox.

## Notes

(1) MPEP is another, passive stimulus presentation toolbox that predates Rigbox. The functions io.MpepUDPDataHosts and tl.mpepListener / tl.bindMpepServer were designed to work with both Rigbox and MPEP. The latter is the function we run on the receiving end.

## Etc.

Author: Miles Wells

v1.1.1

```
%#ok<*FINS,*NASGU,*NOPTS>
```

Published with MATLAB® R2019a
