# Supplementary material for: Rigbox: An Open-Source Toolbox for Probing Neurons and Behavior
Source: eNeuro. 2020 Jul 9;7(4):ENEURO.0406-19.2020. doi: 10.1523/ENEURO.0406-19.2020 (PMC7363478; doi:10.1523/ENEURO.0406-19.2020)
Supplement: Extended Data 1 — We recommend looking at and downloading the code directly from the github repository, at https://github.com/cortex-lab/Rigbox. Download Extended Data 1, ZIP file. [file enu-eN-OTM-0406-19-s02.zip › Rigbox-master-2020-05-18/Rigbox-master/docs/html/using_single_rig.html]

Running experiments on a single computer 

# Running experiments on a single computer

Running experiments with two PCs has two major advantages:

1. An MC computer can control and monitor multiple stimulus computers in parallel.
2. Using mc on a separate computer frees up the stimulus computer's resources. A dedicated experiment computer is likely to have lower latencies.

Nevertheless, it is possible to run experiments using a single computer. The first way is by using srv.expServer's 'single-shot' mode to run an experiment without running mc. The second way is by running mc on the same computer, in a different instance of MATLAB.

## Contents

- Without MC
- With MC
- Etc.

## Without MC

Running experiments on a single computer without MC is simple, however live monitoring of the experiment is not possible. First a new experiment is created, then srv.expServer should be called the experiment reference string. The below code shows how to create and run a ChoiceWorld Experiment using the default parameters, without using Alyx:

```
ref = dat.newExp('test', now, exp.choiceWorldParams);
srv.expServer('expRef', ref, 'preDelay', 10) % Ten second delay before start
```

Below is an example of modifying parameters for a Signals Experiment, then create an experiment in Alyx and run it:

```
% Get the parameter list using inferParameters
paramStruct = exp.inferParameters(@advancedChoiceWorld);

% Modify the parameters using the exp.Parameters object
P = exp.Parameters(paramStruct); %
P.makeTrialSpecific('rewardSize')
P.set('rewardSize', linspace(1,3,P.numTrialConditions))

% Parameters can also be manipulated in the Parameter Editor GUI
PE = eui.ParamEditor(P);
paramStruct = PE.Parameters.Struct;

% Save parameters and register session to Alyx
ai = Alyx;
ref = newExp(ai, 'test', now, P.Struct);
srv.expServer('expRef', ref, 'alyx', ai)
```

## With MC

It is also possible to run mc on the same computer as srv.expServer. This requires that the computer has at least 2 monitors connected.

To do this set up the remote file according to the Configuring WebSockets guide, however, instead of using the hostname or external IP as the URI, use the localhost address (normally 127.0.0.1). Below is the code for setting up the remote file this way:

```
% The stimulus controllers are loaded from a MAT file with the name
% 'remote' in the globalConfig directory, defined in dat.paths:
p = fullfile(getOr(dat.paths, 'globalConfig'), 'remote.mat');

% Let's create a stimulus controller for this PC
stimulusControllers = srv.StimulusControl.create(hostname, '127.0.0.1');

% Save your new configuration.  Note the variable name must be as below:
save(p, 'stimulusControllers')
```

Now simply open another instance of MATLAB and in one, run mc. In the other instance, run srv.expServer.

## Etc.

Author: Miles Wells

v0.0.1

Published with MATLAB® R2019a
