# Supplementary material for: Rigbox: An Open-Source Toolbox for Probing Neurons and Behavior
Source: eNeuro. 2020 Jul 9;7(4):ENEURO.0406-19.2020. doi: 10.1523/ENEURO.0406-19.2020 (PMC7363478; doi:10.1523/ENEURO.0406-19.2020)
Supplement: Extended Data 1 — We recommend looking at and downloading the code directly from the github repository, at https://github.com/cortex-lab/Rigbox. Download Extended Data 1, ZIP file. [file enu-eN-OTM-0406-19-s02.zip › Rigbox-master-2020-05-18/Rigbox-master/docs/html/using_test_gui.html]

using\_test\_gui 

## Contents

- Introduction
- Opening your expDef in the GUI
- Default settings
- Testing different hardware
- Editing parameters
- Live plotting
- Experiment panel
- Debugging
- Notes
- Etc.

## Introduction

This document demonstrates how to test Signals Experiment Definition (expDef) functions in the test GUI. The GUI opens a PTB window and a Parameter Editor for live-updating parameters. Before opening the test GUI, loading the debug settings for PTB will make the window transparent. This is particularly useful on small screens(1).

```
PsychDebugWindowConfiguration
```

## Opening your expDef in the GUI

Upon calling the eui.SignalsTest class with no inputs you will be prompted to select your function from the file browser. As with MC, the default folder location is set by the 'expDefinitions' field in dat.paths.

You can also call the function with the function name or function handle. The function must be on the MATLAB path. Let's run one of the example expDef functions: the Burgess wheel task(2) implemented in Signals.

```
PsychDebugWindowConfiguration % Make window transparant and turn of blocking
root = fileparts(which('addRigboxPaths')); % Location of Rigbox root dir
cd(fullfile(root, 'signals', 'docs', 'examples')) % Change to examples folder

e = eui.SignalsTest('advancedChoiceWorld') % Start GUI and loaded expDef
% e = eui.SignalsTest() % Opens a file navigator
% e = eui.SignalsTest(@advancedChoiceWorld) % Function handle
% e = eui.SignalsTest('full/path/to/expDef.m') % Absolute file path
```

```
PTB-INFO: Using modified PortAudio V19.6.0-devel, revision unknown

e = 

  SignalsTest with properties:

          Hardware: [1×1 struct]
               Ref: '2020-02-19_1_test'
    LoggingDisplay: [1×1 UIControl]
          LivePlot: off
      ShowExpPanel: on
      SingleScreen: off
        Experiment: [0×0 exp.test.Signals]
       ParamEditor: [1×1 eui.ParamEditor]
       LivePlotFig: [0×0 Figure]
          ExpPanel: [0×0 eui.ExpPanel]
         IsRunning: 0
```

## Default settings

The hardware wheel input is simulated in the experiment GUI by the position of the cursor over the stimulus window. Upon clicking start the 'expStart' event updates with the the experiment ref string. The ref string can be changed by editing the Ref property before pressing start:

```
e.Ref = dat.constructExpRef('subject', now-7, 2);

% Moving the cursor over the window will move the visual stimulus in the
% stimulus window.
e.Hardware.mouseInput % Uses the eui.SignalsTest/getMouse method
```

```
ans = 

  struct with fields:

    readAbsolutePosition: @(varargin)obj.getMouse(varargin{:})
       MillimetresFactor: 0.1000
       EncoderResolution: 1
              ZeroOffset: 0
                    zero: @nop
```

## Testing different hardware

A hardware structure can be assigned to the Hardware property of the test object (see configuring hardware devices):

```
e.Hardware = hw.devices;  % Assign a rig's actual hardware settings
```

## Editing parameters

Parameters are shown in the eui.ParamEditor which is in the 'Parameters' box. When you select an expDef, the default parameters are loaded. These are the names and values defined in the try-catch block at the end of the expDef. Parameters can be edited before pressing start, and saved/loaded using the 'saved sets' section. Saved sets are located in the mainRepository (set in your dat.paths file), in a file called 'parameterProfiles.mat'. For more info see Using Parameters. The parameters can be updated live during the experiment. In order to do this, check 'Post new parameters on edit' in the options dialog.

## Live plotting

The values of the event Signals can be plotted live by checking the LivePlot option in the Options popup.

Clicking on each subplot will cycle through the three plot modes. The default mode (0) creates a stair plot with each value update marked with an x. Mode 1 plots each value as a discrete point. Mode 2 plots a simple line, with now markers to indicate value updates. Note, if a Signal takes a vector or matrix as its value, the mode is switched to 1 and the size of the array is added as an text annotation. If the value is a charecter array, the mode is switched to 1 and the value is plotted as a text annotation. For more details on the plotting function see sig.test.timeplot.

For more about options see the help for the setOptions method.

```
e.setOptions % Open Options dialog
help eui.SignalsTest/setOptions
```

```
  SETOPTIONS callback for 'Options' button
    Sets various parameters related to monitering the experiment.
    
    Options:
      Plot Signals (off): Plot all events, input and output Signals
        against time in a separate figure.  Clicking on each subplot 
        will cycle through the plotting styles.
      Show experiment panel (on): Instantiate an eui.SignalsExpPanel
        for monitoring the experiment updates.  The ExpPanelFun
        parameter defines a custom ExpPanel function to display.  NB:
        Unlike in MC, the comments box is hidden.
      View PTB window as single screen (off): When true, the default
        setting of the window simulates a 4:3 aspect ratio screen.
      Post new parameters on edit (off): When true, whenever a
        parameter is edited while the experiment is running, the
        parameter Signals immediately update.
 
  See also SIG.TEST.TIMEPLOT, EUI.SIGNALSEXPPANEL
```

## Experiment panel

The 'Show experiment panel' option is on by default and instantiates an experiment panel (ExpPanel) that displays Signals updates after the experiment has started. A custom ExpPanel can be defined via your expDef's 'expPanelFun' parameter, otherwise eui.SignalsExpPanel is used. Note that unlike in MC, the comments box is hidden from the panel. For more info, see Using ExpPanel.

## Debugging

A number of features make debugging a little easier:

1. The expDef is re-loaded each time you start the experiment, so you can make changes without having to reload the GUI.
2. The parameters can be updated live during the experiment (see Editing Parmeters).
3. You can pause and resume the experiment at any time by pressing the esc key. While paused, no Signals are updated. Note that while the experiment is running you can not use the command prompt, even when paused.
4. Signals is run in debug mode, so the error messages printed give more informative information about the exact cause of the error. Below is an example of a typical Signals error thrown. The top of the stack gives the name of the function that threw the error, the error message and the line at which it occured. The 'Caused by' section gives the node ids involved (e.g. node 61 -> node 62), their values at the time of the error (e.g. bombWorld/timeSampler([0; 0.1; 0.09]) ), and the name and line of problem Signal in your expDef, e.g. 'Error in [...]bombWorld.m (line 22)'; pars.preStimulusDelay.map(@bombWorld/timeSampler). NB: If you call eui.SignalsTest with a function handle, the line number of your expDef can not be determined.

```
Error using bombWorld/timeSampler (line 183) Expected char; was double instead.


[...]


Caused by:  
     Error in Net 1 mapping Node 61 to 62:  
     function call 'bombWorld/timeSampler' with input [0; 0.1; 0.09] produced an error:  
      Expected char; was double instead.  
     Error in C:\Users\User\Documents\Github\rigbox\signals\docs\examples\bombWorld.m (line 22)  
     pars.preStimulusDelay.map(@bombWorld/timeSampler)
```

## Notes

(1) These settings can be cleared by calling the Screen function:

```
clear Screen
% (2) <https://doi.org/10.1016/j.celrep.2017.08.047 DOI:10.1016/j.celrep.2017.08.047>
```

## Etc.

Author: Miles Wells

v0.1.0

```
% INTERNAL:
%  execute code
%#ok<*NOPTS,*NASGU,*ASGLU>
```

Published with MATLAB® R2019a
