# Supplementary material for: Rigbox: An Open-Source Toolbox for Probing Neurons and Behavior
Source: eNeuro. 2020 Jul 9;7(4):ENEURO.0406-19.2020. doi: 10.1523/ENEURO.0406-19.2020 (PMC7363478; doi:10.1523/ENEURO.0406-19.2020)
Supplement: Extended Data 1 — We recommend looking at and downloading the code directly from the github repository, at https://github.com/cortex-lab/Rigbox. Download Extended Data 1, ZIP file. [file enu-eN-OTM-0406-19-s02.zip › Rigbox-master-2020-05-18/Rigbox-master/docs/html/using_visual_stimuli.html]

Visual stimuli in Signals 

# Visual stimuli in Signals

Signals uses the OpenGL MEX wrapper functions provided by PsychToolbox to render the visual stimuli.

In order to build up an enviroment we need to know a few things:

1. Where are the objects (stimuli) with respect to one another in the world
2. Where are the objects with respect to the viewer
3. How to do these coordinates map to a 2D surface (a screen)

Thus when we define a stimulus in visual space it is transformed by our model into physical space by the model (or world-to-camera) matrix then to projected 2D space by our projection matrix. These transformations are done in the shader.

## Contents

- The visual stimulus object
- vis.screen
- vis.init
- Plane projection
- The shader
- Clip space
- NDC space
- Rasterization
- vis.draw
- Viewing model
- Layers
- Stimuli
- Images
- Checker / sparse noise
- Gabor patch & gratings
- Shapes
- Dynamic textures
- vis.rgba & vis.rgbaFromUint8
- Notes
- Etc.

## The visual stimulus object

The visual stimulus object is the 4th input to an experiment definition. It is a StructRef object, which like a structure, can be assigned fields.

## vis.screen

The model produced by vis.screen is a matrix (known as the world-to-camera matrix) that transforms our world coordinates (visual degrees) to camera coodinates (the physical location of the viewer). It technically does two transforms in one: object-to-world the world-to-camera, however in our viewing model the the camera IS the world, so the object-to-world matrix is identity. See also hardware configuration:

```
root = fileparts(which('addRigboxPaths'));
opentoline(fullfile(root, 'docs', 'scripts', 'hardware_config.m'), 344, 1)
```

## vis.init

Loads the shader (slimshady) and initializes a projection matrix with default viewing parameters.

The viewing model is 'pseudo-circular'. An inverted spherical mesh is created using vis.uniSphereTriangles onto which all textures are painted. This is designed to compensate for the fact that the screen edges are further away than the centre when the viewer is facing the middle of the screen. As textures move further along the azimuth, they enlarge.

## Plane projection

A 4x4 plane projection matrix when multiplied by a 3D coordinates in camera space gives you the 2D coordinates on the screen/projection surface. This maxtrix allows us to map 3D coordinates to images that follow the rules of perspective. Clipping happens here.

## The shader

This is the job of the vertex shader. The shader's job is to transform our vertices from camera space (visual degrees) to clip space. The shader returns 'gl\_Position' (an OpenGL global variable) which is the result of multiplying vertex postion by the plane projection and world-to-camera matrices. The shader applies all nessesary transformations (scaling, rotating and translating). The shader converts the vertices to homogeneous coordinates (vec4), i.e. position = [position 1]; See slimshady.vert

```
[Projection matrix] [World-to-camera matrix]
       \                      /
        \                    /
         \                  /
          \                /
           \              /
            \            /
             \          /
              \        /
-------------------------------------GPU----
                \    /
                 \  /
                  \/
            [Vertex shader]
```

Once in clip space, the fragment shader defines its appearence (i.e. colour).

## Clip space

After this plane projection we are in clip space. Here points have homogeneous (4D) coordinates.

## NDC space

After the projection is divided by Clip.W leaving us in Normalized Device Space (NDC). The resulting matrix has values between [-1, 1]. Values outside of this range are outside the clipping space. In OpenGL (c.f. Direct3D) this matrix is cubic. NDC coordinates are agnostic to screen shape and always [-1, 1].

## Rasterization

This step uses the view port and depth range to translate everything to fragment locations in screen/window space. This is done on the GPU.

## vis.draw

- vis.init
- vis.screen
- vis.loadLayerTextures
- vis.reloadLayerTextures

## Viewing model

- vis.planeProjection
- vis.uniSphereTriangles
- vis.quadToTriangles

## Layers

Current layers functions:

- gaussianLayer
- circLayer
- rectLayer
- crossLayer
- sinusoidLayer
- squareWaveLayer
- emptyLayer

```
%VIS.EMPTYLAYER Template texture layer for rendering in Signals
%  Returns a struct of paramters and their defaults used by VIS.DRAW to
%  load a visual stimulus layer.  If n > 1 a non-scalar struct is returned
%  of length n (default 1).
%
%  TODO Document viewAngle, texAngle and pos
%  @body There is currently no information on how these three parameters
%  are used by the viewing model.  For example, what is the practical
%  difference between `texOffset` and `pos`, or `viewAngle` and `texAngle`?
%
%  See also VIS.DRAW, VIS.RGBA

% Create an empty structure
layer = struct;
% SHOW a logical indicating whether or not the stimulus is visible
layer.show = false;
% TEXTUREID a char array used by VIS.DRAW to identify the texture layer.
% Layers with unique texture data (i.e. the data stored in rgba) must have
% unique IDs in order to be loaded into the buffer seperately.  Preceeding
% the ID with '~' indicates that it is a dynamic texture to be loaded anew
% each time. Dynamic textures are those where the underlying image array
% changes.
layer.textureId = [];
% POS
layer.pos = [0 0]';
% SIZE array of the form [azimuth altitude] defining the size of the
% texture in visual degrees
layer.size = [0 0]';
% VIEWANGLE The view angle in degrees
layer.viewAngle = 0;
% TEXANGLE the texture angle in degrees
layer.texAngle = 0;
% TEXOFFSET an array of the form [azimuth altitude] indicating the texture
% offset from the centre of the viewer's visual field in visual degrees
layer.texOffset = [0 0]';
% ISPERIODIC logical - when true the texture is replicated across the
% entire visual space
layer.isPeriodic = true;
% BLENDING char array defining the type of blending used.
% Options:
%  'none' (/ ''),
%  'source' (/ 'src'),
%  'destination' (/ 'dst'),
%  '1-source' (/'1-src')
layer.blending = 'source';
% MINCOLOUR & MAXCOLOUR arrays of the form [R G B A] indicating the min
% (max) intensity of the red, green and blue channels, along with the amout
% of opacity (alpha).  Values must be between 0 and 1.
layer.minColour = [0 0 0 0]';
layer.maxColour = [1 1 1 1]';
% COLOURMASK logical array indicating whether the red, green, blue and
% alpha channels may be written to the frame buffer.  When any of these
% channels are set to false no change is made to that component of any
% pixel in any of the color buffers, regardless of any changes to the
% texture image
layer.colourMask = [true true true true]';
% INTERPOLATION char array indicating the type of interpolation applied.
% Options:
%  'nearest' - Nearest neighbour interpolation
%  'linear' - linear interpolation
layer.interpolation = 'linear';
% RGBA Column array of uint8 RGBA values for each pixel (left to right, top
% to bottom) in the texture image. The values must be between 0 and 255.
% For example take a matrix.  See also VIS.RGBA
layer.rgba = [];
% RGBASIZE array of the form [m n] where m and n are the sizes of the first
% two dimentions of the texture image
layer.rgbaSize = [0 0]';
```

## Stimuli

## Images

The function for making image textures is vis.image. Images can be arrays with values between 0-1 (MATLAB-style) or 0-255. They may be monochromatic ([m,n,1]) or be RGB(A) ([m,n,3-4]). Images can be loaded in a few different ways. If you don't intend for the underlying image to change you can pass in a path to the image:

```
srcImg = which('cell.tif'); % Path to image
img = vis.image(t, srcImg);
% The source image may be a MAT file or an image file (tiff, png, etc.)
% If an alpha layer is present it will used.  This can be overridden by
% providing an alpha layer as a positional argument:
img = vis.image(t, srcImg, 1); % alpha may be scalar or array the size of img.
% If creating more than one visual element (e.g. you have two images you
% want to show at the same time) and are providing a source path, the names
% of the image files must be unique.  This is because the file name is used
% as the texture ID, which is ID used by Signals to distinguish textures.

% The source image may be loaded separately and passed in the same way:
images = load('imdemos.mat');
img = vis.image(t, images.circles);

% Finally the input may be a Signal whose value is the image array.  When
% the source image is a Signal it is loaded as a dynamic texture (the
% layer's textureId field starts with a '~').  This allows the source image
% to change throughout the experiment, however if you don't intend for your
% source image to change, consider pre-loading it like the above examples
% as it is more efficient.

% You can optionally add a Gaussian window over the image:
img.window = 'gauss';
img.sigma = [10 10];

% The image position and size may be set as expected:
img.dims = [40 20];
img.orientation = 180; % upside-down
img.azimuth = 0; % centred in x
img.altitude = 10; % slightly elevated

% The image may also be tiled across the screen by setting the repeat flag:
img.repeat = true; % cover the whole screen with image tiles
```

## Checker / sparse noise

## Gabor patch & gratings

## Shapes

## Dynamic textures

## vis.rgba & vis.rgbaFromUint8

```
%VIS.GRATING Returns a Signals grating stimulus defining a grating texture
%  Produces a visual element for parameterizing the presentation of a
%  grating. Produces a grating that can be either sinusoidal or
%  square-wave, and may be windowed by a Gaussian stencil, producing a
%  Gabor patch.
%
%  Inputs:
%    't' - The "time" signal. Used to obtain the Signals network ID.
%      (Could be any signal within the network - 't' is chosen by
%      convention).
%    'grating' - A char array defining the nature of the grating. Options
%      are 'sinusoid' (default) or 'squarewave'.
%    'window' - A char array defining the type of windowing applied.
%      Options are 'gaussian' (default) or 'none'.
%
%  Outputs:
%    'elem' - a subscriptable signal containing fields which parametrize
%      the stimulus, and a field containing the processed texture layer.
%      Any of the fields may be a signal.
%
%  Stimulus parameters (fields belonging to 'elem'):
%    'grating' - see above
%    'window' - see above
%    'azimuth' - the azimuth of the image (position of the centre pixel in
%     visual degrees).  Default 0
%    'altitude' - the altitude of the image (position of the centre pixel
%     in visual degrees). Default 0
%    'sigma' - if window is Gaussian, the size of the window in visual
%      degrees. Must be an array of the form [width height].
%      Default [10 10]
%    'phase' - the phase of the grating in visual degrees.  Default 0
%    'spatialFreq' - the spatial frequency of the grating in cycles per
%      visual degree.  Default 1/15
%    'orientation' - the orientation of the grating in degrees. Default 0
%    'colour' - an array defining the intensity of the red, green and blue
%      channels respectively. Values must be between 0 and 1.
%      Default [1 1 1]
%    'contrast' - the normalized contrast of the grating (between 0 and 1).
%      Default 1
%    'show' - a logical indicating whether or not the stimulus is visible.
%      Default false
%
%  See Also VIS.EMPTYLAYER, VIS.PATCH, VIS.IMAGE, VIS.CHECKER6, VIS.GRID


% Map the visual element signal through the below function 'makeLayers' and
% assign it to the 'layers' field.  When any of the above parameters takes
% a new value, 'makeLayer' is called, returning the texture layer.
% 'flattenStruct' returns the same texture layer but with all fields
% containing signals replaced by their current value. The 'layers' field
% is loaded by VIS.DRAW
```

## Notes

1. Like MATLAB OpenGl uses column-major order
2. Camera space may also be referred to as view space
3. In the unit cube, 1 means the object is at the far clipping plane (right up against the back-drop as it were) and -1, the near clipping plane (right up against the screen). All points visible to the camera have a negative z-component.
4. In OpenGL (GLUT more precisely), the FOV corresponds to the vertical angle
5. https://www.scratchapixel.com/lessons/3d-basic-rendering/perspective-and-orthographic-projection-matrix/projection-matrix-introduction

## Etc.

Author: Miles Wells

v0.0.1

```
%#ok<*NASGU,*NOPTS>
```

Published with MATLAB® R2019a
