# Supplementary material for: Rigbox: An Open-Source Toolbox for Probing Neurons and Behavior
Source: eNeuro. 2020 Jul 9;7(4):ENEURO.0406-19.2020. doi: 10.1523/ENEURO.0406-19.2020 (PMC7363478; doi:10.1523/ENEURO.0406-19.2020)
Supplement: Extended Data 1 — We recommend looking at and downloading the code directly from the github repository, at https://github.com/cortex-lab/Rigbox. Download Extended Data 1, ZIP file. [file enu-eN-OTM-0406-19-s02.zip › Rigbox-master-2020-05-18/Rigbox-master/docs/html/using_wheel.html]

Working with wheel data 

# Working with wheel data

In the Burgess wheel task a visual stimulus is yoked to LEGO wheel via a rotary encoder. Below are some things to consider when designing or modifying a wheel task. For information on setting up the rotary encoder, see Hardware Configuration: DAQ rotary encoder. For information on wiring a rotary encoder for the Burgess steering wheel task, see the Burgess hardware setup instructions .

## Contents

- The wheel input in Signals
- Load information about the wheel from the hardware file
- Load the wheel data
- Convert to linear displacement (cm)
- Convert to angular displacement (rad)
- Convert to angular displacement (RPM)
- Convert to angular displacement (deg)
- Convert to azimuth (visual degrees)
- Notes
- Etc.

## The wheel input in Signals

There are currently three wheel-related inputs used by the Signals Experiment class. These can be accessed via a subscripted reference(1) to the 'inputs' argument of an experiment definition function (expDef) :

1. wheel - the raw value of the rotary encoder, polled on every iteration of the main experiment loop. Each time the rotary encoder moves suffeciently it sends out a pulse. These are integrated by a counter channel and the output is seen in the wheel signal. This Signal is zero'd at the beginning of the experiment.
2. wheelMM - the wheel movement in units of centimetres linear displacement. That is the distance the wheel would have rolled along a flat surface. This Signal is zero'd at the beginning of the experiment.
3. wheelDeg - the wheel movement in degrees. This Signal is zero'd at the beginning of the experiment.

The wheelMM and wheelDeg signals simply map the values of wheel through a function based on information found in the hardware file's mouseInput object, namely the 'WheelDiameter' and 'EncoderResolution' properties.

## Load information about the wheel from the hardware file

For a given experiment you may wish to load the hardware used, and to view the settings for the rotary encoder. Each experiment, a JSON copy of the hardware file is saved to the main repository. This preserves the settings as they were at the time the experiment ran. The below code searches for this JSON file and tries to load it. If it doesn't exist, the current hardware file is loaded instead. Some information about the rotary encoder settings are the printed.

```
expRef = '2019-03-28_1_default'; % Example experiment
jsonPath = dat.expFilePath(expRef, 'hw-info', 'master', 'json');
if exist(jsonPath, 'file') % Check is hardware JSON exists
  % If the JSON file exists load that as the wheel may have sinced changed
  rig = jsondecode(fileread(jsonPath));
else
  % Otherwise load the existing harware file
  rigName = 'exampleRig';
  rig = hw.devices(rigName, false);
end

% Print some info:
D = rig.mouseInput.WheelDiameter;
res = rig.mouseInput.EncoderResolution;
a = rig.mouseInput.MillimetresFactor;
fprintf(['Details for experiment <strong>%s</strong>:\n'...
  'Wheel diameter (mm): %.1f, '...
  'encoder resolution: %d, '...
  'calculated millimetres factor: %.4f\n'], expRef, D, res, a)
```

## Load the wheel data

If availiable, load the auto-extracted ALF file as the data is quicker to load, in centimeters linear displacment units and resampled evenly at 1000Hz.

```
expPath = dat.expPath(expRef, 'main', 'master');
files = dir(expPath);
Fs = 1000; % Frequency to resample at
if any(endsWith({files.name}, 'wheel.position.npy'))
  fullFileFn = @(nm) readNPY(fullfile(expPath, endsWith({files.name}, nm)));
  pos = fullFileFn('wheel.position.npy'); % in cm
  rawT = fullFileFn('wheel.timestamps.npy'); % in sec
  vel = fullFileFn('wheel.velocity.npy'); % in cm/sec
  t = (rawT(1,2):1/Fs:rawT(2,2))';
else % Otherwise load from block file and preprocess
  data = dat.loadBlock(expRef);
  pos = data.inputs.wheelValues; % in samples
  tRaw = data.inputs.wheelTimes; % in sec
  % Resample values
  t = 0:1/Fs:tRaw(end);
  pos = interp1(tRaw, pos, t);
  % Correct for over-/underflow
  pos = wheel.correctCounterDiscont(pos);
end
```

## Convert to linear displacement (cm)

If the units are in samples (i.e. loaded from inputs.wheel or inputSensorPos), convert to units of centimetres linear displacement. That is the distance the wheel would have rolled along a flat surface.

```
posCM = (rig.mouseInput.MillimetresFactor/10) .* pos; %#ok<*NASGU>
% or alternatively
res = rig.mouseInput.EncoderResolution*4; % Resolution * 4 for '4X' encoders
D = rig.mouseInput.WheelDiameter/10; % Converted to cm from mm
posCM = pos./res * pi * D;

% The velocity is the derivative of this, so it's the tangential velocity
% in cm/sec
smoothSize = 0.03; % Gaussian smoothing window
[vel, acc] = wheel.computeVelocity2(pos, smoothSize, Fs);
```

## Convert to angular displacement (rad)

For angular displacement / velocity, just divide by the wheel radius

```
posRad = posCM / 0.5*D; % in radians
velAng = vel / 0.5*D; % in rad/sec
```

## Convert to angular displacement (RPM)

Convert this to the more intuitive revolutions per minute:

```
RPM = velAng*60 / 2*pi;
```

## Convert to angular displacement (deg)

For displacement in degrees:

```
posDeg = rad2deg(posRad);
velDeg = rad2deg(velAng);
% or...
posDeg = pos / res*360;
```

## Convert to azimuth (visual degrees)

If you know the response threshold in visual degrees, you can convert this to visual degrees.

```
thresh = 35; % visual degrees azimuth
% Position relative to interactive on
pos = pos - pos(1);
% Distance moved in whatever units
dist = diff([pos(1) pos(end)]);
% Convert to visual degrees moved, assuming correct is an element of [-1 0
% 1]
posAzi = (pos/abs(dist) * thresh) - (sign(dist) * sign(correct) * thresh);
velAzi = (vel/abs(dist) * thresh) - (sign(dist) * sign(correct) * thresh);
```

## Notes

(1) e.g. 'inputs.foo'. This is know as dot notation. More info here .

## Etc.

Author: Miles Wells

v1.0.0

Published with MATLAB® R2019a
