# Supplementary material for: Rigbox: An Open-Source Toolbox for Probing Neurons and Behavior
Source: eNeuro. 2020 Jul 9;7(4):ENEURO.0406-19.2020. doi: 10.1523/ENEURO.0406-19.2020 (PMC7363478; doi:10.1523/ENEURO.0406-19.2020)
Supplement: Extended Data 1 — We recommend looking at and downloading the code directly from the github repository, at https://github.com/cortex-lab/Rigbox. Download Extended Data 1, ZIP file. [file enu-eN-OTM-0406-19-s02.zip › Rigbox-master-2020-05-18/Rigbox-master/docs/html/websocket_config.html]

Introduction 

# Introduction

Once the hardware files are set up for srv.expServer and mc, it is necessary to configure the websockets so that the two computers can connect to one another. This connection serves a few purposes:

1. To start and stop experiments via the MC GUI (currently the only way to do so).
2. To monitor the experiment, e.g. the current parameters, trial number, reward total, etc.
3. To send an Alyx instance so that the stimulus computer can register its files to the database.

## Contents

- Configuring WebSockets
- Ports
- Experiment delays
- Saving & loading
- Using Websockets
- - Server
- - Client
- - Close connections
- Stimulus Controller
- expServer messages
- - signals
- - status
- - AlyxRequest
- Schematic
- UDP communication & Services
- Debugging
- - Stimulus server
- - MC computer
- - Clean up
- Etc.

## Configuring WebSockets

This section demonstrates how to configure WebSockets so that MC can connect to the Stimulus computer. See this guide if you wish to configure things for running mc and srv.expServer on the same computer (not recommended).

```
% The stimulus controllers are loaded from a MAT file with the name
% 'remote' in the globalConfig directory, defined in dat.paths:
p = fullfile(getOr(dat.paths, 'globalConfig'), 'remote.mat');

% Let's create a new stimulus controller
name = ipaddress(hostname);
stimulusControllers = srv.StimulusControl.create(name);

% A note on adding new computers.  Do not simply copy objects, instead use
% the following method:
uri = 'ws://192.168.0.1:5000';
stimulusControllers(end+1) = srv.StimulusControl.create('rig2', uri);
```

## Ports

```
% Note that the DefaultPort property is only used when the uri provided
% doesn't already have a port (':5000' in the example above):
stimulusControllers(end+1) = srv.StimulusControl.create('ZSCOPE');
stimulusControllers(end).Uri % 'ws://ZSCOPE:2014'

stimulusControllers(end).DefaultPort = 4820;
stimulusControllers(end).Uri % 'ws://ZSCOPE:4820'

% However as we set the uri for 'rig2' above including a port, the
% DefaultPort property isn't used:
rig2 = strcmp('rig2', {stimulusControllers.Name});
stimulusControllers(rig2).DefaultPort % *2014
stimulusControllers(rig2).Uri % 'ws://192.168.0.1:5000'
```

The port set in the URI of a rig's StrimulusControl object must be the same as the port used by that rig's srv.expServer. When expServer is started it listens on the default port (normally 2014). If you wish to set a port that is different to the default port, you must save a Communicator object into the rig's hardware file and set it there. Below is some code for setting the default port to 3000 on a Stimulus Computer:

```
% Create a communicator object (var name must be 'communicator')
communicator = io.WSJCommunicator.server(3000);

% Save into the hardware file
hardware = fullfile(getOr(dat.paths, 'rigConfig'), 'hardware.mat');
save(hardware, 'communicator', '-append') % append to the hardware file
```

## Experiment delays

Setting default delays is sometimes useful. These can also be changed in the 'rig options' dialog in MC. The ExpPreDelay property is the time in seconds to wait between starting the services and initializing the experiment object, and actually beginning the experiment (for a Signals experiment this means updating the 'events.expStart' signal). Hence if the pre-delay is 5 seconds then the inputs, parameters and time signals are updated in the main loop for 5 seconds before the first trial officially begins. This can be useful when an auxiliary recording device takes some time to initialize, or if you want to record some sort of baseline activity before the first stimulus appears. It also ensures that everything is running smoothly before the first trial (sometimes there are suspect timings on the first propogation through the network).

```
stimulusControllers(end).ExpPreDelay = 10; % Initialize then wait 10s

% Likewise the ExpPostDelay is the time in seconds between the experiment
% ending (i.e. the events.expStop signal updating), and the stop command
% being sent to the services.  The main loop still runs during this time
% and values continue to be posted to the input and timing signals, however
% no trial signals will update.  This may be useful if you wish to record
% some baseline after the stimulus presentation has ended for example.
%
% Trigger ExperimentEnded event, wait 30s then trigger the
% ExperimentCleanup event:
stimulusControllers(end).ExpPostDelay = 30;
```

## Saving & loading

```
% Save your new configuration.  Note the variable name must be as below:
save(p, 'stimulusControllers')

% The stimulus controllers can be loaded using the srv.stimulusControllers
% function.  If no remote file exists, a default StimulusControl object is
% returned.
sc = srv.stimulusControllers;
```

## Using Websockets

The stimulusControllers list will appear in the 'rig' drop-down list in MC, allowing you to choose which one to connect to and where to start an experiment. In this way you can manage multiple experiments from one computer.

The Websockets are set up automatically when you run MC and srv.expServer, however you can use these for your own code if you wish. Below is some information on using the Websockets and the +io package.

The srv.StimulusControl object builds on the io.WSJCommunicator object by adding extra events and useful properties such as the Status property. Let's look at the lower level classes first:

One computer should be running as a server and another as a client. The relationship is many-to-one in that a client may connect to only one server at a time, while a server may broadcast to any number of clients. Hence an number of MC computers (clients) can listen for experiment updates from a particular rig (server).

You can test this class by running the two on the same computer. Thi must be done in two different instances of MATLAB.

## - Server

```
server = io.WSJCommunicator.server()

server.open() % Start listening on default port 2014
server.wtf % Get status of socket, e.g. OPEN

% When EventMode is set to false (default), the message is kept in the
% buffer.  Checking for messages can be done by looking at the
% IsMessageAvailable property:
server.IsMessageAvailable
% If a message is availiable it may be accessed with the receive method:
[msgId, data, host] = server.receive;

% When EventMode is set to true, new messages notify any MessageReceived
% listeners.  This will the callbacks are evaluated with the source
% variable being the io.WSJCommunicator object and the event variable being
% a structure with the fields Id, Data and Sender.
server.EventMode = true; % Allows us to create callback listeners
callback = @(~,evt) sprintf(...
  'Message with id %s from %s with the following data %s', ...
  evt.Id, evt.Sender, toStr(evt.Data));
el = event.listener(server, 'MessageReceived', callback); % Display message ids
```

## - Client

```
ip = ipaddress; % This computer's IP
client = io.WSJCommunicator.client(ip)

client.open() % Open the connection
client.wtf % OPEN
client.WebSocket.isOpen % Similar status but as a bool
client.send('test message', randi(100,1,5))
% Sending uses the function hlp_serialize
```

## - Close connections

```
client.close()
server.close()
```

## Stimulus Controller

The srv.StimulusControl class methods are pretty self explanatory...

- create - As seen above this is the constructor
- connect - Bind web socket. Calls io.WSJCommunicator/open()
- disconnect - Unbind. Sends 'goodbye' message then calls io.WSJCommunicator/close()
- startExperiment - Called with an expRef and, optionally, an Alyx instance. Sends both to the remote host with the message id 'run'
- quitExperiment - Called with a flag to indicate an abort or not. If true the experiment 'aborts', otherwise it's end status is set to 'end'. Currently the behaviours of these two are identical. It may be that in the future this flag will determine whether expServer waits for the trial to end before quitting. Sends 'quit' message id

## expServer messages

When a new messages arrives it is expected to be one of the IDs mentioned below. These usually leads to listeners to one of the events being notfied. The listener callbacks are called with an event object of the class srv.ExpEvent, which are like regular event objects but have three properties: Name, Ref and Data, which may be used by the callback functions.

The following IDs are expected:

## - signals

This id means an experiment update from a currently running signals object (exp.SignalsExp) has arrived. Listeners to the ExpUpdate event are notified with a 'signals' ExpEvent. In MC the listeners to this are objects of the eui.ExpPanel class, e.g. eui.SignalsExpPanel. These panel objects plot and display the update data.

## - status

Messages with this id are usually sent before and after an experiment or in the case of 'update' messages, they indicate a new phase of the experiment (in legacy experiments such as exp.ChoiceWorld only. The data object is expected to be a cell array with at least two elements. The first is the status, which may be one of the following:

1. starting - Indicates that expServer received the message and is able to begin the experiment. data{2} contains the expRef of the experiment. Listeners of the ExpStarting event are notified with a 'starting' ExpEvent.
2. completed - Indicates that the experiment stopped without any exceptions. data{2} contains the expRef of the experiment. Listeners of the ExpStopped event are notified with a 'completed' ExpEvent.
3. expException - Indicates that the experiment stopped because of an uncaught error. data{2} contains the expRef of the experiment; data{3} contains the error message from the MException object. Listeners of the ExpStopped event are notfied with an 'exception' ExpEvent. The event object's Data field contains the error message.
4. update - Indicates a new phase of the experiment. data{2} contains the expRef of the experiment; data{3} contains a cell array. This may be the name of the new phase (e.g. 'feedback', 'interactive'), in which case listeners of the ExpUpdate event are notified with an 'update' ExpEvent. One special case is when data{3} contains the string 'event', iindicating that the experiment has finished initializing and has now officially started (sent after the experiment pre-delay has ended). In this case the ExpUpdate event listeners are notified with a 'started' ExpEvent.

## - AlyxRequest

expServer requested the AlyxInstance. Data = an experiment reference string. The object will return whatever is in the AlyxInstance property at that time and notify listeners of the AlyxRequest event.

## Schematic

Below is a schematic of the messages between MC and srv.expServer. The words within the dashed lines are the message ids. The works in brackets to the right of the '<<' arrows are the messages / data that are sent alongside the message id. The words on the left in brackets are the actions that cause, or are a response to, a message.

```
%       MC                                           srv.expServer
% -------------------------------------------------------------------------
% [rig selected]-------------status------> +    [check if exp running]
%                                          |               | <false>
%       + <--status------------------------+ <<         [idle]
%       |
%    [begin]-------------------------run-->+    [check idle; params valid]
%                                          |        <true> | <false>
%       + <--status------------------------+ <<    [starting / fail]
%       |                                  |               |
% [create panel]                           |    [exp init; start services]
%                                          |               |
%       + <--event-------------------------+ <<     [experimentInit]
%       |                                  |
% [notify user]                            |
%                                          |
%       + <--signals-----------------------+      [signals events updated]
%       |                                  |
% [update plots] <--signals----------------+      [signals events updated]
%
% [rig selected]-------------status------> +      [check if exp running]
%                                          |        <true> | <false>
%       + <--status------------------------+ <<     [running / idle]
%       |
%  [notify user]
%
%  [end / abort]-------------------quit--> +           [run quit]
%                                                          |
%                                                 [check alyx token valid]
%                                                   <true> | <false>
%       + <--AlyxRequest-------------------+ <<    [<do nothing> / expRef]
%       |
% [send new token]---updateAlyxInstance--> +        [register files]
%
%       + <--status------------------------+ <<       [completed]
%       |
% [notify user]
%
%   [quit mc]-------------------goodbye--> +       [log: disconnected]
```

## UDP communication & Services

You can also add a list of auxiliary service ids to the Services property of your StimulusControl objects. This list will show up in the 'rig options' dialog in MC, allowing you to select which services to activate for a given experiment. For information on controlling auxiliary software devices during an experiment see using\_services

## Debugging

University networks are often quite complicated and operating within various firewalls and workgroups or within an intranet can cause problems. These Websockets just use a basic TCP/IP protocol and therefore you (or your IT administrator) should be able to diagnose any issues with the below information.

If you can get your computers to show up in each other's Windows network list then there's a very good chance the Websockets will work. Sometimes Windows fails to resolve a given hostname (i.e. computer name) so trying the IP address first is more reliable. You can find your computer's IP address by searching 'what is my IP' online.

Sometimes the defualt port of 2014 is in use by another program, in which case you may see the following error:

```
Error using io.WSJCommunicator/startClient (line 194)
Could not connect to 'ws://128.40.198.177:2014'
```

Try setting a different port (anthing between 1024-49151 should be safe). This is done by either adding/changing the port to the end of the Uri property, after a colon, or by setting the DefaultPort property. The latter is only used in the Uri doesn't already have a port.

Sometimes when you can't connect to a remote computer it's because the remote computer failed to start the connection. When this happens the following may be printed to the command window:

```
onError: java.net.BindException: Address already in use: bind
```

This usually means that the previous time you opened a socket it was not closed properly. Restarting MATLAB and/or clearing java may resolve the issue.

## - Stimulus server

```
clear all; clear java % Make sure everything is cleared
system('netstat -nao | find ":2014"'); % Should return nothing
com = io.WSJCommunicator.server; % Create server object
com.EventMode = true; % Allows us to create callback listeners
com.open() % Start listening on default port 2014
el = addlistener(com, 'MessageReceived', @(~,msg)disp(msg.Id)); % Display message ids

system('netstat -nao | find ":2014"');
% Example:
%  TCP    0.0.0.0:2014           0.0.0.0:0              LISTENING       26756
%  TCP    [::]:2014              [::]:0                 LISTENING       26756
```

## - MC computer

```
clear all; clear java % Make sure everything is cleared
system('netstat -nao | find ":2014"'); % Should return nothing
% Check this matches exactly to computer name in
% Control Panel --> All Control Panel Items --> System
com = io.WSJCommunicator.client('CSSD901341') ;
open(com) % Establish commection with CSSD901341

system('netstat -nao | find ":2014"');
% Example:
%  TCP    128.40.198.140:2014    128.40.198.162:49458   ESTABLISHED     6640

com.send('hello', []) % Should display 'hello' on stimulus computer
```

## - Clean up

```
close(com); delete(com); clear all; clear java
```

## Etc.

Author: Miles Wells

v1.0.1

```
%#ok<*NOPTS,*ASGLU,*NASGU,*CLJAVA,*CLALL>
```

Published with MATLAB® R2019a
