# Supplementary material for: Rigbox: An Open-Source Toolbox for Probing Neurons and Behavior
Source: eNeuro. 2020 Jul 9;7(4):ENEURO.0406-19.2020. doi: 10.1523/ENEURO.0406-19.2020 (PMC7363478; doi:10.1523/ENEURO.0406-19.2020)
Supplement: Extended Data 1 — We recommend looking at and downloading the code directly from the github repository, at https://github.com/cortex-lab/Rigbox. Download Extended Data 1, ZIP file. [file enu-eN-OTM-0406-19-s02.zip › Rigbox-master-2020-05-18/Rigbox-master/README.pdf]

Rigbox

custom badge inaccessible custom badge inaccessible

NOTE Due to space constraints on eNeuro, this version of the repository is missing the `/docs/reqs/` folder, which contains installers for Rigbox's software dependencies. In order to run the quick installation set-up via the `installRigboxReqs` MATLAB function, you *must* have this `reqs/` folder, which can be found on the Rigbox's online github repository. Or, you can proceed to install via the longer, detailed instructions below.

Rigbox is a high-performance, open-source MATLAB toolbox for managing behavioral neuroscience experiments. Rigbox's main goals are to simplify hardware/software interfacing, behavioral task design, and visual and auditory stimuli presentation. Additionally, Rigbox can time-align datastreams from multiple sources and communicate with a remote database to manage experiment data. Rigbox is mostly object-oriented and highly modular, which simplifies the process of designing experiments. For detailed information, see the publication.

Requirements

For exploring Rigbox's features and running test experiments, Rigbox only needs to be installed on a single computer.

For running experiments, Rigbox should be installed on two computers: one computer (which we refer to as the "Stimulus Computer" or "SC") communicates with an experiment rig's hardware and presents stimuli, and the other computer (which we refer to as the "Master Computer" or "MC") runs a GUI that the experimenter can use to start, monitor, parameterize, and stop the experiment.

Hardware

Below are the **minimum** computer hardware specs: * CPU: 4 logical processors @ 3.0 GHz base speed (e.g. Intel Core i5-6500) * RAM: DDR4 16 GB @ 2133 MHz (e.g. Corsair Vengeance 16 GB) * GPU: 2 GB @ 1000 MHz base and memory speed (e.g. NVIDIA Quadro P400)

For most experiments, typical, contemporary, factory-built desktops running Windows 10 with dedicated graphics cards should suffice. Specific requirements of a SC will depend on the complexity of the experiment. For example, running an audio-visual integration task on multiple screens will require quality graphics and sound cards. SCs may additionally require an i/o device to communicate with external rig hardware, of which currently only National Instruments Data Acquisition Devices (NI-DAQs, e.g. NI-DAQ USB 6211) are supported.

Software

Below are the **minimum** computer software dependencies that must be installed before installing Rigbox:

- OS: 64 Bit Windows 10
- Libraries: Visual C++ Redistributable Packages for Visual Studio 2013 & 2015
- MATLAB: 2018b or later, including the Data Acquisition Toolbox
- Community MATLAB toolboxes:
  - GUI Layout Toolbox (v2 or later)
  - Psychophysics Toolbox (v3 or later)

Similar to the hardware requirements, software requirements for a SC will depend on the experiment: if acquiring data through a NI-DAQ, the SC will additionally require the MATLAB NI-DAQmx support package.

Installation

Before starting, ensure you have read and installed the above requirements. You can also install these requirements by running the `installRigboxReqs` function in MATLAB, and following the instructions that appear in the MATLAB command window.

Below we provide brief instructions for installing Rigbox via Git. These instructions explain how to 1) clone the repository from GitHub to your computer, and 2) appropriately add Rigbox to MATLAB's paths. For a detailed installation guide, including installing Rigbox's software requirements, see here.

1. In your git terminal, run:

```
git clone --recurse-submodules https://github.com/cortex-lab/Rigbox
```

2. In MATLAB, navigate to the Rigbox root directory (where Rigbox was cloned), and run: `addRigboxPaths()`, OR `addRigboxPaths('SavePaths', false)` if you don't want to save the paths for future MATLAB sessions. (*Note*: Do **not** manually add all Rigbox folders and subfolders to the paths.)

Getting started

To ensure the installation completed successfully, try running the example experiments from the Rigbox paper.

Rigbox uses the *Signals* framework for programatically designing and running behavioral tasks. See the *Signals* docs for more information on *Signals* and how to run example test experiments on a single computer via Rigbox's `+eui/SignalsTest.m` GUI.

(The above is an example of running the `signals/docs/examples/exp defs/advancedChoiceWorld.m` file in the `+eui/SignalsTest.m` GUI.)

Rigbox's online documentation, including detailed set-up and usage guides for running experiments on a MC and SC, can be found at cortex-lab.github.io/Rigbox.

Updating the code

With Git it's very easy to keep the code up-to-date. We strongly recommend regularly updating Rigbox and its submodules by running the following git command

(within the Rigbox directory):

```
git pull --recurse-submodules
```

Contributing

If you experience a bug or have a feature request, please report it via github Issues. For details on contributing code and our code of conduct, please see our contributing page.

Authors & Accreditation

Rigbox was created by Chris Burgess in 2013, initially developed to probe mouse behavior for the Steering Wheel Setup. It is now maintained and developed by Miles Wells (miles.wells@ucl.ac.uk), Jai Bhagat (j.bhagat@ucl.ac.uk) and a number of others at CortexLab. See also the full list of contributors.

Please cite the Rigbox publication appropriately in publications which use Rigbox to run behavioral tasks and/or acquire data.

Acknowledgements

- GUI Layout Toolbox for code pertaining to Rigbox's UI
- Psychophysics Toolbox for code pertaining to visual stimulus presentation
- NI-DAQmx for code pertaining to inerfacing with a NI-DAQ device
- TooTallNate for code pertaining to using Java Websockets
- Timothy E. Holy for the `distinguishable_colors` function
